# Room temperature catalytic carbon–hydrogen bond alumination of unactivated arenes: mechanism and selectivity†
†Electronic supplementary information (ESI) available: Experimental procedures, details of the DFT studies, single crystal X-ray data and multinuclear NMR spectra. CCDC X-ray crystallographic data for **Pd_2_Al_2_**, **PdAl_2_**, **3a** and **4-*o*** (CIF) 1829418–1829421. For ESI and crystallographic data in CIF or other electronic format see DOI: 10.1039/c8sc02072h


**DOI:** 10.1039/c8sc02072h

**Published:** 2018-05-29

**Authors:** Thomas N. Hooper, Martí Garçon, Andrew J. P. White, Mark R. Crimmin

**Affiliations:** a Department of Chemistry , Imperial College London , South Kensington , London , SW7 2AZ , UK . Email: m.crimmin@imperial.ac.uk

## Abstract

We report the first catalytic methods for the transformation of C–H bonds of unactivated arenes into C–Al bonds. The catalytic reactions occur at 25 °C (benzene, toluene and xylenes) with palladium loadings as low as 0.1 mol%. Remarkably, the C–H activation of toluene and xylenes proceeds with *ortho*- and *meta*-selectivity. This selectivity is highly unusual and complementary to both Friedel–Crafts and the majority of C–H borylation methods. Through a detailed mechanistic analysis (Eyring analysis, KIE, DFT, QTAIM) we show that unusual Pd–Al intermetallic complexes are on the catalytic cycle and that the selectivity is determined by weak attractive dispersion forces in the transition state for C–H bond breaking.

## Abstract 2

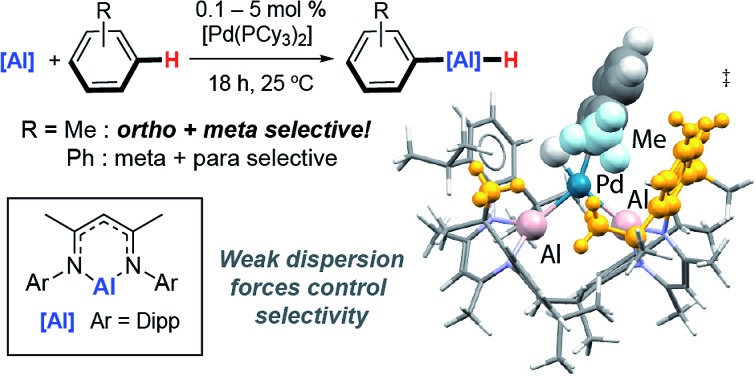
We report the first catalytic methods for the transformation of C–H bonds of unactivated arenes into C–Al bonds.

## Introduction

Catalytic methods that selectively break C–H bonds in organic molecules are now considered indispensable in synthesis. The activation and functionalisation of inert C–H bonds in arenes from oil refineries, such as benzene, toluene and xylenes, is arguably one of the most challenging facets of this field.^1^,^2^ Nevertheless a number of homogeneous catalysts are now known that result in carbon–carbon or carbon–heteroatom bond formation from inert C–H bonds of simple aromatic hydrocarbons.^3^ Of particular relevance to this study are methods that generate C–B bonds from C–H bonds.

**Fig. 1:**
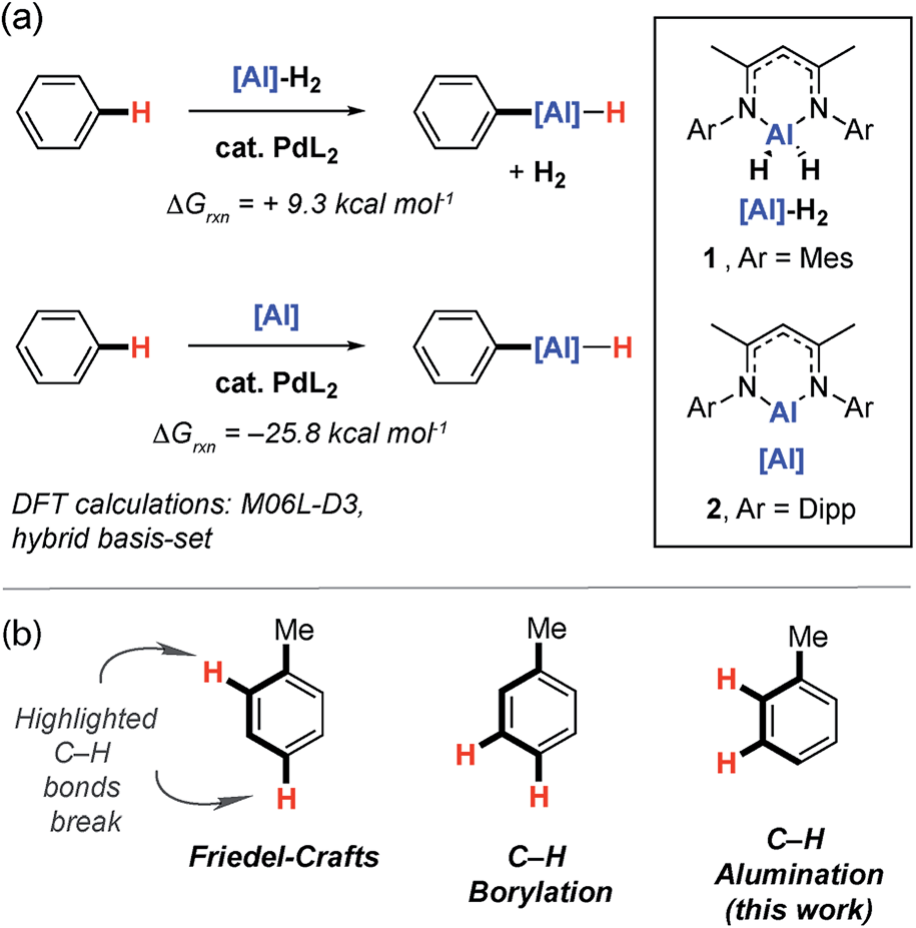
(a) Catalytic C–H alumination of arenes with Al(iii) and Al(i) reagents. (b) Regiocontrol of C–H functionalisation.

Originally documented in 1999,^4^ the catalytic C–H borylation of arenes can be traced back to careful stoichiometric studies in which transition metal boryl complexes were shown to react with aromatic solvents.^5^,^6^ The newly formed products contain reactive C–B bonds ripe for further synthetic exploitation.^7^,^8^ The importance of the work is clear from the rapid development of new catalysts for these transformations and diverse applications of the boron-containing products in synthesis.^9^–^11^ In the absence of a functional group that coordinates the catalyst, the regioselectivity of C–H borylation is, in most cases, dictated by steric factors. For mono-substituted arenes, the majority of known catalysts are selective for the formation of *meta* and *para* functionalised products (Fig. 1).^12^–^14^ Bulky iridium catalysts have been developed which can bias the system toward the *para*- rather than the *meta*-selective pathway.^15^ Challenges still remain in this field and catalysts that are selective for the borylation of the *ortho* C–H bonds of arenes, in the absence of a directing group, are rare. Chatani and co-workers have reported platinium-based catalysts bearing N-heterocyclic carbene ligands for the C–H borylation of sterically inaccessible positions of arenes.^16^ Substrates include toluene, xylenes and mesitylene and while significant amounts of the *ortho*-borylation products are observed in these reactions, the *meta*-isomer is the dominant product.

Aluminium compounds are applied on surprising scales in chemical manufacture. For example, modern variants of the Aufbau reaction,^17^,^18^ such as the Ineos (Ethyl) Process^19^ and Chevron Phillips Chemical Company (Gulf) Process,^20^ rely on the oligomerisation of ethylene with triethylaluminium to produce long-chain alcohols or α-olefins in plants that operate on scales of ∼250 000–500 000 metric tons per annum. In addition, LiAlH_4_ is used on modest scales for the preparation of intermediates in the manufacture of pharmaceuticals.^21^ Despite aluminium being the most abundant metal in the Earth's crust,^22^ reactions that transform C–H bonds into C–Al bonds are rare and often limited to substrates containing a suitable directing group or activated, acidic C–H bonds.^23^–^26^ During the preparation of this manuscript, Aldridge, Goicoechea and co-workers reported a nucleophilic aluminyl anion which was shown to react with benzene to effect the transformation of a C–H to C–Al bond. This remarkable transformation is currently limited to a single substrate.^27^

While investigating the mechanism of a palladium-catalysed transformation of C–H bonds of heteroarenes and fluoroarenes into C–Al bonds,^28^ we discovered some unusual Pd–Al complexes capable of breaking the C–H bond of benzene at 25 °C. Herein we document these complexes and show that they catalyse the C–H alumination of unactivated arenes. New catalytic reactions have been developed using both Al(i) and Al(iii) dihydride reagents. Remarkably the reactions of the Al(i) reagent proceed primarily with *ortho*- and *meta*-selectivity, indicative of a new mode of regiocontrol that is complementary to both C–H borylation and Friedel–Crafts methodology (Fig. 1).^29^

## Results and discussion

### C–H activation of benzene with Pd–Al intermetallics

**Fig. 2:**
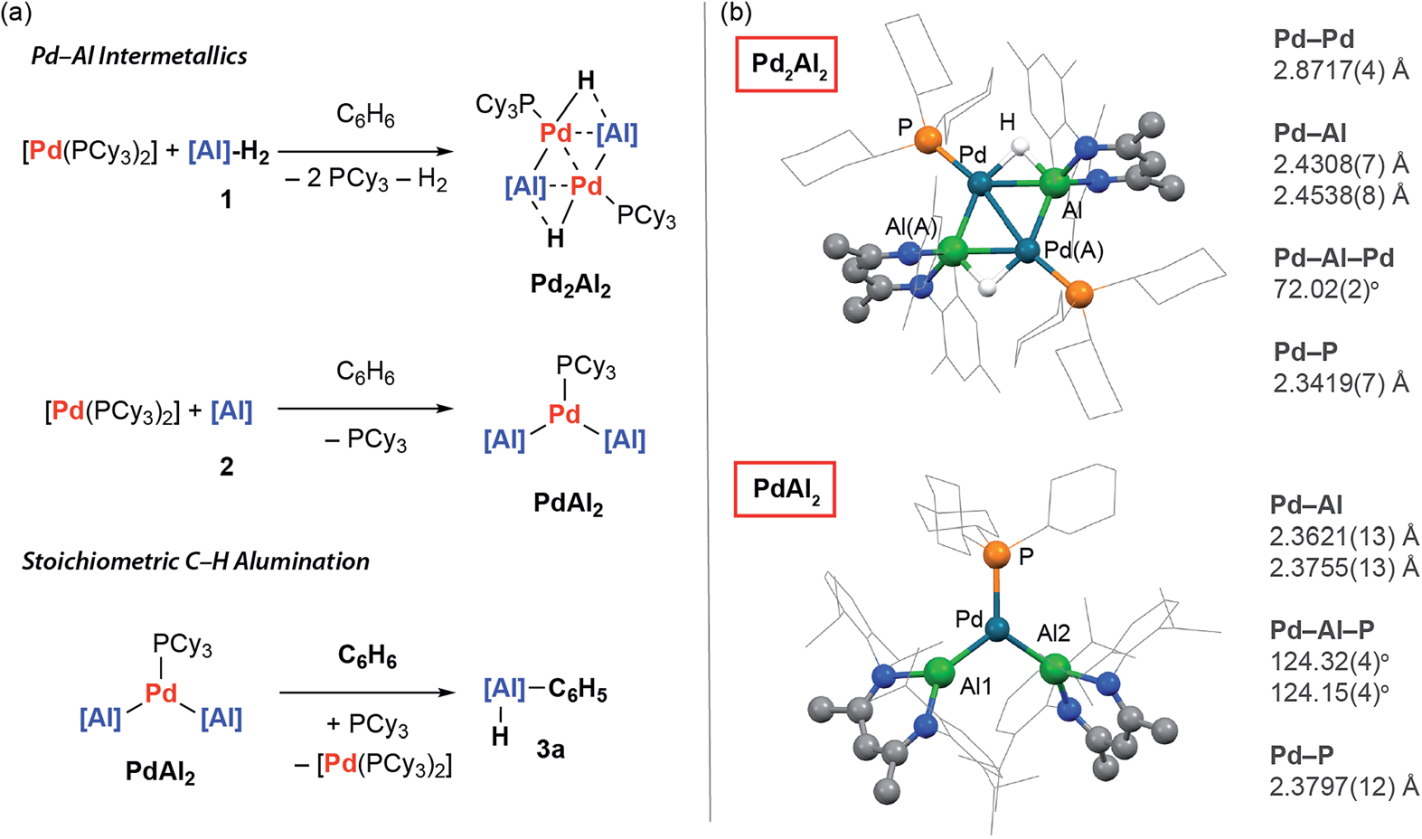
(a) Preparation and reactivity of Pd···Al intermetallics **Pd_2_Al_2_** and **PdAl_2_**. (b) The crystal structures of **Pd_2_Al_2_** and **PdAl_2_**.

Reaction of 1 equiv. of the aluminium dihydride **1** and 1 equiv. of [Pd(PCy_3_)_2_] in benzene led to isolation of the tetrametallic complex **Pd_2_Al_2_**. X-ray diffraction experiments on single crystals of **Pd_2_Al_2_** revealed a structure with a planar Pd_2_Al_2_ motif with two hydrides bridging the Pd–Al edges. The Pd···Pd and Pd–Al distances are both short (Fig. 2). The geometry of the metallic core is similar to {Pd_2_Si_2_H_2_} and {Pd_2_Ge_2_H_2_} complexes reported by Osakada and co-workers and a {Rh_2_Al_2_H_2_} intermetallic recently reported by our group.^30^–^32^ While comparison to these latter species allows a tentative assignment of the formal oxidation states in **Pd_2_Al_2_** as Pd(i) and Al(i), regardless of the interpretation it is clear that the formation of this complex requires a partial dehydrogenation of **1** and proceeds with liberation of H_2_.

The on-metal dehydrogenation of **1** with [Pd(PCy_3_)_2_] led us to speculate whether the formation of low-valent Al(i) species^33^ might be important in our previously reported methodology.^28^ The reaction between two equiv. of **2** and one equiv. of [Pd(PCy_3_)_2_] in cyclohexane solution at 25 °C gave a dark green solution from which black crystals of **PdAl_2_** could be isolated. In the solid state, the trigonal planar Pd(0) centre of **PdAl_2_** is coordinated by one phosphine and two aluminylene ligands. The Pd–Al distances are similar to previously reported Pd–Al(i) compounds (Fig. 2).^34^–^38^

Although performing the same reaction in benzene led to the initial formation of **PdAl_2_** this species was unstable, ultimately effecting C–H activation of the solvent and the clean formation of **3a**. The formation of **3a** occurred with regeneration of [Pd(PCy_3_)_2_] and was unambiguously confirmed by X-ray diffraction of a single crystal grown from the reaction mixture.

### Catalytic C–H alumination of benzene

**Fig. 3:**
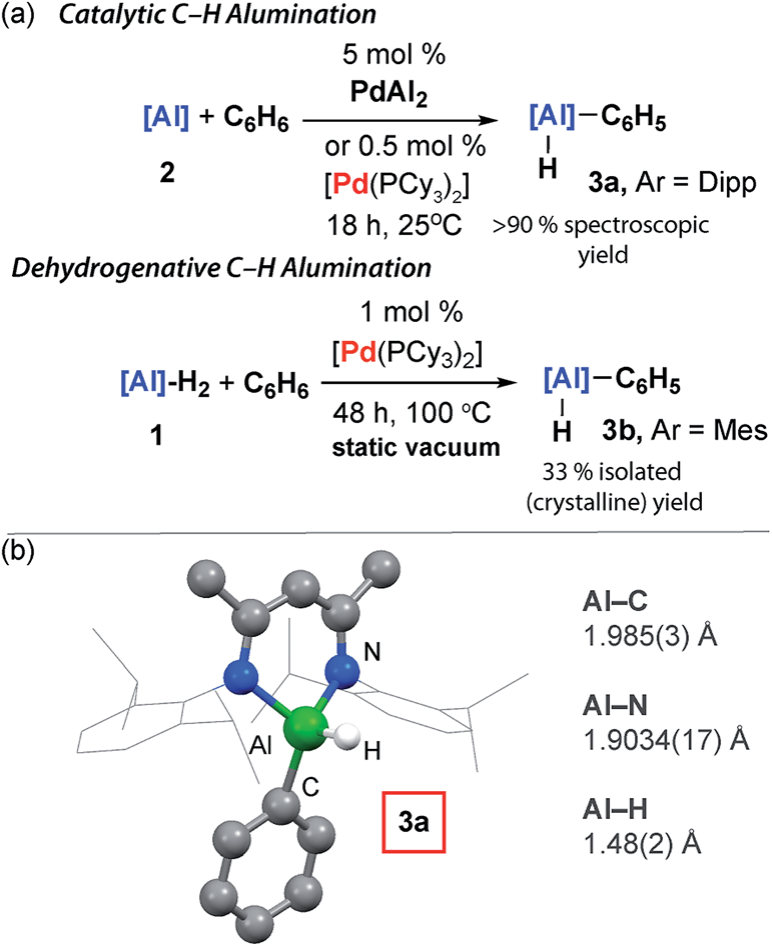
(a) Catalytic C–H alumination of C_6_H_6_. (b) The crystal structure of **3a**.

[Pd(PCy_3_)_2_] could be used catalytically and the formation of **3a** still occurred at 25 °C with 5 mol% (18 h, 90%), 0.5 mol% (18 h, 97%), and 0.1 mol% (3 d, 99%) loading (Fig. 3). While the C–H activation of benzene by addition across a metal–metal bond of a Ni–Al intermetallic has been previously reported, this reaction is not catalytic in nickel as the aluminium containing product remains coordinated to the transition metal.^39^ The presence of a catalytic quantity of [Pd(PCy_3_)_2_] is crucial for C–H alumination. No reaction occurs between **2** and benzene in the absence of a catalyst.^40^–^42^

The utility of the C–H alumination protocol was further expanded by demonstrating that the preparation of low-valent Al(i) complexes is not a requirement for catalysis. Hence, the reaction of the aluminium dihydride **1** with benzene can be catalysed by 1 mol% [Pd(PCy_3_)_2_], and after 48 h at 100 °C under static vacuum **3b** can be isolated as a crystalline solid in 33% yield (Fig. 3). This reaction is endergonic based on DFT calculations and removal of the liberated H_2_ is a requirement for catalytic turnover.

### Preliminary scope and regioselectivity

**Fig. 4:**
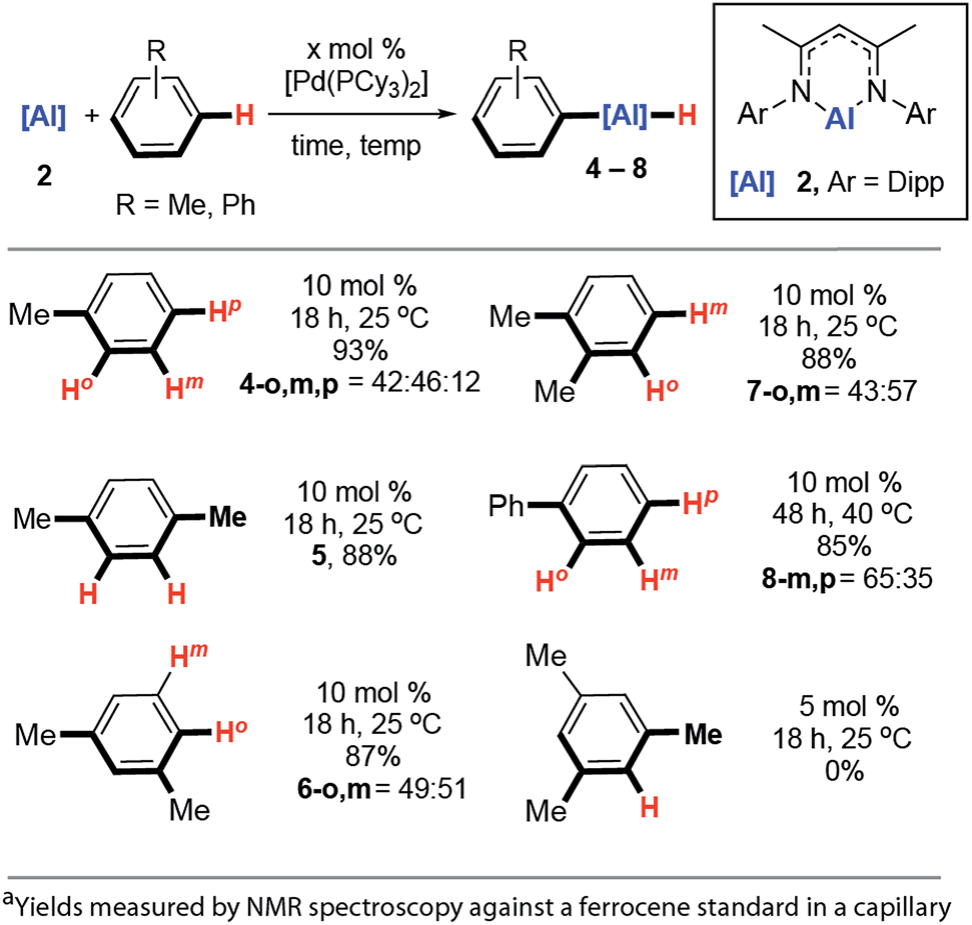
Scope of the Pd-catalysed C–H alumination of arenes.

The reaction scope was expanded. Reaction of **2** with toluene at 25 °C catalysed by 10 mol% [Pd(PCy_3_)_2_] produced a mixture of aluminium compounds arising from C–H activation at regioisomeric positions *ortho*-, *meta*- and *para*- to the methyl group (Fig. 4, **4-*o*,*m*,*p***). The selectivity of this reaction is notable and C–H alumination of toluene proceeds to form only negligible amounts (12%) of the *para*-isomer, and approximately equal amounts of *ortho*- (42%) and *meta*-isomers (46%). The vast majority of *ortho*-selective C–H borylation protocols rely on a suitable heteroatom containing functional group to direct the catalyst to the adjacent C–H bond.

**Fig. 5:**
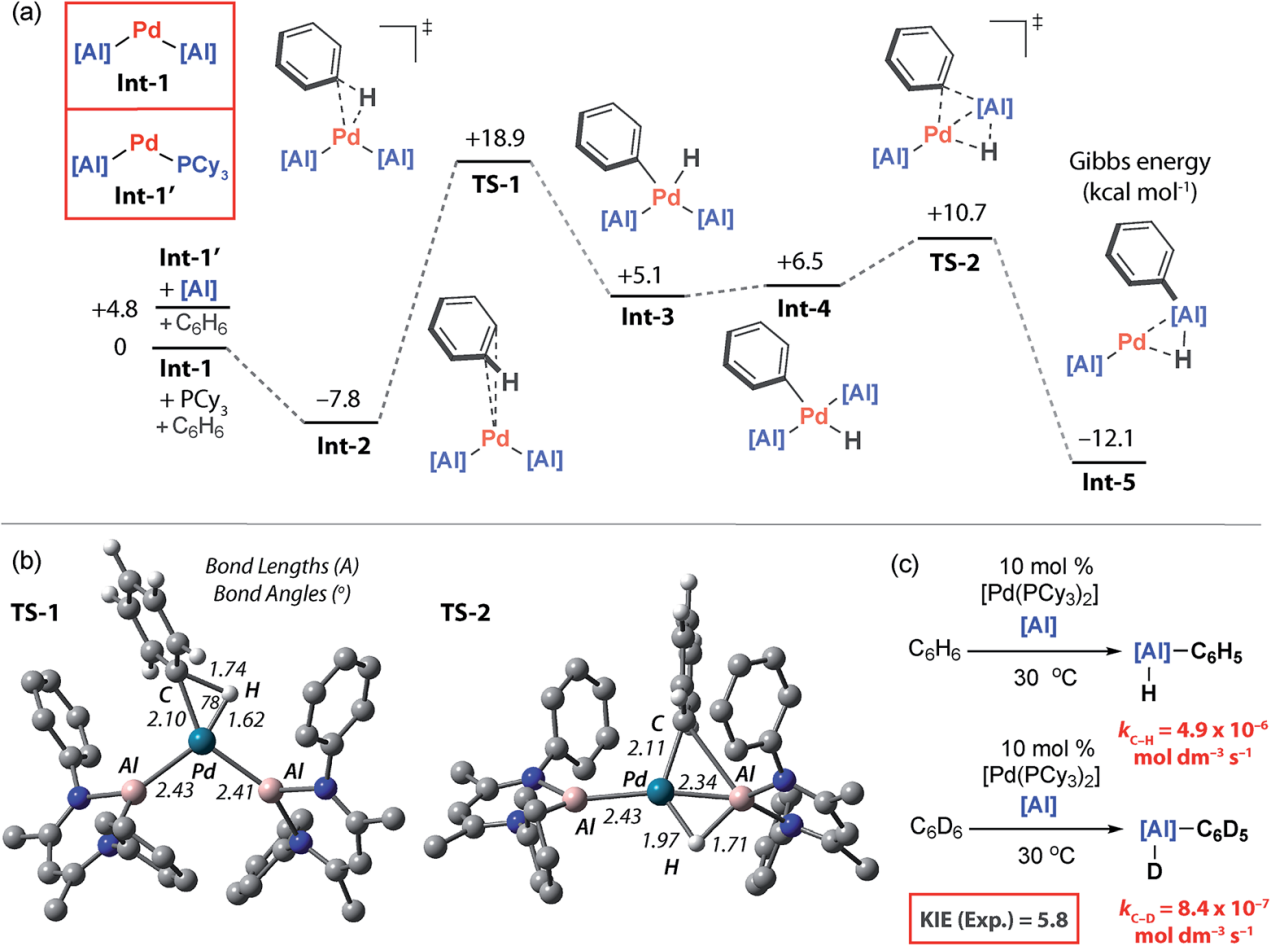
(a) Calculated PES for C–H alumination of benzene, M06L-D3, hybrid basis set. (b) Geometries of oxidative addition transition state (**TS-1**) and double migration transition state (**TS-2**), *i*-propyl groups omitted for clarity. (c) Experimentally determined Kinetic Isotope Effect (KIE).

**Fig. 6:**
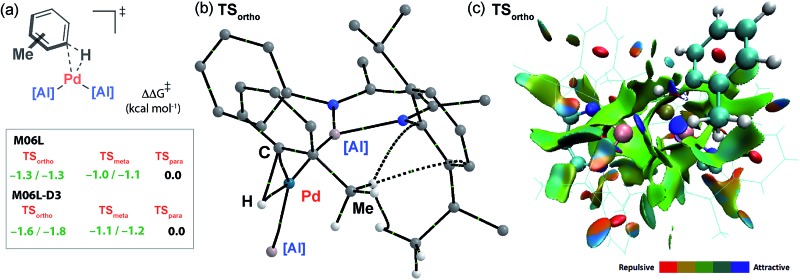
(a) Comparison of the Gibbs energies of the regioisomeric transition states for oxidative addition of C–H bonds of toluene to **Int-1**. (b) QTAIM plot of **TS*_ortho_*** (selected atoms only) showing the presence of weak non-covalent interactions. (c) NCI plot of **TS*_ortho_***.

1,4-, 1,3- and 1,2-dimethylbenzene also undergo C–H alumination at 25 °C and in the cases where regioisomeric products are possible, there is a preference to functionalise the C–H bonds in the *ortho*- and *meta*-positions with respect to the methyl groups (Fig. 3, **5**, **6-*o*,*m***, **7-*o*,*m***). For 1,3-dimethylbenzene there is no evidence for C–H bond activation at the sterically inaccessible position between the two methyl groups and, consistent with this result 1,3,5-trimethylbenzene (mesitylene) does not yield products of C–H alumination even at longer reaction times and elevated temperatures. For comparison C–H borylation of 1,3-disubstituted arenes occurs with high *meta*-selectivities and *ortho*-functionalised products are not observed in significant amounts.^14^

The reaction between **2** and biphenyl had to be carried out in cyclohexane as a solvent as this substrate is a solid under ambient conditions. As a result, this reaction proved to be slower than the C–H functionalisation of arenes carried out under neat conditions and required a slightly elevated temperature and longer reaction time (40 °C, 48 h). The formation of *meta*- and *para*-aluminated complexes (**8-*m*,*p***) was observed consistent with a switch in regiochemistry relative to toluene and xylenes. Attempts to expand the reaction scope to the C–H alumination of alkanes, such as *n*-hexane or methylcyclopentane, did not lead to clean formation of organoaluminium compounds under the reaction conditions reported herein.

The catalytic competency of **PdAl_2_**, along with its observation during catalytic turnover, strongly suggest that this species is an important resting state. Inspection of the solid-state structure of **PdAl_2_** reveals that this 16-electron complex is coordinatively saturated and unlikely to be directly responsible for C–H bond activation. Dissolution of single crystals of **PdAl_2_** in C_6_D_6_ at 25 °C forms a complex equilibrium mixture containing **PdAl_2_**, [Pd(PCy_3_)_2_], PCy_3_ and further unidentified species as measured by ^31^P NMR spectroscopy. The position of the equilibrium could be biased entirely toward **PdAl_2_** by addition of a further equiv. of **2** and lowering the temperature to –40 °C. Either phosphine or aluminylene dissociation from **PdAl_2_** are plausible components of this equilibrium and would lead to the formation of two-coordinate palladium(0) intermediates **Int-1** and **Int-1′** respectively (Fig. 5a and b). These electron-rich 14-electron two-coordinate intermediates are reminiscent of [PdL_2_] species that are widely invoked in oxidative addition reactions.^43^ While both are potential catalytic intermediates, a further experiment demonstrates that the phosphine is not required to generate the active species. Hence, the C–H alumination of toluene with **2** can be catalysed by 10 mol% [Pd(η^5^-C_5_H_5_)(η^3^-C_3_H_4_Ph)] and after 18 h at 25 °C yields an identical mixture of products to that obtained with [Pd(PCy_3_)_2_].

### Mechanism and DFT studies

To gain further insight into the mechanism of C–H bond activation, and the unusual regioselectivity, DFT calculations were conducted.^44^ Calculations were initiated from **Int-1** (Fig. 5a). The C–H bond functionalisation mechanism begins with coordination of benzene to **Int-1** to form **Int-2**. The long Pd···C_6_H_6_ distance in **Int-2** (∼3 Å) as well as minimal distortion of the aromatic ring suggest a very weak electrostatic interaction. This assignment is confirmed by NBO analysis, **Int-2** is a loose encounter complex. Breaking of the C–H bond occurs by a classical three-centred oxidative addition transition state, **TS-1**, to generate the *cis*-palladium complex **Int-3**. This is the highest activation barrier on the potential energy surface, Δ*H*^‡^ = 24.9 kcal mol^–1^ and Δ*G*‡298 K = 26.8 kcal mol^–1^. Subsequent *cis*/*trans* isomerisation leads to **Int-4**.^45^–^47^ Al–C and Al–H bond formation occurs in an exergonic step by the concerted migration of both the phenyl and hydride ligands on palladium to aluminium through a low energy transition state, **TS-2**.

Aldridge and co-workers have recently provided the first experimental data for double hydride migration from a main group centre to a late-transition metal.^48^ In this study, a series of rhodium–gallium heterobimetallic complexes were interrogated by neutron diffraction. Modification of the phosphine ligand on rhodium led to the isolation and characterisation of a series of complexes that model the trajectory of the double hydride migration pathway. Our calculations suggest that the microscopic reverse of this striking pathway may be operating under catalytic conditions. Double migration is accompanied by a formal change in the not only the palladium oxidation state from +2 → 0 but also the aluminium oxidation state from +1 → +3. Analysis of the NPA charges is consistent with this argument and the Al centre involved in the double migration pathway becomes more electropositive as it is oxidised (**Int-4**, +1.35; **Int-5**, +1.78) while the innocent Al centre becomes marginally less electropositive as the palladium centre is reduced (**Int-4**, +1.19; **Int-5**, +1.12). In **Int-5**, the reaction product is still coordinated to Pd as a σ-alane complex, and dissociation is required to liberate the product **3a** and allow catalytic turnover (Fig. 5a and b).

The calculations suggest that C–H bond breaking is the turnover limiting step. A series of kinetics experiments were run to complement the computational data. Under pseudo-first order conditions (excess benzene) the catalytic reaction is zero-order in **2**, rate-constants were measured across a 15–35 °C temperature range. The activation parameters were determined as Δ*H*‡298 K = 23.4 ± 1.3 kcal mol^–1^, Δ*S*‡298 K = –5.7 ± 4.5 cal K^–1^ mol^–1^, Δ*G*‡298 K = 25.1 ± 2.6 kcal mol^–1^. These data match closely with the computational activation parameters listed above. Comparing the rates of the reaction of **2** with excess C_6_D_6_ and C_6_H_6_ in two independent experiments gave a large primary KIE of 5.8 ± 0.1 at 30 °C, consistent with almost complete cleavage of the C–H bond of benzene in the turnover-limiting step (Fig. 5c).

### Origin of regioselectivity

The computational model can be used to rationalise the unusual *ortho*- and *meta*-selectivity found during the C–H alumination of toluene. Due to the weak and reversible binding of the arene in the encounter complex **Int-2**, the Curtin–Hammett principle applies and the regioselectivity is determined by the difference in energy of the transition states for C–H bond activation. The five possible regioisomeric transition states for the oxidative addition of the sp^2^ C–H bonds of toluene to **Int-1** were calculated. While the Gibbs activation energies were within a small energy span they follow the trend **TS*_ortho_*** ≈ **TS*_meta_*** < **TS*_para_*** consistent with the experiment (Fig. 6a). Due to the small energy differences involved, a series of functionals were tested (M06L, B3PW91, ωB97x) to probe the reproducibility of the trend across different computational methods. As part of these calculations dispersion forces were modelled as single-point energy corrections allowing a direct assessment of their effect (see ESI† for details). The data consistently show that the inclusion of the dispersion correction is necessary to reproduce the experimental trend. This correction results in a small but significant stabilisation to both **TS*_ortho_*** and, to a lesser extent, **TS*_meta_*** but has no effect on **TS*_para_***. Hence, the origin the unusual regioselectivity in catalytic C–H alumination of toluene and xylenes can be traced back to non-covalent interactions in the coordination sphere and more specifically weakly stabilising dispersion interactions between the flanking aromatic substituents on the aluminylene ligands and the substituent on the arene.^49^–^51^ The nature of these interactions can be further appreciated by Quantum Theory of Atoms-In-Molecules (QTAIM) calculations which show bond-critical points between the C–H bonds of methyl group of toluene and the ligand periphery in **TS*_ortho_*** (C–H···H–C and C–H···π interactions) but not **TS*_para_*** (Fig. 6b) along with a Non-Covalent Interaction (NCI) plot which shows attractive forces between the ligand periphery and methyl group in **TS*_ortho_*** (Fig. 6c).

## Conclusions

In summary, we report the discovery and mechanistic analysis of a new catalytic method for the C–H activation of unactivated arenes (benzene, toluene, xylenes). These reactions proceed with unusual regiocontrol and a preference for the functionalisation of both the *ortho*- and *meta*-position of toluene and xylenes. Our analysis shows that the methyl group of the substrate is in fact acting as a directing group, forming weak attractive interactions with the catalyst that determine the regioselectivity of C–Al bond formation. This is a rare example of catalyst control in which solely dispersion interactions determine selectivity. These findings could lead to the development of new strategies for catalyst design and selectivity control.

## Conflicts of interest

There are no conflicts to declare.

## Supplementary Material

Supplementary information

Supplementary movie

Supplementary information

Crystal structure data
