# Supplementary material for: Room temperature catalytic carbon–hydrogen bond alumination of unactivated arenes: mechanism and selectivity
Source: Chem Sci. 2018 May 29;9(24):5435–40. doi: 10.1039/c8sc02072h (PMC6011207; doi:10.1039/c8sc02072h)
Supplement: Supplementary file 3 [file SC-009-C8SC02072H-s003.pdf]

Electronic Supplementary Material (ESI) for Chemical Science.
This journal is © The Royal Society of Chemistry 2018

Electronic Supplementary Information for:

Room Temperature Catalytic Carbon–Hydrogen Bond Alumination of Unactivated Arenes: Mechanism and Selectivity

Thomas N. Hooper, Martí Garçon, Andrew J. P. White, Mark R. Crimmin*

Department of Chemistry, Imperial College London, South Kensington, London, SW7 2AZ, UK.

Table of contents

1	General Experimental	S-3
2	Experimental Results	S-4
3	X-ray Crystallographic Data	S-19
4	Kinetics Studies by NMR Spectroscopy	S-25
5	Multinuclear NMR Data	S-28
6	Computational Details	S-43

1. General Experimental, Preparation of Materials and Instruments

1.1 Materials

Aromatic compounds (benzene, toluene, xylenes) were purchased as anhydrous reagents (Sigma Aldrich or Acros) and further dried over 3 Å molecular sieves, degassed by the freeze-pump-thaw method (x 3) and stored under inert atmosphere stored with activated 3 Å molecular sieves. Dipp-*BDI*AlH_2 (**S1**), Mes-*BDI*AlH_2 (**1**), Dipp-*BDI*Al(I) (**2**) and Dipp-*BDI*$AlCl_2$ were synthesised by the literature procedures (Ar-*BDI* = $\{(ArNCMe)_2CH\}^-$, Ar = 2,4,6-$Me_3C_6H_2$ (Mes) or 2,6-$^iPr_2C_6H_3$ (Dipp)).[1] $[Pd(PCy_3)_2]$ was synthesised as an analytically pure pale brown crystalline solid from $PdCl_2$ in a 3 step procedure (via intermediates $[Pd(\eta^3\text{-}C_3H_4Ph)(\mu\text{-}Cl)]_2$ and $[CpPd(\eta^3\text{-}C_3H_4Ph)]$) based on literature reactions.[2] All other reagents were purchased from common suppliers (Sigma-Aldrich, Merck, Alfa Aesar, TCI etc.) and used without further purification.

Solvents for air sensitive procedures (toluene, diethyl ether, *n*-hexane, *n*-heptane, cyclohexane) were dried using a solvent purification system (SPS) and stored over activated 3 Å molecular sieves under an inert atmosphere of N_2 or argon before use. C_6H_6 (Sigma-Aldrich anhydrous grade) and C_6D_6 were degassed by the freeze-pump-thaw method (x 3) and stored under inert atmosphere over activated 3 Å molecular sieves.

1.2 Instruments

^{1}H NMR, ^{31}P NMR and ^{13}C NMR spectra were recorded and analysed using Bruker 400 MHz Spectrometer at 298 K. Spectra were recorded in C_6D_6 solvent unless otherwise stated. The reported values for ^{1}H, ^{31}P NMR and ^{13}C NMR data are as follows: chemical shifts (δ ppm), multiplicity (where s = singlet, d = doublet, t = triplet, m = multiplet), integration (not ^{13}C) and coupling constant, *J* (Hz).

[1] (a) S. Yow, S. J. Gates, A. J. P. White, M. R. Crimmin, *Angew. Chem. Int. Ed.,* 2012, **51**, 12559. (b) C. Cui, H.W. Roesky, H.-G. Schmidt, M. Noltemeyer, H. Hao and F. Cimpoesu, *Angew. Chem. Int. Ed.*, 2000, **39**, 4274. (c) M. Stender, B.E. Eichler, N.J. Hardman, P.P. Power, J. Prust, M. Noltemeyer and H.W. Roesky, *Inorg. Chem.*, 2001, **40**, 2794.

[2] (a) P. R. Auburn, P. B. Mackenzie, B. Bosnich, *J. Am. Chem. Soc.*, 1985, **107**, 2033. (b) S. D. Robinson , B. L. Shaw, *J. Chem. Soc.*, 1963, 4806. (c) T. Yoshida, S. Otsuka, Inorg. Synth., 1990, **28**, 114.

2. Experimental Results

2.1 Synthesis of Heterometallic Products

2.1.1 Synthesis of [H{Pd(PCy$_3$)}(Mes-*BDI*Al)]$_2$ (Pd$_2$Al$_2$)

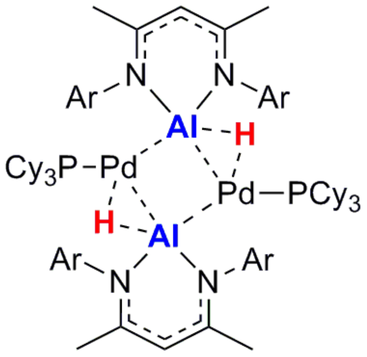

Ar = Mes

[Pd(PCy$_3$)$_2$] (18.4 mg, 0.0276 mmol) and Mes-*BDI*AlH$_2$ (**1**, 10.0 mg, 0.0276 mmol) were dissolved in benzene (1 mL) to form a red solution which was left to stand at 25 °C in the glove box for 72 h. Red crystals of **Pd$_2$Al$_2$** formed and the mother liquor was decanted, the solid washed with benzene (3 x 1 mL) and dried *in vacuo* (6.8 mg, 33%). **Elemental analysis**: calc. for $C_{82}H_{126}Al_2N_4P_2Pd_2$ – C 65.79%, H 8.49%, N 3.74%; found – C 65.90%, H 8.38%, N 3.89%. **FT-IR** (solid, cm^{-1}): 2918, 2846, 1622, 1526, 1476, 1445, 1387, 1273, 1246, 1199, 1147, 1012, 859, 766, 731, 675.

Due to the insolubility of the isolated crystalline material of **Pd$_2$Al$_2$** in common organic solvents (benzene, toluene, fluorobenzene, 1,2-difluorobenzene, hexane, dichloromethane), solution phase analysis (e.g. NMR spectroscopy) of purified **Pd$_2$Al$_2$** was not possible. A ^{31}P{^{1}H} NMR spectrum of the reaction mixture before precipitation of the **Pd$_2$Al$_2$** product in which a dynamic process involving [Pd(PCy$_3$)$_2$] and free PCy$_3$ is shown in section 5.1.1 (figure S8).

2.1.2 Synthesis of [Pd(PCy$_3$)(Dipp-*BDI*Al)$_2$] (PdAl$_2$)

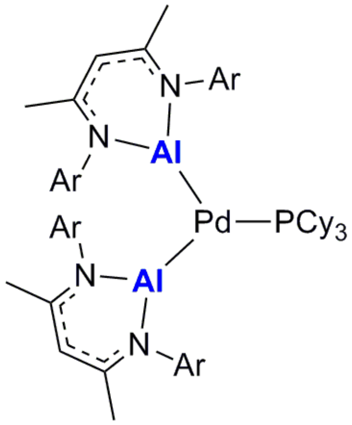

Ar = Dipp

[Pd(PCy$_3$)$_2$] (15.0 mg, 0.0225 mmol) and Dipp-*BDI*Al (**2**, 20.0 mg, 0.0450 mmol) were dissolved in cyclohexane (1 mL) and mixed thoroughly to form a dark green/black solution. The solvent was immediately removed *in vacuo*. The resulting black oily product was redissolved in a 1:1 mixture of *n*-hexane and *n*-heptane (1 mL) and stored at -35 °C. If no crystals formed after a few days the volume of solvent was reduced slightly *in vacuo* and stored at -35 °C again. This process was repeated until black crystals formed. The mother liquor was then decanted by pipette and the black crystals washed with cold (-35 °C) *n*-heptane (2 x 0.5 mL). The black crystals were dried *in vacuo* (the isolated solid appeared to lose crystallinity upon drying, likely due to loss of *n*-hexane solvent of crystallisation), yield 19.2 mg (67%).

The ^{1}H NMR spectrum of **PdAl$_2$** in d$_8$-toluene at 25 °C in section 5.1.2 (figure S9) showed broad peaks indicative of dynamic processes in solution. The ^{31}P{^{1}H} NMR spectrum (figure S10) revealed a number of broad peaks including a peak at 9.8 ppm suggestive of free PCy$_3$. In addition, **PdAl$_2$** will decompose slowly in aromatic solvents to give the aluminated arene products. Cooling of this solution to -60 °C did result in a sharpening of the resonances but several resonances were still observed in the ^{31}P{^{1}H} NMR spectrum. Addition of 1 equivalent of **2** to an NMR sample of **PdAl$_2$** resulted in perturbation of the equilibrium to give a single phosphorus containing Pd complex at -40 °C, (figure S11-S13). The ^{1}H NMR data of this mixture of **PdAl$_2$** and **2** at -40 °C is given below in section 5.1.3, assigned using integrations, comparison to **2** and the COSY spectrum of the mixture. The ^{13}C{^{1}H} (figure S13) and HMQC NMR spectra were also collected.

^{1}H NMR (500 MHz, d$_8$-toluene, 233 K): δ 7.29 (apparent t, $^{3}J_{HH}$ = 7.6 Hz, 2H, Ar-H **PdAl$_2$**), 7.21 (apparent t, $^{3}J_{HH}$ = 8.4 Hz, 2H, Ar-H **PdAl$_2$**) 7.13-7.01 (m overlapping with residual solvent peaks, Ar-H **PdAl$_2$** and **2**), 5.16 (s, **2** C-H), 5.06 (s, 2H, **PdAl$_2$** C-H), 3.73 (sept, $^{3}J_{HH}$ = 6.7 Hz, 2H, **PdAl$_2$** -CMe$_2$H), 3.44 (sept, $^{3}J_{HH}$ = 6.7 Hz, 2H, **PdAl$_2$** -CMe$_2$H), 3.73 (sept, $^{3}J_{HH}$ = 6.7 Hz, 2H, **PdAl$_2$** -CMe$_2$H), 3.19 (overlapping sept, $^{3}J_{HH}$ = 6.7 Hz, 2H, **PdAl$_2$** -CMe$_2$H), 3.17 (overlapping sept, $^{3}J_{HH}$ = 6.7 Hz, **2** -CMe$_2$H), 2.83 (sept, $^{3}J_{HH}$ = 6.7 Hz, 2H, **PdAl$_2$** -CMe$_2$H), 1.93 (br d, J = 12.1 Hz, 1H, **PdAl$_2$** Cy-H), 1.84-1.77 (br m, 8H, **PdAl$_2$** Cy-H), 1.73 (d, $^{3}J_{HH}$ = 6.7 Hz, 6H, **PdAl$_2$** Dipp-CH$_3$), 1.74-1.66 (overlapping br m, **PdAl$_2$** Cy-H), 1.65 (s, **2** CH$_3$), 1.51 (d, $^{3}J_{HH}$ = 6.7 Hz, 6H, **PdAl$_2$** Dipp-CH$_3$), 1.48 (s, 6H, **PdAl$_2$** CH$_3$), 1.46 (overlapping d, $^{3}J_{HH}$ = 6.7 Hz, 6H, **PdAl$_2$** Dipp-CH$_3$), 1.44 (overlapping d, $^{3}J_{HH}$ = 6.7 Hz, **2** Dipp-CH$_3$), 1.42 (s, 6H, **PdAl$_2$** CH$_3$), 1.41 (overlapping d, $^{3}J_{HH}$ = 6.7 Hz, 6H, **PdAl$_2$** Dipp-CH$_3$), 1.27-1.25 (br m, 4H, **PdAl$_2$** Cy-H), 1.23 (d, $^{3}J_{HH}$ = 6.7 Hz, 6H, **PdAl$_2$** Dipp-CH$_3$), 1.17 (overlapping d, $^{3}J_{HH}$ = 6.7 Hz, **2** Dipp-CH$_3$), 1.16 (overlapping d, $^{3}J_{HH}$ = 6.7 Hz, 6H, **PdAl$_2$** Dipp-CH$_3$), 1.14 (d, $^{3}J_{HH}$ = 6.7 Hz, 6H, **PdAl$_2$** Dipp-CH$_3$), 1.01-0.93 (overlapping br m, **PdAl$_2$** Cy-H), 0.42 (d, $^{3}J_{HH}$ = 6.7 Hz, 6H, **PdAl$_2$** Dipp-CH$_3$). Additional cyclohexyl peaks for the PCy$_3$ ligand are obscured beneath the resonances for the Dipp-BDI ligand. Peaks for the *n*-hexane solvent of crystallisation of **PdAl$_2$** were also observed at δ 1.37-1.31 (br m) and 0.99 (t, $^{3}J_{HH}$ = 6.9 Hz). **^{31}P{^{1}H} NMR** (202.4 MHz, d$_8$-toluene, 233 K): δ 29.96 (s, **PdAl$_2$**). **Elemental analysis**: calc. for $C_{76}H_{115}Al_2N_4PPd$ – C 71.51%, H 9.09%, N 4.39%; found – C 71.42%, H 8.93%, N 4.27%.

2.2 Synthesis of Aluminated Arene Products

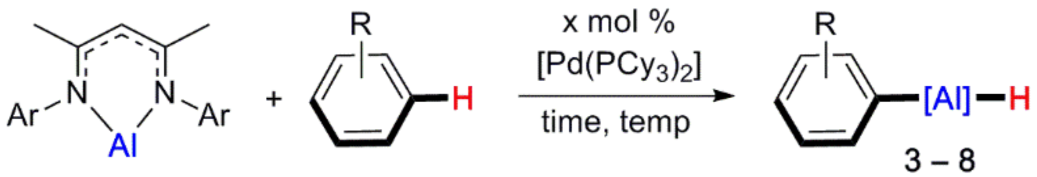

2.2.1 General Procedure for the Catalytic Syntheses of 3-7 from 2 for Liquid Substrates

Dipp-*BDI*Al (**2**, 5.0 mg, 0.0113 mmol) was dissolved in the substrate (0.55 mL, benzene, toluene, *o*-xylene, *m*-xylene, *p*-xylene) to form a red solution and added to an NMR tube containing a capillary insert standard (ferrocene in C_6D_6). The ^{1}H NMR spectrum was measured to obtain a t = 0 value (the ligand backbone proton was integrated vs the ferrocene standard). $Pd(PCy_3)_2$ (11 µL of 0.1 M solution in substrate, 0.0011 mmol, 10 mol%) was then added to the NMR tube to form a red/brown solution after mixing. The reaction mixture was left to stand for 18 h at 25 °C at which point a colourless solution had formed. The ^{1}H NMR spectrum was measured again to obtain a value for the NMR yield. The substrate/solvent was then removed *in vacuo* and the non-volatiles dissolved in C_6D_6 to obtain the NMR spectra of the product(s) in the absence of non-deuterated substrate.

2.2.2 Procedure for the Catalytic Syntheses of 8 from 2 and Biphenyl

Dipp-*BDI*Al (**2**, 5.0 mg, 0.0113 mmol), $Pd(PCy_3)_2$ (~0.7 mg, 0.0011 mmol, ~10 mol%) and biphenyl (3.5 mg, 0.0226 mmol) were weighed into a vial and dissolved in cyclohexane (0.5 mL) and mixed thoroughly to form a red/brown solution. The reaction mixture was transferred to a Young's tap NMR tube and heated to 40 °C for 48 h at which point a colourless solution had formed. The solvent was removed *in vacuo* and the non-volatiles dissolved in C_6D_6 and the products determined by NMR spectroscopy.

2.2.3 Characterisation of Aluminated Species

The aluminated products were synthesised on a larger scale by a non-catalytic route (see section 2.2.4) for full characterisation, including individual regioisomers. In each case the NMR spectra of the products formed catalytically and non-catalytically were compared and found to be identical.

2.2.4 Synthesis of [{(Dipp-NCMe)$_2$CH}Al(Cl)H] (S2)

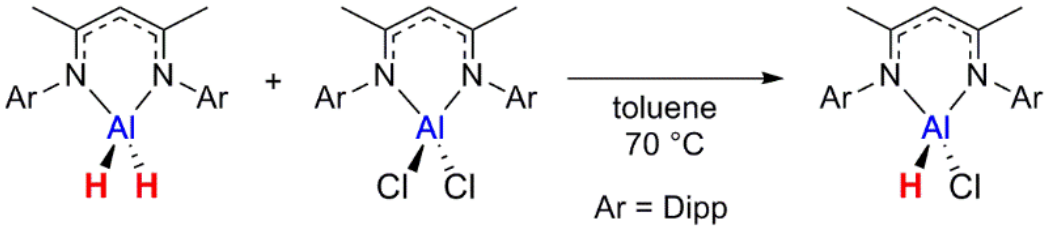

The synthesis of Dipp-*BDI*AlHCl was based on the procedure for the homologous Dipp-*BDI*GaHCl reported by Aldridge and co-workers.[3] Dipp-*BDI*AlH_2 (**S1**, 0.500 g, 1.12 mmol) and Dipp-*BDI*$AlCl_2$ (0.577 g, 1.12 mmol) were dissolved in toluene (30 mL) and transferred to an ampoule. The ampoule was sealed and heated to 70 °C for 48 hours. The toluene was removed *in vacuo* and the solid redissolved in Et_2O (20 mL). The solution was transferred to a Schlenk tube by filter cannula and the volume reduced *in vacuo* to approx. 10 mL. Storage of the solution at -18 °C overnight led to formation of colourless crystals of Dipp-*BDI*AlHCl which were isolated by filter cannula and dried *in vacuo*. Reduction in volume of the mother liquor and storage at -35 °C led to 3 further crops of Dipp-*BDI*AlHCl (**S2**), combined yield (0.635 g, 59%). **^{1}H NMR** (400 MHz, C_6D_6, 298 K): δ 7.16-7.11 (m, 4H, Ar-H), 7.08-7.05 (m, 2H, Ar-H), 4.87 (s, 1H, C-H), 3.47 (sept, $^3J_{HH}$ = 6.8 Hz, 2H, -CMe_2H), 3.30 (sept, $^3J_{HH}$ = 6.7 Hz, 2H, -CMe_2H), 1.51 (s, 6H, CH_3), 1.47 (d, $^3J_{HH}$ = 6.7 Hz, 6H, Dipp-CH_3), 1.34 (d, $^3J_{HH}$ = 6.7 Hz, 6H, Dipp-CH_3), 1.12 (d, $^3J_{HH}$ = 6.8 Hz, 6H, Dipp-CH_3), 1.10 (d, $^3J_{HH}$ = 6.8 Hz, 6H, Dipp-CH_3). The signal for Al-H could not be observed. **^{13}C{^{1}H} NMR** (100.6 MHz, C_6D_6, 298 K) δ 170.75, 144.89, 143.73, 138.30, 127.65, 124.70, 124.29, 97.27, 28.46, 28.19, 25.11, 24.86, 24.52, 24.32, 22.88 ppm. **Elemental analysis**: calc. for $C_{29}H_{42}AlClN_2$ – C 72.38%, H 8.80%, N 5.83%; found – C 72.26%, H 8.63%, N 5.78%.

2.2.5 General Procedure for Non-Catalytic Synthesis of Dipp-*BDI*AlHAr

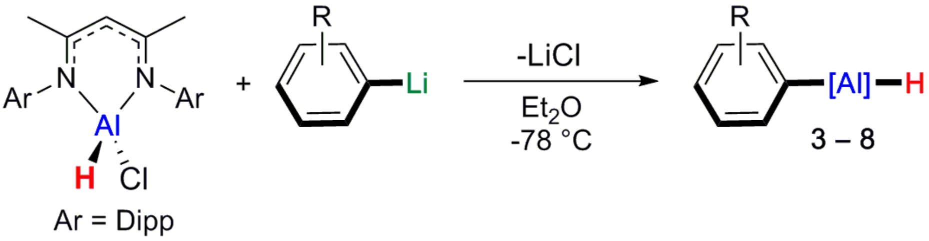

The aryllithium reagents were synthesised from reaction of the parent iodo- or bromo-aryls with ^{n}BuLi in pentane to precipitate the aryl lithium as a solid which was dried and isolated in

[3] J. Turner, J.A.B. Abdalla, J.I. Bates, R. Tirfoin, M.J. Kelly, N. Phillips, S. Aldridge, *Chem. Sci.*, 2013, **4**, 4245.

accordance with the literature procedure for PhLi.[4] Dipp-*BDI*AlHCl (**S2**, 0.100 g, 0.208 mmol, see section 2.2.4) and the aryllithium reagent (0.250 mmol, 1.2 equiv.) were weighed into separate Schlenks and dissolved in Et_2O (5 mL each). The solutions were cooled to -78 °C and the aryllithium solution transferred to the Dipp-*BDI*AlHCl solution by cannula. The solution was stirred for 5 min at -78 °C and allowed to warm to room temperature, during which time a white precipitate formed. After stirring for 1 h at room temperature, the Et_2O was removed *in vacuo* and *n*-hexane (10 mL) added with vigorous stirring. The solution was filtered by cannula and the solvent again removed *in vacuo*. The products were crystallised from *n*-hexane (~0.5-2 mL) at -35 °C in the glove box freezer.

2.2.6 Synthesis of Dipp-*BDI*Al(Ph)H (3a): Catalytic synthesis from the reaction of **2** with benzene via catalytic procedure in section 2.2.1 (0.5 mol%, catalyst, 18 h, 25 °C). Yield: 97% by NMR spectroscopy. Also synthesised non-catalytically from procedure in section 2.2.5 (yield 76 mg, 70%) where sample for characterisation was isolated. See section 5.2.1 for comparison of NMR spectra. **^{1}H NMR** (400 MHz, C_6D_6, 298 K): δ 7.35-7.32 (m, 2H, Ar-H), 7.20-6.99 (overlapping m, 9H, Ar-H), 4.94 (s, 1H, C-H), 4.71 (br {FWHM ~ 160 Hz}, 1H, Al-H), 3.60 (sept, $^3J_{HH}$ = 6.8 Hz, 2H, -$CHMe_2$), 3.15 (sept, $^3J_{HH}$ = 6.8 Hz, 2H, -$CHMe_2$), 1.59 (s, 6H, CH_3), 1.44 (d, $^3J_{HH}$ = 6.8 Hz, 6H, Dipp-CH_3), 1.18 (d, $^3J_{HH}$ = 6.8 Hz, 6H, Dipp-CH_3), 1.09 (d, $^3J_{HH}$ = 6.8 Hz, 6H, Dipp-CH_3), 0.59 (d, $^3J_{HH}$ = 6.8 Hz, 6H, Dipp-CH_3). **^{13}C{^{1}H} NMR** (100.6 MHz, C_6D_6, 298 K): δ 170.23, 144.36, 143.98, 139.98, 137.76, 127.49, 127.20, 126.72, 124.38, 124.36, 97.04, 28.49, 28.25, 25.01, 24.34, 24.27, 23.69, 22.89. The signal for C–Al could not be observed.

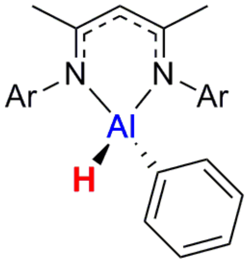

3a, Ar = Dipp

2.2.7 Synthesis of Dipp-*BDI*Al{C_6H_4(2-Me)}H (4-o): Formed as part of a mixture of regioisomers from the reaction of **2** with toluene via catalytic procedure in section 2.2.1 (10 mol%, catalyst, 18 h, 25 °C). Combined yield for **4-o**, **4-m** and **4-p**: 93% by NMR spectroscopy (**4-o**:**4-m**:**4-p** = 42:46:12). Also synthesised non-catalytically from procedure in section 2.2.5 (yield 97 mg, 87%) where sample for characterisation was isolated. See section 5.2.2 for comparison of NMR spectra. **^{1}H NMR** (400 MHz, C_6D_6, 298 K): δ 7.72 (br d, $^3J_{HH}$ = 6.6 Hz, 1H, Ar-H), 7.15-7.07 (overlapping m, 6H, Ar-H), 6.96 (apparent dd, $^3J_{HH}$ = 7.0 Hz, $^4J_{HH}$ = 2.2 Hz, 2H, Ar-H), 6.96-6.89 (overlapping m, 1H, Ar-H), 4.94 (s, 1H, C-H), 4.60 (br {FWHM ~ 170 Hz}, 1H, Al-H), 3.56 (sept, $^3J_{HH}$ = 6.8 Hz, 2H,

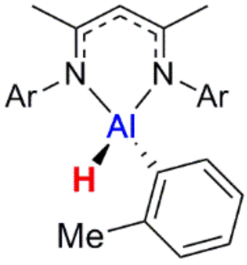

4o, Ar = Dipp

[4] S. Thapa, P. Basnet, S.K. Gurung, R. Giri, *Chem. Commun.*, 2015, **51**, 4009.

-CHMe$_2$), 3.10 (sept, $^3J_{HH}$ = 6.8 Hz, 2H, -CHMe$_2$), 2.60 (br s, 3H, Ar-CH$_3$), 1.54 (s, 6H, CH$_3$), 1.48 (d, $^3J_{HH}$ = 6.8 Hz, 6H, Dipp-CH$_3$), 1.17 (d, $^3J_{HH}$ = 6.8 Hz, 6H, Dipp-CH$_3$), 1.09 (d, $^3J_{HH}$ = 6.8 Hz, 6H, Dipp-CH$_3$), 0.39 (d, $^3J_{HH}$ = 6.8 Hz, 6H, Dipp-CH$_3$). **^{13}C{^{1}H} NMR** (100.6 MHz, C_6D_6, 298 K): δ 169.89, 145.22, 145.04, 143.57, 139.85, 139.54, 128.32, 128.04, 127.22, 124.49, 124.28, 123.94, 97.26, 28.68, 27.69, 25.81 (Ar-CH$_3$), 24.69, 24.48, 24.38, 23.59, 23.02. The signal for C–Al could not be observed. **Elemental analysis**: calc. for $C_{36}H_{49}AlN_2$ – C 80.54%, H 9.21%, N 5.22%; found – C 80.38%, H 9.27%, N 5.38%.

2.2.8 Synthesis of Dipp-*BDI*Al{C_6H_4(3-Me)}H (4-m): Formed as part of a mixture of regioisomers from the reaction of **2** with toluene via catalytic procedure in section 2.2.1 (10 mol%, catalyst, 18 h, 25 °C). Combined yield for **4-o**, **4-m** and **4-p**: 93% by NMR spectroscopy (**4-o**:**4-m**:**4-p** = 42:46:12). Also synthesised non-catalytically from procedure in section 2.2.5 (yield 87 mg, 78%) where sample for characterisation was isolated. See section 5.2.2 for comparison of NMR spectra. **^{1}H NMR** (400 MHz, C_6D_6, 298 K): δ 7.16-7.11 (overlapping m, 5H, Ar-H), 7.05-6.97 (overlapping m, 4H, Ar-H), 6.94-6.90 (m, 1H, Ar-H), 4.94 (s, 1H, C-H), 4.72 (br {FWHM ~ 150 Hz}, 1H, Al-H), 3.61 (sept, $^3J_{HH}$ = 6.8 Hz, 2H, -CHMe$_2$), 3.18 (sept, $^3J_{HH}$ = 6.8 Hz, 2H, -CHMe$_2$), 2.02 (s, 3H, Ar-CH$_3$), 1.60 (s, 6H, CH$_3$), 1.44 (d, $^3J_{HH}$ = 6.8 Hz, 6H, Dipp-CH$_3$), 1.19 (d, $^3J_{HH}$ = 6.8 Hz, 6H, Dipp-CH$_3$), 1.10 (d, $^3J_{HH}$ = 6.8 Hz, 6H, Dipp-CH$_3$), 0.64 (d, $^3J_{HH}$ = 6.8 Hz, 6H, Dipp-CH$_3$). **^{13}C{^{1}H} NMR** (100.6 MHz, C_6D_6, 298 K): δ 170.15, 144.32, 144.11, 140.10, 138.74, 134.99, 134.77, 128.06, 127.14, 126.64, 124.39, 124.28, 96.98, 28.45, 28.29, 25.12, 24.33, 24.32, 23.65, 22.88, 21.18 (Ar-CH$_3$). The signal for C–Al could not be observed.

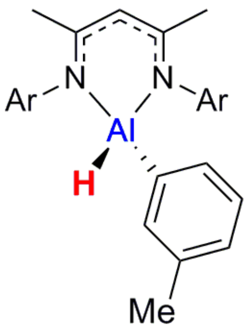

4m, Ar = Dipp

2.2.9 Synthesis of Dipp-*BDI*Al{C_6H_4(4-Me)}H (4-p): Formed as part of a mixture of regioisomers from the reaction of **2** with toluene via catalytic procedure in section 2.2.1 (10 mol%, catalyst, 18 h, 25 °C). Combined yield for **4-o**, **4-m** and **4-p**: 93% by NMR spectroscopy (**4-o**:**4-m**:**4-p** = 42:46:12). Also synthesised non-catalytically from procedure in section 2.2.5 (yield 79 mg, 71%) where sample for characterisation was isolated. See section 5.2.2 for comparison of NMR spectra. **^{1}H NMR** (400 MHz, C_6D_6, 298 K): δ 7.24 (d, $^3J_{HH}$ = 7.6 Hz, 2H, Ar-H), 7.17-7.10 (overlapping m, 4H, Ar-H), 7.03 (apparent dd, $^3J_{HH}$ = 6.2 Hz, $^4J_{HH}$ = 3.1 Hz, 2H, Ar-H), 6.86 (d, $^3J_{HH}$ = 7.6 Hz, 2H, Ar-H), 4.95

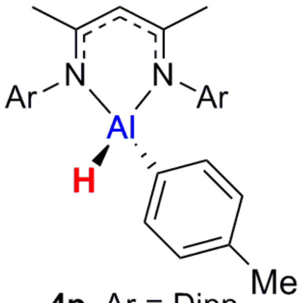

4p, Ar = Dipp

(s, 1H, C-H), 4.72 (br {FWHM ~ 150 Hz}, 1H, Al-H), 3.61 (sept, $^3J_{HH}$ = 6.8 Hz, 2H, -CHMe$_2$), 3.19 (sept, $^3J_{HH}$ = 6.8 Hz, 2H, -CHMe$_2$), 2.05 (s, 3H, Ar-CH$_3$), 1.60 (s, 6H, CH$_3$), 1.44 (d, $^3J_{HH}$ = 6.8 Hz, 6H, Dipp-CH$_3$), 1.18 (d, $^3J_{HH}$ = 6.8 Hz, 6H, Dipp-CH$_3$), 1.10 (d, $^3J_{HH}$ = 6.8 Hz, 6H, Dipp-CH$_3$), 0.65 (d, $^3J_{HH}$ = 6.8 Hz, 6H, Dipp-CH$_3$). **^{13}C{^{1}H} NMR** (100.6 MHz, C$_6$D$_6$, 298 K): δ 170.14, 144.32, 144.06, 140.11, 137.87, 136.44, 127.54, 127.15, 124.36, 124.31, 97.02, 28.46, 28.25, 25.08, 24.32, 24.28, 23.76, 22.89, 21.21 (Ar-CH$_3$). The signal for C–Al could not be observed.

2.2.10 Synthesis of Dipp-*BDI*Al{C$_6$H$_3$(2,5-Me$_2$)}H (5): Catalytic synthesis from the reaction of **2** with *p*-xylene via catalytic procedure in section 2.2.1 (10 mol%, catalyst, 18 h, 25 °C). Yield: 88% by NMR spectroscopy. Isolated as a pure solid from this reaction mixture by crystallisation from *n*-hexane (~1 mL) at -35 °C. **^{1}H NMR** (400 MHz, C$_6$D$_6$, 298 K): δ 7.49 (br s, 1H, Ar-H), 7.16-7.06 (overlapping m, 5H, Ar-H), 7.00-6.94 (overlapping m, 3H, Ar-H), 4.95 (s, 1H, C-H), 4.62 (br {FWHM ~ 120 Hz}, 1H, Al-H), 3.58 (sept, $^3J_{HH}$ = 6.8 Hz, 2H, -CHMe$_2$), 3.13 (sept, $^3J_{HH}$ = 6.8 Hz, 2H, -CHMe$_2$), 2.63 (br s, 3H, Ar-CH$_3$), 1.96 (s, 3H, Ar-CH$_3$), 1.54 (s, 6H, CH$_3$), 1.50 (d, $^3J_{HH}$ = 6.8 Hz, 6H, Dipp-CH$_3$), 1.18 (d, $^3J_{HH}$ = 6.8 Hz, 6H, Dipp-CH$_3$), 1.11 (d, $^3J_{HH}$ = 6.8 Hz, 6H, Dipp-CH$_3$), 0.42 (d, $^3J_{HH}$ = 6.8 Hz, 6H, Dipp-CH$_3$). **^{13}C{^{1}H} NMR** (100.6 MHz, C$_6$D$_6$, 298 K): δ 169.77, 145.05, 143.61, 140.58, 139.92, 132.35, 128.73, 128.33, 127.98, 127.22, 124.50, 124.30, 97.21, 28.68, 27.62, 25.47 (Ar-CH$_3$), 24.74, 24.49, 24.45, 23.61, 22.70, 20.56 (Ar-CH$_3$). The signal for C–Al could not be observed. Peaks for *n*-hexane solvent of crystallisation were also observed.

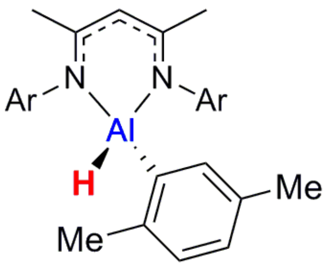

5, Ar = Dipp

2.2.11 Synthesis of Dipp-*BDI*Al{C$_6$H$_3$(2,4-Me$_2$)}H (6-o): Formed as part of a mixture of regioisomers from the reaction of **2** with *m*-xylene via catalytic procedure in section 2.2.1 (10 mol%, catalyst, 18 h, 25 °C). Combined yield for **6o** and **6m**: 87% by NMR spectroscopy (**6-o**:**6-m** = 49:51). Also synthesised non-catalytically from procedure in section 2.2.5 (yield 52 mg, 45%) where sample for characterisation was isolated. See section 5.2.3 for comparison of NMR spectra. **^{1}H NMR** (400 MHz, C$_6$D$_6$, 298 K): δ 7.65 (br d, $^3J_{HH}$ = 7.1 Hz, 1H, Ar-H), 7.13-7.07 (overlapping m, 4H, Ar-H), 6.99-6.95 (overlapping m, 3H, Ar-H), 6.77 (br d, $^3J_{HH}$ = 7.1 Hz, 1H, Ar-H), 4.96 (s, 1H, C-H), 4.72 (br {FWHM ~ 160 Hz}, 1H, Al-H), 3.57 (sept, $^3J_{HH}$ = 6.8 Hz, 2H, -CHMe$_2$), 3.13 (sept, $^3J_{HH}$ = 6.8 Hz, 2H, -CHMe$_2$), 2.59 (br s, 3H, Ar-CH$_3$), 2.15 (s, 3H, Ar-CH$_3$), 1.55 (s, 6H, CH$_3$), 1.48 (d, $^3J_{HH}$ = 6.8 Hz, 6H, Dipp-CH$_3$), 1.18 (d, $^3J_{HH}$ = 6.8

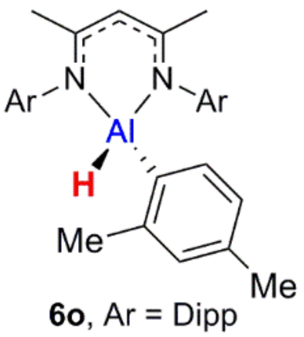

6o, Ar = Dipp

Hz, 6H, Dipp-CH_3), 1.11 (d, $^3J_{HH}$ = 6.8 Hz, 6H, Dipp-CH_3), 0.42 (d, $^3J_{HH}$ = 6.8 Hz, 6H, Dipp-CH_3). **^{13}C{^{1}H} NMR** (100.6 MHz, C_6D_6, 298 K): δ 169.80, 145.07, 143.59, 139.96, 139.68, 137.01, 129.35, 127.18, 124.72, 124.46, 124.26, 97.21, 28.67, 27.67, 25.69 (Ar-CH_3), 24.69, 24.47, 24.37, 23.65, 23.02, 21.17 (Ar-CH_3). Peaks for *n*-hexane solvent of crystallisation were also observed. The signal for C–Al and one other C(ipso) could not be observed. **Elemental analysis**: calc. for $C_{37}H_{51}AlN_2$ – C 80.67%, H 9.34%, N 5.09%; found – C 80.48%, H 9.39%, N 5.02%.

2.2.12 Synthesis of Dipp-*BDI*Al{C_6H_3(3,5-Me_2)}H (6-m): Formed as part of a mixture of regioisomers from the reaction of **2** with *m*-xylene via catalytic procedure in section 2.2.1 (10 mol%, catalyst, 18 h, 25 °C). Combined yield for **6-o** and **6-m**: 87% by NMR spectroscopy (**6-o**:**6-m** = 49:51). Also synthesised non-catalytically from procedure in section 2.2.5 (yield 62 mg, 54%) where sample for characterisation was isolated. See section 5.2.3 for comparison of NMR spectra. **^{1}H NMR** (400 MHz, C_6D_6, 298 K): δ 7.17-7.15 (overlapping m with residual solvent peak, 4H, Ar-H), 7.04 (apparent dd, $^3J_{HH}$ = 5.6 Hz, $^4J_{HH}$ = 3.7 Hz, 2H, Ar-H), 6.82 (s, 2H, Ar-H), 6.75 (s, 1H, Ar-H), 4.95 (s, 1H, C-H), 4.73 (br {FWHM ~ 150 Hz}, 1H, Al-H), 3.62 (sept, $^3J_{HH}$ = 6.8 Hz, 2H, -$CHMe_2$), 3.21 (sept, $^3J_{HH}$ = 6.8 Hz, 2H, -$CHMe_2$), 2.06 (s, 6H, Ar-CH_3), 1.62 (s, 6H, CH_3), 1.46 (d, $^3J_{HH}$ = 6.8 Hz, 6H, Dipp-CH_3), 1.20 (d, $^3J_{HH}$ = 6.8 Hz, 6H, Dipp-CH_3), 1.12 (d, $^3J_{HH}$ = 6.8 Hz, 6H, Dipp-CH_3), 0.67 (d, $^3J_{HH}$ = 6.8 Hz, 6H, Dipp-CH_3). **^{13}C{^{1}H} NMR** (100.6 MHz, C_6D_6, 298 K): δ 170.07, 144.28, 144.21, 140.18, 135.77, 134.84, 128.94, 127.07, 124.40, 124.20, 96.91, 28.41, 28.35, 25.21, 24.41, 24.32, 23.61, 22.88, 21.10 (Ar-CH_3). The signal for C–Al could not be observed. **Elemental analysis**: calc. for $C_{37}H_{51}AlN_2$ – C 80.67%, H 9.34%, N 5.09%; found – C 80.62%, H 9.42%, N 5.05%.

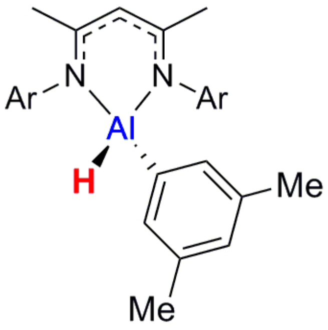

6m, Ar = Dipp

2.2.13 Synthesis of Dipp-*BDI*Al{C_6H_3(2,3-Me_2)}H (7-o): Formed as part of a mixture of regioisomers from the reaction of **2** with *o*-xylene via catalytic procedure in section 2.2.1 (10 mol%, catalyst, 18 h, 25 °C). Combined yield for **7-o** and **7-m**: 88% by NMR spectroscopy (**7-o**:**7-m** = 43:57). Also synthesised non-catalytically from procedure in section 2.2.5 (yield 46 mg, 40%) where sample for characterisation was isolated. See section 5.2.4 for comparison of NMR spectra. **^{1}H NMR** (400 MHz, C_6D_6, 298 K): δ 7.57 (br d, $^3J_{HH}$ = 7.1 Hz, 1H, Ar-H), 7.13-7.06 (m, 4H, Ar-H), 7.01 (br d, $^3J_{HH}$ = 7.1 Hz, 1H, Ar-H), 6.94 (apparent dd, J_{HH} = 7.3 Hz, J_{HH} = 2.0 Hz, 2H, Ar-H), 6.89 (br t, $^3J_{HH}$ = 7.1 Hz, 1H, Ar-H), 4.96 (s, 1H, C-H), 4.60 (br {FWHM ~ 160 Hz}, 1H, Al-H), 3.58 (sept, $^3J_{HH}$ = 6.8 Hz, 2H, -$CHMe_2$), 3.09 (sept, $^3J_{HH}$ = 6.8 Hz, 2H, -$CHMe_2$), 2.55 (s, 3H, Ar-CH_3), 2.23 (s, 3H, Ar-CH_3), 1.56 (s, 6H, CH_3), 1.50 (d, $^3J_{HH}$ = 6.8 Hz, 6H, Dipp-CH_3), 1.18 (d, $^3J_{HH}$ = 6.8 Hz, 6H, Dipp-CH_3), 1.09 (d, $^3J_{HH}$ = 6.8 Hz, 6H, Dipp-CH_3), 0.33 (d, $^3J_{HH}$ = 6.8 Hz, 6H, Dipp-CH_3). **^{13}C{^{1}H} NMR** (100.6 MHz, C_6D_6, 298 K): δ 169.78, 144.98, 143.61, 143.37, 139.85, 137.71, 134.49, 129.73, 127.20, 124.75, 124.45, 124.29, 97.05, 28.68, 27.62, 24.68, 24.51, 24.50, 24.14 (Ar-CH_3), 23.60, 23.01, 20.38 (Ar-CH_3). The signal for C–Al could not be observed. **Elemental analysis**: calc. for $C_{37}H_{51}AlN_2$ – C 80.67%, H 9.34%, N 5.09%; found – C 80.51%, H 9.49%, N 5.16%.

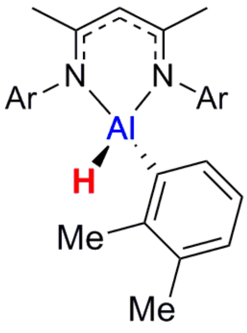

7o, Ar = Dipp

2.2.14 Synthesis of Dipp-*BDI*Al{C_6H_3(3,4-Me_2)}H (7-m): Formed as part of a mixture of regioisomers from the reaction of **2** with *o*-xylene via catalytic procedure in section 2.2.1 (10 mol%, catalyst, 18 h, 25 °C). Combined yield for **7-o** and **7-m**: 88% by NMR spectroscopy (**7-o**:**7-m** = 43:57). Also synthesised non-catalytically from procedure in section 2.2.5 (yield 52 mg, 45%) where sample for characterisation was isolated. See section 5.2.4 for comparison of NMR spectra. **^{1}H NMR** (400 MHz, C_6D_6, 298 K): δ 7.16-7.15 (overlapping m with residual solvent peak, 4H, Ar-H), 7.05-7.02 (overlapping m, 3H, Ar-H), 6.93 (s, 1H, Ar-H), 6.85 (d, $^3J_{HH}$ = 7.1 Hz, 1H, Ar-H), 4.96 (s, 1H, C-H), 4.74 (br {FWHM ~ 130 Hz}, 1H, Al-H), 3.62 (sept, $^3J_{HH}$ = 6.8 Hz, 2H, -$CHMe_2$), 3.22 (sept, $^3J_{HH}$ = 6.8 Hz, 2H, -$CHMe_2$), 1.99 (s, 3H, Ar-CH_3), 1.95 (s, 3H, Ar-CH_3), 1.61 (s, 6H, CH_3), 1.44 (d, $^3J_{HH}$ = 6.8 Hz, 6H, Dipp-CH_3), 1.19 (d, $^3J_{HH}$ = 6.8 Hz, 6H, Dipp-CH_3), 1.11 (d, $^3J_{HH}$ = 6.8 Hz, 6H, Dipp-CH_3), 0.70 (d, $^3J_{HH}$ = 6.8 Hz, 6H, Dipp-CH_3). **^{13}C{^{1}H} NMR** (100.6 MHz, C_6D_6, 298 K): δ 170.07, 144.24, 144.22, 140.24, 139.42, 135.55, 134.92, 133.71, 128.12, 127.09, 124.39, 124.22, 96.98, 28.40, 28.31, 25.23, 24.34, 24.29, 23.70, 22.88, 19.44 (Ar-CH_3), 19.14 (Ar-

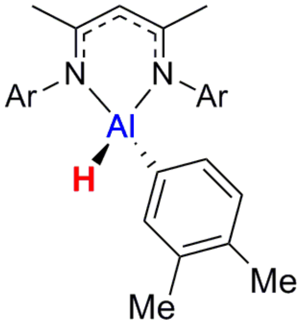

7m, Ar = Dipp

CH_3). The signal for C–Al could not be observed. **Elemental analysis**: calc. for $C_{37}H_{51}AlN_2$ – C 80.67%, H 9.34%, N 5.09%; found – C 80.45%, H 9.12%, N 4.97%.

2.2.15 Synthesis of Dipp-*BDI*Al{C_6H_4(3-Ph)}H (8-m): Formed as part of a mixture of regioisomers from the reaction of **2** with biphenyl via catalytic procedure in section 2.2.2 (10 mol%, catalyst, 48 h, 40 °C). Combined yield for **8-m** and **8-p**: 85% by NMR spectroscopy (**8-m**:**8-p** = 65:35). Also synthesised non-catalytically from procedure in section 2.2.5 (yield 62 mg, 50%) where sample for characterisation was isolated. See section 5.2.5 for comparison of NMR spectra. **^{1}H NMR** (400 MHz, C_6D_6, 298 K): δ 7.60 (br d, $^3J_{HH}$ = 2.1 Hz, 1H, Ar-H), 7.37-7.33 (m, 3H, Ar-H), 7.24-7.18 (m, 3H, Ar-H), 7.16-7.05 (overlapping m, 6H, Ar-H), 7.03-7.00 (m, 2H, Ar-H), 4.94 (s, 1H, C-H), 4.78 (br {FWHM ~ 140 Hz}, 1H, Al-H), 3.61 (sept, $^3J_{HH}$ = 6.8 Hz, 2H, -$CHMe_2$), 3.19 (sept, $^3J_{HH}$ = 6.8 Hz, 2H, -$CHMe_2$), 1.59 (s, 6H, CH_3), 1.43 (d, $^3J_{HH}$ = 6.8 Hz, 6H, Dipp-CH_3), 1.18 (d, $^3J_{HH}$ = 6.8 Hz, 6H, Dipp-CH_3), 1.07 (d, $^3J_{HH}$ = 6.8 Hz, 6H, Dipp-CH_3), 0.62 (d, $^3J_{HH}$ = 6.8 Hz, 6H, Dipp-CH_3). **^{13}C{^{1}H} NMR** (100.6 MHz, C_6D_6, 298 K): δ 170.32, 144.28, 144.15, 142.49, 140.04, 138.97, 136.74, 136.56, 128.34, 127.24, 127.09, 127.05, 126.30, 126.26, 124.45, 124.43, 97.10, 28.48, 28.31, 25.12, 24.30, 24.28, 23.66, 22.87. The signal for C–Al could not be observed. **Elemental analysis**: calc. for $C_{41}H_{51}AlN_2$ – C 82.22%, H 8.59%, N 4.68%; found – C 82.18%, H 8.70%, N 4.56%.

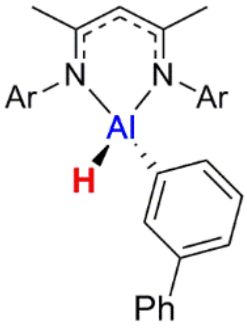

8m, Ar = Dipp

2.2.16 Synthesis of Dipp-*BDI*Al{C_6H_4(4-Ph)}H (8-p): Formed as part of a mixture of regioisomers from the reaction of **2** with biphenyl via catalytic procedure in section 2.2.2 (10 mol%, catalyst, 48 h, 40 °C). Combined yield for **8-m** and **8-p**: 85% by NMR spectroscopy (**8-m**:**8-p** = 65:35). Also synthesised non-catalytically from procedure in section 2.2.5 (yield 51 mg, 41%) where sample for characterisation was isolated. See section 5.2.5 for comparison of NMR spectra. **^{1}H NMR** (400 MHz, C_6D_6, 298 K): δ 7.43-7.37 (m, 5H, Ar-H), 7.32-7.29 (m, 2H, Ar-H), 7.20-7.12 (overlapping m, 5H, Ar-H), 7.08-7.02 (overlapping m, 3H, Ar-H), 4.96 (s, 1H, C-H), 4.75 (br {FWHM ~ 120 Hz}, 1H, Al-H), 3.62 (sept, $^3J_{HH}$ = 6.8 Hz, 2H, -$CHMe_2$), 3.19 (sept, $^3J_{HH}$ = 6.8 Hz, 2H, -$CHMe_2$), 1.61 (s, 6H, CH_3), 1.46 (d, $^3J_{HH}$ = 6.8 Hz, 6H, Dipp-CH_3), 1.20 (d, $^3J_{HH}$ = 6.8 Hz, 6H, Dipp-CH_3), 1.10 (d, $^3J_{HH}$ = 6.8 Hz, 6H, Dipp-CH_3), 0.64 (d, $^3J_{HH}$ = 6.8 Hz, 6H, Dipp-CH_3). **^{13}C{^{1}H} NMR** (100.6 MHz, C_6D_6, 298 K): δ 170.27,

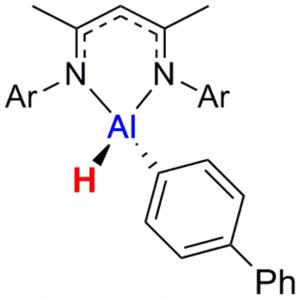

8p, Ar = Dipp

144.37, 144.05, 140.04, 139.98, 138.27, 128.47, 127.25, 126.91, 126.53, 125.30, 124.42, 124.40, 97.06, 29.09, 28.51, 28.26, 25.06, 24.34, 24.28, 23.74, 22.89. The signal for C–Al and one other C(ipso) could not be observed. **Elemental analysis**: calc. for $C_{41}H_{51}AlN_2$ – C 82.22%, H 8.59%, N 4.68%; found – C 82.01%, H 8.69%, N 4.67%.

2.3 Catalytic Synthesis of Aluminated Arene Product from 1

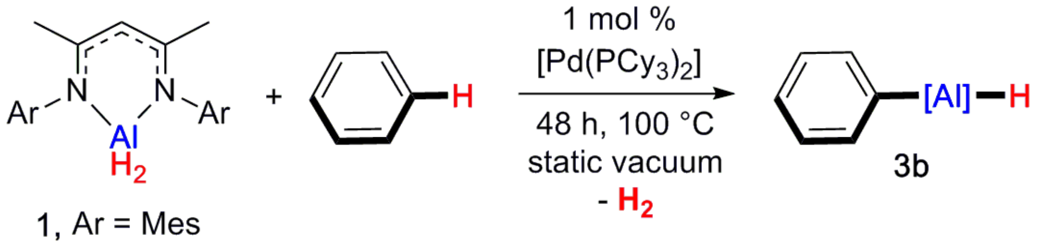

2.3.1 Synthesis of Mes-*BDI*Al(Ph)H (3b)

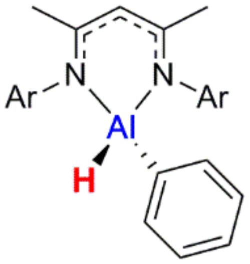

3b, Ar = Mes

1 (160 mg, 0.44 mmol) and [Pd(PCy$_3$)$_2$] (2.9 mg, 0.0044 mmol, 1 mol%) were dissolved in benzene (~5 mL) in the glove box. The solution transferred to a large Young's tap ampoule (~250 mL) and sealed. This was removed from the glove box degassed by the freeze/pump/thaw method (x 3) and followed by heating under vacuum to 100 °C in an oil bath for 48 h. The reaction mixture was cooled to 25 °C and the ampoule was attached to a vacuum line and the solvent removed *in vacuo*. The ampoule was transferred back to the glove box and the residue dissolved in hexane (~5 mL) and filtered through a 0.45 µm syringe filter. The volume of the solution was reduced to ~2 mL *in vacuo* and the solution stored at -35 °C to form a white precipitate. The mother liquor was decanted by pipette and the solid dried *in vacuo.* A further crop of crude product was obtained by concentration of the mother liquor to ~0.5 mL and storage at -35 °C. The combined crude products were recrystallized by dissolving in the benzene (~1 mL) followed by addition of *n*-heptane (~2 mL). The solution was then concentrated *in vacuo* to ~1 mL and storage at -35 °C yielded **3b** as a white solid (yield 64 mg, 33%).

^{1}H NMR (400 MHz, C_6D_6, 298 K): δ 7.81-7.77 (m, 2H, Ar-H), 7.21-7.17 (m, 3H, Ar-H), 6.75 (s, 2H, Mes-Ar-H), 6.56 (s, 2H, Mes-Ar-H), 4.94 (s, 1H, C-H), 4.76 (br {FWHM ~ 150 Hz}, 1H, Al-H), 2.40 (s, 6H, Mes-CH$_3$), 2.03 (s, 6H, Mes-CH$_3$), 1.92 (s, 6H, Mes-CH$_3$), 1.53 (s, 6H, CH$_3$) ppm. **^{13}C{^{1}H} NMR** (100.6 MHz, C_6D_6, 298 K): δ 169.61, 140.01, 138.04, 135.27, 133.44, 132.79, 129.59, 129.50, 127.70, 126.74, 96.65, 22.16, 20.46, 18.81, 18.59. The signal for C–

Al could not be observed. **Elemental analysis**: calc. for $C_{29}H_{35}AlN_2$ – C 79.40%, H 8.05%, N 6.39%; found – C 79.31%, H 8.17%, N 6.47%.

2.4 Reactions of Pd_2Al_2

2.4.1 Synthesis of Mes-*BDI*Al(Ph)H (3b) using Pd_2Al_2 as a Catalyst

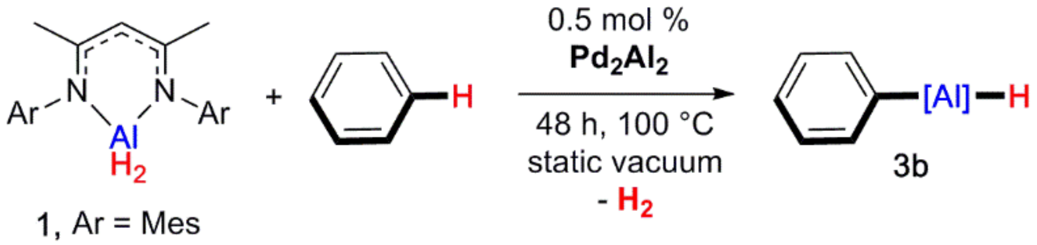

The catalytic procedure detailed in section 2.4.1 was repeated on a smaller scale, **1** (40 mg, 0.11 mmol) in C_6H_6 (1 mL) but using $\mathbf{Pd_2Al_2}$ (0.8 mg, 0.00055 mmol, 0.5 mol%) rather than $[Pd(PCy_3)_2]$ as the catalyst. Although $\mathbf{Pd_2Al_2}$ is insoluble in C_6H_6 at room temperature, heating under vacuum led to a homogeneous yellow solution. Removal of the solvent and analysis of the crude by ^{1}H NMR spectroscopy showed formation of **3b** as the major product.

2.4.2 Reaction of Pd_2Al_2 with Benzene in the Presence of PCy_3

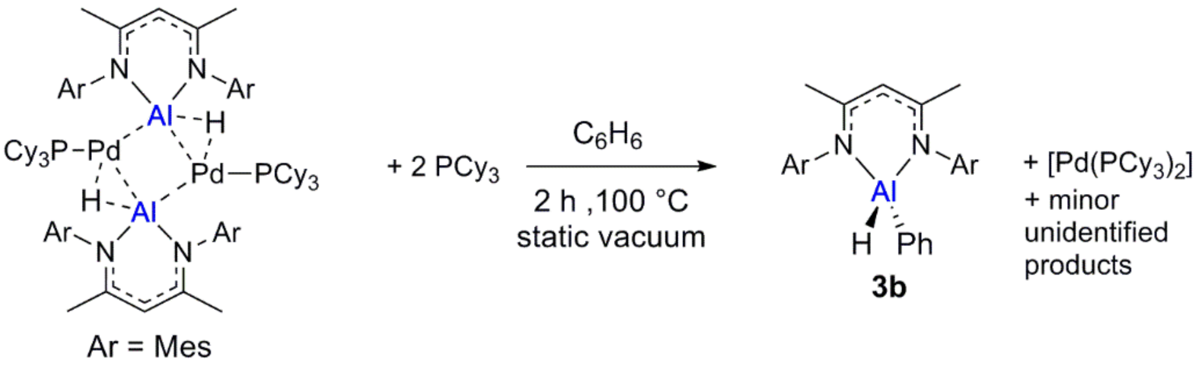

[H{Pd(PCy$_3$)}(Al*BDI*-Mes)]$_2$ ($\mathbf{Pd_2Al_2}$, 10.0 mg, 0.0067 mmol) and PCy_3 (3.7 mg, 0.013 mmol) were weighed into a small vial and C_6H_6 (1 mL) was added. The solution was mixed but due to its insolubility, the red crystals of $\mathbf{Pd_2Al_2}$ did not dissolve. The suspension was transferred to an ampoule, carefully ensuring all solid was transferred. The suspension was then degassed by the freeze/pump/thaw method (x 3) and heated to 100 °C under vacuum for 2 h. Over this time the solid slowly dissolved and a yellow solution formed. After cooling to room temperature the solvent was removed *in vacuo* and the crude sample redissolved in C_6D_6.

The NMR spectrum showed formation of $[Pd(PCy_3)_2]$ and **3b** as the major Al containing product.

2.5 C–H Alumination Using Phosphine Free Catalyst

2.5.1 C–H Alumination of Toluene from 2 Using $[CpPd(\eta^3\text{-}C_3H_4Ph)]$) as the Catalyst

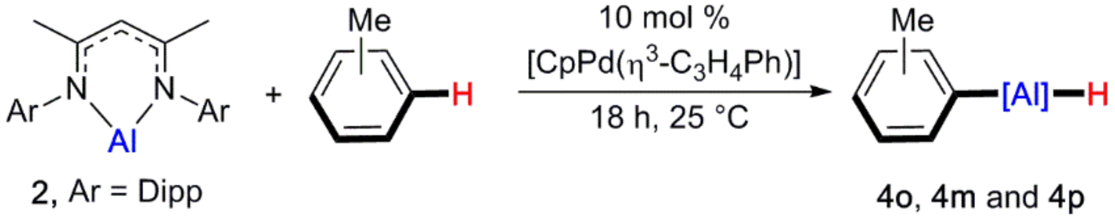

A reaction was carried out between **2** and toluene in the same manner as detailed in section 2.2.1 but using $[CpPd(\eta^3\text{-}C_3H_4Ph)]$ (0.0011 mmol, 10 mol%, see section 1.1) as the catalyst. After 18 h at 25 °C a brown solution was obtained and the ^{1}H NMR spectrum (section 5.3.1) showed the same products **4-o**, **4-m** and **4-p** produced in the same ratio as when $[Pd(PCy_3)_2]$ (10 mol%) was used as the catalyst.

2.6 Palladium Catalysed Reactivity of Dipp-BDIAl (2) with H_2

2.6.1 Reaction of H_2 with $[Pd(PCy_3)_2]$ and 2 equiv. Dipp-BDIAl (2)

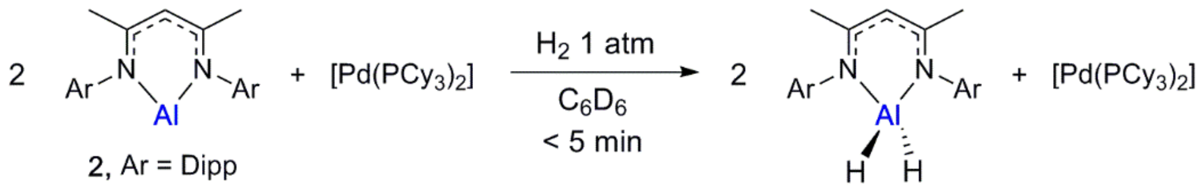

$[Pd(PCy_3)_2]$ (3.8 mg, 0.0056 mmol) and **2** (5.0 mg, 0.0113) were weighed into a small vial and C_6D_6 (0.55 mL) was added. The solution was mixed to dissolve the solid to form a dark green/black solution and transferred to a Young's tap NMR tube. The tube was sealed and the NMR spectrum immediately recorded before H_2 addition. The tube was then degassed by the freeze/pump/thaw method (x 2) and H_2 (1 atm) was added immediately. Upon inversion the solution began to decolourise and after 30 seconds of mixing the solution had become pale yellow. The ^{1}H and $^{31}P\{^1H\}$ NMR spectra (section 5.4.1) showed the products to be $[Pd(PCy_3)_2]$ and Dipp-BDIAlH_2 (**S1**).

2.6.2 Reaction of H_2 with $[Pd(PCy_3)_2]$ and 20 equiv. Dipp-BDIAl (2)

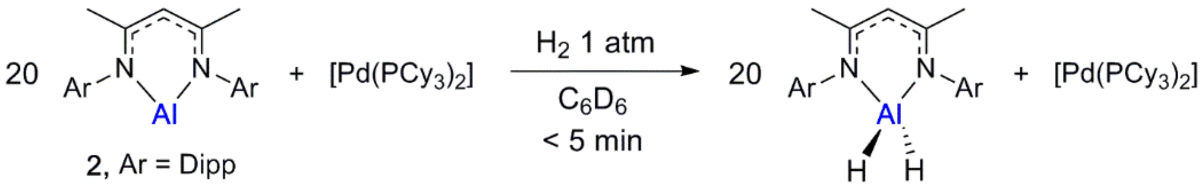

2, Ar = Dipp

2 (5.0 mg, 0.0113) was weighed into a small vial and C_6D_6 (0.49 mL) was added. The solution was mixed to dissolve the solid to form a red solution. $[Pd(PCy_3)_2]$ (56 μL, 0.01 M solution in C_6D_6, 0.00056 mmol) was added, the solution mixed and transferred to a Young's tap NMR tube. The tube was sealed and the NMR spectrum immediately recorded before H_2 addition. The tube was then degassed by the freeze/pump/thaw method (x 2) and H_2 (1 atm) was added immediately. Upon inversion the red/brown solution began to decolourise and after 30 seconds of mixing the solution had become pale yellow. The ^{1}H NMR spectrum (section 5.4.2) showed the product to be Dipp-BDIAlH_2 (**S1**).

2.6.3 Control Reaction of H_2 with Dipp-BDIAl (2)

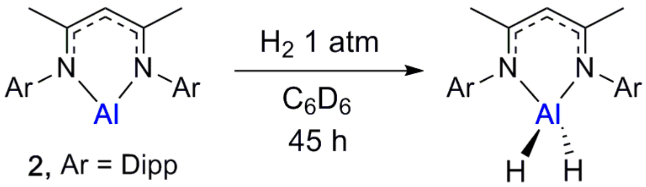

2, Ar = Dipp

2 (5.0 mg, 0.0113) was weighed into a small vial and C_6D_6 (0.55 mL) was added. The solution was mixed to dissolve the solid to form a red solution. The solution was transferred to a Young's tap NMR tube. The tube was sealed and the NMR spectrum immediately recorded before H_2 addition. The tube was then degassed by the freeze/pump/thaw method (x 2) and H_2 (1 atm) was added immediately. Upon mixing by inversion no colour change of the red solution was observed. The NMR spectrum recorded immediately showed little reaction of **2** (section 5.4.3). The solution was monitored with Dipp-BDIAlH_2 (**S1**) forming as the major product slowly over time and full consumption of **2** reached after 45 h at 25 °C.

3. X-ray Crystallographic Data

Table S1: Crystal Data, Data Collection and Refinement Parameters for the structures of **Pd_2Al_2, $PdAl_2$, 3, 4-o**

Compound	**Pd_2Al_2**	**$PdAl_2$**	**3a**
CCDC No.	1829418	1829419	1829420
Formula	$C_{82}H_{126}Al_2N_4P_2Pd_2$	$C_{85}H_{136}Al_2N_4PPd$	$C_{35}H_{47}AlN_2$
M	1496.56	1405.30	522.72
Crystal System	Triclinic	Triclinic	Orthorhombic
Space group	*P*-1 (2)	*P*-1 (2)	*Pnma* (62)
T [K]	173(2)	173(2)	173(2)
a [Å]	12.8924(6)	13.2046(8)	16.6858(14)
b [Å]	13.4295(6)	14.1589(11)	20.950(2)
c [Å]	13.7975(6)	23.4405(11)	9.0089(9)
α [°]	110.488(4)	94.720(5)	90
β [°]	112.313(4)	98.247(4)	90
γ [°]	100.712(4)°	108.643(6)	90
V [$Å^3$]	1925.07(16)	4071.0(5)	3149.1(5)
Z	1[5]	2	4
Density [g cm^{-3}]	1.291	1.146	1.103
Radiation Used	Mo-Kα	Mo-Kα	Mo-Kα
μ (mm^{-1})	0.576	0.313	0.089
θ range [°]	$2.429 \le \theta \le 28.286$	$2.497 \le \theta \le 28.248$	$2.441 \le \theta \le 28.331$
Reflns collected	11174	23629	6552
R_{int}	0.0205	0.0373	0.0401
Completeness (to θ = 25.242°)	98.5%	98.8%	99.1%
No. of data/restr/param	7609 / 0 / 427	16085/5/861	6552/0/187
R_1 [$I > 2\sigma(I)$]	0.0350	0.0679	0.0479
wR_2 [all data]	0.0775	0.1662	0.1371
GoF	1.046	1.044	0.699
Largest diff. pk and hole [e$Å^{-3}$]	0.377, -0.369	1.053, -1.087	0.480, -0.477

[5] The value of Z = 1 is due to the complex having crystallographic C_i symmetry.

Table S1 cont: Crystal Data, Data Collection and Refinement Parameters for the structures of **Pd_2Al_2, $PdAl_2$, 3, 4o**

Compound	**4-o**
CCDC No.	1829421
Formula	$C_{36}H_{48.9}AlCl_{0.1}N_2$
M	540.19
Crystal System	Monoclinic
Space group	$P2_1/c$ (14)
T [K]	173(2)
a [Å]	12.0860(4)
b [Å]	22.1931(7)
c [Å]	12.9055(4)
α [°]	90
β [°]	106.077(4)
γ [°]	90
V [Å^3]	3326.2(2)
Z	4
Density [g cm^{-3}]	1.079
Radiation Used	Mo-Kα
μ (mm^{-1})	0.094
θ range [deg]	$2.539 \leq \theta \leq 28.215$
Reflns collected	11620
R_{int}	0.0486
Completeness (to θ = 25.242°)	98.7%
No. of data/restr/param	6657/7/376
R_1 [$I > 2\sigma(I)$]	0.0589
wR_2 [all data]	0.1540
GoF	1.099
Largest diff. pk and hole [eÅ^{-3}]	0.459, -0.242

Table S1 provides a summary of the crystallographic data for the structures of **Pd_2Al_2, $PdAl_2$, 3, 4o**. Data were collected using an Agilent Xcalibur 3 E diffractometer, and the structures were refined using the SHELXTL and SHELX-2014 program systems.[6]

[6] SHELXTL v5.1, Bruker AXS, Madison, WI, 1998. SHELX-2014, G.M. Sheldrick, *Acta Cryst.*, 2015, **C71**, 3-8.

The X-ray crystal structure of Pd_2Al_2

The structure of **Pd_2Al_2** was found to sit across a centre of symmetry at the middle of the **Pd_2Al_2** ring. The unique Al–H–Pd bridging hydrogen atom was located from a Δ*F* map and refined freely.

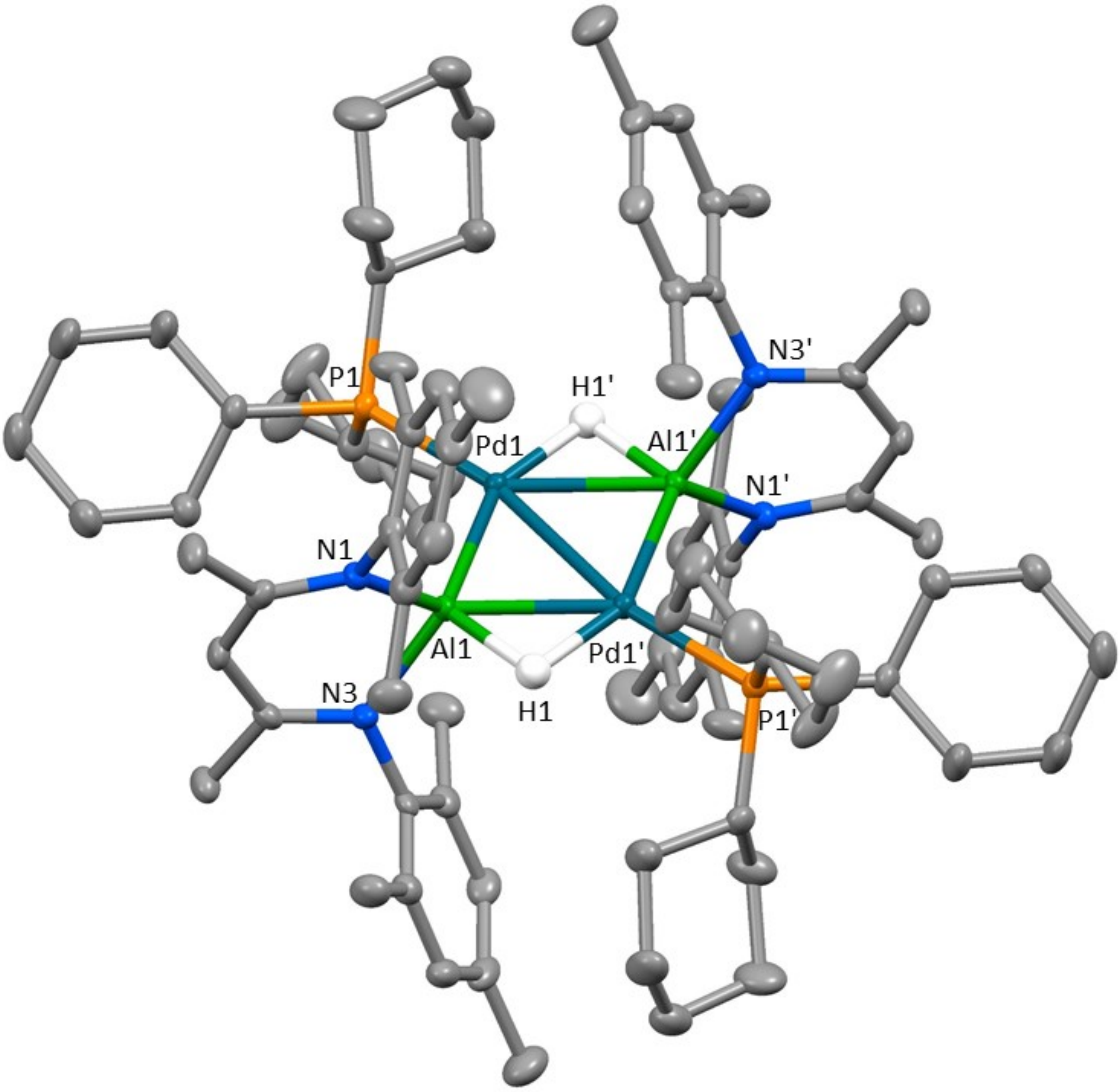

Figure S1: The crystal structure of **Pd_2Al_2** (50% probability ellipsoids). Selected hydrogen atoms omitted for clarity.

The X-ray crystal structure of $PdAl_2$

The data collected for **$PdAl_2$** suggested the possibility of crystal twinning. Attempts were made to resolve and model the twinning but the results proved less satisfactory than treating the data as not twinned. The result of this poor data quality is a slightly large R_1 value (0.0679). This also resulted in residual electron density close to the Pd atom. The unit cell was found to contain 3 molecules of *n*-hexane solvent of crystallisation.

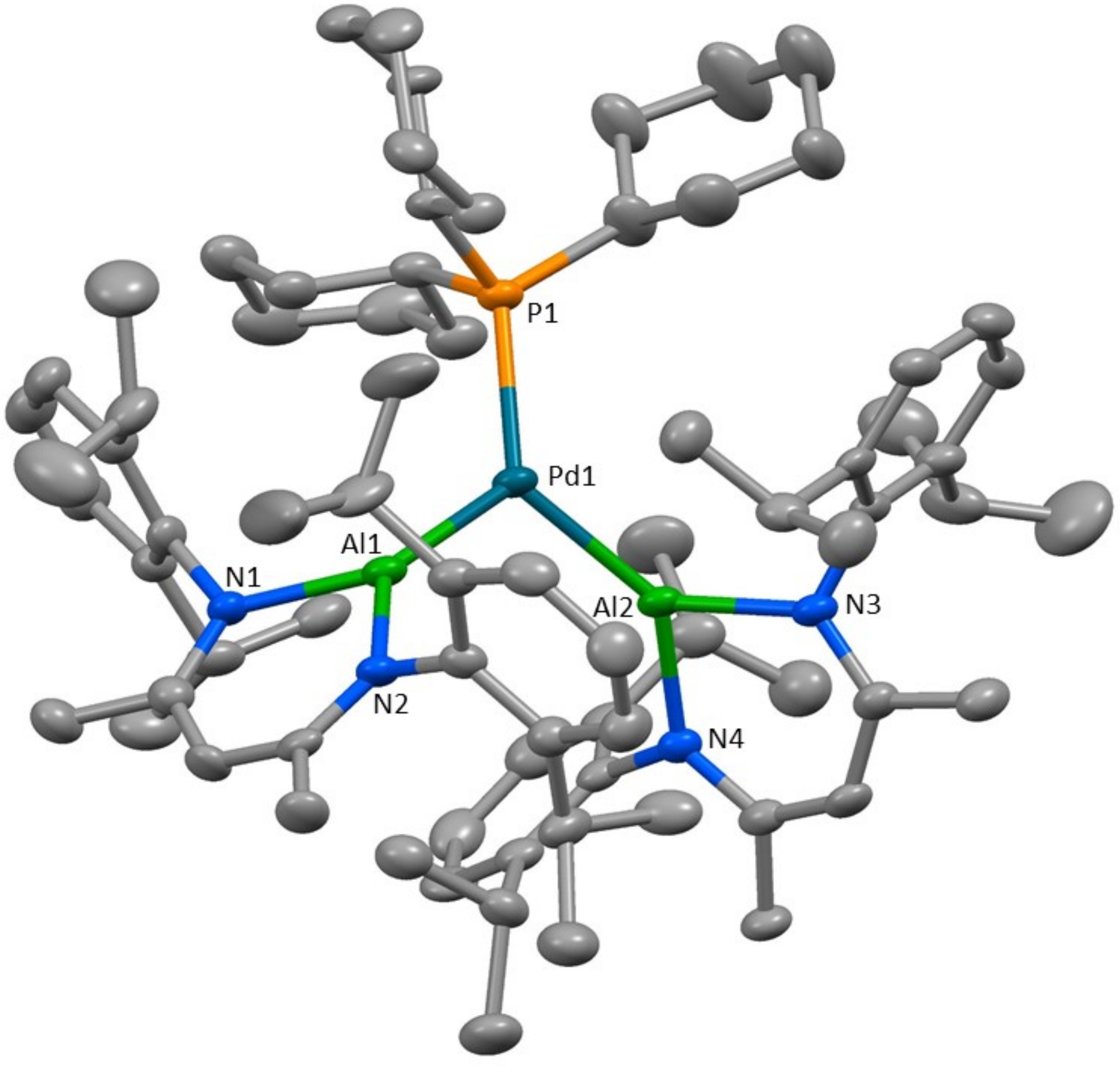

Figure S2: The crystal structure of **$PdAl_2$** (50% probability ellipsoids). Hydrogen atoms and solvent of crystallization omitted for clarity.

The X-ray crystal structure of 3a

The data collected for **3a** suggested the possibility of crystal twinning. The structure was refined as a 2 component twin with a ratio of 56:44. The molecule sits on a mirror plane which lies in the plane of Al and bisects the supporting ligand and the Al-bound phenyl group. The Al–H hydrogen atom in the structure of **3a** was located from a Δ*F* map and refined freely.

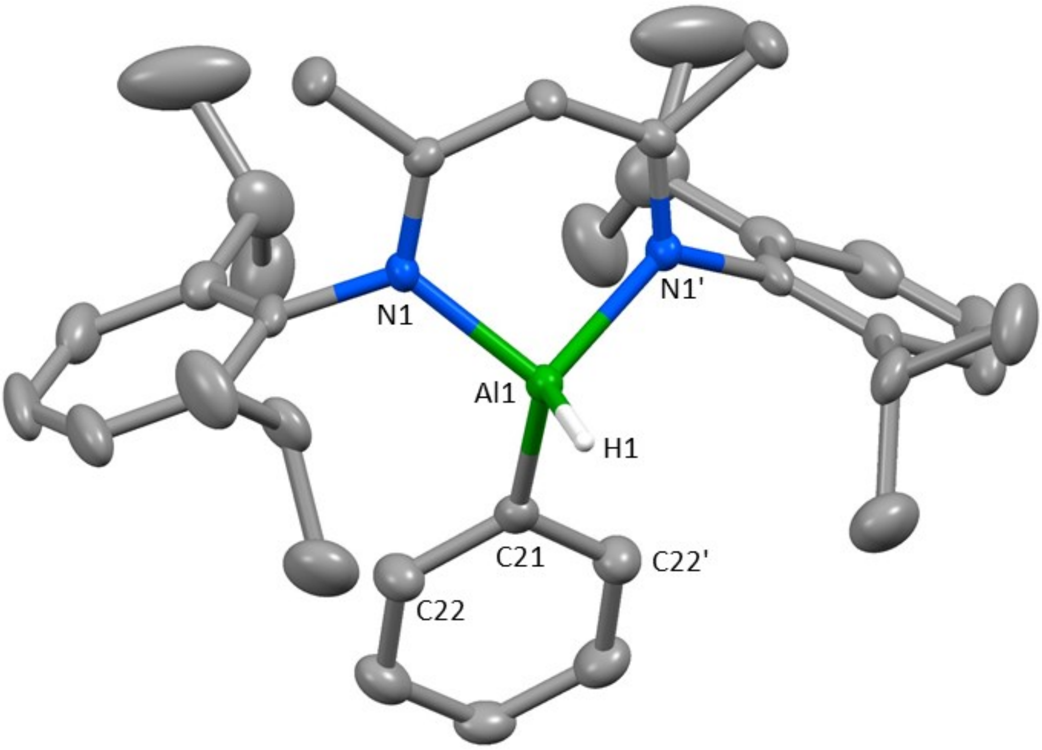

Figure S3: The crystal structure of **3a** (50% probability ellipsoids). Selected hydrogen atoms omitted for clarity.

The X-ray crystal structure of 4-o

The data for **4-o** showed more electron density adjacent to the Al centre than caused by a hydride ligand. The likely explanation was a partial occupancy chloride (contained in the starting materials for **4-o**) which was modelled at 10% occupancy with 90% occupancy of the hydride. This co-crystallisation of aryl-aluminium hydrides with aryl-aluminium halides has been observed previously in X-ray structures (and supported by NMR spectroscopy) and was modelled in the same way here.[7] The position and thermal parameters of the hydride were modelled using the DFIX and SIMU commands.

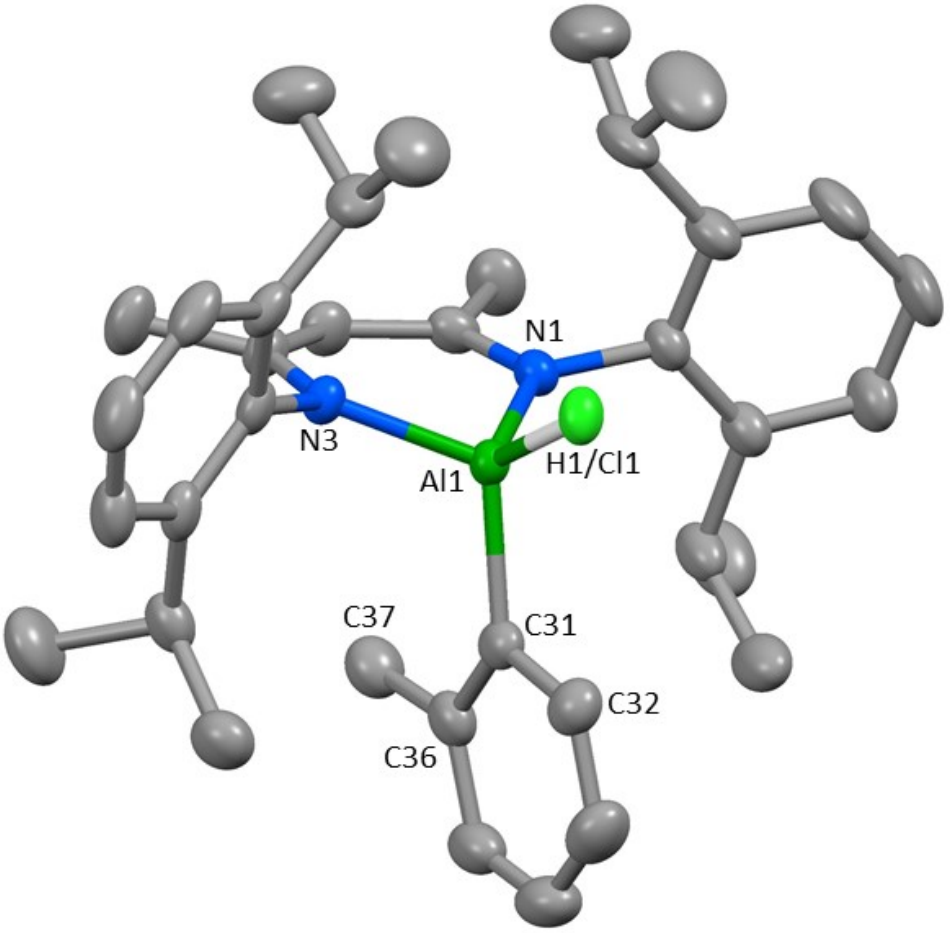

Figure S4: The crystal structure of **4-o** (50% probability ellipsoids). Selected hydrogen atoms omitted for clarity.

[7] S. Yow, S. J Gates, A. J. P. White. M. R. Crimmin, *Angew. Chem., Int. Ed.* **2012**, *51*, 12599.

4. Kinetics Studies by NMR Spectroscopy

4.1 Kinetic Isotope Effect of Paladium Catalysed C–H Alumination of Benzene

Experimental Procedure

2 (5.0 mg, 0.0113 mmol) was weighed into a vial and dissolved in either C_6H_6 or C_6D_6 (437 µL). A solution of [Pd(PCy$_3$)$_2$] (113 µL, 0.01 M solution in C_6H_6 or C_6D_6, 0.00113 mmol, 10 mol%) was added. The solution was mixed and transferred to a Young's NMR tube containing a capillary insert standard (ferrocene in C_6D_6). The tube was sealed, removed from the glove box and cooled in ice while transported directly to the NMR spectrometer. The sample was warmed to room temperature and loaded into the spectrometer pre-set at 30 °C. The sample was locked and shimmed and the first ^{1}H NMR spectrum recorded ~5 min after mixing.

A plot of [**2**] (determined from initial concentration and integration against internal standard) vs time for both reactions using C_6H_6 or C_6D_6 showed a straight line (figure S5) indicating the reaction is (pseudo)-zero order in [**2**]. Standard errors were calculated by use of the regression analysis calculation in Microsoft Excel software. The rate constant for the C_6H_6 reaction was found to be $k_{C\text{-}H}$ = 4.86 x 10^{-6} (± 1.09 x 10^{-7}) mol dm^{-3} s^{-1} and $k_{C\text{-}D}$ = 8.43 x 10^{-7} (± 4.13 x 10^{-9}) mol dm^{-3} s^{-1} for C_6D_6. This gave a KIE of 5.8 (± 0.1) for the reaction indicative of a strong primary KIE. While this value is large for a KIE in comparison to many in the literature,[8] the maximum value for k_H/k_D is estimated to be 6.5-7.0 at 298 K[9] and is consistent with the proposed mechanism in which the oxidative addition of the C–H bond is the highest transition state.

[8] E.M. Simmons and J.F. Hartwig, *Angew. Chem. Int. Ed.*, **2012**, *51*, 3066.
[9] M. Gómez-Gallego and M.A. Sierra, *Chem. Rev.*, **2011**, *111*, 4857.

Figure S5. Kinetic study of the C–H vs C–D alumination reaction.

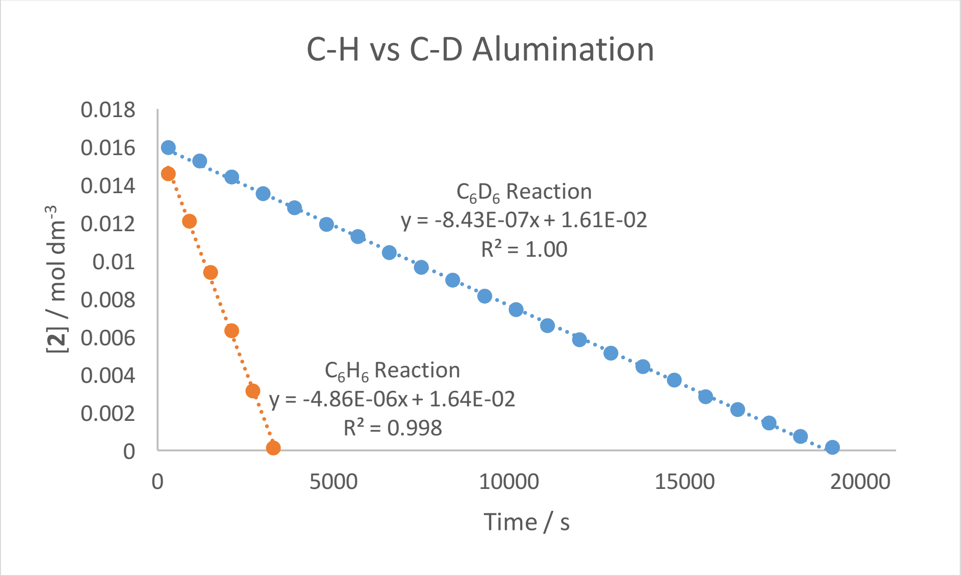

4.2 Eyring Analysis of Benzene C–H Alumination

Experimental Procedure

2 (437 μL, 0.0258 M solution in C_6H_6, 0.0113 mmol) was mixed with a solution of [Pd(PCy_3)$_2$] (113 μL, 0.01 M solution in C_6H_6, 0.00113 mmol, 10 mol%). The solution was rapidly transferred to a Young's NMR tube containing a capillary insert standard (ferrocene in C_6D_6). The tube was sealed, removed from the glove box and cooled in ice while transported directly to the NMR spectrometer. The sample was warmed to room temperature and loaded into the spectrometer pre-set at the required temperature. The sample was locked and shimmed and the first ^{1}H NMR spectrum recorded ~5 min after mixing.

A plot of [**2**] (determined from initial concentration and integration against internal standard) vs time for the reactions at different temperatures showed a straight lines (figure S6) indicating the reaction is (pseudo)-zero order in [**2**]. The rate constants were calculated and a plot of ln k vs $1/T$ (figure S7) allowed calculation of the thermodynamic parameters using the Eyring equation. Standard errors were calculated by use of the regression analysis calculation in Microsoft Excel software. The enthalpy of activation was found to be $\Delta H^{\ddagger}$ = 23.4 (± 1.3) kcal mol^{-1} and the entropy of activation $\Delta S^{\ddagger}$ = -5.7 (± 4.5) cal K^{-1} mol^{-1}. This gave a value for the Gibbs free energy of activation of $\Delta G^{\ddagger}_{298\ K}$ = 25.1 (± 2.6) kcal mol^{-1}.

Figure S6. Kinetic study of the C–H alumination reaction at a range of temperatures.

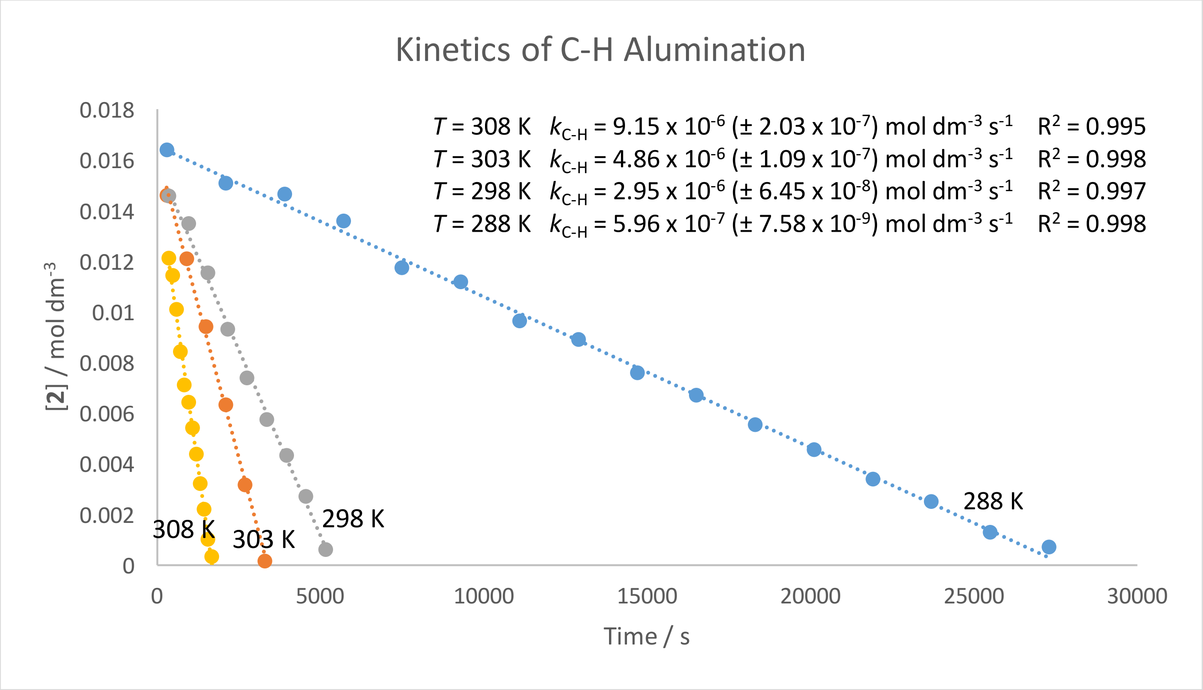

Figure S7. Eyring Plot of C–H alumination reaction.

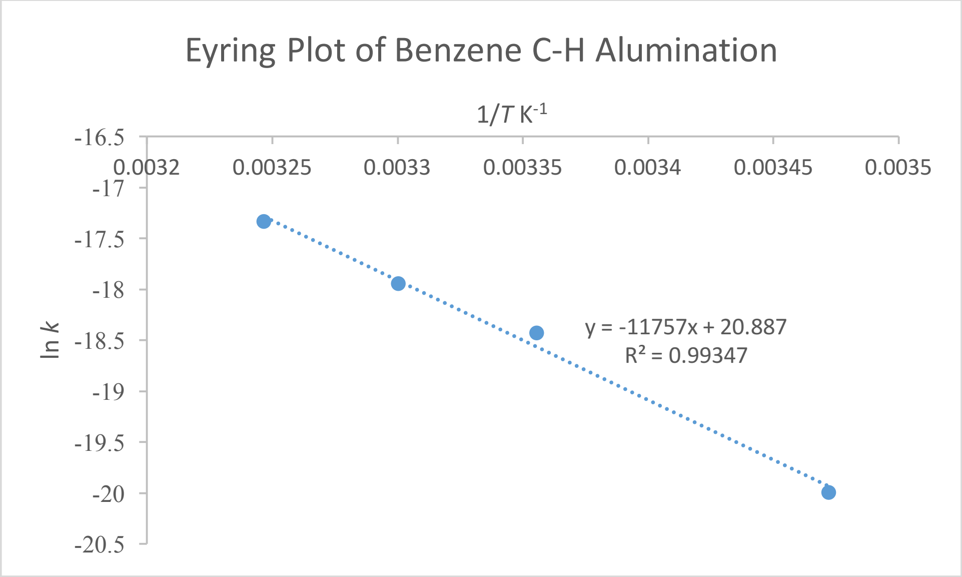

5. Multinuclear NMR Data

5.1 NMR Spectra of Heterometallic Products

5.1.1 NMR Spectrum of Reaction Mixture of Pd_2Al_2

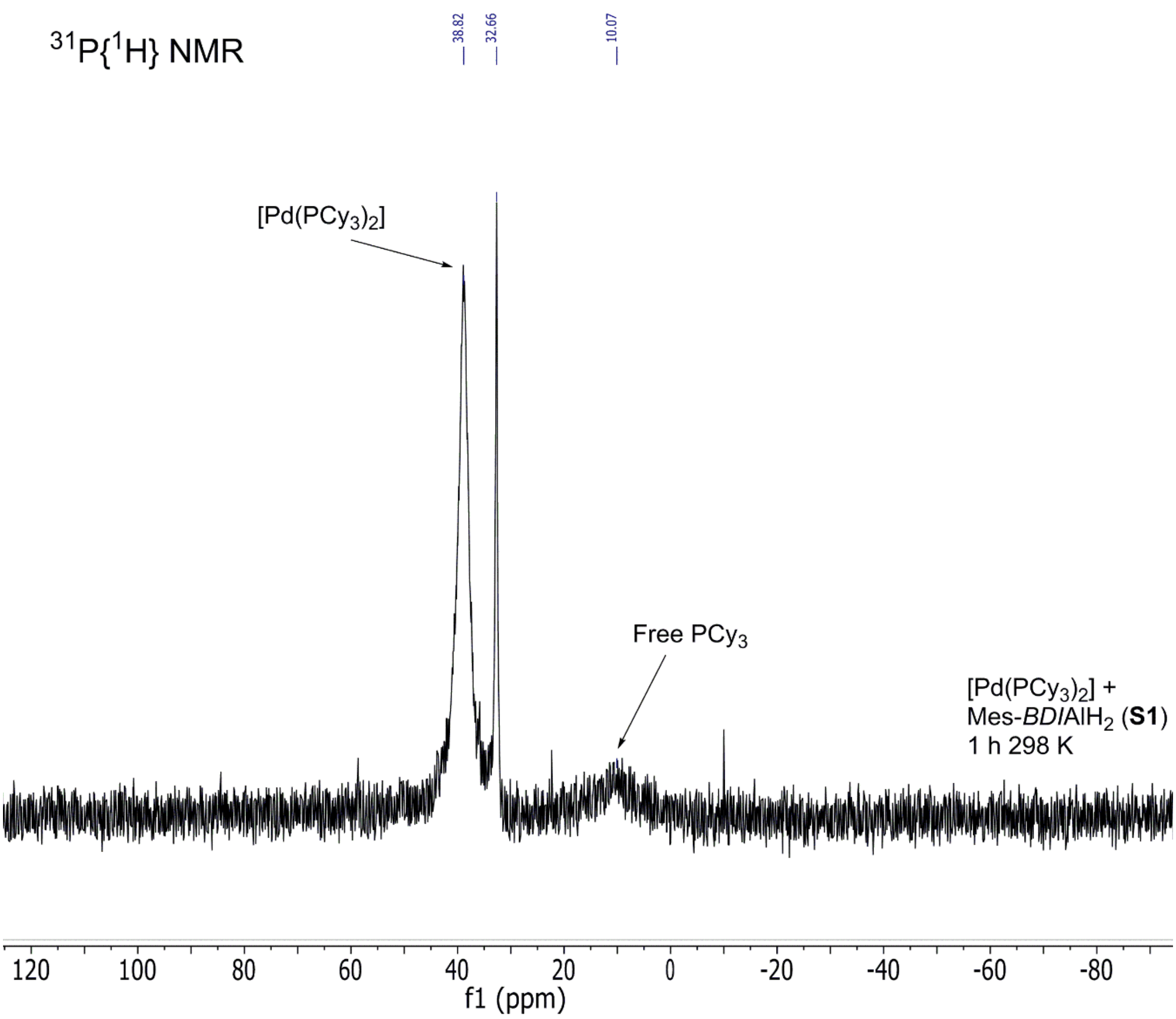

Figure S8. $^{31}P\{^1H\}$ NMR spectrum of the reaction mixture of $[Pd(PCy_3)_2]$ with Mes-*BDI*AlH_2 (**S1**) in C_6D_6 after 1 h at 298 K and before precipitation of $\mathbf{Pd_2Al_2}$. Broadened signals for $[Pd(PCy_3)_2]$ and PCy_3 are observed suggesting a dynamic process in solution.

5.1.2 NMR Spectra of Pure $PdAl_2$ at 298 K in d_8-toluene

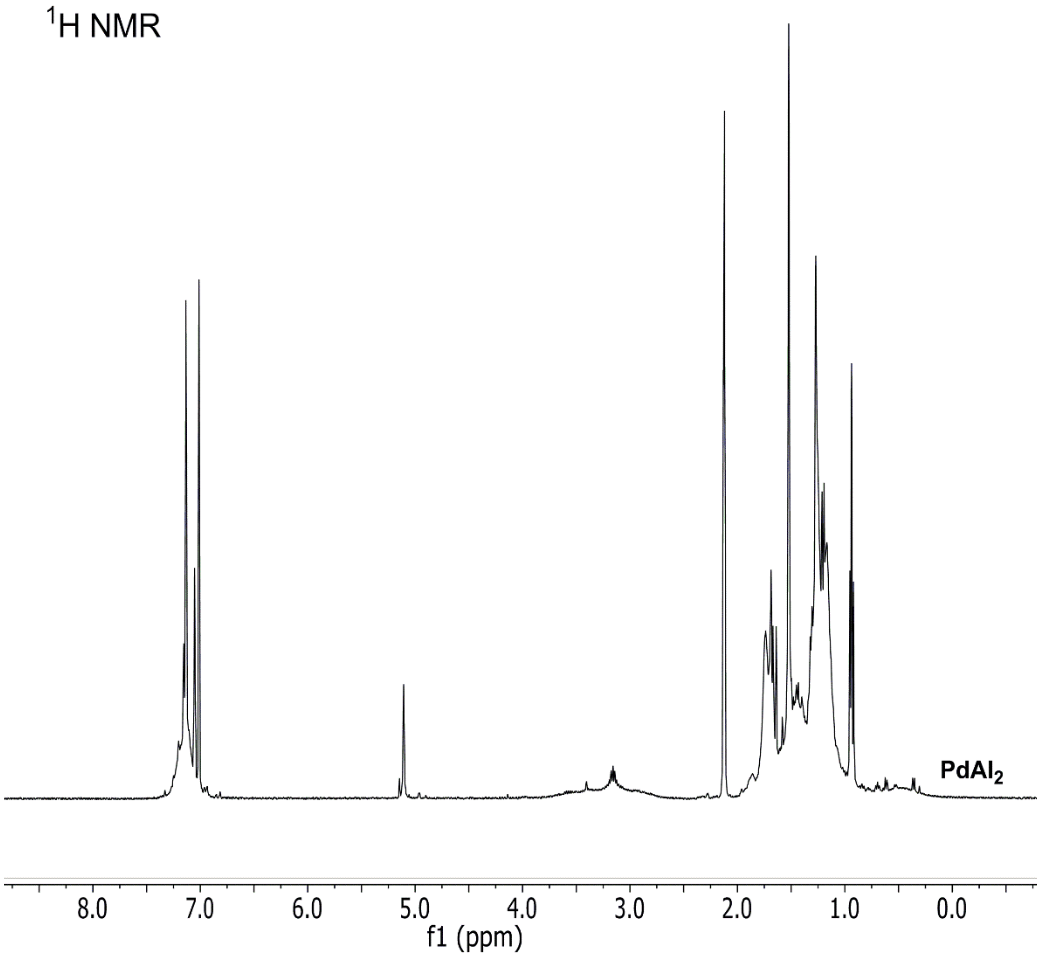

Figure S9. ^{1}H NMR spectrum of a pure sample of **$PdAl_2$** in d_8-toluene at 298 K showing a dynamic process in solution.

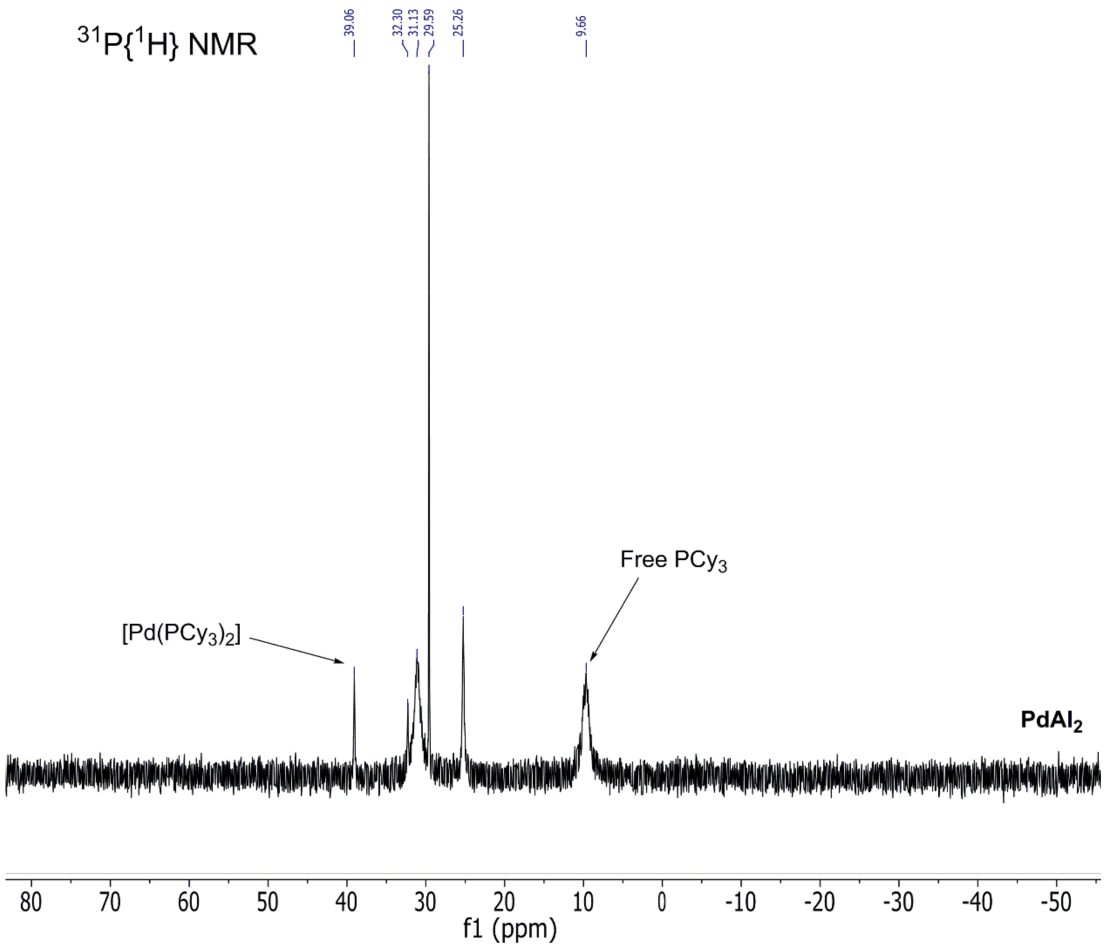

Figure S10. $^{31}P\{^1H\}$ NMR spectrum of a pure sample of $\mathbf{PdAl_2}$ in d_8-toluene at 298 K showing a dynamic process in solution.

5.1.3 NMR Spectra of $PdAl_2$ + 1 equiv. of 2 at 233 K in d_8-toluene

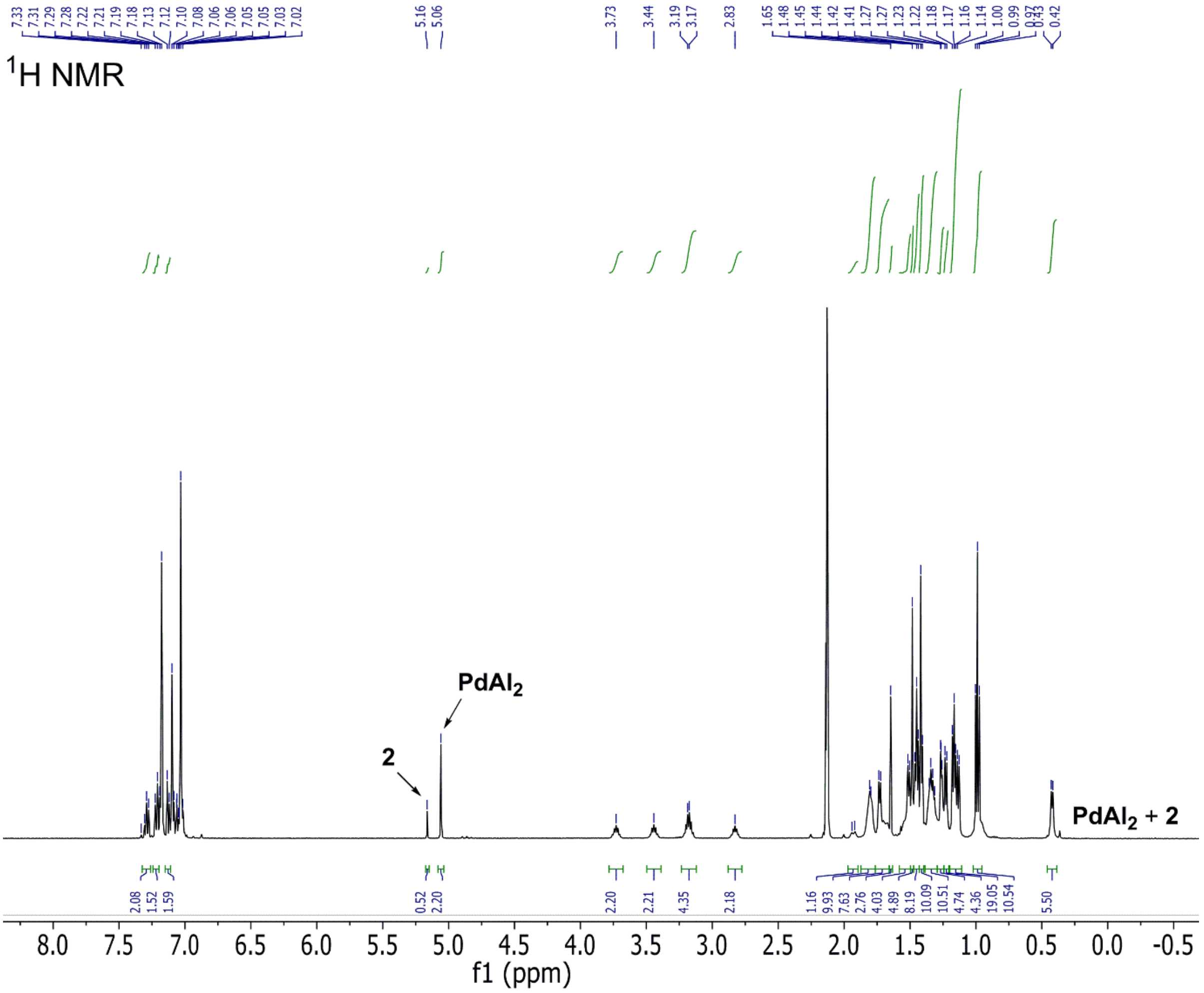

Figure S11. ^{1}H NMR spectrum of a sample of **$PdAl_2$** with 1 equiv. of **2** in d_8-toluene at 233 K.

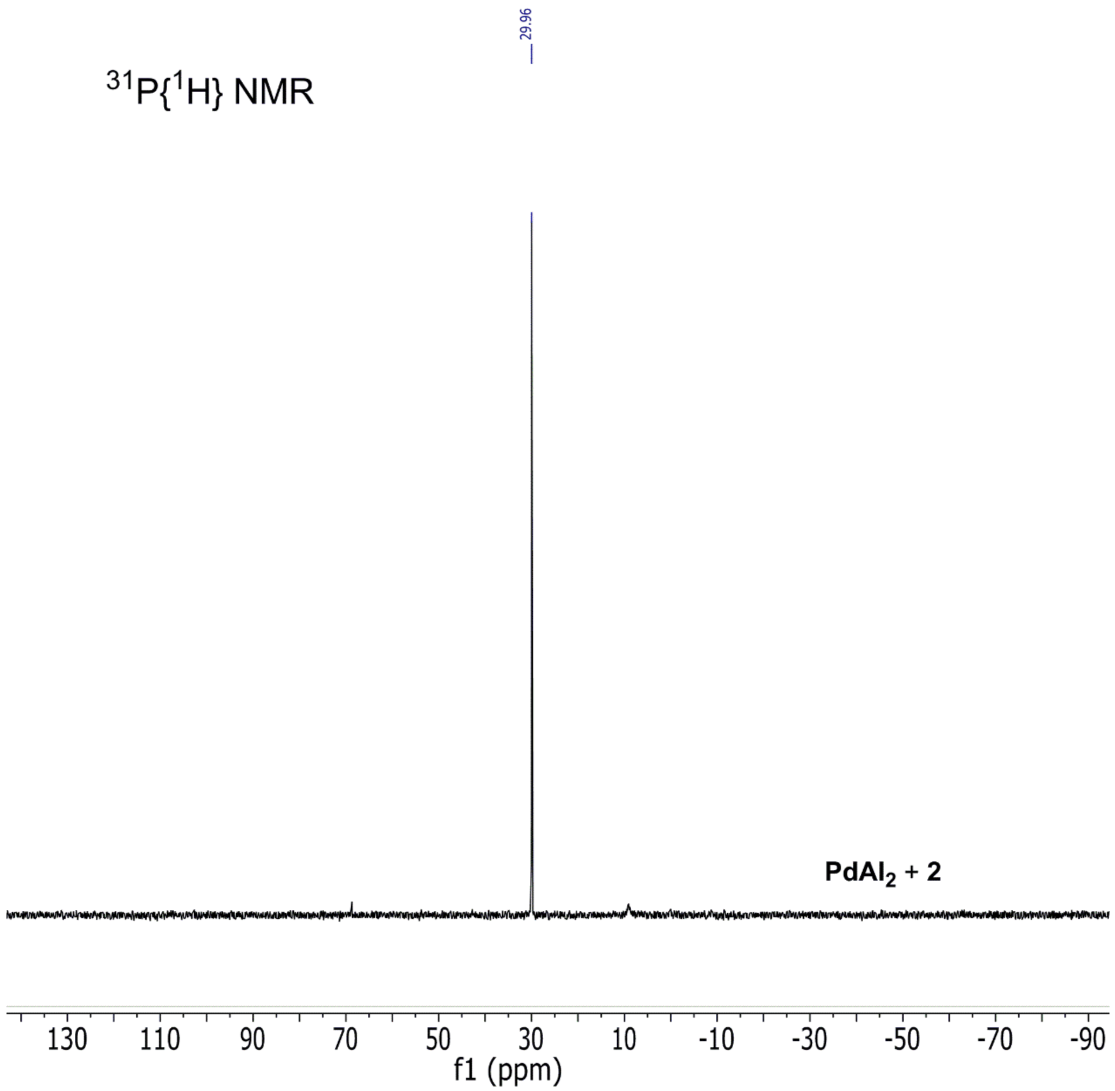

Figure S12. $^{31}P\{^1H\}$ NMR spectrum of a sample of **$PdAl_2$** with 1 equiv. of **2** in d_8-toluene at 233 K.

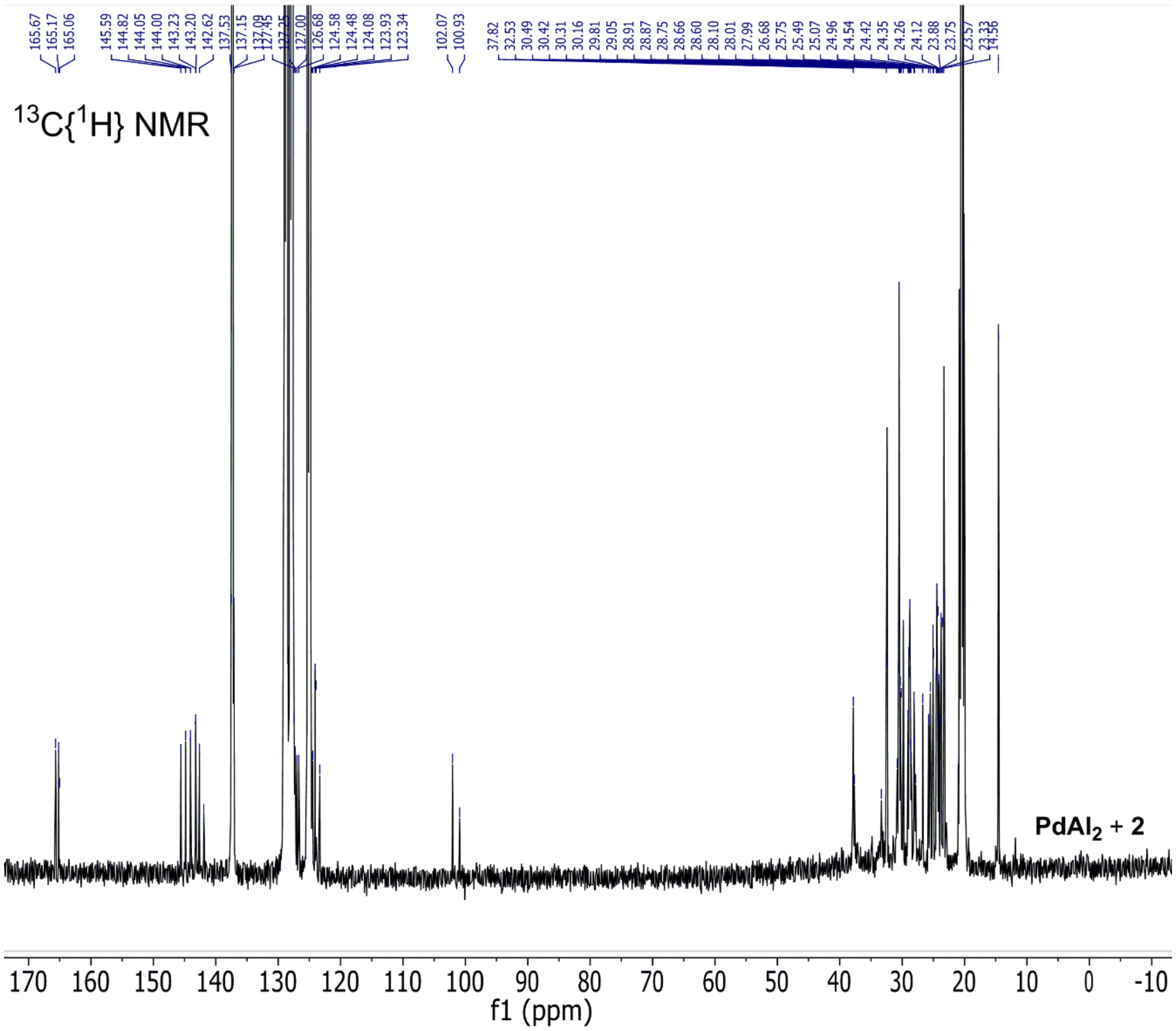

Figure S13. $^{13}C\{^1H\}$ NMR spectrum of a sample of **PdAl$_2$** with 1 equiv. of **2** in d_8-toluene at 233 K.

5.2 Stacked Plots Comparing ^{1}H NMR Spectra of Aluminated Products Synthesised Catalytically and Non-Catalytically

5.2.1 ^{1}H NMR Spectra of 3a

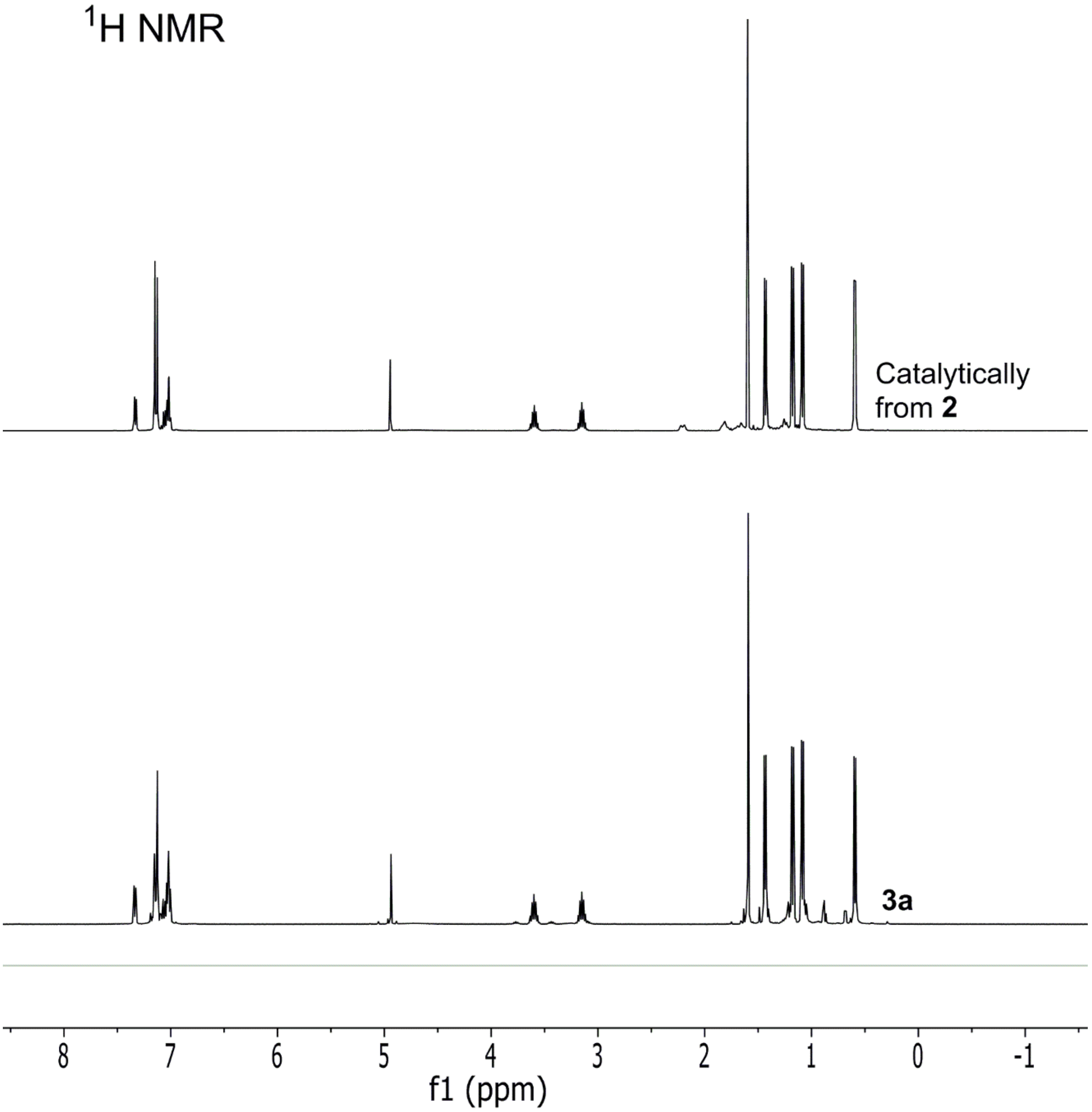

Figure S14. ^{1}H NMR spectra of **3a** catalytically generated from **2** and isolated sample of **3a**, synthesised non-catalytically.

5.2.2 ^{1}H NMR Spectra of 4-o, 4-m and 4-p

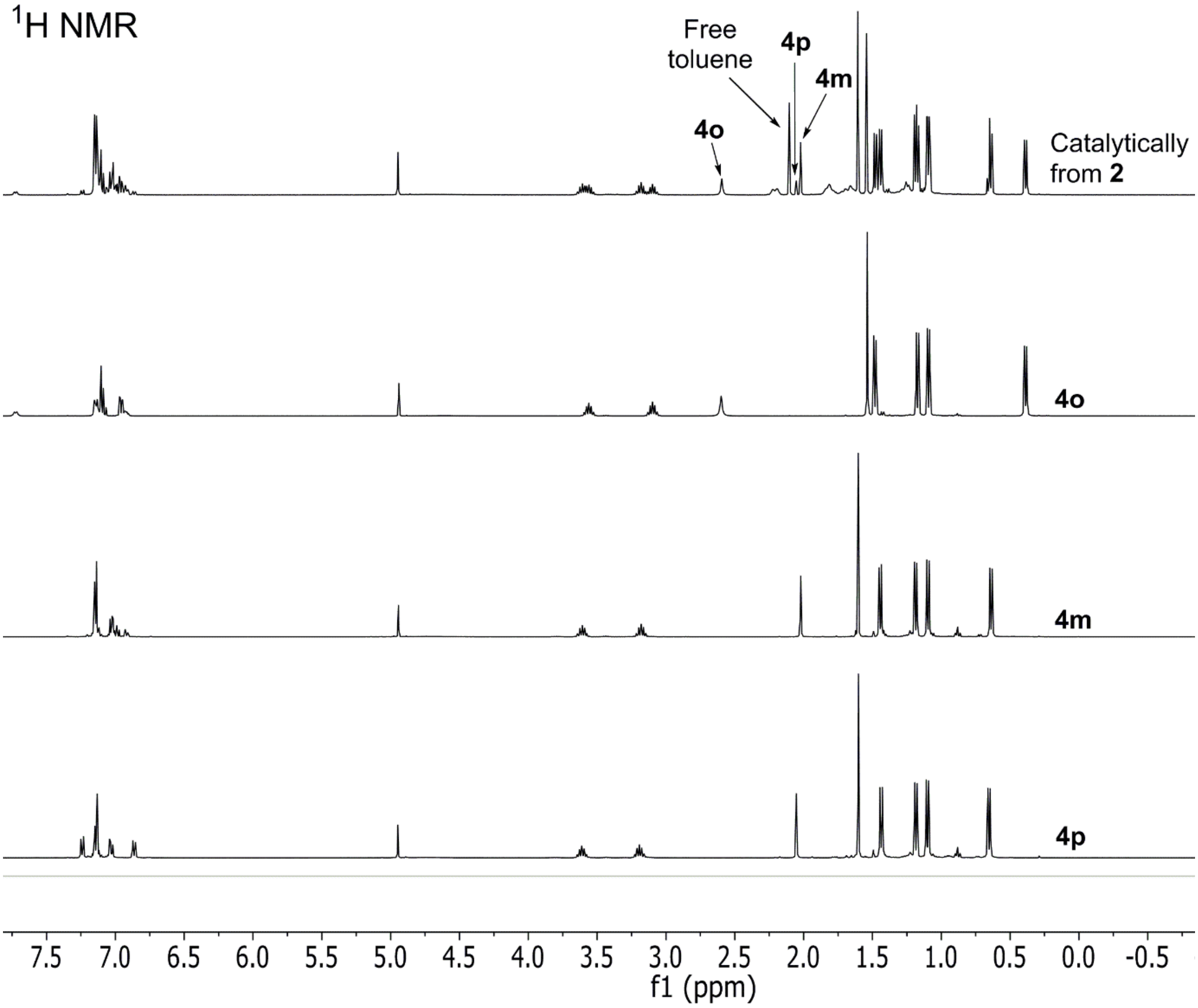

Figure S15. ^{1}H NMR spectra of the mixture of regioisomers catalytically generated from **2** and isolated samples of **4-o**, **4-m** and **4-p** synthesised non-catalytically.

5.2.3 ^{1}H NMR Spectra of 6-o and 6-m

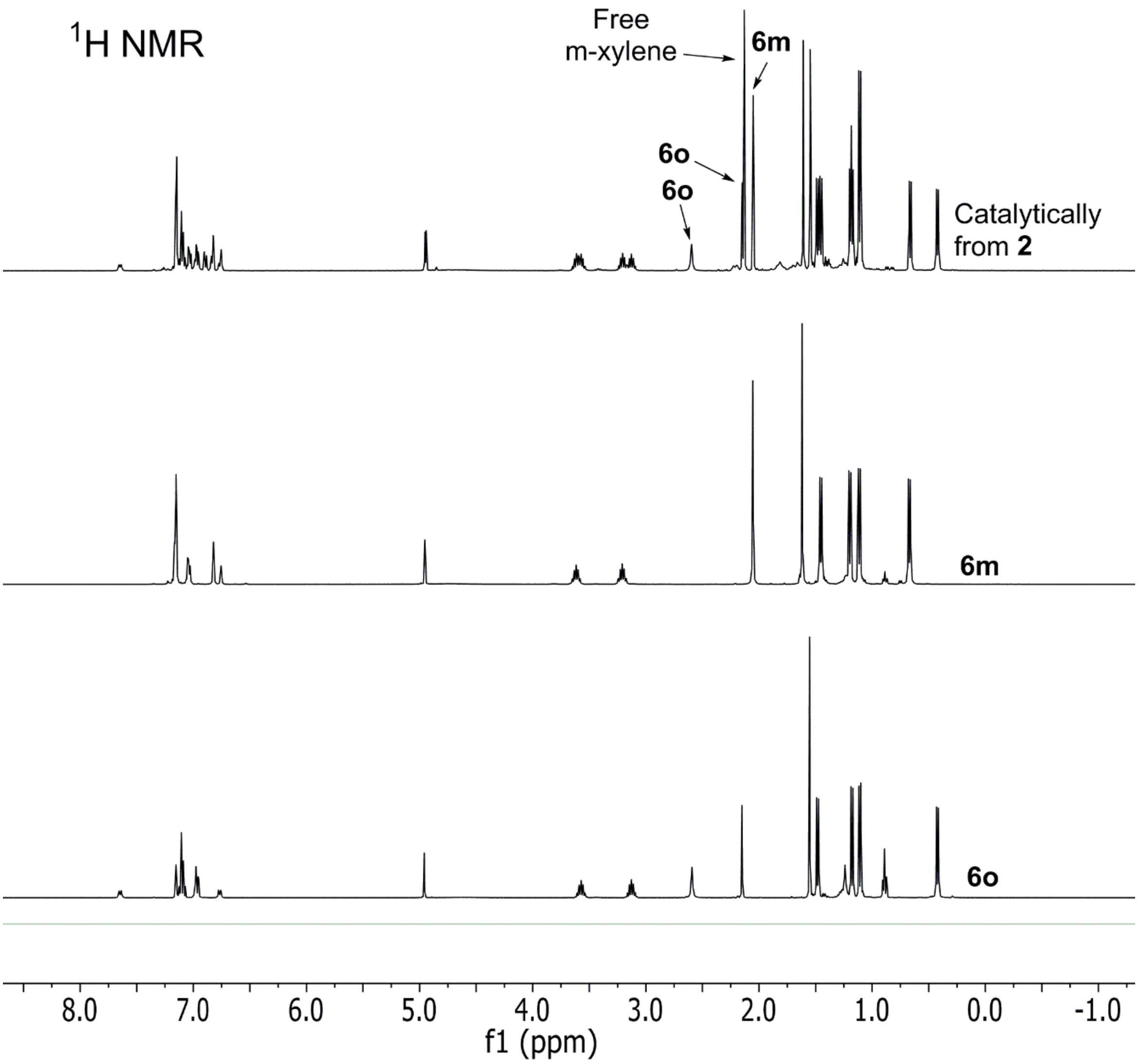

Figure S16. ^{1}H NMR spectra of the mixture of regioisomers catalytically generated from **2** and isolated samples of **6-o** and **6-m** synthesised non-catalytically.

5.2.4 ¹H NMR Spectra of 7-o and 7-m

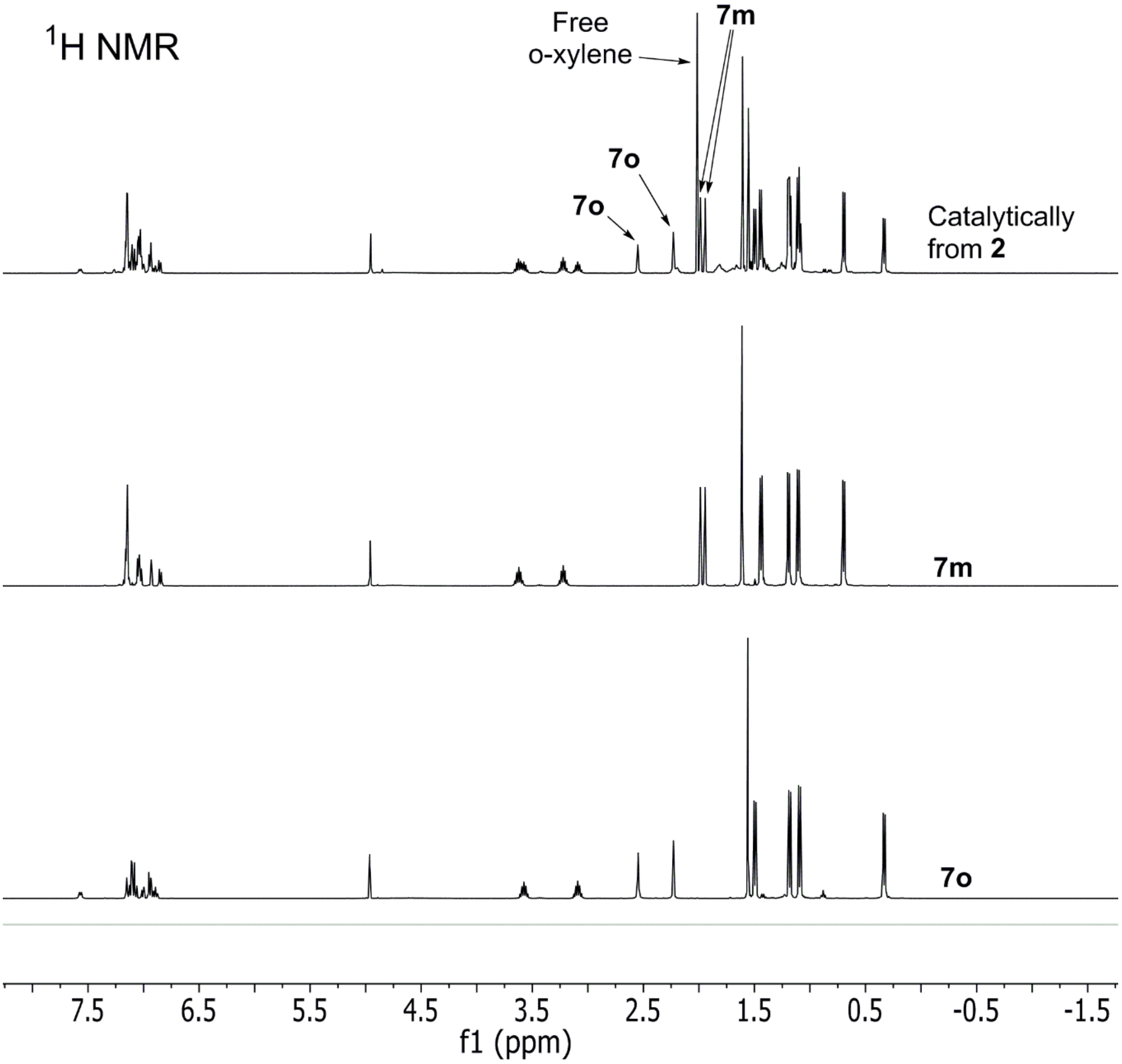

Figure S17. ¹H NMR spectra of the mixture of regioisomers catalytically generated from **2** and isolated samples of **7-o** and **7-m** synthesised non-catalytically.

5.2.5 ^{1}H NMR Spectra of 8-m and 8-p

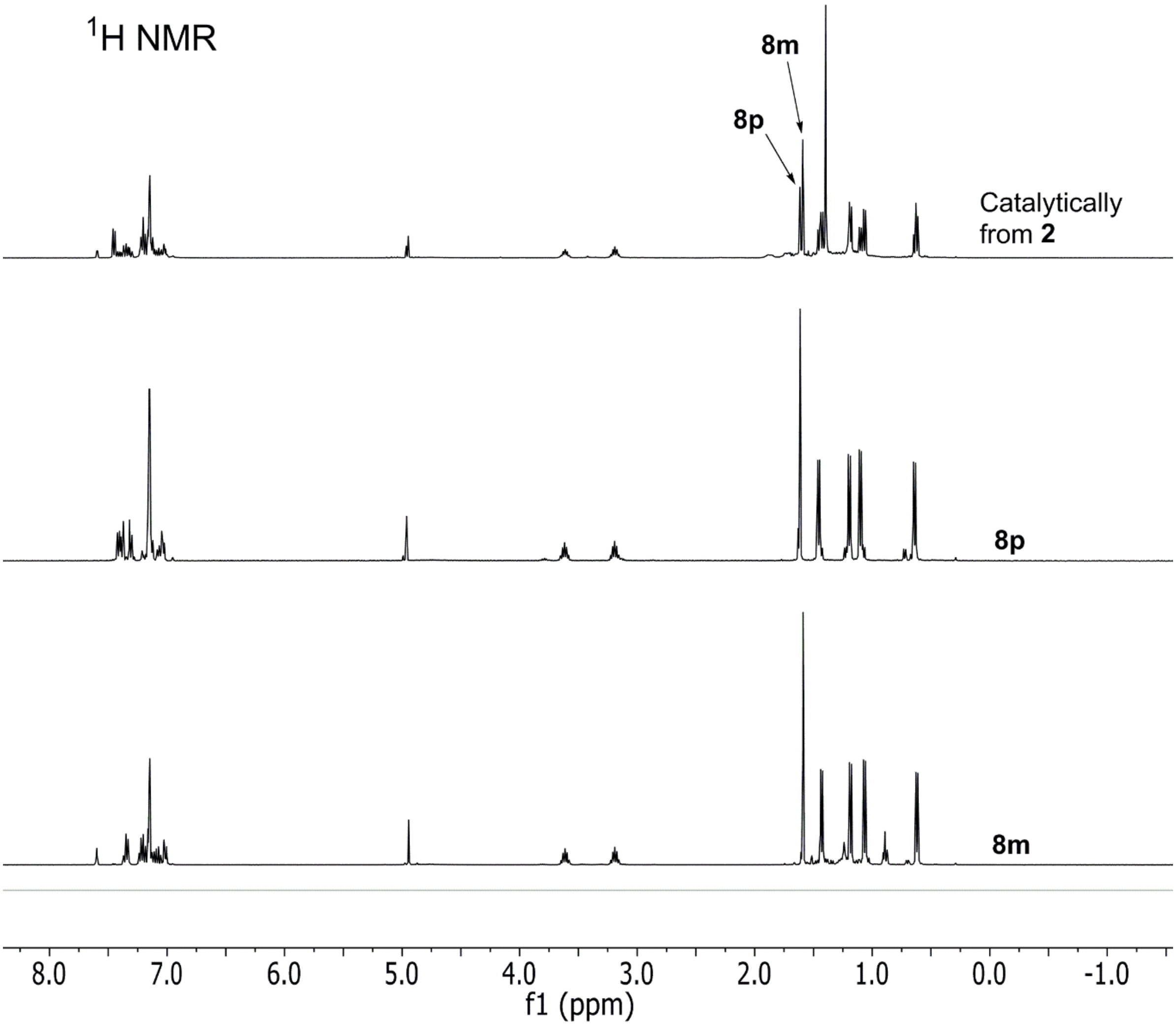

Figure S18. ^{1}H NMR spectra of the mixture of regioisomers catalytically generated from **2** and isolated samples of **8-p** and **8-m** synthesised non-catalytically.

5.3 C–H Alumination of Toluene Using a Phosphine Free Catalyst

5.3.1 ^{1}H NMR Spectra of Toluene Alumination Using Different Pd Catalysts

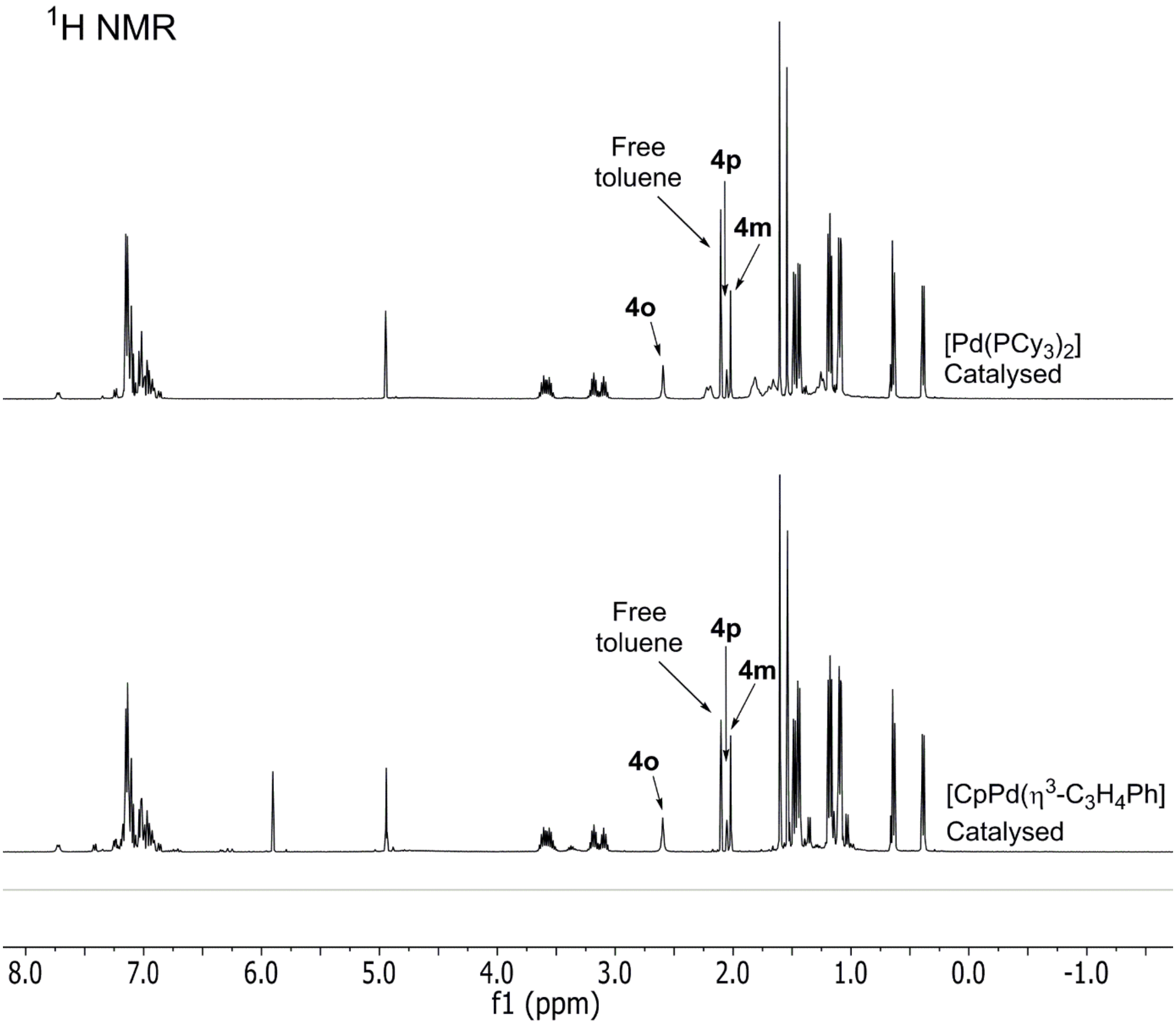

Figure S19. ^{1}H NMR spectra of the products (**4-o**, **4-m** and **4-p**) of C–H alumination of toluene from **2** using $[Pd(PCy_3)_2]$ and $[CpPd(\eta^3\text{-}C_3H_4Ph)]$ as catalysts.

5.4 Palladium Catalysed Reactivity of Dipp-BDIAl (2) with H_2

5.4.1 NMR Spectra of Reaction of H_2 with $[Pd(PCy_3)_2]$ and 2 equiv. Dipp-BDIAl (2)

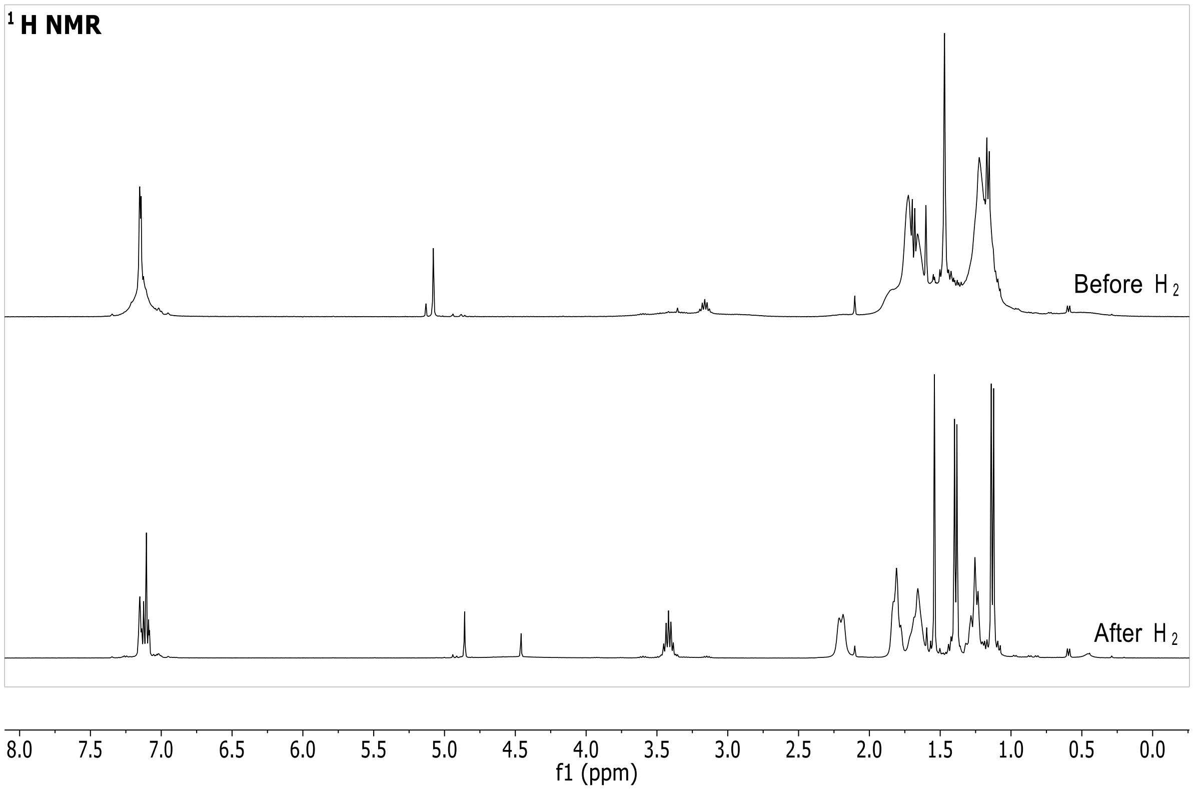

Figure S20: ^{1}H NMR spectrum of reaction of 2 equiv. of **2** and $[Pd(PCy_3)_2]$ with H_2 (1 atm).

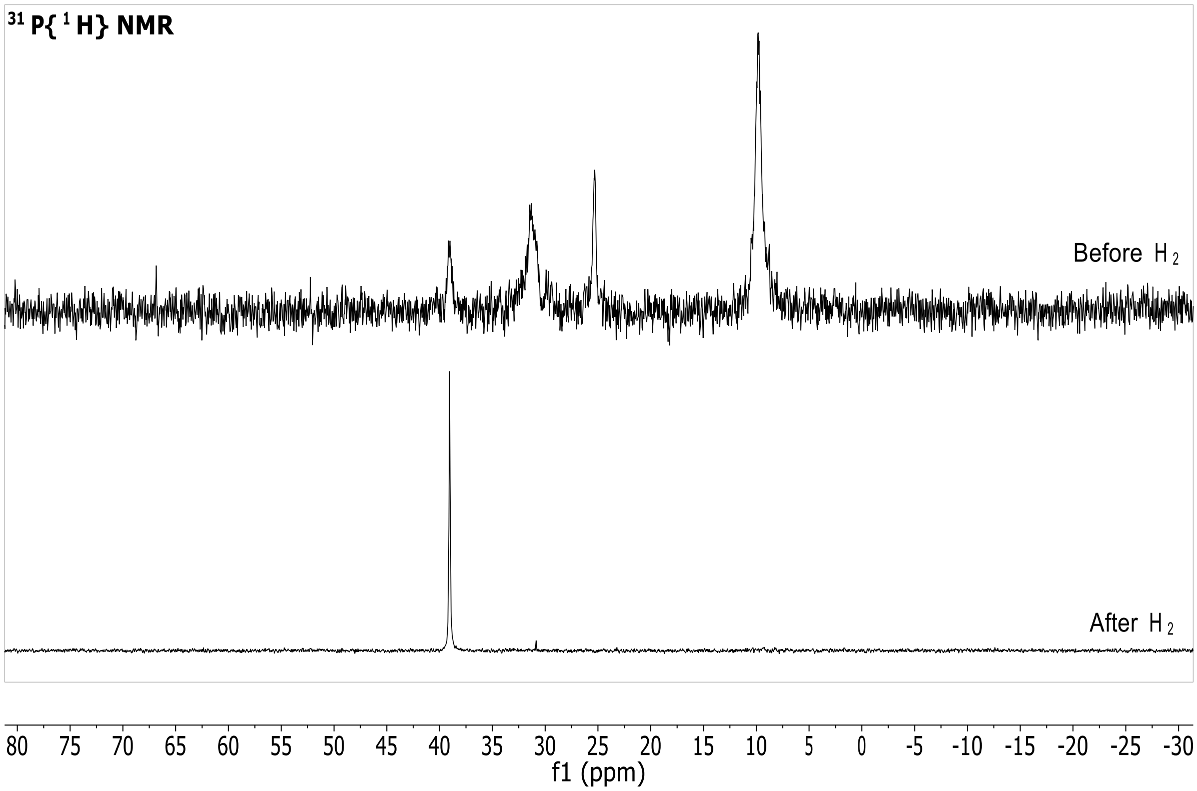

Figure S21: $^{31}P\{^1H\}$ NMR spectrum of reaction of 2 equiv. of **2** and $[Pd(PCy_3)_2]$ with H_2 (1 atm).

5.4.2 ^{1}H NMR Spectrum of Reaction of H_2 with [Pd(PCy_3)$_2$] and 20 equiv. Dipp-BDIAl (2)

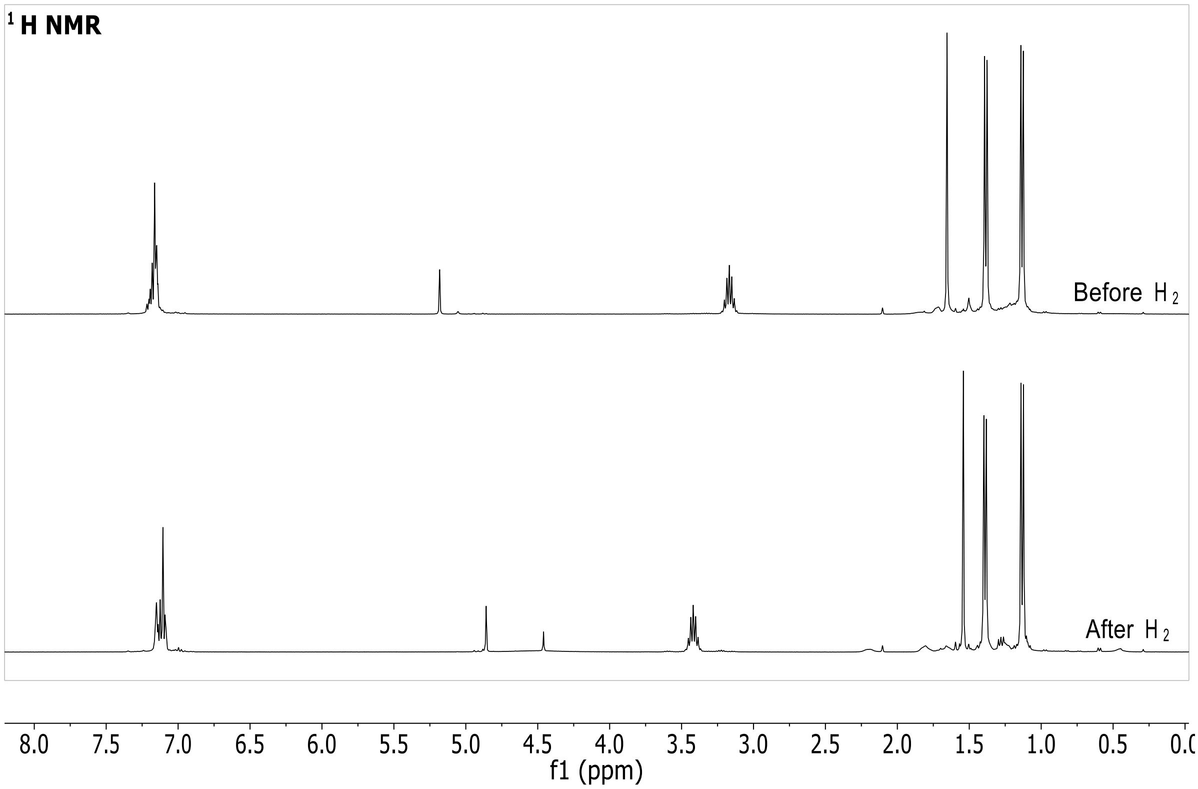

Figure S22: ^{1}H NMR spectrum of reaction of 20 equiv. of **2** and [Pd(PCy_3)$_2$] with H_2 (1 atm).

5.4.3 ^{1}H NMR Spectrum of Control Reaction of H_2 with Dipp-BDIAl (2)

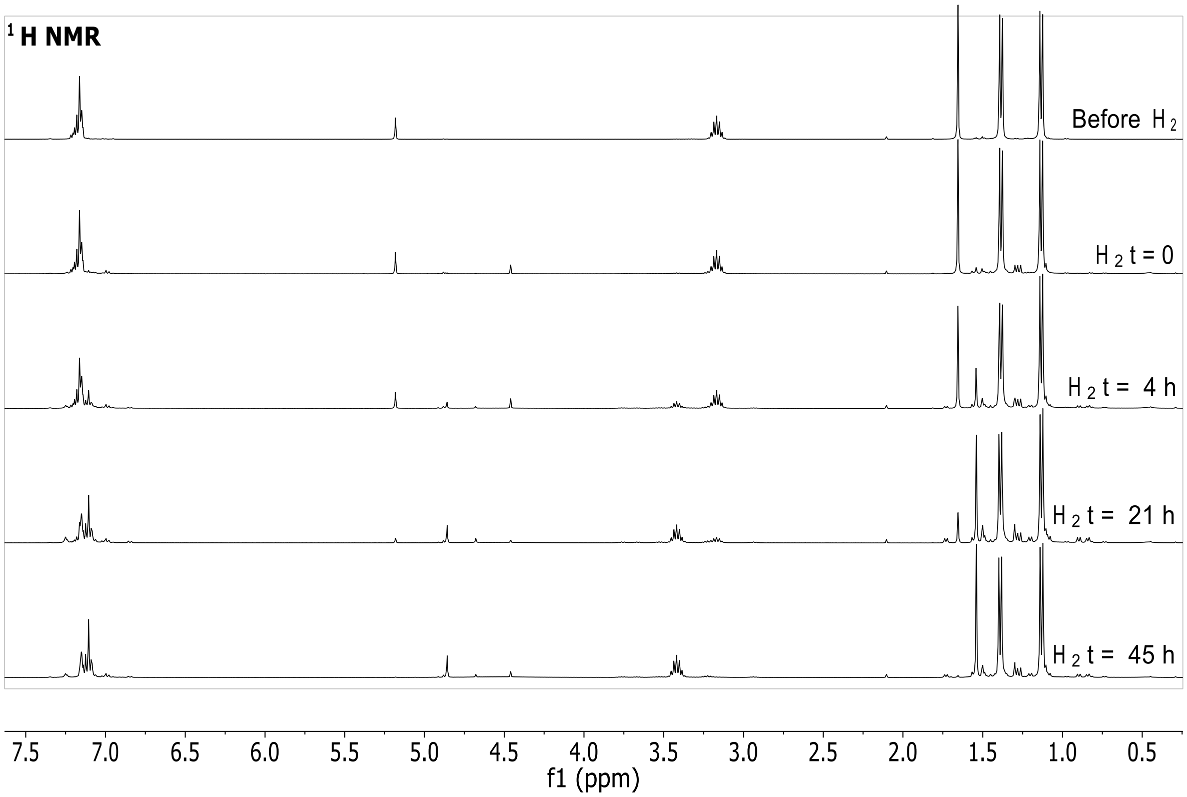

Figure S23: ^{1}H NMR spectrum of reaction of **2** with H_2 (1 atm).

6. Computational Details

6.1 Methods

The geometries of products were optimised with the M06L Minnesota DFT functional using the Gaussian09 program package.[10] NBO analysis was performed using the NBO 6.0 version program.[11] QTAIM analysis was conducted with the AIMAll package.[12] Non-covalent interactions were analysed with the NCIPLOT 3.0 program.[13] The ωB97x hybrid exchange-correlation DFT functional and the B3PW91 functional were also employed to assess differences in performance arising from the level of theory. Stationary points were characterised depending on their imaginary frequencies (0 for minima and 1 for TSs). A combined basis set was employed.

```
C H 0
6-31G*
****
N P 0
6-311+G*
****
127 0 (atom 127 is the hydride)
S 1 1.00
0.0360000 1.0000000
P 1 1.00
3.0000000 1.0000000
P 1 1.00
0.7500000 1.0000000
P 1 1.00
0.1875000 1.0000000
D 1 1.00
1.0000000 1.0000000
****
Pd Al 0
SDDAll
```

[10] Frisch, M. J.; Trucks, G. W.; Schlegel, H. B.; Scuseria, G. E.; Robb, M. A.; Cheeseman, J. R.; Scalmani, G.; Barone, V.; Mennucci, B.; Petersson, G. A.; Nakatsuji, H.; Caricato, M.; Li, X.; Hratchian, H. P.; Izmaylov, A. F.; Bloino, J.; Zheng, G.; Sonnenberg, J. L.; Hada, M.; Ehara, M.; Toyota, K.; Fukuda, R.; Hasegawa, J.; Ishida, M.; Nakajima, T.; Honda, Y.; Kitao, O.; Nakai, H.; Vreven, T.; Montgomery, J. A., Jr.; Peralta, J. E.; Ogliaro, F.; Bearpark, M.; Heyd, J. J.; Brothers, E.; Kudin, K. N.; Staroverov, V. N.; Kobayashi, R.; Normand, J.; Raghavachari, K.; Rendell, A.; Burant, J. C.; Iyengar, S. S.; Tomasi, J.; Cossi, M.; Rega, N.; Millam, J. M.; Klene, M.; Knox, J. E.; Cross, J. B.; Bakken, V.; Adamo, C.; Jaramillo, J.; Gomperts, R.; Stratmann, R. E.; Yazyev, O.; Austin, A. J.; Cammi, R.; Pomelli, C.; Ochterski, J. W.; Martin, R. L.; Morokuma, K.; Zakrzewski, V. G.; Voth, G. A.; Salvador, P.; Dannenberg, J. J.; Dapprich, S.; Daniels, A. D.; Farkas, Ö.; Foresman, J. B.; Ortiz, J. V.; Cioslowski, J.; Fox, D. J. *Gaussian 09, Revision D.01*; Gaussian, Inc., Wallingford, CT, 2009.

[11] *NBO 6.0.* Glendening, E. D.; Badenhoop, J. K.; Reed, A. E.; Carpenter, J. E.; Bohmann, J. A.; Morales, C. M.; Landis, C. R.; Weinhold, F. Theoretical Chemistry Institute, University of Wisconsin, Madison (2013).

[12] AIMAll (Version 13.10.19), Todd A. Keith, TK Gristmill Software, Overland Park KS, USA, 2013 (aim.tkgristmill.com).

[13] Johnson, E. R.; Keinan, S.; Mori-Sánchez, P.; Contreras-García, J.; Cohen, A. J.; Yang, W. *J. Am. Chem. Soc.* 2010, **132**, 6498–6506.

The SDD effective core potential was used for all metals (SDDAll). The split-valence 6-31G* basis set was used for C and H atoms. The basis set for metal hydrides was expanded by adding one extra set of diffuse functions and three sets of p- and one set of d- polarisation functions, i.e. formally [6-31++G(d,3pd)]. The triple-ξ 6-311+G* basis set was used for heteroatoms. This basis set provided very similar results to the 6-31+G(d,p) basis set, but it was much more efficient in terms of computational cost.

The default numerical integration grid was also improved using a pruned grid with 99 radial shells and 590 angular points per shell (int=ultrafine). Dispersion effects were included *via* single point energy corrections and were modelled using the ωB97xD functional for ωB97x, using Grimme's D3 correction for M06L (EmpiricalDispersion=GD3)[14] and Grimme's D3 correction with Becke-Johnson damping (EmpiricalDispersion=GD3BJ) for B3PW91.[15] Solvent effects were not included since the reactions are carried out in rather nonpolar solvents. Furthermore, the computed results matched closely to the experimental ones without need to include solvent corrections.

6.1.1 Functional benchmarking

The DFT functionals employed were in good agreement with the experimental geometries and they have provided good results in previous benchmarking analysis done in the group.[16]

The performance of the three functionals was first inspected analysing the regioselectivity of the C–H activation of toluene (see below, Section 6.2). Although inclusion of dispersion improved the results regardless of the functional, M06L appeared to be more robust. The performance of M06L was next compared to the experimental values obtained from the kinetic analysis. The activation parameters were very close to the experimentally obtained ones in the Eyring analysis, *i.e.* $\Delta H^{\ddagger}_{M06L\text{-}D3}$ = 24.9 kcal·mol^{-1} ($\Delta H^{\ddagger}_{exp}$ = 23.4 (± 1.3) kcal·mol^{-1}) and $\Delta G^{\ddagger}_{M06L\text{-}D3}$ = 26.8 kcal·mol^{-1} ($\Delta G^{\ddagger}_{exp}$ = 25.1 (± 2.6) kcal·mol^{-1}) at 298 K.

The oxidative addition of benzene was also modelled using C_6D_6 as a means to obtain a computational estimate of the KIE. The difference in $\Delta G^{\ddagger}$ allowed to derive an estimated KIE = 5.9. This value is virtually identical to the experimental one (KIE = 5.8 (± 0.1)), which gives additional support to the choice of M06L as the best functional for this system.

---

[14] Grimme, S.; Antony, J.; Ehrlich, S.; Krieg, H. *J Chem. Phys.* 2010, **132**, 154104.
[15] Grimme, S.; Ehrlich, S.; Goerigk, L. *J Comp. Chem.* 2011, **32**, 1456–1465.
[16] a) Butler, M. J.; White, A. J. P.; Crimmin, M. R. *Angew. Chem. Int. Ed.*, 2016, **55**, 6951–6953. b) Hicken, A.; White, A. J. P.; Crimmin, M.R. *Angew. Chem. Int. Ed.* 2017, **56**, 15127–15130.

6.2 Origin of Regioselectivity

The regioselectivity of the C–H activation of toluene was inspected computationally with a series of functionals (ωB97x, M06L and B3PW91). The oxidative addition of the C–H bond to Pd is the rate-determining and regiochemistry determining step.

Int-2 and **Int-2’** stationary points contain a loosely bound toluene molecule. These stationary points are very close in energy ($\Delta\Delta G < 2.1$ kcal·mol^{-1}) and the toluene moiety can undergo facile rotation or dissociation and reassociation. It is a valid assumption that all these regioisomers of the encounter complex **Int-2** and **Int-2’** are in fast equilibrium under the reaction conditions. The activation energy of C–H activation will be determined by the energy difference of the lowest energy encounter complex to the C–H activation transition states. Hence the system can be considered to be under Curtin-Hammett control and the regioselectivity is determined by considering the ΔΔG between possible isomeric transition states of **TS-1** and **TS-1’**.

When dispersion corrections were included into the calculations, a systematic and functional-independent stabilisation of the ortho TSs was observed. A minor stabilisation of the meta TSs compared to the para TSs was also observed (Figure S24). The data for the pathway from **Int-1** are included in the paper while the data from **Int-1’** are provided below. Once the dispersion correction is applied the data are consistent across the series of functionals and predict the correct regiochemistry as observed in experiment for both pathways involving both **TS-1** and **TS-1’** (ortho ~ meta > para).

The weak dispersive interactions are the cause of this systematic stabilisation as well as for the observed selectivity. These interactions are apparent in QTAIM analysis of the regioisomeric transition states (see main text) with bond critical points and bond critical paths for weak dispersion interactions observed between the methyl group of toluene and C–H bonds of the β-position of PCy_3 or the methyl of the *i*-Pr groups on the β-diketiminate ligands.

The non-covalent interactions can be further analysed using the NCIPLOT program. The plots show a network of weakly attractive interactions (green) that can be attributed to dispersion forces. For both ortho transition states, significant attractive interactions are detected between the methyls and the ligand scaffold. Meta and para TSs show practically no interaction between the methyls and the ligand. The rest of interactions are relatively similar for all five transition states (Fig. S26)

Figure S24. (a) Relative free energies (kcal·mol[-1]) for the five regioisomeric TSs of C–H activation of toluene from **Int-2’** using different DFT functionals. **(b)** QTAIM for activation at the ortho position showing dispersive interactions between the methyl group and the ligands. The ligands have been partially truncated for clarity.

(a)

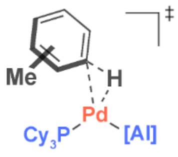

ΔΔG ‡
(kcal mol⁻¹)

persistent stabilisation of TS_{ortho} on inclusion of dispersion correction

	TS_{ortho}	TS_{meta}	TS_{para}
**wB97x**	+0.5 / +1.1	−0.5 / −0.5	0.0
**wB97x-D**	−0.5 / −0.9	−0.6 / −0.9	0.0
**B3PW91**	+4.2 / +5.7	+0.2 / +0.4	0.0
**B3PW91-D3**	**−0.5** / +1.3	0.0 / −0.7	0.0
**M06L**	**−2.5 / −3.3**	−1.4* / −2.9	0.0
**M06L-D3**	**−2.9 / −3.7**	−1.9* / −3.3	0.0

(b)

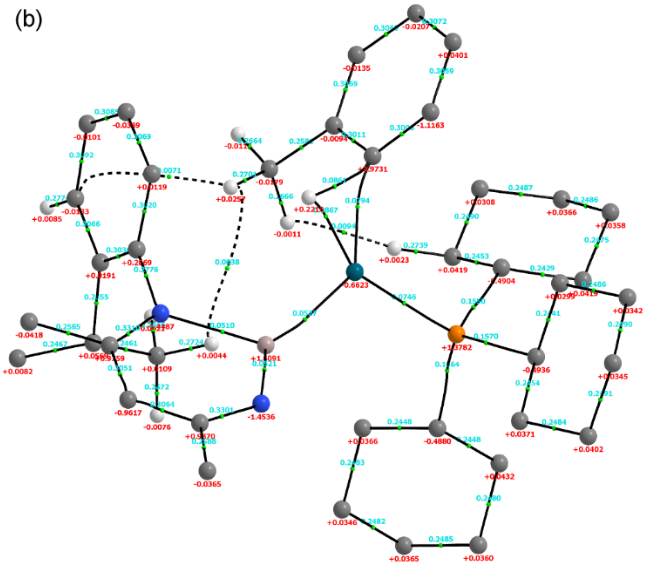

*The TS for oxidative addition was obtained with an additional small, spurious imaginary frequency, which could not be removed.

Figure S25. (a) Relative free energies (kcal·mol[-1]) for the five regioisomeric TSs of C–H activation of toluene from Int-2’ using different DFT functionals. **(b)** QTAIM for activation at the ortho (H^o) position showing dispersive interactions between the methyl group and the ligands. The ligands have been partially truncated for clarity.

(a)

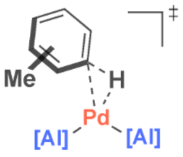

ΔΔG ‡
(kcal mol⁻¹)

persistent stabilisation of TS_{ortho} on inclusion of dispersion correction

	TS_{ortho}	TS_{meta}	TS_{para}
**wB97x**	+1.3 / +2.0	−0.1 / +1.3	0.0
**wB97x-D**	+0.2 / +0.9	−0.2 / +1.2	0.0
**B3PW91**	+3.7 / +4.1	+0.4 / +0.5	0.0
**B3PW91-D3**	−0.6 / +0.9	−0.3 / +0.2	0.0
**M06L**	−1.3 / −1.3	−1.0 / −1.1	0.0
**M06L-D3**	−1.6 / −1.8	−1.1 / −1.2	0.0

(b)

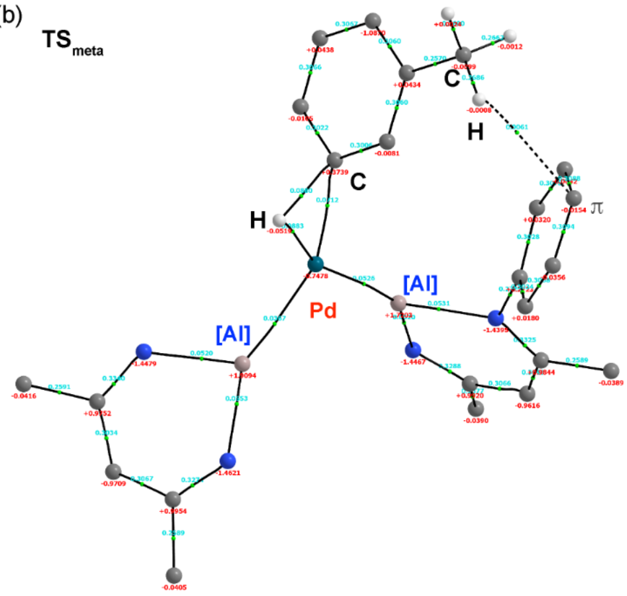

Figure S26. Non-covalent interaction (NCI) plots of the five regioisomeric TSs of C–H activation of toluene.

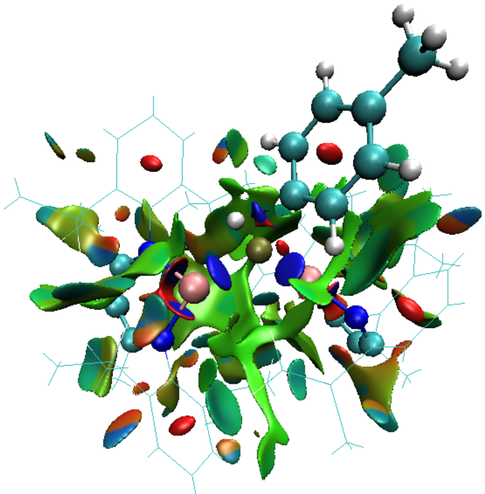

TS$_{para}$

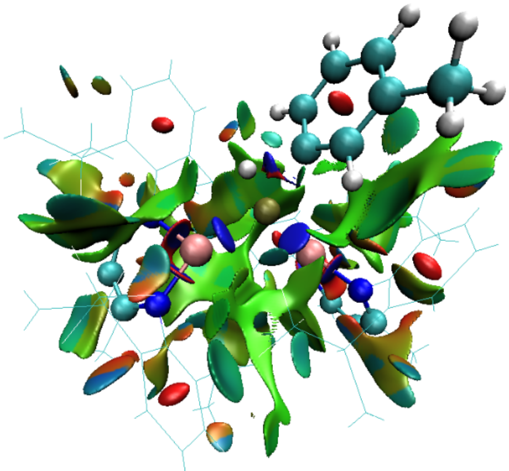

TS$_{meta1}$

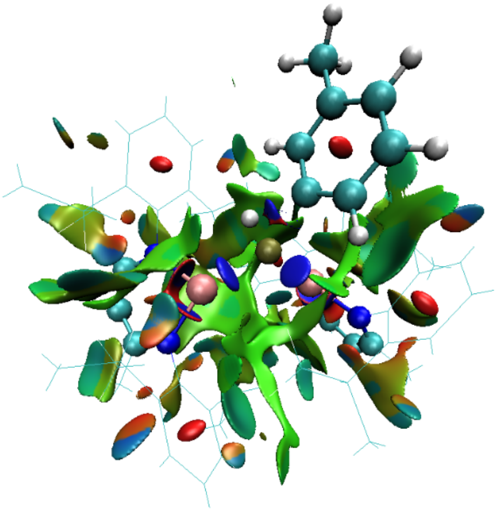

TS$_{meta2}$

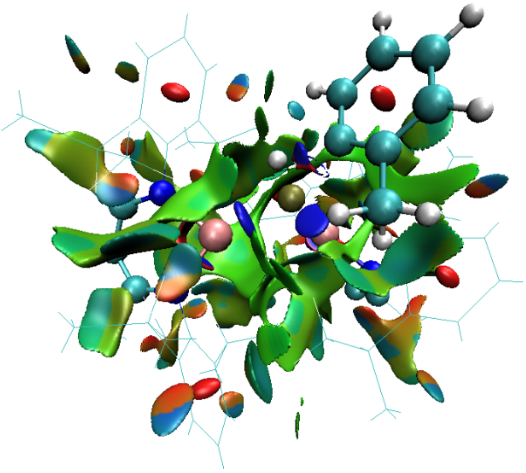

TS$_{ortho1}$

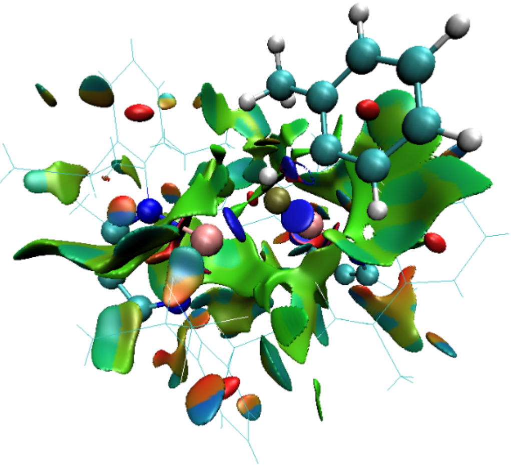

TS$_{ortho2}$

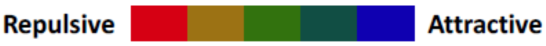

6.3 Mechanism. C_6H_6 activation

*6.3.1 Key geometrical parameters of intermediates, **Int.2** – **Int.5**.*

Figure S27. Selected bond lengths (in Å) for **Int-2**. *i*-Pr groups and some hydrogens have been omitted for clarity.

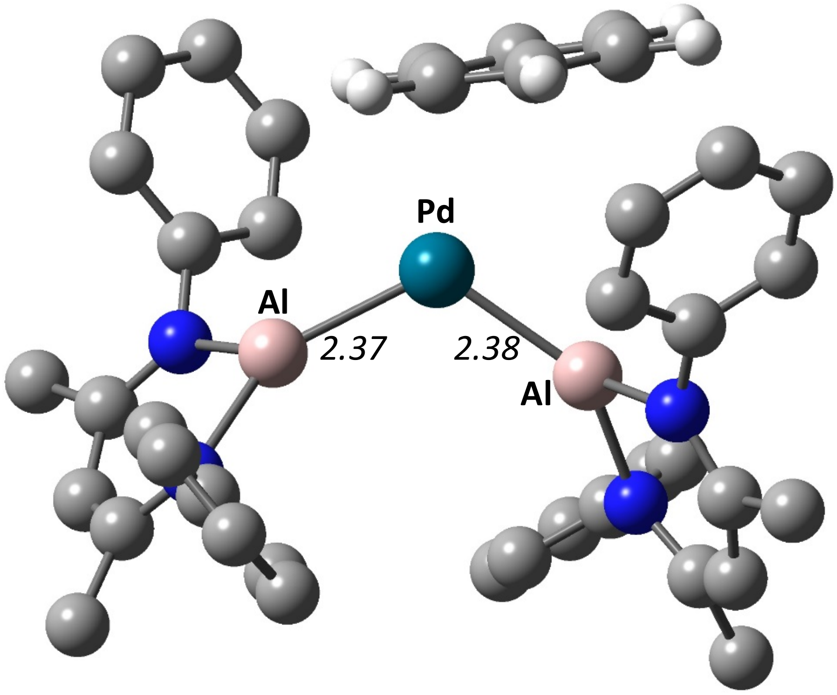

Figure S28. Selected bond lengths (in Å) for **Int-3**. *i*-Pr groups and some hydrogens have been omitted for clarity.

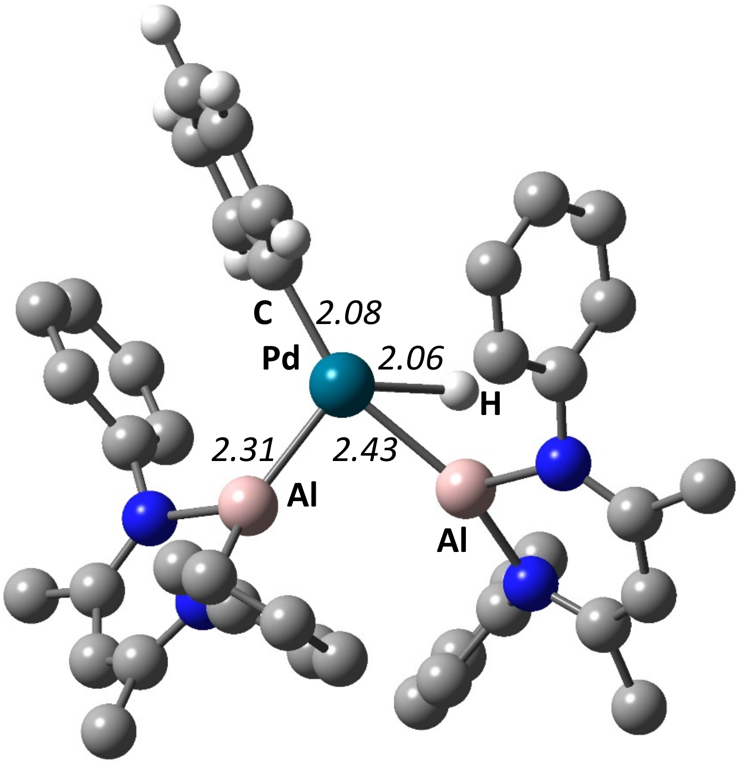

Figure S29. Selected bond lengths (in Å) for **Int-4**. *i*-Pr groups and some hydrogens have been omitted for clarity.

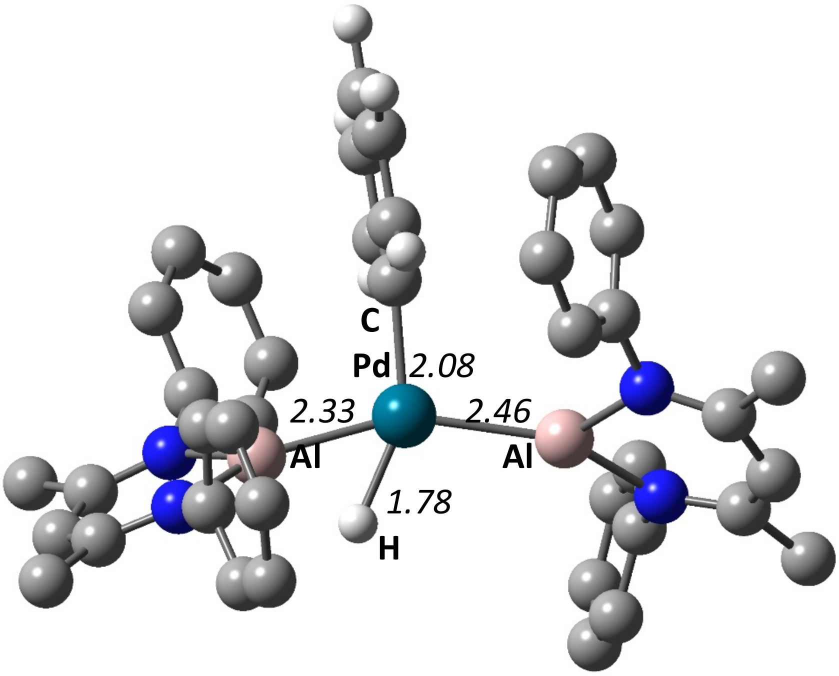

Figure S30. Selected bond lengths (in Å) for **Int-5**. *i*-Pr groups and some hydrogens have been omitted for clarity.

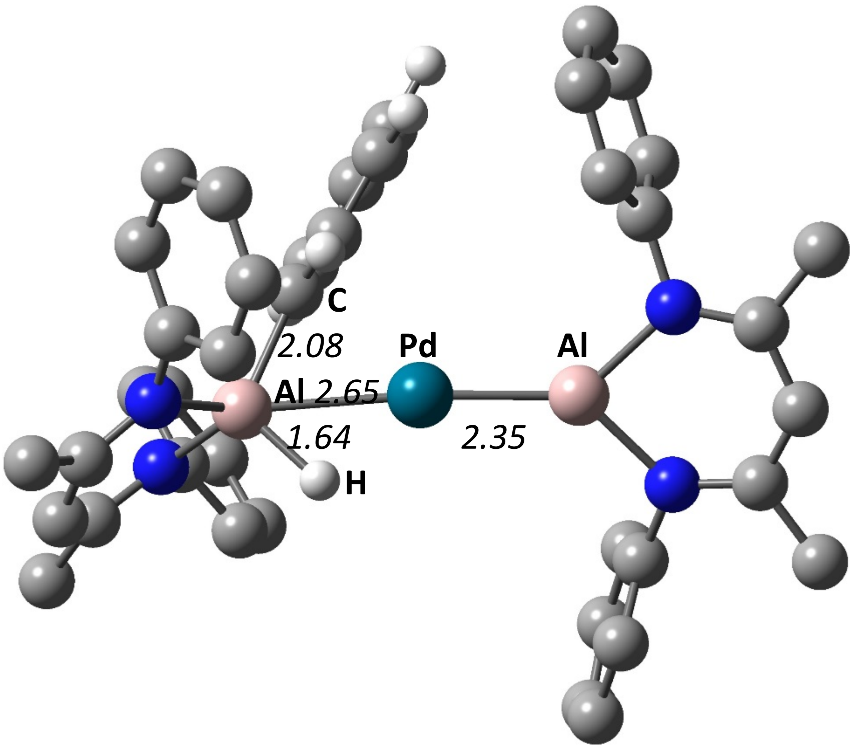

6.3.2 C–H activation pathway from Int-1'

Depending on the PdL_2 fragment that is formed under catalytic conditions, two pathways can be considered. The pathway from **Int-1** is provided in the main text, while the pathway from **Int-1'** is given below. The focus on **Int-1** as the most relevant reactive intermediate is based on the observation that catalysis not only occurs in the absence of the phosphine ligand but also that the ratio of regioisomeric products from the reaction of toluene with **2** is identical from catalytic runs employing either $[Pd(PCy_3)_2]$ or $[Pd(\eta^5\text{-}C_5H_5)(\eta^3\text{-}C_3H_4Ph)]$ as catalyst.

Figure S31. Calculated free energy profile for C–H alumination of benzene from Int-1'.

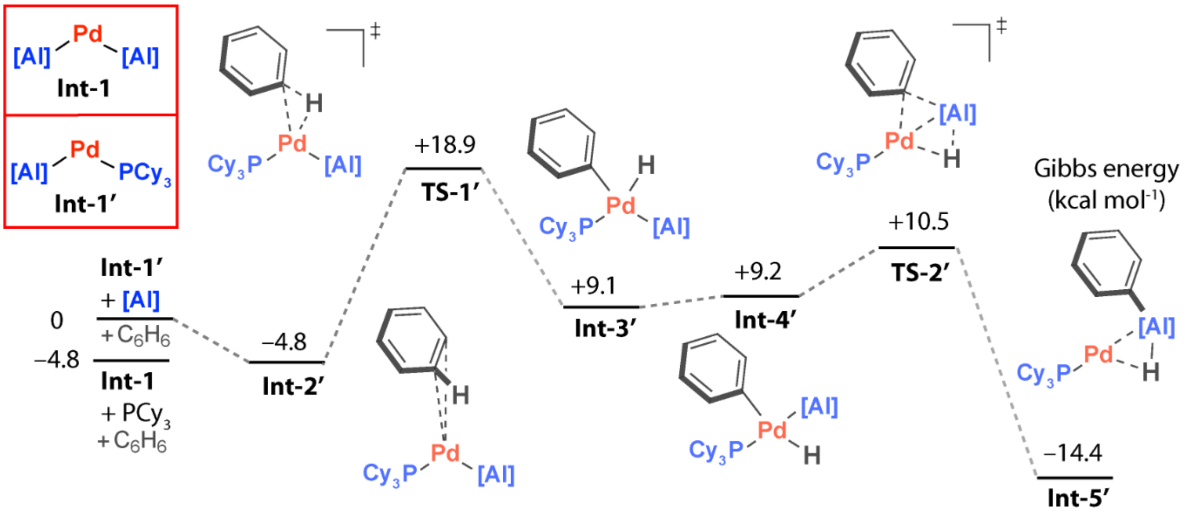

Both pathways, initiated from either **Int-1** or **Int-1'**, have very similar profiles. Both involve a rate-limiting oxidative addition of the C–H bond to palladium. Other mechanistic possibilities were explored. These included *(i)* C–H activation by 1,2-addition of the C–H bond of benzene across the Pd–Al bond of **Int-1** or **Int-1'**, or (*ii*) C–Al and Al–H bond formation from **Int-3** rather than **Int-4**, or (*iii*) oxidative addition of the C–H bond to **Int-1'** to form the diastereomer with the Ph group *trans* to PCy_3, or (*iv*) C–H activation at $Pd(PCy_3)_2$. These calculations did not lead to the identification of viable pathways for C–H alumination and either led to high energy intermediates or did not converge to suitable stationary points.

In order to confirm that the small energy difference between the pathways propagating from **Int-1** or **Int-1'** is not functional dependent, both pathways were also computed at the ωB97x

level. The profiles presented side by side in Figure S31 parallel those found for the M06L functional.

Figure S32. Calculated free energy profile for C–H alumination of benzene from **Int-1** and **Int-1’** at the ωB97x level.

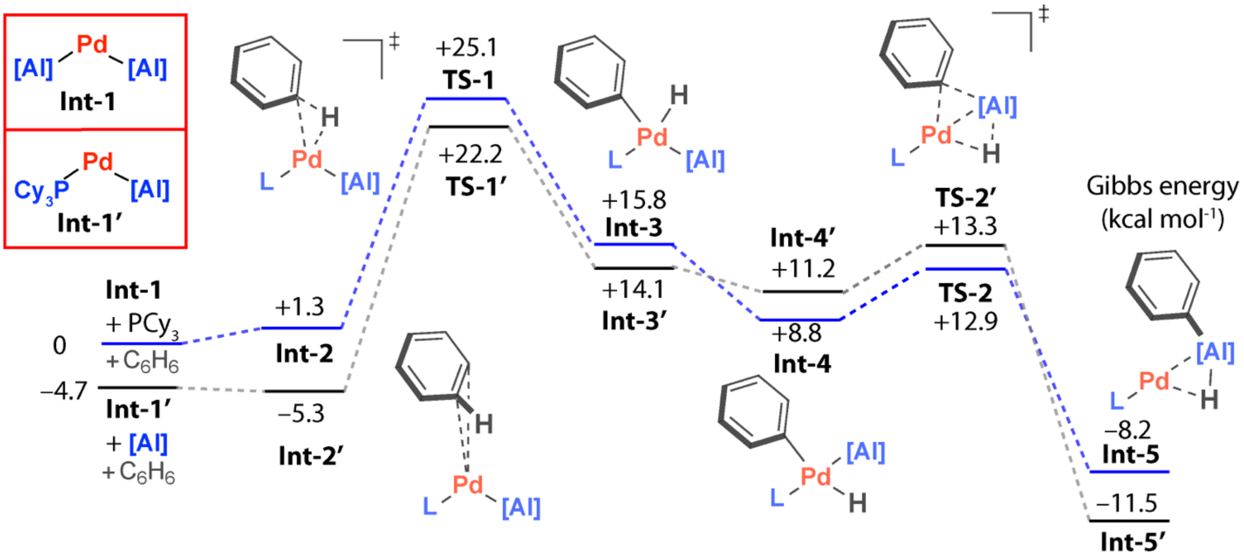

6.4 NBO analysis

The NPA charges and Wiberg Bond Indices (WBI) were inspected (M06L) for pathways from both **Int-1** and **Int-1’**.

Figure S33. Models for **TS1** and **TS2**, showcasing the atoms and bonds relevant to the NBO analysis.

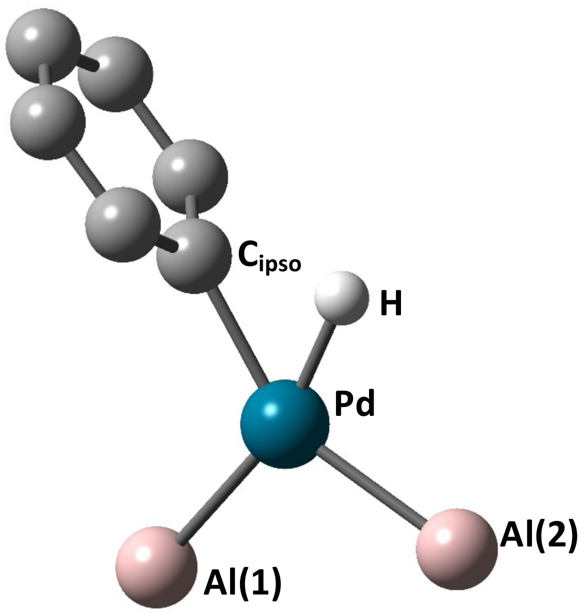

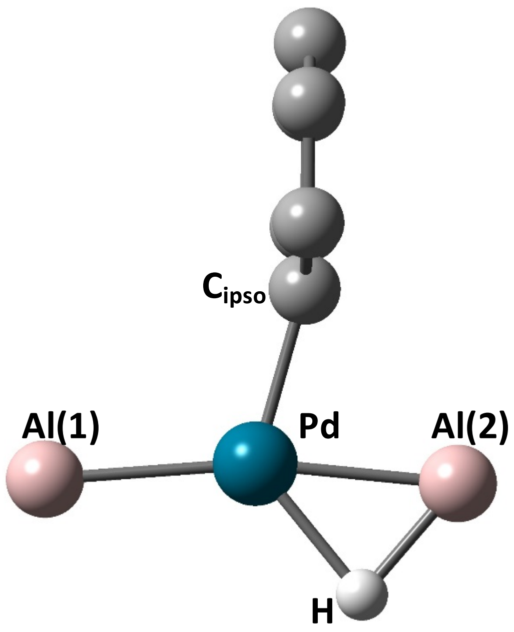

Table S2. Wiberg Bond Indices on stationary points on the pathway from **Int-1**

	Int-2	TS-1	Int-3	Int-4	TS-2	Int-5
Pd-Al(1)	0.54	0.49	0.60	0.44	0.58	0.81
Pd-Al(2)	0.55	0.50	0.33	0.36	0.33	0.11
Pd-H	0.00	0.28	0.08	0.24	0.12	0.08
Pd-C$_{ipso}$	0.08	0.29	0.37	0.42	0.34	0.09
Al(1)-H	0.00	0.16	0.08	0.05	0.04	0.03
Al(1)-C$_{ipso}$	0.01	0.12	0.20	0.09	0.08	0.02
Al(2)-H	0.00	0.15	0.57	0.42	0.55	0.61
Al(2)-C$_{ipso}$	0.02	0.16	0.18	0.23	0.38	0.46
C$_{ipso}$-H	0.90	0.36	0.01	0.12	0.05	0.02

Table S3. *NPA* charges on stationary points on the pathway from **Int-1**

	Pd	H	C$_{ipso}$	Al(2)	Al(1)
Int-2	-0.39	0.24	-0.28	0.99	1.00
TS-1	-0.27	-0.02	-0.26	1.14	1.11
Int-3	-0.08	-0.47	-0.30	1.22	1.22
Int-4	-0.19	-0.38	-0.25	1.35	1.19
TS-2	-0.21	-0.45	-0.32	1.47	1.13
Int-5	-0.30	-0.45	-0.70	1.78	1.12

Figure S34. Models for **TS1'** and **TS2'**, showcasing the atoms and bonds relevant to the NBO analysis.

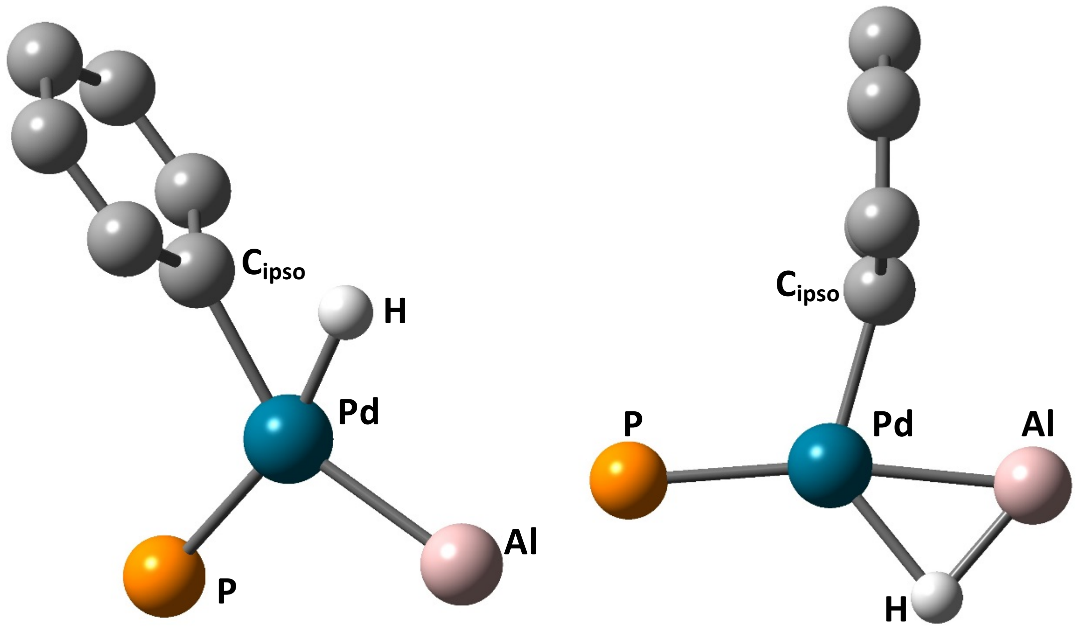

Table S4. Wiberg Bond Indices on stationary points on the pathway from **Int-1'**

	Int-2'	TS-1'	Int-3'	Int-4'	TS-2'	Int-5'
Pd-P	0.34	0.29	0.42	0.26	0.31	0.47
Pd-Al	0.70	0.60	0.47	0.53	0.44	0.32
Pd-H	0.00	0.35	0.31	0.24	0.15	0.25
Pd-C_{ipso}	0.05	0.28	0.30	0.45	0.39	0.02
Al-H	0.00	0.11	0.36	0.42	0.53	0.48
Al-C_{ipso}	0.01	0.18	0.30	0.19	0.36	0.45
C_{ipso}-H	0.91	0.40	0.05	0.14	0.07	0.02

Table S5. *NPA* charges on stationary points on the pathway from **Int-1'**

	Pd	H	C_{ipso}	Al	P
Int-2'	-0.29	0.24	-0.25	1.05	0.90
TS-1'	-0.09	0.01	-0.28	1.13	0.96
Int-3'	0.08	-0.33	-0.31	1.19	1.10
Int-4'	0.05	-0.38	-0.25	1.30	0.99
TS-2'	0.04	-0.44	-0.30	1.47	0.91
Int-5'	-0.18	-0.37	-0.64	1.73	0.99

6.5 Additional QTAIM analysis

Figure S35. QTAIM contour plot of ρ for **TS-1**. Charges are depicted in red and the Laplacian of ρ ($\nabla^2\rho$) in blue.

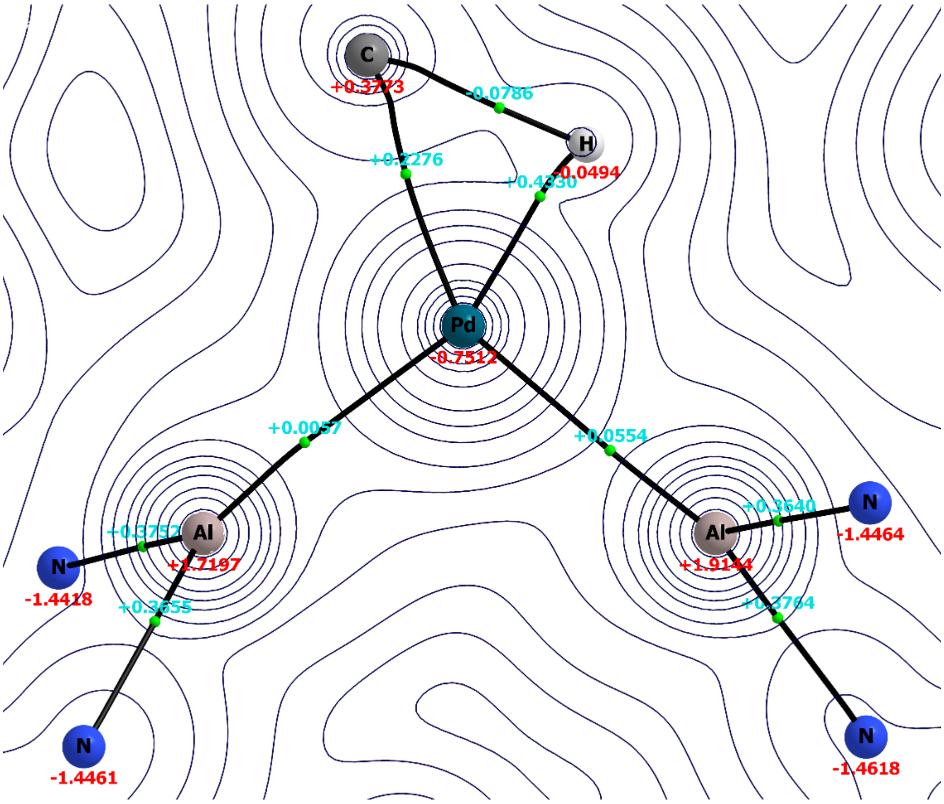

Figure S36. QTAIM contour plot of ρ for **TS-2**. Charges are depicted in red and the Laplacian of ρ ($\nabla^2\rho$) in blue.

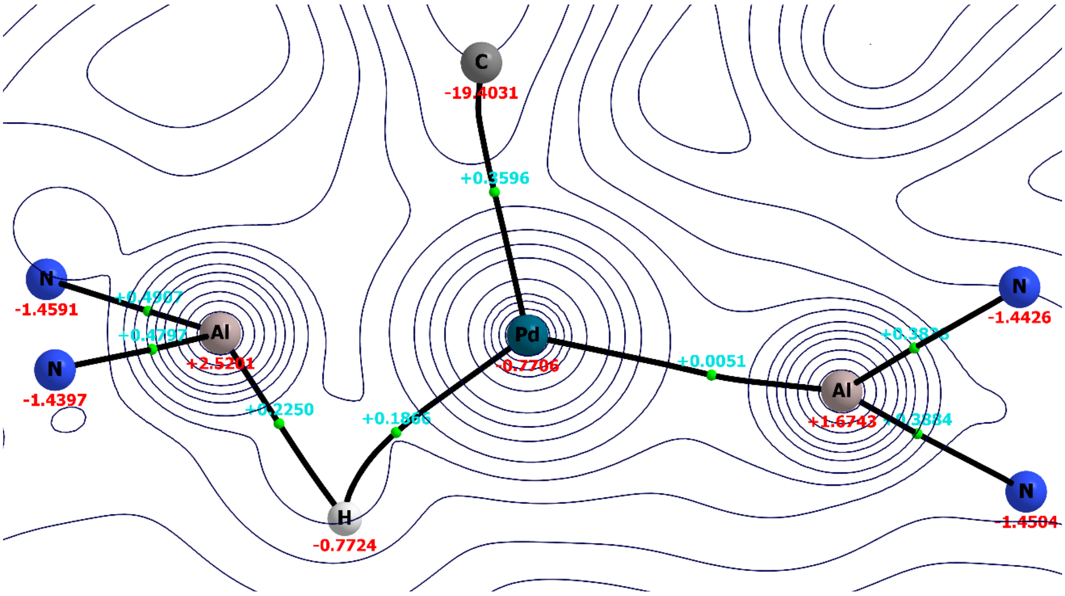

6.6 XYZ Coordinates

DippAlH2.log

SCF (wB97x) = -1242.43254984
H(0 K)= -1241.778088
H(298 K)= -1241.742111
G(298 K)= -1241.844421
Lowest Frequency = 20.2274cm-1

N	1.412495	0.386717	0.413152
C	1.237907	0.519981	1.728773
Al	-0.009028	0.640270	-0.882598
H	0.057540	-0.475642	-1.986261
H	-0.078394	2.155160	-1.306157
N	-1.423631	0.261718	0.397240
C	-0.027443	0.633783	2.324709
H	-0.039839	0.775418	3.401147
C	-1.275694	0.429692	1.712868
C	2.439404	0.510719	2.626902
H	3.154028	1.289562	2.331103
H	2.981439	-0.439994	2.546662
H	2.161719	0.665137	3.672438
C	-2.482907	0.377488	2.603553
H	-3.112857	1.263511	2.450821
H	-2.199553	0.339831	3.658485
H	-3.113390	-0.489094	2.372605
C	2.706609	0.048967	-0.100601
C	3.587871	1.064561	-0.517476
C	4.818338	0.689170	-1.063262
H	5.509577	1.465570	-1.392856
C	5.171214	-0.647327	-1.195424
H	6.135327	-0.919182	-1.621795
C	4.284510	-1.636164	-0.787491
H	4.557577	-2.686221	-0.898827
C	3.041072	-1.313233	-0.240721
C	2.096906	-2.424250	0.176906
H	1.180545	-1.964379	0.574580
C	1.695810	-3.286514	-1.018567
H	1.193988	-2.690779	-1.790859
H	1.011872	-4.086029	-0.704435
H	2.571276	-3.765752	-1.476756
C	2.702477	-3.288585	1.282262
H	2.979561	-2.696123	2.163084
H	3.608883	-3.802035	0.934785
H	1.992187	-4.059139	1.607452
C	3.233076	2.534429	-0.412760
H	2.267023	2.616919	0.104623
C	4.269860	3.314583	0.394121
H	4.415485	2.893697	1.396930
H	3.963368	4.361270	0.509953
H	5.248235	3.314216	-0.104087
C	3.066091	3.150925	-1.801435
H	2.300835	2.627328	-2.385664
H	4.007637	3.108962	-2.365683
H	2.767216	4.203799	-1.726975
C	-2.712073	-0.060722	-0.141753
C	-3.663587	0.953664	-0.364152
C	-4.895773	0.592627	-0.916898

H	-5.641384	1.367026	-1.098662
C	-5.178811	-0.724536	-1.246440
H	-6.145714	-0.986315	-1.672889
C	-4.216245	-1.708211	-1.048635
H	-4.438308	-2.735253	-1.331808
C	-2.967051	-1.401523	-0.505870
C	-1.916264	-2.478191	-0.301204
H	-0.937156	-2.018046	-0.505433
C	-1.886432	-2.979283	1.144023
H	-1.150630	-3.787041	1.256446
H	-2.865425	-3.377495	1.444121
H	-1.611233	-2.187085	1.850463
C	-2.070680	-3.649765	-1.263415
H	-1.216436	-4.330353	-1.172058
H	-2.122071	-3.313507	-2.305876
H	-2.971576	-4.241121	-1.053238
C	-3.379961	2.416573	-0.082054
H	-2.429339	2.487256	0.465072
C	-4.469599	3.066475	0.769835
H	-4.655944	2.517538	1.701416
H	-5.423430	3.117943	0.229034
H	-4.191865	4.094189	1.033054
C	-3.210821	3.187453	-1.392218
H	-2.403429	2.770026	-2.003796
H	-2.973181	4.240574	-1.196553
H	-4.135665	3.156395	-1.984021

DippAlHPh.log

SCF (wB97x) = -1473.46044615
H(0 K)= -1472.721953
H(298 K)= -1472.681329
G(298 K)= -1472.793932
Lowest Frequency = 18.6537cm-1

N	-1.405211	-0.400146	0.576110
C	-1.241156	-0.522283	1.896516
Al	0.015598	0.171664	-0.625495
H	0.087494	-0.710061	-1.921140
N	1.445048	-0.264531	0.610427
C	0.009562	-0.444934	2.531575
H	0.000186	-0.539165	3.613192
C	1.273956	-0.410479	1.925739
C	-2.432702	-0.788051	2.769461
H	-3.074759	-1.568436	2.345127
H	-3.055455	0.110651	2.855634
H	-2.128592	-1.086259	3.776033
C	2.476297	-0.577011	2.807004
H	3.111046	-1.402481	2.463250
H	2.191253	-0.760632	3.845832
H	3.104752	0.322192	2.772598
C	-2.707121	-0.612692	0.003776
C	-2.960015	-1.848953	-0.634995
C	-4.220194	-2.054453	-1.199587
H	-4.437008	-3.000175	-1.691835
C	-5.202496	-1.072302	-1.152693
H	-6.178083	-1.254100	-1.600411
C	-4.927873	0.143129	-0.546233
H	-5.690516	0.922061	-0.527581

C	-3.684899	0.402690	0.039582
C	-3.447807	1.772657	0.646186
H	-2.454624	1.778984	1.118426
C	-3.451959	2.850546	-0.438629
H	-2.710262	2.648350	-1.218052
H	-3.221933	3.833623	-0.009494
H	-4.439486	2.914106	-0.915960
C	-4.492534	2.123250	1.707440
H	-4.582048	1.359340	2.489317
H	-5.487545	2.239531	1.258274
H	-4.243507	3.075186	2.191857
C	-1.903101	-2.938253	-0.688102
H	-0.931972	-2.444018	-0.838382
C	-1.816142	-3.716171	0.626483
H	-1.503839	-3.081917	1.464675
H	-1.083070	-4.529591	0.542529
H	-2.784703	-4.166791	0.883221
C	-2.094641	-3.899828	-1.855105
H	-2.191087	-3.366943	-2.808557
H	-2.983182	-4.532224	-1.728719
H	-1.234584	-4.574647	-1.933592
C	2.736510	-0.510355	0.032841
C	3.001447	-1.807638	-0.455131
C	4.239028	-2.052044	-1.052760
H	4.460040	-3.053378	-1.423649
C	5.188205	-1.045747	-1.180415
H	6.146802	-1.253943	-1.652631
C	4.906229	0.227101	-0.704100
H	5.648134	1.019346	-0.808868
C	3.685927	0.522715	-0.089476
C	3.432287	1.933246	0.403138
H	2.452794	1.950753	0.902199
C	4.491679	2.384924	1.408808
H	4.245531	3.376150	1.808719
H	5.480568	2.460448	0.937636
H	4.590380	1.694130	2.255529
C	3.373311	2.918474	-0.763718
H	3.128014	3.927011	-0.408292
H	2.611436	2.633356	-1.497083
H	4.341553	2.969021	-1.280571
C	2.005694	-2.943549	-0.313726
H	1.065563	-2.529661	0.077781
C	2.503038	-3.982248	0.692099
H	2.674544	-3.542870	1.682678
H	3.449874	-4.430449	0.362060
H	1.774703	-4.794629	0.809295
C	1.691050	-3.598260	-1.655856
H	1.262833	-2.875365	-2.360749
H	0.968089	-4.413705	-1.522720
H	2.587796	-4.032473	-2.116977
C	-0.055935	2.133294	-0.857039
C	-0.114426	2.755431	-2.115867
C	-0.038565	2.986457	0.261827
C	-0.155575	4.142366	-2.256994
H	-0.127568	2.138762	-3.018078
C	-0.078449	4.374106	0.139920
H	0.008101	2.552746	1.267032
C	-0.137873	4.955166	-1.125959
H	-0.200781	4.592304	-3.248641

H	-0.063631	5.005906	1.028360
H	-0.169435	6.039249	-1.229645

DippAl.log

SCF (wB97x) = -1241.19058768
H(0 K)= -1240.551547
H(298 K)= -1240.516158
G(298 K)= -1240.618535
Lowest Frequency = 10.0608cm-1

Al	-0.000004	0.000281	-1.006713
N	-1.401832	0.026299	0.454094
N	1.401854	-0.025994	0.454108
C	-1.251376	0.060418	1.782047
C	0.000012	0.000296	2.405979
H	0.000014	0.000375	3.492140
C	1.251402	-0.059940	1.782056
C	-2.464311	0.156365	2.663770
H	-3.082208	1.019471	2.386652
H	-2.188189	0.245390	3.717940
H	-3.109492	-0.724248	2.548205
C	2.464340	-0.155895	2.663775
H	3.109608	0.724634	2.548112
H	3.082144	-1.019099	2.386729
H	2.188229	-0.244793	3.717959
C	-2.733378	0.055535	-0.078105
C	-3.268136	1.274171	-0.535936
C	-4.534866	1.259954	-1.127410
H	-4.960888	2.196563	-1.488828
C	-5.252630	0.080608	-1.263252
H	-6.236300	0.089179	-1.729585
C	-4.711090	-1.113990	-0.800797
H	-5.278901	-2.037129	-0.910117
C	-3.451138	-1.152697	-0.202617
C	-2.528244	2.591534	-0.399647
H	-1.574290	2.398214	0.113698
C	-2.203762	3.188813	-1.767939
H	-1.598376	2.498608	-2.369550
H	-1.647223	4.128846	-1.661961
H	-3.120110	3.407296	-2.332400
C	-3.315311	3.588039	0.450697
H	-3.542562	3.188199	1.446766
H	-4.270943	3.848813	-0.022847
H	-2.750007	4.518868	0.582474
C	-2.852121	-2.469380	0.256831
H	-2.116954	-2.250743	1.045183
C	-3.882974	-3.424597	0.849969
H	-4.478283	-2.948413	1.639033
H	-3.386087	-4.301006	1.282658
H	-4.580678	-3.797987	0.089731
C	-2.094308	-3.141438	-0.888733
H	-1.319453	-2.479615	-1.304487
H	-2.776636	-3.394288	-1.710984
H	-1.607758	-4.066566	-0.552848
C	2.733361	-0.055530	-0.078142
C	3.267796	-1.274308	-0.535978
C	4.534484	-1.260415	-1.127551
H	4.960252	-2.197141	-1.488966

C	5.252519	-0.081247	-1.263483
H	6.236149	-0.090062	-1.729897
C	4.711304	1.113493	-0.801010
H	5.279331	2.036491	-0.910406
C	3.451410	1.152525	-0.202734
C	2.527627	-2.591506	-0.399523
H	1.573685	-2.397909	0.113741
C	2.203088	-3.188981	-1.767707
H	1.597872	-2.498768	-2.369480
H	1.646342	-4.128873	-1.661567
H	3.119402	-3.407784	-2.332099
C	3.314474	-3.587983	0.451061
H	3.541699	-3.187992	1.447076
H	4.270111	-3.848963	-0.022363
H	2.749033	-4.518711	0.582964
C	2.852742	2.469367	0.256693
H	2.117488	2.250947	1.045022
C	3.883844	3.424297	0.849875
H	4.479002	2.947935	1.638947
H	3.387183	4.300834	1.282564
H	4.581670	3.797506	0.089661
C	2.095154	3.141639	-0.888892
H	1.320096	2.480052	-1.304643
H	2.777568	3.394268	-1.711140
H	1.608891	4.066926	-0.553025

Int-1a_PdAl2_noP.log

SCF (wB97x) = -2610.48002348
H(0 K)= -2609.198428
H(298 K)= -2609.125161
G(298 K)= -2609.306295
Lowest Frequency = 12.2527cm-1

Pd	0.014195	-0.036338	-1.390243
Al	2.146010	-0.185077	-0.349351
N	3.420571	1.330505	-0.209843
N	2.869832	-0.976239	1.323809
C	4.126326	1.693543	0.862991
C	4.204298	0.904222	2.020894
H	4.816392	1.295580	2.828160
C	3.689289	-0.383776	2.202377
C	4.917275	2.969753	0.843937
H	5.566420	3.019493	-0.038928
H	5.533066	3.070993	1.741753
H	4.251823	3.840292	0.782754
C	4.108559	-1.124603	3.439344
H	3.242216	-1.363502	4.069166
H	4.815671	-0.540449	4.034016
H	4.572652	-2.085749	3.186103
C	3.539268	2.112408	-1.412882
C	4.349368	1.606009	-2.454287
C	4.472762	2.362224	-3.621531
H	5.095021	1.994547	-4.434660
C	3.802779	3.570472	-3.773425
H	3.912381	4.142966	-4.693020
C	2.977328	4.029637	-2.758376
H	2.424490	4.959809	-2.890472
C	2.824171	3.315510	-1.567081

C	5.063221	0.272934	-2.314393
H	4.360821	-0.414913	-1.808183
C	5.409186	-0.368027	-3.652255
H	4.531329	-0.446723	-4.304294
H	5.805206	-1.378333	-3.495618
H	6.183514	0.194988	-4.189507
C	6.313611	0.369222	-1.439232
H	7.026924	1.092806	-1.857150
H	6.821011	-0.603333	-1.384402
H	6.082861	0.675567	-0.412466
C	1.847878	3.817779	-0.525121
H	2.005564	3.235243	0.390869
C	0.410817	3.569175	-0.984770
H	-0.306420	3.891694	-0.218065
H	0.230143	2.500054	-1.187621
H	0.194696	4.127972	-1.906676
C	2.044791	5.292000	-0.177602
H	3.075169	5.520974	0.125602
H	1.376658	5.578200	0.645374
H	1.803363	5.943843	-1.027272
C	2.560733	-2.367433	1.511964
C	1.510293	-2.773371	2.360093
C	1.231725	-4.138638	2.463898
H	0.416677	-4.461621	3.112672
C	1.960417	-5.086750	1.758105
H	1.720526	-6.144602	1.852154
C	2.993248	-4.672039	0.929381
H	3.567819	-5.411472	0.370785
C	3.312502	-3.319001	0.789694
C	0.664928	-1.799842	3.158462
H	1.110660	-0.799827	3.062507
C	-0.756747	-1.730503	2.601352
H	-1.205930	-2.731521	2.531309
H	-0.762385	-1.290061	1.592509
H	-1.408517	-1.125272	3.245881
C	0.623787	-2.159672	4.644224
H	0.084993	-3.100593	4.814878
H	0.100424	-1.381124	5.213415
H	1.625873	-2.278171	5.075230
C	4.457748	-2.933762	-0.127755
H	4.596125	-1.843700	-0.071642
C	4.136422	-3.288410	-1.579164
H	3.954459	-4.365530	-1.694347
H	4.971426	-3.020131	-2.239523
H	3.239558	-2.759705	-1.931574
C	5.770888	-3.578786	0.313683
H	6.030580	-3.309934	1.345019
H	6.596646	-3.261075	-0.335035
H	5.718098	-4.674200	0.262148
Al	-2.130137	0.166548	-0.373481
N	-3.363820	-1.369261	-0.120430
N	-2.870219	1.056376	1.239849
C	-4.136069	-1.630895	0.933241
C	-4.195873	-0.787474	2.053121
H	-4.821083	-1.123813	2.875308
C	-3.661732	0.500272	2.169113
C	-5.052226	-2.821246	0.923812
H	-5.961639	-2.590572	0.351704
H	-5.361179	-3.089043	1.938645

H	-4.597484	-3.691793	0.440545
C	-4.039749	1.286203	3.392722
H	-3.162116	1.475371	4.023672
H	-4.781395	0.752299	3.992806
H	-4.443064	2.271171	3.127397
C	-3.508988	-2.175433	-1.301366
C	-4.585638	-1.917627	-2.173264
C	-4.699326	-2.686685	-3.333317
H	-5.524565	-2.494532	-4.019611
C	-3.772061	-3.675691	-3.632491
H	-3.877242	-4.268114	-4.539949
C	-2.699726	-3.894833	-2.776926
H	-1.963892	-4.659318	-3.022817
C	-2.536746	-3.149566	-1.606328
C	-5.566721	-0.784305	-1.937911
H	-5.415923	-0.392416	-0.921138
C	-5.281378	0.357391	-2.915039
H	-5.429309	0.028391	-3.952294
H	-4.244918	0.712630	-2.829091
H	-5.954110	1.206959	-2.734508
C	-7.023681	-1.227081	-2.049982
H	-7.698653	-0.400816	-1.793234
H	-7.252912	-2.068767	-1.384476
H	-7.272344	-1.543020	-3.071355
C	-1.373712	-3.431433	-0.676663
H	-1.183083	-2.516786	-0.097212
C	-1.726594	-4.548944	0.305290
H	-2.572640	-4.277686	0.950271
H	-0.870184	-4.774906	0.953386
H	-1.997073	-5.470865	-0.228760
C	-0.074253	-3.744867	-1.409390
H	0.753002	-3.799278	-0.688669
H	0.171009	-2.954335	-2.131215
H	-0.111176	-4.707837	-1.938293
C	-2.557992	2.453853	1.368586
C	-1.552875	2.901404	2.247729
C	-1.324699	4.276229	2.354678
H	-0.547161	4.631664	3.031948
C	-2.052559	5.189307	1.605391
H	-1.861870	6.256815	1.704090
C	-3.007887	4.730723	0.706203
H	-3.555588	5.448392	0.098304
C	-3.273865	3.367253	0.562035
C	-0.659895	1.949821	3.016910
H	-1.075586	0.938418	2.921094
C	0.731943	1.939340	2.385608
H	1.177675	2.944599	2.413317
H	0.681655	1.619825	1.332739
H	1.414839	1.266299	2.921547
C	-0.556611	2.286353	4.503102
H	0.027283	1.519957	5.028861
H	-1.537703	2.355441	4.989469
H	-0.046849	3.245056	4.664787
C	-4.323917	2.889130	-0.424198
H	-3.989791	1.910886	-0.811897
C	-4.467222	3.805251	-1.632683
H	-4.921008	4.769746	-1.370199
H	-5.115941	3.341698	-2.385004
H	-3.495086	4.002727	-2.101252

C	-5.675002	2.658765	0.253165
H	-6.048562	3.586105	0.708784
H	-5.614425	1.896169	1.039578
H	-6.422675	2.319628	-0.476847

Int-1b_PPdAl.log

SCF (wB97x) = -2416.32214567
H(0 K)= -2415.195245
H(298 K)= -2415.136805
G(298 K)= -2415.291314
Lowest Frequency = 8.9592cm-1

Pd	0.555083	-0.336616	-0.870263
P	2.789635	-0.367893	-0.239042
C	3.527944	1.342079	-0.206795
H	4.586142	1.275272	0.102594
C	2.805796	2.268314	0.775061
H	1.729955	2.286016	0.521740
H	2.876062	1.880303	1.802344
C	3.375867	3.681631	0.727902
H	2.837810	4.323889	1.438772
H	4.426914	3.661553	1.062113
C	3.307277	4.261344	-0.678444
H	3.733236	5.274169	-0.703042
H	2.248289	4.357268	-0.969611
C	4.019536	3.353814	-1.673530
H	5.097581	3.333107	-1.440668
H	3.932267	3.753256	-2.693553
C	3.465572	1.934679	-1.620224
H	4.001557	1.292553	-2.335091
H	2.408479	1.932927	-1.940938
C	4.034563	-1.303882	-1.283557
H	3.723801	-1.035412	-2.309500
C	5.520294	-0.950071	-1.161239
H	5.881605	-1.189688	-0.149558
H	5.679345	0.128320	-1.297904
C	6.347232	-1.719904	-2.189357
H	6.052306	-1.391942	-3.199851
H	7.411579	-1.468957	-2.083003
C	6.136170	-3.224714	-2.072263
H	6.517715	-3.567214	-1.096116
H	6.720956	-3.755942	-2.835354
C	4.658449	-3.580196	-2.180861
H	4.510774	-4.662048	-2.060571
H	4.295138	-3.327502	-3.190046
C	3.835952	-2.816662	-1.149256
H	2.768092	-3.068230	-1.230951
H	4.154677	-3.131348	-0.141521
C	2.968559	-0.927583	1.528879
H	2.435807	-0.114687	2.053834
C	2.168735	-2.198466	1.836064
H	1.168141	-2.117985	1.383595
H	2.650739	-3.070255	1.367319
C	2.082595	-2.425259	3.341230
H	1.524702	-3.346757	3.558367
H	1.506363	-1.600735	3.794586
C	3.468754	-2.480584	3.975608
H	3.392522	-2.610707	5.063757

H	4.001802	-3.367621	3.595344
C	4.283379	-1.234808	3.644179
H	5.289325	-1.303595	4.080712
H	3.804078	-0.354594	4.105083
C	4.369332	-1.012189	2.135878
H	4.915654	-1.853539	1.677724
H	4.952060	-0.105412	1.914602
Al	-1.765588	0.128060	-0.599705
N	-2.579982	1.806516	0.018753
N	-3.458221	-0.820740	-0.317778
C	-3.884217	2.074412	0.145885
C	-4.874045	1.091058	0.012664
H	-5.903300	1.420859	0.118340
C	-4.669576	-0.286802	-0.131740
C	-4.328792	3.475074	0.454948
H	-3.840860	3.858232	1.359694
H	-5.412426	3.529249	0.588855
H	-4.043907	4.159926	-0.354626
C	-5.864959	-1.188564	-0.041176
H	-5.904083	-1.881683	-0.890389
H	-6.796272	-0.617288	-0.003883
H	-5.809209	-1.816395	0.857796
C	-1.639504	2.839744	0.352074
C	-1.046258	2.809991	1.631591
C	-0.191336	3.856806	1.983739
H	0.271608	3.861801	2.969753
C	0.101064	4.878991	1.088332
H	0.767306	5.689271	1.381585
C	-0.424333	4.841633	-0.196129
H	-0.148077	5.614949	-0.913148
C	-1.293604	3.822670	-0.592448
C	-1.321601	1.672170	2.597431
H	-1.522261	0.773415	1.990787
C	-0.117670	1.338143	3.469714
H	0.773071	1.154189	2.853347
H	-0.313513	0.433182	4.057542
H	0.118745	2.138599	4.182833
C	-2.566343	1.927081	3.447004
H	-2.457155	2.845024	4.040724
H	-2.739357	1.096368	4.144536
H	-3.467929	2.030541	2.830064
C	-1.759761	3.747535	-2.031524
H	-2.587625	3.025819	-2.086641
C	-0.619126	3.210002	-2.900477
H	-0.941213	3.084659	-3.942332
H	-0.257802	2.238562	-2.529869
H	0.232432	3.905737	-2.892484
C	-2.270939	5.081444	-2.568543
H	-3.065973	5.503247	-1.940063
H	-2.671771	4.960238	-3.582008
H	-1.469739	5.829148	-2.627612
C	-3.320912	-2.251048	-0.304353
C	-3.292737	-2.964050	-1.517109
C	-3.088896	-4.345401	-1.466166
H	-3.061831	-4.910387	-2.398339
C	-2.912275	-5.004881	-0.257399
H	-2.753182	-6.081712	-0.239438
C	-2.930004	-4.282210	0.929269
H	-2.773980	-4.798264	1.876778

C	-3.130290	-2.900486	0.931555
C	-3.454081	-2.287184	-2.863287
H	-3.654183	-1.220535	-2.686939
C	-2.165624	-2.389957	-3.679840
H	-1.922904	-3.438781	-3.897868
H	-1.314896	-1.952939	-3.139812
H	-2.269491	-1.866182	-4.638653
C	-4.637208	-2.856616	-3.644905
H	-4.482816	-3.916147	-3.887703
H	-4.770258	-2.320837	-4.592751
H	-5.576528	-2.783935	-3.082274
C	-3.072293	-2.136061	2.240630
H	-3.432763	-1.112089	2.061983
C	-1.624169	-2.035956	2.722844
H	-1.199742	-3.035076	2.891816
H	-1.561192	-1.481447	3.669187
H	-0.983544	-1.530534	1.983327
C	-3.958110	-2.751015	3.321406
H	-4.998315	-2.857616	2.989335
H	-3.954387	-2.125563	4.222816
H	-3.604884	-3.746971	3.618036

Int-2a_cc-PdAl2.log

SCF (wB97x) = -2842.72193682
H(0 K)= -2841.338283
H(298 K)= -2841.259450
G(298 K)= -2841.450825
Lowest Frequency = 12.1959cm-1

Pd	-0.014814	-0.094533	-1.213913
Al	1.996596	-0.102594	0.064597
N	3.202507	1.485748	0.202779
N	2.685813	-0.790961	1.799894
C	3.936741	1.864035	1.252312
C	4.049841	1.094672	2.418837
H	4.702192	1.490716	3.192279
C	3.561562	-0.201504	2.623959
C	4.777066	3.109023	1.178423
H	5.710119	2.907821	0.633880
H	5.048414	3.459906	2.178630
H	4.273479	3.915483	0.635915
C	4.140592	-0.963531	3.785528
H	3.402407	-1.581782	4.305304
H	4.611988	-0.286452	4.503865
H	4.914600	-1.654274	3.422784
C	3.374481	2.199686	-1.032294
C	4.519205	1.930432	-1.813814
C	4.671566	2.607091	-3.025429
H	5.546177	2.405116	-3.643327
C	3.727123	3.531017	-3.456238
H	3.867098	4.057094	-4.399393
C	2.601364	3.773888	-2.681139
H	1.857417	4.491421	-3.025802
C	2.389817	3.107269	-1.470414
C	5.535087	0.879655	-1.403267
H	5.465129	0.730014	-0.316562
C	5.193629	-0.458945	-2.058068
H	4.160772	-0.766782	-1.831656

H	5.873926	-1.250223	-1.713727
H	5.279475	-0.391396	-3.151662
C	6.975144	1.279107	-1.711369
H	7.168862	1.316557	-2.790849
H	7.673747	0.547282	-1.288236
H	7.225549	2.264406	-1.298389
C	1.158899	3.417387	-0.643456
H	0.960217	2.546973	-0.002980
C	-0.096674	3.616630	-1.484810
H	-0.972786	3.696438	-0.827665
H	-0.262979	2.758213	-2.150238
H	-0.056925	4.533428	-2.090440
C	1.401287	4.625831	0.260584
H	2.217831	4.447803	0.972147
H	0.498106	4.859736	0.839178
H	1.662616	5.514175	-0.331968
C	2.337504	-2.154365	2.088210
C	1.192355	-2.426366	2.866803
C	0.953737	-3.751265	3.240583
H	0.095413	-3.979835	3.871237
C	1.785425	-4.784149	2.822999
H	1.580240	-5.808493	3.129912
C	2.855756	-4.505786	1.984632
H	3.477250	-5.321046	1.613349
C	3.148560	-3.195603	1.595454
C	0.268964	-1.316766	3.337733
H	0.364749	-0.486317	2.620541
C	-1.202066	-1.721118	3.355126
H	-1.407710	-2.547985	4.048078
H	-1.558243	-2.019153	2.361483
H	-1.822197	-0.876499	3.683334
C	0.678152	-0.798415	4.717317
H	0.698600	-1.614261	5.453449
H	-0.040005	-0.048190	5.073997
H	1.668399	-0.328014	4.709815
C	4.272575	-2.955528	0.604955
H	4.435760	-1.869616	0.519544
C	3.853773	-3.472063	-0.774713
H	3.606802	-4.541950	-0.731504
H	4.660436	-3.340968	-1.507566
H	2.966756	-2.938235	-1.146060
C	5.591755	-3.587375	1.042482
H	5.901706	-3.247855	2.038789
H	6.392658	-3.334369	0.336549
H	5.524722	-4.682595	1.075929
Al	-2.071554	0.142883	-0.059137
N	-3.298917	-1.429320	0.111088
N	-3.023208	1.063151	1.414366
C	-4.217253	-1.647498	1.050677
C	-4.414969	-0.770179	2.130604
H	-5.130987	-1.087989	2.883256
C	-3.901196	0.522828	2.272724
C	-5.144815	-2.825116	0.947667
H	-5.964552	-2.594355	0.253003
H	-5.593351	-3.060670	1.917481
H	-4.645650	-3.715963	0.553276
C	-4.398170	1.337377	3.432866
H	-3.577325	1.564513	4.125820
H	-5.176331	0.807060	3.988437

H	-4.797663	2.304513	3.103494
C	-3.261538	-2.285670	-1.042511
C	-4.181780	-2.078219	-2.088716
C	-4.104652	-2.899143	-3.217254
H	-4.808954	-2.743794	-4.035031
C	-3.141611	-3.893409	-3.318717
H	-3.098225	-4.526597	-4.203395
C	-2.219948	-4.062490	-2.292287
H	-1.450426	-4.827474	-2.383815
C	-2.248526	-3.261693	-1.147776
C	-5.193675	-0.949150	-2.072262
H	-5.196313	-0.492058	-1.071112
C	-4.774063	0.130582	-3.070968
H	-4.733612	-0.276277	-4.090383
H	-3.777251	0.529732	-2.833082
H	-5.488946	0.964149	-3.072366
C	-6.613816	-1.425788	-2.369905
H	-7.328711	-0.599831	-2.266810
H	-6.929781	-2.231809	-1.695909
H	-6.703910	-1.806249	-3.395600
C	-1.243293	-3.476384	-0.036269
H	-1.069328	-2.498034	0.439396
C	-1.799725	-4.434217	1.017516
H	-2.692509	-4.029856	1.511984
H	-1.049835	-4.628531	1.794250
H	-2.074622	-5.397023	0.563968
C	0.118917	-3.939123	-0.532175
H	0.831159	-3.946965	0.301366
H	0.507553	-3.248960	-1.292970
H	0.097386	-4.955928	-0.950141
C	-2.779923	2.478395	1.477632
C	-1.842551	3.028392	2.373401
C	-1.660907	4.415072	2.376696
H	-0.933909	4.850346	3.063105
C	-2.374508	5.242319	1.523207
H	-2.220379	6.319868	1.546616
C	-3.269952	4.683366	0.619966
H	-3.805854	5.333171	-0.068896
C	-3.479483	3.303927	0.564794
C	-0.994306	2.191234	3.311023
H	-1.359791	1.153970	3.274669
C	0.463930	2.185575	2.848962
H	0.859526	3.208838	2.780774
H	0.560032	1.723347	1.855566
H	1.103298	1.627855	3.545756
C	-1.084984	2.684386	4.755689
H	-0.533093	2.018263	5.430412
H	-2.120736	2.746086	5.112529
H	-0.645216	3.683922	4.864861
C	-4.436990	2.717921	-0.458241
H	-4.024664	1.741389	-0.769134
C	-4.546798	3.565275	-1.720267
H	-5.055483	4.520222	-1.535396
H	-5.132631	3.037412	-2.481047
H	-3.559574	3.782482	-2.147059
C	-5.821567	2.452530	0.133680
H	-6.268410	3.380393	0.516600
H	-5.787324	1.728095	0.956215
H	-6.497558	2.047712	-0.631878

H	-0.980674	0.853310	-3.646602
C	-0.107987	0.211485	-3.726540
C	1.149461	0.756317	-4.013995
C	-0.247178	-1.178957	-3.589747
C	2.248418	-0.078767	-4.209332
H	1.265364	1.835634	-4.100157
C	0.865469	-2.007786	-3.770538
H	-1.227519	-1.609074	-3.381936
C	2.105893	-1.460815	-4.096750
H	3.220177	0.358825	-4.435995
H	0.751934	-3.086715	-3.665122
H	2.969581	-2.111978	-4.231501

Int-2a-toluene_meta1.log

SCF (wB97x) = -2882.03133019
H(0 K)= -2880.620288
H(298 K)= -2880.539663
G(298 K)= -2880.734444
Lowest Frequency = 20.9116cm-1

Pd	0.009527	-0.139941	1.127364
Al	-1.967477	-0.137169	-0.190025
N	-3.125561	1.466017	-0.388406
N	-2.604239	-0.813366	-1.959126
C	-3.751765	1.897934	-1.485934
C	-3.862770	1.120519	-2.646298
H	-4.448899	1.544733	-3.456562
C	-3.433474	-0.201853	-2.814157
C	-4.417954	3.243960	-1.487384
H	-5.121264	3.345932	-0.651701
H	-4.953925	3.424166	-2.423389
H	-3.672195	4.039177	-1.357599
C	-4.015561	-0.957850	-3.977611
H	-3.295471	-1.624204	-4.462473
H	-4.429737	-0.272797	-4.723277
H	-4.837074	-1.597375	-3.624316
C	-3.341587	2.178619	0.840253
C	-4.328446	1.669461	1.713971
C	-4.606756	2.382965	2.880722
H	-5.384843	2.025117	3.553670
C	-3.899968	3.534891	3.208824
H	-4.131137	4.074369	4.126345
C	-2.884852	3.977693	2.373200
H	-2.304712	4.859639	2.646855
C	-2.584506	3.314857	1.179505
C	-5.080599	0.390313	1.389851
H	-4.401990	-0.244504	0.793032
C	-5.438137	-0.420387	2.629384
H	-4.541776	-0.663572	3.213157
H	-5.917599	-1.362837	2.337918
H	-6.144003	0.103650	3.286978
C	-6.316776	0.654442	0.530830
H	-7.024273	1.314245	1.051773
H	-6.839542	-0.285109	0.304584
H	-6.058458	1.125525	-0.425644
C	-1.429322	3.802567	0.331535
H	-1.438585	3.234856	-0.608161
C	-0.101137	3.508280	1.029554

H	0.744973	3.828962	0.405954
H	0.015870	2.430328	1.222940
H	-0.036858	4.040137	1.991098
C	-1.526037	5.287753	-0.012512
H	-2.477423	5.549593	-0.494012
H	-0.711202	5.571623	-0.691251
H	-1.433150	5.915080	0.884097
C	-2.287891	-2.188409	-2.222877
C	-1.099230	-2.498237	-2.917208
C	-0.852966	-3.837173	-3.229384
H	0.044399	-4.096626	-3.789502
C	-1.724931	-4.845902	-2.834903
H	-1.508462	-5.883076	-3.086087
C	-2.860021	-4.526229	-2.102902
H	-3.527152	-5.320505	-1.767540
C	-3.164894	-3.199838	-1.784910
C	-0.146053	-1.409057	-3.374351
H	-0.229874	-0.578501	-2.654394
C	1.316106	-1.840559	-3.391845
H	1.512925	-2.633969	-4.125616
H	1.655066	-2.196829	-2.411764
H	1.954812	-0.991517	-3.669666
C	-0.535449	-0.878405	-4.755286
H	-0.569396	-1.693542	-5.491703
H	0.202520	-0.145004	-5.106331
H	-1.514647	-0.385901	-4.754258
C	-4.391011	-2.897459	-0.944814
H	-4.568603	-1.811340	-0.970752
C	-4.138240	-3.289710	0.512799
H	-3.903146	-4.360213	0.592515
H	-5.025538	-3.093845	1.128995
H	-3.294274	-2.730686	0.942828
C	-5.650783	-3.580646	-1.472107
H	-5.834271	-3.350290	-2.529198
H	-6.529485	-3.259288	-0.899154
H	-5.588684	-4.672817	-1.381422
Al	2.073280	0.099527	-0.020141
N	3.316753	-1.456703	-0.158159
N	3.049205	1.038907	-1.461576
C	4.252546	-1.668590	-1.081924
C	4.471147	-0.782242	-2.150685
H	5.201866	-1.094173	-2.891676
C	3.953434	0.509229	-2.298913
C	5.171337	-2.851842	-0.972712
H	4.658542	-3.739909	-0.589067
H	5.984571	-2.632770	-0.266989
H	5.629663	-3.086559	-1.938143
C	4.465734	1.331149	-3.446853
H	3.657493	1.540617	-4.160395
H	5.267585	0.815255	-3.981947
H	4.836885	2.306698	-3.109683
C	3.275109	-2.290083	1.012240
C	4.202673	-2.066941	2.050019
C	4.114976	-2.848808	3.205258
H	4.824246	-2.677874	4.015533
C	3.131481	-3.817482	3.345139
H	3.076971	-4.417606	4.251982
C	2.203640	-4.001604	2.327599
H	1.419850	-4.748351	2.445790

C	2.243079	-3.242119	1.155603
C	5.225657	-0.948447	2.006300
H	5.230783	-0.509619	0.997184
C	4.815534	0.152703	2.985338
H	4.777413	-0.233141	4.012895
H	3.818865	0.551406	2.745565
H	5.533963	0.982910	2.966132
C	6.642435	-1.429628	2.312340
H	7.363163	-0.611043	2.192157
H	6.952432	-2.251239	1.654690
H	6.730435	-1.790065	3.345392
C	1.231810	-3.492638	0.057143
H	1.086049	-2.542911	-0.481106
C	1.771668	-4.531101	-0.926876
H	2.681477	-4.182404	-1.433540
H	1.026195	-4.757536	-1.698440
H	2.016691	-5.468429	-0.407841
C	-0.144026	-3.890397	0.577797
H	-0.863248	-3.904641	-0.251206
H	-0.507289	-3.163257	1.317622
H	-0.150351	-4.892504	1.030184
C	2.785995	2.451280	-1.526999
C	1.847728	2.985552	-2.430649
C	1.633217	4.367660	-2.430212
H	0.901814	4.788842	-3.120718
C	2.325818	5.207618	-1.571888
H	2.151909	6.282334	-1.595243
C	3.224188	4.664037	-0.661957
H	3.741462	5.322578	0.032847
C	3.456169	3.288323	-0.603526
C	1.022133	2.131743	-3.371761
H	1.398726	1.098200	-3.322992
C	-0.437564	2.115412	-2.917560
H	-0.855586	3.133292	-2.900929
H	-0.526325	1.701737	-1.901643
H	-1.063593	1.513276	-3.588241
C	1.119955	2.614368	-4.819005
H	0.565293	1.948037	-5.491122
H	2.157333	2.666071	-5.172771
H	0.688291	3.616659	-4.935191
C	4.401738	2.718948	0.438897
H	4.014135	1.724489	0.721098
C	4.437570	3.550315	1.715820
H	4.914341	4.526976	1.562890
H	5.015748	3.034274	2.490262
H	3.427286	3.722934	2.108893
C	5.811094	2.507661	-0.114209
H	6.241414	3.455190	-0.466551
H	5.821787	1.799043	-0.951509
H	6.475887	2.106997	0.663340
H	0.998470	1.201254	3.371099
C	0.275535	0.450133	3.679875
C	-0.939349	0.836860	4.249012
C	0.586837	-0.912256	3.546363
C	-1.846369	-0.115284	4.730245
H	-1.174995	1.898381	4.342738
C	-0.345736	-1.866409	3.961396
H	1.557193	-1.221152	3.155350
C	-1.540881	-1.468784	4.556768

H	-0.118822	-2.925402	3.838949
H	-2.253039	-2.221568	4.898269
C	-3.088077	0.309518	5.454491
H	-3.793155	-0.521332	5.575971
H	-2.852196	0.682681	6.460976
H	-3.606671	1.120857	4.929282

Int-2a-toluene_meta2.log

SCF (wB97x) = -2882.03389892
H(0 K)= -2880.622736
H(298 K)= -2880.542252
G(298 K)= -2880.736069
Lowest Frequency = 22.7697cm-1

Pd	-0.028444	-0.239648	-1.120646
Al	1.984240	-0.079502	0.139834
N	3.221707	1.475946	0.115666
N	2.640796	-0.576712	1.952474
C	3.850551	2.026705	1.158421
C	3.953695	1.387496	2.401000
H	4.557451	1.888479	3.152425
C	3.504692	0.099074	2.719218
C	4.545709	3.348127	1.000387
H	5.266926	3.324046	0.173865
H	5.069230	3.636621	1.916075
H	3.822408	4.135653	0.752369
C	4.101176	-0.547308	3.939403
H	3.376758	-1.132701	4.514161
H	4.561821	0.197988	4.594522
H	4.888828	-1.249597	3.631032
C	3.446394	2.038097	-1.188218
C	4.420588	1.417797	-2.003970
C	4.686247	1.977448	-3.255134
H	5.439966	1.521851	-3.895517
C	3.990041	3.091919	-3.710632
H	4.212095	3.511109	-4.690817
C	2.996049	3.649698	-2.919836
H	2.424583	4.501570	-3.290227
C	2.701302	3.137526	-1.652714
C	5.150875	0.168779	-1.539303
H	4.414393	-0.444769	-0.987927
C	5.663869	-0.694477	-2.685506
H	4.868949	-0.932641	-3.402059
H	6.059557	-1.639746	-2.293696
H	6.484289	-0.210429	-3.231706
C	6.299029	0.483008	-0.578388
H	7.037888	1.139282	-1.058813
H	6.817043	-0.440047	-0.284421
H	5.957785	0.972079	0.340482
C	1.561829	3.741462	-0.859545
H	1.573527	3.293374	0.142412
C	0.220794	3.385522	-1.502025
H	-0.611501	3.796287	-0.914548
H	0.082807	2.294160	-1.560367
H	0.152477	3.797344	-2.520481
C	1.682981	5.256005	-0.700341
H	2.643724	5.560381	-0.264578
H	0.880374	5.632728	-0.052851

H	1.587407	5.770667	-1.665728
C	2.288869	-1.905339	2.367209
C	1.121991	-2.097285	3.137403
C	0.847502	-3.386404	3.599376
H	-0.034533	-3.554341	4.215953
C	1.670238	-4.461311	3.280040
H	1.433798	-5.457659	3.650507
C	2.775159	-4.263291	2.463611
H	3.395908	-5.113494	2.180769
C	3.104637	-2.990051	1.990267
C	0.212431	-0.935011	3.493525
H	0.272490	-0.212151	2.662903
C	-1.253352	-1.329798	3.641293
H	-1.425719	-1.981269	4.508866
H	-1.637017	-1.844914	2.752574
H	-1.870878	-0.434099	3.792640
C	0.674195	-0.229458	4.769805
H	0.730786	-0.937449	5.608589
H	-0.037140	0.559135	5.049440
H	1.658222	0.239894	4.659292
C	4.275719	-2.823932	1.040174
H	4.502941	-1.749573	0.959484
C	3.885159	-3.319723	-0.355835
H	3.613023	-4.384484	-0.328779
H	4.717592	-3.201177	-1.062708
H	3.021932	-2.764063	-0.752234
C	5.541893	-3.523588	1.526911
H	5.825972	-3.205439	2.537912
H	6.382126	-3.306070	0.855774
H	5.421827	-4.614431	1.547786
Al	-2.086853	0.152446	-0.017342
N	-3.389707	-1.305483	0.377198
N	-2.988707	1.334916	1.297660
C	-4.284926	-1.354133	1.362685
C	-4.443198	-0.311886	2.290612
H	-5.156690	-0.484932	3.091306
C	-3.887542	0.970929	2.223479
C	-5.221705	-2.522843	1.475032
H	-5.672213	-2.575116	2.470563
H	-4.722890	-3.472072	1.252201
H	-6.038145	-2.425691	0.746239
C	-4.356498	1.976824	3.235880
H	-3.530542	2.277946	3.893501
H	-5.158514	1.571858	3.858857
H	-4.713470	2.895254	2.753849
C	-3.426397	-2.311055	-0.648563
C	-4.385032	-2.204562	-1.676998
C	-4.395039	-3.172086	-2.684459
H	-5.129028	-3.094554	-3.487063
C	-3.482420	-4.218156	-2.685899
H	-3.509710	-4.970208	-3.472855
C	-2.523078	-4.289105	-1.683785
H	-1.798929	-5.103389	-1.689852
C	-2.458745	-3.336025	-0.663225
C	-5.342904	-1.033380	-1.778684
H	-5.276265	-0.440797	-0.854030
C	-4.920268	-0.129216	-2.937471
H	-4.958938	-0.673440	-3.890605
H	-3.891035	0.235629	-2.806318

H	-5.587383	0.738992	-3.021520
C	-6.796615	-1.469496	-1.947627
H	-7.464559	-0.599032	-1.940946
H	-7.120845	-2.148339	-1.149010
H	-6.952922	-1.990776	-2.900911
C	-1.402992	-3.461129	0.414889
H	-1.287215	-2.472433	0.882264
C	-1.850861	-4.454065	1.487607
H	-2.768360	-4.125539	1.994196
H	-1.072450	-4.576676	2.250404
H	-2.049983	-5.441254	1.047768
C	-0.028885	-3.821888	-0.136378
H	0.713871	-3.798855	0.671557
H	0.285486	-3.089974	-0.894698
H	0.000105	-4.827309	-0.580618
C	-2.675908	2.730982	1.156943
C	-1.714313	3.356584	1.973717
C	-1.465597	4.719902	1.785234
H	-0.717320	5.211374	2.407990
C	-2.144611	5.454073	0.824624
H	-1.943700	6.517298	0.701936
C	-3.062919	4.816051	0.000089
H	-3.569282	5.387761	-0.775129
C	-3.331209	3.451947	0.131098
C	-0.897969	2.613982	3.012822
H	-1.302484	1.594338	3.109411
C	0.552858	2.494574	2.545579
H	0.998078	3.488769	2.389360
H	0.610808	1.948354	1.591878
H	1.173815	1.967681	3.281012
C	-0.955010	3.288509	4.383504
H	-0.411214	2.697679	5.130986
H	-1.983264	3.423535	4.741639
H	-0.487829	4.281296	4.359793
C	-4.307340	2.774330	-0.813966
H	-3.963150	1.734166	-0.949908
C	-4.321918	3.411003	-2.198756
H	-4.757358	4.418406	-2.189107
H	-4.927212	2.811021	-2.887418
H	-3.309113	3.482320	-2.615862
C	-5.717613	2.704919	-0.228411
H	-6.104476	3.710847	-0.014857
H	-5.746675	2.126597	0.703467
H	-6.407881	2.224475	-0.935245
H	-1.009571	0.594935	-3.518390
C	-0.133381	-0.035440	-3.644491
C	1.095379	0.523777	-4.009506
C	-0.246758	-1.426851	-3.484756
C	2.184098	-0.309711	-4.260577
H	1.198425	1.603772	-4.108787
C	0.851571	-2.267234	-3.717817
H	-1.211311	-1.863888	-3.214340
C	2.060990	-1.688173	-4.120210
H	2.920789	-2.333816	-4.309913
H	3.136233	0.127366	-4.558569
C	0.730357	-3.754957	-3.579255
H	-0.265828	-4.042791	-3.224031
H	0.906244	-4.264027	-4.536651
H	1.464363	-4.156070	-2.866940

Int-2a-toluene_ortho1.log

SCF (wB97x) = -2882.03252879
H(0 K)= -2880.621261
H(298 K)= -2880.540790
G(298 K)= -2880.734823
Lowest Frequency = 19.0316cm-1

Pd	0.033438	-0.164549	1.177654
Al	-1.971855	-0.158422	-0.105176
N	-3.272378	1.337387	-0.283276
N	-2.603724	-0.921726	-1.836095
C	-3.920901	1.714716	-1.391528
C	-3.988488	0.914488	-2.538793
H	-4.611727	1.285069	-3.347553
C	-3.487756	-0.385798	-2.685222
C	-4.695290	3.001436	-1.408273
H	-5.430900	3.030706	-0.594344
H	-5.217974	3.138216	-2.358857
H	-4.032007	3.861355	-1.251321
C	-4.057353	-1.206808	-3.809921
H	-3.314580	-1.845542	-4.297590
H	-4.531498	-0.568269	-4.561155
H	-4.830154	-1.880764	-3.413299
C	-3.508805	2.093943	0.917391
C	-4.424825	1.569908	1.858375
C	-4.711639	2.334294	2.992344
H	-5.421332	1.956679	3.725965
C	-4.101424	3.564526	3.210156
H	-4.344423	4.143978	4.099419
C	-3.161842	4.034637	2.304016
H	-2.651465	4.978988	2.494849
C	-2.841142	3.311667	1.151587
C	-5.065353	0.205550	1.659506
H	-4.260606	-0.474254	1.315884
C	-5.628598	-0.387823	2.945815
H	-4.905241	-0.357402	3.768165
H	-5.912428	-1.434920	2.784662
H	-6.533340	0.141433	3.274304
C	-6.162491	0.205002	0.592066
H	-6.959086	0.914212	0.856551
H	-6.619859	-0.791216	0.519231
H	-5.791145	0.466636	-0.404190
C	-1.746524	3.814761	0.234749
H	-1.802029	3.243516	-0.699861
C	-0.376510	3.537121	0.854797
H	0.428282	3.846732	0.174890
H	-0.239623	2.464462	1.068937
H	-0.257195	4.091428	1.797647
C	-1.880065	5.294396	-0.116856
H	-2.864564	5.541102	-0.536380
H	-1.115875	5.575514	-0.853136
H	-1.729622	5.936131	0.761179
C	-2.218296	-2.284675	-2.073506
C	-1.053289	-2.547921	-2.824384
C	-0.762509	-3.878137	-3.135572
H	0.116491	-4.104090	-3.737796
C	-1.567601	-4.919627	-2.686884

H	-1.319464	-5.948930	-2.941433
C	-2.669532	-4.643560	-1.888780
H	-3.275873	-5.463731	-1.504351
C	-3.015472	-3.328552	-1.565052
C	-0.169671	-1.421487	-3.329511
H	-0.241879	-0.600514	-2.596548
C	1.303476	-1.801297	-3.438140
H	1.484104	-2.558156	-4.213558
H	1.705476	-2.184989	-2.493159
H	1.899564	-0.920585	-3.712804
C	-0.652851	-0.894235	-4.682071
H	-0.702085	-1.705743	-5.421716
H	0.042527	-0.136934	-5.067736
H	-1.644539	-0.431298	-4.624655
C	-4.190909	-3.066600	-0.642109
H	-4.432011	-1.993086	-0.687648
C	-3.801297	-3.387483	0.803797
H	-3.527522	-4.446908	0.909095
H	-4.637361	-3.185199	1.487900
H	-2.940262	-2.786772	1.131743
C	-5.445860	-3.835878	-1.046266
H	-5.729287	-3.644703	-2.088959
H	-6.292503	-3.551998	-0.409119
H	-5.311082	-4.919668	-0.936700
Al	2.087425	0.155266	0.038749
N	3.411435	-1.322238	-0.193387
N	2.954283	1.179484	-1.431318
C	4.313745	-1.468013	-1.162749
C	4.452518	-0.543471	-2.210123
H	5.176861	-0.794497	-2.979750
C	3.866651	0.723734	-2.302633
C	5.285201	-2.613392	-1.127221
H	5.743315	-2.774167	-2.107573
H	4.816116	-3.543202	-0.789099
H	6.093874	-2.400136	-0.414504
C	4.324955	1.607423	-3.428321
H	3.499772	1.814666	-4.121990
H	5.137123	1.142409	-3.993495
H	4.665310	2.582857	-3.059408
C	3.466503	-2.204014	0.940044
C	4.401440	-1.944118	1.963168
C	4.432892	-2.795735	3.070065
H	5.147135	-2.599312	3.870219
C	3.572539	-3.881063	3.167131
H	3.619699	-4.542840	4.030367
C	2.638060	-4.104952	2.164426
H	1.948210	-4.943753	2.251910
C	2.546056	-3.266019	1.050023
C	5.325410	-0.741158	1.935445
H	5.256680	-0.266528	0.945062
C	4.871007	0.288974	2.970154
H	4.888342	-0.138819	3.981595
H	3.845310	0.630891	2.770460
H	5.533156	1.165233	2.966992
C	6.787286	-1.117744	2.168331
H	7.435157	-0.239377	2.055846
H	7.134490	-1.884635	1.464707
H	6.945980	-1.510107	3.181144
C	1.508490	-3.547613	-0.017263

H	1.351951	-2.616552	-0.581659
C	2.013257	-4.619533	-0.983888
H	2.917891	-4.300004	-1.518179
H	1.247967	-4.854581	-1.733588
H	2.254727	-5.547542	-0.447046
C	0.145148	-3.919645	0.554721
H	-0.573634	-4.058800	-0.261916
H	-0.236968	-3.114873	1.197039
H	0.167060	-4.855123	1.132029
C	2.615242	2.575994	-1.478420
C	1.670614	3.074437	-2.396436
C	1.414762	4.449174	-2.409506
H	0.681522	4.843988	-3.113457
C	2.066179	5.315232	-1.543743
H	1.859246	6.383822	-1.576700
C	2.962076	4.804358	-0.613008
H	3.446821	5.481321	0.087903
C	3.240591	3.437401	-0.547482
C	0.871464	2.182357	-3.325278
H	1.298269	1.168196	-3.285817
C	-0.573763	2.096551	-2.832978
H	-1.043245	3.091607	-2.825561
H	-0.612291	1.700370	-1.806510
H	-1.184101	1.448998	-3.475088
C	0.907256	2.665453	-4.774665
H	0.373284	1.967161	-5.430861
H	1.930743	2.771696	-5.155599
H	0.418166	3.641775	-4.883628
C	4.203080	2.907079	0.499401
H	3.878965	1.883224	0.754827
C	4.159869	3.707720	1.795445
H	4.574714	4.716877	1.676629
H	4.753579	3.209551	2.570284
H	3.131788	3.803672	2.168244
C	5.629670	2.802647	-0.039020
H	6.000418	3.784243	-0.364916
H	5.694927	2.116442	-0.892949
H	6.311559	2.428558	0.736836
H	1.312759	0.040867	3.624855
C	0.306376	-0.364481	3.685567
C	-0.774078	0.491781	3.957661
C	0.100451	-1.741521	3.528923
C	-2.043639	-0.074993	4.134071
C	-1.172435	-2.283859	3.703766
H	0.948168	-2.383510	3.290415
C	-2.239262	-1.449565	4.032770
H	-3.235125	-1.871240	4.169204
H	-2.886458	0.586115	4.345355
H	-1.328649	-3.355204	3.577378
C	-0.553955	1.966019	4.109692
H	0.385579	2.275803	3.635232
H	-1.367664	2.541863	3.651201
H	-0.505272	2.260007	5.168212

Int-2a-toluene_ortho2.log

SCF (wB97x) = -2882.03271461
H(0 K)= -2880.622448
H(298 K)= -2880.541682

G(298 K)= -2880.737020
Lowest Frequency = 25.0085cm-1

Pd	-0.015975	0.087762	1.130510
Al	2.024358	0.204389	-0.096387
N	3.426402	-1.195100	-0.228293
N	2.624724	0.966769	-1.835154
C	4.077537	-1.579640	-1.331509
C	4.105766	-0.805483	-2.498334
H	4.739595	-1.167366	-3.303056
C	3.545733	0.468351	-2.667025
C	4.878479	-2.849591	-1.319809
H	5.635962	-2.838226	-0.525992
H	5.377593	-3.017057	-2.278318
H	4.229438	-3.709844	-1.110436
C	4.078444	1.305737	-3.796560
H	3.301537	1.884512	-4.305840
H	4.606533	0.689224	-4.530002
H	4.797161	2.036445	-3.398604
C	3.749038	-1.847996	1.011048
C	4.718059	-1.231439	1.836685
C	5.076459	-1.872226	3.024272
H	5.826943	-1.420282	3.670936
C	4.469181	-3.061167	3.414646
H	4.758804	-3.540579	4.348319
C	3.477973	-3.618624	2.620246
H	2.978807	-4.533634	2.940964
C	3.098829	-3.029843	1.409753
C	5.332417	0.106356	1.457912
H	4.527562	0.703288	0.991146
C	5.834032	0.905238	2.655281
H	5.062807	1.020404	3.425749
H	6.139903	1.908336	2.332616
H	6.713528	0.441038	3.120592
C	6.459946	-0.028940	0.432357
H	7.265102	-0.665235	0.825086
H	6.894505	0.954746	0.208441
H	6.120410	-0.457732	-0.516128
C	1.982688	-3.655746	0.600769
H	1.930693	-3.129983	-0.360993
C	0.635003	-3.463492	1.296734
H	-0.173352	-3.910765	0.703472
H	0.399327	-2.394002	1.426115
H	0.631967	-3.941476	2.288158
C	2.222688	-5.137201	0.314386
H	3.192530	-5.321654	-0.166391
H	1.435839	-5.527075	-0.344292
H	2.198283	-5.734652	1.235430
C	2.137157	2.291578	-2.096622
C	0.950261	2.446831	-2.845112
C	0.524341	3.744698	-3.133409
H	-0.383555	3.891845	-3.716696
C	1.226527	4.855338	-2.676430
H	0.874520	5.858055	-2.915284
C	2.357390	4.681424	-1.891519
H	2.883624	5.553168	-1.502512
C	2.832059	3.403185	-1.583281
C	0.171973	1.241492	-3.341931
H	0.263574	0.460995	-2.567132

C	-1.315053	1.514870	-3.536033
H	-1.505363	2.196803	-4.376081
H	-1.780394	1.945109	-2.640640
H	-1.843956	0.579551	-3.761926
C	0.744204	0.683097	-4.646617
H	0.768553	1.458505	-5.425192
H	0.114404	-0.137426	-5.016623
H	1.759603	0.288939	-4.531471
C	4.026867	3.249878	-0.661339
H	4.369681	2.205111	-0.715846
C	3.605189	3.525301	0.785279
H	3.232397	4.553653	0.891673
H	4.451856	3.400707	1.473811
H	2.802577	2.846464	1.109378
C	5.204322	4.136150	-1.058664
H	5.506108	3.977776	-2.101720
H	6.072441	3.928200	-0.420516
H	4.969238	5.202051	-0.943083
Al	-2.087887	-0.232120	0.028838
N	-3.502229	1.095253	-0.403624
N	-2.811091	-1.464540	-1.368346
C	-4.297938	1.120092	-1.476211
C	-4.339146	0.079792	-2.412616
H	-5.007818	0.217133	-3.257459
C	-3.706063	-1.166444	-2.318647
C	-5.224329	2.282836	-1.689860
H	-4.680044	3.233431	-1.624428
H	-5.995568	2.325100	-0.910430
H	-5.720627	2.223901	-2.662416
C	-4.087322	-2.200738	-3.339676
H	-3.240377	-2.434614	-3.996883
H	-4.916476	-1.855329	-3.962894
H	-4.373554	-3.146346	-2.863274
C	-3.768433	2.047758	0.640194
C	-4.777517	1.736636	1.576713
C	-5.069761	2.664296	2.578184
H	-5.847214	2.431102	3.306638
C	-4.382482	3.868024	2.666459
H	-4.629115	4.585270	3.447706
C	-3.359262	4.134802	1.768356
H	-2.792114	5.062100	1.857086
C	-3.018743	3.232972	0.755512
C	-5.505392	0.405344	1.578161
H	-5.196051	-0.166363	0.691174
C	-5.095069	-0.403201	2.809523
H	-5.347214	0.130277	3.736061
H	-4.011091	-0.590835	2.819433
H	-5.611781	-1.372252	2.831859
C	-7.022376	0.567160	1.515193
H	-7.516609	-0.411876	1.478537
H	-7.337136	1.133180	0.629688
H	-7.409041	1.094697	2.397009
C	-1.854326	3.566489	-0.152411
H	-1.708705	2.726256	-0.845486
C	-2.136035	4.823558	-0.973690
H	-3.013863	4.703289	-1.623268
H	-1.277069	5.069555	-1.608514
H	-2.326421	5.688271	-0.323693
C	-0.556356	3.704601	0.642832

H	0.285267	3.906844	-0.034348
H	-0.326984	2.773681	1.185425
H	-0.610107	4.527579	1.370997
C	-2.356410	-2.825446	-1.278721
C	-1.354526	-3.317746	-2.138254
C	-0.986596	-4.662305	-2.029945
H	-0.208618	-5.051812	-2.687511
C	-1.586195	-5.505076	-1.105630
H	-1.293770	-6.552393	-1.046438
C	-2.540924	-4.994934	-0.234684
H	-2.984574	-5.649513	0.513089
C	-2.929009	-3.654383	-0.287024
C	-0.613817	-2.444899	-3.132843
H	-1.114178	-1.465152	-3.178389
C	0.815272	-2.212893	-2.642800
H	1.358864	-3.166176	-2.560508
H	0.809175	-1.742698	-1.647667
H	1.381266	-1.568659	-3.327289
C	-0.590767	-3.041137	-4.539986
H	-0.107315	-2.351996	-5.243701
H	-1.594967	-3.263227	-4.921751
H	-0.019923	-3.978120	-4.567444
C	-3.953314	-3.118634	0.696197
H	-3.700307	-2.060859	0.888000
C	-3.899929	-3.823661	2.046186
H	-4.251136	-4.862021	1.988713
H	-4.544448	-3.309817	2.768462
H	-2.879956	-3.831198	2.451462
C	-5.367222	-3.145618	0.116404
H	-5.659982	-4.168245	-0.158922
H	-5.455494	-2.516163	-0.778378
H	-6.095285	-2.776750	0.851600
H	-0.930263	-0.785452	3.616603
C	-0.078208	-0.118091	3.730314
C	1.217053	-0.639558	3.802220
C	-0.296062	1.268101	3.859233
C	2.302305	0.203696	4.047896
C	0.810004	2.097787	4.062484
C	2.096772	1.572391	4.179009
H	2.940154	2.240312	4.355495
H	3.301673	-0.220336	4.125410
H	0.650816	3.173836	4.148588
H	1.378748	-1.711758	3.691538
C	-1.685168	1.824765	3.818466
H	-2.283324	1.486420	4.676965
H	-1.682178	2.920341	3.826321
H	-2.217254	1.499359	2.912442

Int-2a-toluene_para.log

SCF (wB97x) = -2882.03432711
H(0 K)= -2880.623486
H(298 K)= -2880.542931
G(298 K)= -2880.736836
Lowest Frequency = 24.8488cm-1

Pd	0.027596	0.257996	-1.114834
Al	-1.961442	0.001114	0.167056
N	-3.086649	-1.635837	0.059327

N	-2.644844	0.348932	2.010946
C	-3.691052	-2.277531	1.062858
C	-3.827174	-1.720917	2.341849
H	-4.398432	-2.300902	3.061067
C	-3.450595	-0.432174	2.740479
C	-4.322373	-3.618631	0.823599
H	-5.018484	-3.585720	-0.023746
H	-4.860023	-3.970696	1.708329
H	-3.559680	-4.364015	0.564769
C	-4.066781	0.089670	4.009921
H	-3.380812	0.704159	4.601004
H	-4.441606	-0.729747	4.630384
H	-4.922693	0.734076	3.762742
C	-3.241020	-2.147376	-1.275139
C	-4.202381	-1.524131	-2.104187
C	-4.383615	-2.023776	-3.395371
H	-5.122913	-1.562998	-4.048367
C	-3.629218	-3.091595	-3.869363
H	-3.789490	-3.467636	-4.878702
C	-2.657580	-3.658524	-3.057516
H	-2.041304	-4.472959	-3.439901
C	-2.436938	-3.197977	-1.756049
C	-5.015079	-0.339777	-1.607703
H	-4.328949	0.292569	-1.013070
C	-5.553703	0.536577	-2.731586
H	-4.762576	0.834374	-3.429191
H	-5.998194	1.450466	-2.318800
H	-6.342097	0.030957	-3.305056
C	-6.163625	-0.758691	-0.687944
H	-6.850823	-1.438934	-1.209832
H	-6.742528	0.120969	-0.374342
H	-5.814779	-1.260337	0.221344
C	-1.308623	-3.799306	-0.943958
H	-1.378596	-3.403777	0.077876
C	0.042374	-3.361499	-1.510836
H	0.867123	-3.768337	-0.909253
H	0.138605	-2.264286	-1.512248
H	0.167481	-3.718789	-2.544449
C	-1.373987	-5.323829	-0.866435
H	-2.340482	-5.689601	-0.495751
H	-0.588431	-5.701986	-0.199152
H	-1.209875	-5.783193	-1.850254
C	-2.394011	1.675520	2.498252
C	-1.218425	1.927173	3.236150
C	-1.032892	3.210715	3.755358
H	-0.146185	3.420431	4.352046
C	-1.952067	4.227348	3.521874
H	-1.782116	5.221351	3.932576
C	-3.075947	3.973790	2.747493
H	-3.781164	4.778803	2.540515
C	-3.320630	2.701008	2.225091
C	-0.216235	0.823937	3.521869
H	-0.268960	0.109496	2.684150
C	1.226333	1.309833	3.604746
H	1.393283	1.998011	4.444517
H	1.545551	1.816028	2.686032
H	1.901448	0.457701	3.759470
C	-0.576878	0.073747	4.805302
H	-0.648382	0.765780	5.656068

H	0.195646	-0.668352	5.045579
H	-1.531857	-0.458507	4.724445
C	-4.533647	2.474500	1.342987
H	-4.670085	1.389592	1.215006
C	-4.287118	3.071514	-0.044197
H	-4.116093	4.155274	0.020371
H	-5.150591	2.904390	-0.702816
H	-3.402051	2.623274	-0.519503
C	-5.819306	3.031334	1.949309
H	-5.996799	2.648540	2.962270
H	-6.684075	2.762320	1.330391
H	-5.797867	4.126868	2.012684
Al	2.087113	-0.056285	0.008736
N	3.252837	1.524574	0.371992
N	3.110619	-1.148796	1.297408
C	4.182346	1.641305	1.318415
C	4.441636	0.620976	2.250888
H	5.157735	0.857019	3.033171
C	3.986417	-0.701596	2.209728
C	5.051480	2.864861	1.383016
H	5.888620	2.768243	0.677789
H	5.479854	2.991979	2.381914
H	4.509900	3.773814	1.102067
C	4.534532	-1.655094	3.232697
H	3.732118	-2.016379	3.890052
H	5.298437	-1.180428	3.854475
H	4.966932	-2.545919	2.760573
C	3.161729	2.513867	-0.667088
C	4.090121	2.489801	-1.727372
C	3.950112	3.421002	-2.760690
H	4.660692	3.405098	-3.587609
C	2.913681	4.343348	-2.762159
H	2.818375	5.060684	-3.575740
C	1.986469	4.331591	-1.727395
H	1.161886	5.042561	-1.739116
C	2.078398	3.419593	-0.673481
C	5.171272	1.433023	-1.844429
H	5.204532	0.852903	-0.910057
C	4.815880	0.465371	-2.974152
H	4.753466	0.993537	-3.935115
H	3.842876	-0.016254	-2.797689
H	5.577518	-0.319330	-3.073153
C	6.558831	2.028818	-2.072842
H	7.323025	1.241399	-2.070710
H	6.827138	2.761811	-1.301915
H	6.622485	2.539515	-3.042401
C	1.068327	3.458757	0.453850
H	0.945423	2.427271	0.820959
C	1.592760	4.325235	1.599499
H	2.512238	3.914886	2.037594
H	0.847281	4.400945	2.400294
H	1.815269	5.342382	1.247267
C	-0.318699	3.913224	0.015407
H	-1.033515	3.760190	0.834173
H	-0.670276	3.326550	-0.845040
H	-0.348136	4.980899	-0.246003
C	2.919054	-2.566817	1.151919
C	2.003526	-3.274618	1.953953
C	1.862086	-4.651040	1.750006

H	1.148899	-5.205026	2.361092
C	2.601682	-5.320093	0.787135
H	2.484059	-6.394069	0.651262
C	3.472943	-4.603492	-0.023850
H	4.025974	-5.125447	-0.802106
C	3.633903	-3.224223	0.122767
C	1.121161	-2.608669	2.990037
H	1.447883	-1.563359	3.102631
C	-0.328962	-2.591242	2.504134
H	-0.698785	-3.613251	2.331626
H	-0.416216	-2.039948	1.555050
H	-0.995352	-2.119145	3.237572
C	1.217368	-3.294344	4.352763
H	0.612206	-2.766502	5.099938
H	2.249177	-3.340636	4.723434
H	0.842458	-4.324783	4.309327
C	4.550723	-2.460791	-0.816096
H	4.114059	-1.455802	-0.950949
C	4.628032	-3.091967	-2.201267
H	5.151357	-4.056625	-2.189779
H	5.181231	-2.440524	-2.886759
H	3.627388	-3.252848	-2.623056
C	5.947554	-2.264775	-0.227017
H	6.425279	-3.231683	-0.017189
H	5.922143	-1.690180	0.707064
H	6.591144	-1.719229	-0.930739
H	1.059330	-0.719783	-3.563062
C	0.262137	0.012701	-3.652215
C	-1.016918	-0.373902	-4.065274
C	0.521643	1.370216	-3.400808
C	-2.010250	0.583711	-4.261133
H	-1.234932	-1.425920	-4.247977
C	-0.498758	2.311067	-3.556732
H	1.523347	1.694602	-3.114641
C	-1.773634	1.935695	-3.998231
H	-2.996219	0.265700	-4.603166
H	-0.289517	3.360855	-3.343958
C	-2.868872	2.950869	-4.117654
H	-3.371614	3.105740	-3.150756
H	-2.489281	3.928887	-4.437249
H	-3.640045	2.638467	-4.832304

Int-2b_cc-PPdAl.log

SCF (wB97x) = -2648.55898393
H(0 K)= -2647.330990
H(298 K)= -2647.266737
G(298 K)= -2647.431584
Lowest Frequency = 18.1251cm-1

Pd	-0.444247	0.539644	-0.339440
P	-2.677875	-0.062426	-0.007880
C	-3.116824	-1.554318	-1.036846
H	-4.189147	-1.781968	-0.900763
C	-2.326577	-2.807469	-0.649985
H	-1.243914	-2.590733	-0.717068
H	-2.519862	-3.084043	0.396772
C	-2.685785	-3.977935	-1.559070
H	-2.124855	-4.872312	-1.257838

H	-3.753855	-4.222680	-1.430830
C	-2.421542	-3.642404	-3.020359
H	-2.681297	-4.490238	-3.669657
H	-1.340915	-3.463394	-3.151339
C	-3.191022	-2.393627	-3.433485
H	-4.272868	-2.604984	-3.392203
H	-2.969169	-2.127286	-4.476402
C	-2.872580	-1.220149	-2.514079
H	-3.454168	-0.333677	-2.811610
H	-1.810097	-0.935295	-2.624192
C	-4.039964	1.135471	-0.492270
H	-3.644233	1.546677	-1.437708
C	-5.442774	0.600471	-0.801128
H	-5.892735	0.155255	0.098338
H	-5.395281	-0.202070	-1.550207
C	-6.350295	1.716869	-1.313323
H	-5.954029	2.087063	-2.274201
H	-7.353948	1.322785	-1.524864
C	-6.422081	2.875374	-0.326742
H	-6.884441	2.523593	0.610650
H	-7.072837	3.672206	-0.712002
C	-5.029125	3.415316	-0.028660
H	-5.077044	4.240624	0.695497
H	-4.601857	3.836210	-0.953096
C	-4.105868	2.315050	0.483479
H	-3.092274	2.709666	0.660183
H	-4.480224	1.964733	1.460087
C	-3.039551	-0.647910	1.727677
H	-2.466987	-1.590371	1.765682
C	-2.401147	0.260571	2.783029
H	-1.345418	0.421675	2.514892
H	-2.876098	1.254881	2.767281
C	-2.525312	-0.335558	4.181114
H	-2.098525	0.351146	4.926217
H	-1.924305	-1.258408	4.230517
C	-3.971215	-0.673104	4.526245
H	-4.035926	-1.123499	5.526160
H	-4.562865	0.256580	4.566142
C	-4.576320	-1.601146	3.479497
H	-5.620047	-1.838910	3.727362
H	-4.028798	-2.559110	3.484298
C	-4.487208	-0.984618	2.086021
H	-5.094662	-0.065149	2.067786
H	-4.925791	-1.661008	1.336903
Al	1.731068	-0.153275	0.303728
N	2.672014	-1.877498	0.482656
N	3.350265	0.811332	0.894740
C	3.888423	-2.100980	0.994439
C	4.700082	-1.077391	1.501275
H	5.647197	-1.383003	1.936340
C	4.468142	0.301518	1.420072
C	4.437342	-3.498847	1.027700
H	3.841384	-4.147440	1.682527
H	5.471363	-3.511283	1.382158
H	4.399073	-3.958118	0.032430
C	5.553344	1.222110	1.898825
H	6.053111	1.710649	1.051668
H	6.311143	0.680814	2.471919
H	5.144861	2.030191	2.516952

C	1.943231	-2.991622	-0.057124
C	1.261042	-3.870635	0.810334
C	0.570629	-4.947968	0.253811
H	0.036440	-5.635248	0.909540
C	0.544427	-5.154023	-1.121130
H	0.011249	-6.007600	-1.537329
C	1.179965	-4.252884	-1.962493
H	1.135946	-4.398963	-3.042451
C	1.879554	-3.154029	-1.454290
C	1.202684	-3.617740	2.303820
H	2.043714	-2.964887	2.575656
C	-0.081581	-2.860287	2.647670
H	-0.177327	-1.936401	2.055547
H	-0.106518	-2.594086	3.712757
H	-0.965085	-3.480074	2.434236
C	1.321768	-4.889200	3.138315
H	0.450179	-5.543940	3.012791
H	1.383868	-4.642496	4.204868
H	2.212325	-5.473999	2.874298
C	2.525323	-2.186234	-2.426319
H	3.022606	-1.393931	-1.845655
C	1.464413	-1.517243	-3.300596
H	1.919049	-0.772801	-3.968387
H	0.713476	-1.008567	-2.674979
H	0.941445	-2.252110	-3.928735
C	3.598592	-2.864128	-3.275593
H	4.384533	-3.313051	-2.655329
H	4.073611	-2.141774	-3.951347
H	3.172331	-3.662955	-3.896716
C	3.286168	2.223707	0.648658
C	3.942506	2.742859	-0.489846
C	3.841074	4.109847	-0.750758
H	4.336432	4.524138	-1.627839
C	3.102687	4.947579	0.077769
H	3.035399	6.011967	-0.140807
C	2.437823	4.415381	1.172610
H	1.845478	5.068996	1.813828
C	2.503640	3.051129	1.475656
C	4.638639	1.831108	-1.483364
H	4.939039	0.909124	-0.965013
C	3.643923	1.431647	-2.576634
H	3.321283	2.312240	-3.149223
H	2.736708	0.975259	-2.147468
H	4.089312	0.712437	-3.277607
C	5.896874	2.439779	-2.093498
H	5.666506	3.294141	-2.742213
H	6.416139	1.700275	-2.714451
H	6.598334	2.788351	-1.325214
C	1.742833	2.532269	2.679150
H	1.832494	1.435767	2.695572
C	0.255303	2.864919	2.579127
H	0.083717	3.950078	2.558497
H	-0.288389	2.462354	3.443810
H	-0.184352	2.427399	1.667423
C	2.341219	3.066381	3.980222
H	3.398910	2.793694	4.086761
H	1.802558	2.669526	4.849569
H	2.278509	4.162071	4.023667
H	0.428876	1.337310	-2.930960

C	-0.213548	2.134392	-2.562977
C	-1.472981	2.345140	-3.128715
C	0.245736	2.979757	-1.544723
C	-2.275399	3.392220	-2.676467
H	-1.825325	1.688733	-3.923887
C	-0.572752	4.009444	-1.077567
H	1.243285	2.840851	-1.129673
C	-1.829884	4.216600	-1.643603
H	-3.257608	3.555609	-3.120906
H	-0.217215	4.653726	-0.273942
H	-2.464238	5.024829	-1.280501

Int-2b-toluene_meta1.log

SCF (wB97x) = -2687.86525337
H(0 K)= -2686.609316
H(298 K)= -2686.543353
G(298 K)= -2686.713146
Lowest Frequency = 11.3904cm-1

Pd	0.599347	0.025193	-0.769384
P	2.230730	-0.260359	0.857560
C	2.285791	1.279120	1.909368
H	3.061330	1.168451	2.688443
C	0.950188	1.566617	2.602750
H	0.155207	1.592940	1.834460
H	0.688070	0.749235	3.291693
C	0.981897	2.891400	3.359936
H	-0.000424	3.084854	3.816330
H	1.702434	2.821323	4.192106
C	1.374797	4.048172	2.449734
H	1.403789	4.991248	3.013673
H	0.600481	4.169506	1.674448
C	2.710756	3.775827	1.771578
H	3.510491	3.728951	2.530109
H	2.976540	4.599098	1.092916
C	2.656728	2.461162	1.005706
H	3.607347	2.272986	0.485265
H	1.886663	2.527008	0.215351
C	4.033545	-0.454366	0.366965
H	4.104820	0.228039	-0.499771
C	5.119176	-0.024985	1.360716
H	5.083541	-0.655205	2.262149
H	4.949106	1.005909	1.700812
C	6.505780	-0.128910	0.730207
H	6.565822	0.576609	-0.116373
H	7.274764	0.183574	1.450284
C	6.780984	-1.538255	0.222180
H	6.785642	-2.235256	1.076944
H	7.779920	-1.598788	-0.230966
C	5.712490	-1.967657	-0.775021
H	5.896389	-2.993134	-1.124596
H	5.768408	-1.318149	-1.662146
C	4.315339	-1.861297	-0.173040
H	3.550260	-2.136011	-0.916259
H	4.229811	-2.593295	0.646833
C	1.816889	-1.608761	2.079582
H	0.912755	-1.201080	2.568025
C	1.365740	-2.898144	1.379013

H	0.585542	-2.654922	0.639506
H	2.202902	-3.330620	0.809392
C	0.863997	-3.929084	2.384391
H	0.582180	-4.855416	1.864676
H	-0.057184	-3.549900	2.856281
C	1.895839	-4.209270	3.470853
H	1.508554	-4.940134	4.193983
H	2.789095	-4.668186	3.015085
C	2.305381	-2.922499	4.177455
H	3.055972	-3.126307	4.953630
H	1.429166	-2.498726	4.695736
C	2.839513	-1.898642	3.179900
H	3.761589	-2.297726	2.727675
H	3.126384	-0.970011	3.696076
Al	-1.752005	0.129150	-0.884483
N	-3.179562	1.413260	-0.447738
N	-3.097065	-1.290437	-1.136736
C	-4.436861	1.337046	-0.901384
C	-4.970715	0.169621	-1.466035
H	-5.996242	0.226738	-1.818923
C	-4.372705	-1.100343	-1.475331
C	-5.336055	2.534347	-0.790370
H	-5.424370	2.869338	0.250705
H	-6.336343	2.320385	-1.176001
H	-4.920783	3.384582	-1.347136
C	-5.223628	-2.277589	-1.849869
H	-6.171564	-1.960904	-2.293455
H	-5.450629	-2.888417	-0.966186
H	-4.700433	-2.939379	-2.550400
C	-2.788230	2.580075	0.291580
C	-2.830827	2.523932	1.702409
C	-2.504804	3.679050	2.416786
H	-2.543245	3.664442	3.504429
C	-2.113082	4.843874	1.766857
H	-1.859630	5.731526	2.344721
C	-2.001587	4.854646	0.384109
H	-1.643059	5.751421	-0.121560
C	-2.326159	3.728571	-0.377694
C	-3.190316	1.237637	2.425972
H	-2.735516	0.412882	1.849546
C	-2.616051	1.173476	3.836704
H	-1.542458	1.395835	3.851238
H	-2.757562	0.172198	4.259472
H	-3.116344	1.878220	4.513977
C	-4.697979	0.979927	2.470908
H	-5.221306	1.811615	2.962643
H	-4.913299	0.068182	3.044952
H	-5.132365	0.845954	1.473879
C	-2.092623	3.751240	-1.874516
H	-2.558249	2.854621	-2.309207
C	-0.589473	3.667977	-2.152240
H	-0.385740	3.632130	-3.230951
H	-0.158622	2.764495	-1.693353
H	-0.067220	4.542526	-1.736723
C	-2.704966	4.972627	-2.555367
H	-3.779282	5.065640	-2.350543
H	-2.573729	4.913805	-3.642453
H	-2.227642	5.903362	-2.222750
C	-2.598510	-2.622248	-0.952691

C	-1.802159	-3.222365	-1.947627
C	-1.274241	-4.492073	-1.696640
H	-0.662223	-4.968918	-2.462834
C	-1.508652	-5.149682	-0.496898
H	-1.084287	-6.136829	-0.320115
C	-2.278328	-4.534209	0.482397
H	-2.442727	-5.038385	1.435822
C	-2.830984	-3.268427	0.279256
C	-1.504834	-2.550568	-3.272519
H	-1.963304	-1.551315	-3.259447
C	-0.002624	-2.357388	-3.470548
H	0.521214	-3.320358	-3.549264
H	0.434388	-1.797420	-2.626092
H	0.193041	-1.794148	-4.393121
C	-2.112014	-3.328850	-4.439389
H	-1.681446	-4.336668	-4.510874
H	-1.917795	-2.819215	-5.391263
H	-3.198385	-3.443980	-4.335318
C	-3.603677	-2.610343	1.406313
H	-4.005882	-1.653468	1.043306
C	-2.670519	-2.301582	2.576694
H	-2.267798	-3.227364	3.009245
H	-3.206869	-1.770493	3.374238
H	-1.815980	-1.683379	2.264265
C	-4.779495	-3.462056	1.880972
H	-5.469199	-3.707505	1.064021
H	-5.351842	-2.937155	2.656289
H	-4.437310	-4.411162	2.313986
H	1.079522	0.712778	-3.080337
C	2.169133	0.808742	-3.069906
C	2.757149	1.960967	-2.544231
C	2.973594	-0.203777	-3.590471
C	4.144683	2.123085	-2.525056
H	2.122303	2.745635	-2.131811
C	4.358418	-0.047314	-3.594977
H	2.521770	-1.109331	-3.992049
C	4.937444	1.102967	-3.066919
H	4.992707	-0.830453	-4.009013
H	6.023502	1.211578	-3.065382
C	4.773086	3.345380	-1.925000
H	5.440416	3.852986	-2.633272
H	4.015832	4.068631	-1.601418
H	5.382720	3.094883	-1.044664

Int-2b-toluene_meta2.log

SCF (wB97x) = -2687.86441221
H(0 K)= -2686.608612
H(298 K)= -2686.542588
G(298 K)= -2686.712360
Lowest Frequency = 13.6195cm-1

Pd	0.626495	0.088751	-0.741299
P	2.187183	0.153894	0.979287
C	1.988470	1.773365	1.883945
H	2.705888	1.819140	2.722749
C	0.578643	1.961283	2.454655
H	-0.152373	1.830862	1.635335
H	0.354626	1.178910	3.196103

C	0.406694	3.340105	3.085954
H	-0.621913	3.456415	3.458376
H	1.067561	3.427091	3.964829
C	0.734424	4.451049	2.096874
H	0.612194	5.436905	2.567620
H	0.013352	4.409762	1.264212
C	2.140678	4.283327	1.538853
H	2.879069	4.401793	2.349942
H	2.361740	5.068743	0.802214
C	2.301302	2.909733	0.901562
H	3.309564	2.796723	0.475652
H	1.601787	2.813063	0.051381
C	4.032775	0.148616	0.627260
H	4.088086	0.771547	-0.284080
C	4.977231	0.778602	1.658490
H	4.947449	0.210190	2.600164
H	4.657176	1.799326	1.906742
C	6.412383	0.810724	1.139198
H	6.455179	1.461918	0.249391
H	7.076164	1.264222	1.888311
C	6.896347	-0.582845	0.760652
H	6.917518	-1.216143	1.663320
H	7.927728	-0.546756	0.384051
C	5.969233	-1.206293	-0.274217
H	6.306169	-2.217722	-0.542496
H	6.011963	-0.605802	-1.196056
C	4.527010	-1.244271	0.221070
H	3.861465	-1.667615	-0.548542
H	4.470971	-1.922575	1.088460
C	1.846195	-1.131663	2.288595
H	0.861992	-0.807183	2.673390
C	1.619375	-2.521097	1.675675
H	0.876124	-2.442852	0.865501
H	2.548212	-2.880139	1.206018
C	1.173690	-3.530309	2.728727
H	1.062096	-4.523991	2.273005
H	0.172516	-3.248039	3.092973
C	2.134843	-3.581586	3.910409
H	1.781515	-4.296650	4.666033
H	3.114917	-3.953103	3.567311
C	2.316749	-2.198865	4.524516
H	3.016267	-2.237557	5.371015
H	1.352464	-1.853268	4.933025
C	2.803676	-1.199159	3.479531
H	3.800584	-1.514073	3.130940
H	2.933825	-0.203159	3.929080
Al	-1.718371	-0.078582	-0.930896
N	-3.280607	1.095191	-0.684360
N	-2.911359	-1.646286	-1.057663
C	-4.500467	0.847127	-1.178256
C	-4.898317	-0.424526	-1.615148
H	-5.906912	-0.508588	-2.009036
C	-4.185359	-1.622431	-1.450918
C	-5.505208	1.960366	-1.259787
H	-5.689637	2.405780	-0.274372
H	-6.456526	1.610733	-1.669568
H	-5.128933	2.774429	-1.893048
C	-4.908052	-2.913118	-1.700075
H	-5.870000	-2.744817	-2.191889

H	-5.097701	-3.438344	-0.754464
H	-4.305110	-3.595693	-2.310870
C	-3.047761	2.368953	-0.064190
C	-3.225993	2.474693	1.333341
C	-3.078972	3.732688	1.922125
H	-3.225571	3.842343	2.995150
C	-2.729963	4.846849	1.167378
H	-2.618873	5.817929	1.647641
C	-2.473873	4.704343	-0.188446
H	-2.143263	5.565926	-0.769108
C	-2.615400	3.469165	-0.827453
C	-3.537662	1.252068	2.179562
H	-2.990594	0.407141	1.727201
C	-3.048974	1.392884	3.617113
H	-1.993874	1.687949	3.662868
H	-3.155103	0.441246	4.150449
H	-3.632249	2.135454	4.177226
C	-5.022321	0.881911	2.174085
H	-5.635390	1.720155	2.533070
H	-5.205682	0.028331	2.840970
H	-5.381782	0.598912	1.178525
C	-2.227121	3.342755	-2.286370
H	-2.530673	2.346057	-2.639308
C	-0.704228	3.429878	-2.414151
H	-0.387384	3.307000	-3.458672
H	-0.217941	2.642766	-1.815326
H	-0.336379	4.404384	-2.059870
C	-2.908421	4.381112	-3.174355
H	-4.001916	4.346509	-3.086731
H	-2.650419	4.216804	-4.227418
H	-2.592601	5.400981	-2.919382
C	-2.294987	-2.895763	-0.717875
C	-1.417683	-3.518650	-1.626443
C	-0.777202	-4.695430	-1.228131
H	-0.100039	-5.188781	-1.926063
C	-0.981657	-5.239711	0.032224
H	-0.469068	-6.154865	0.324713
C	-1.836529	-4.602552	0.923207
H	-1.979606	-5.016004	1.922491
C	-2.503424	-3.427772	0.571498
C	-1.145658	-2.966346	-3.010415
H	-1.711398	-2.030511	-3.123055
C	0.333125	-2.626254	-3.184367
H	0.962104	-3.526237	-3.134162
H	0.666285	-1.929733	-2.396075
H	0.506276	-2.149591	-4.158782
C	-1.615853	-3.930223	-4.099192
H	-1.073232	-4.883618	-4.047655
H	-1.442377	-3.506423	-5.096060
H	-2.685852	-4.157755	-4.012255
C	-3.371754	-2.731758	1.601711
H	-3.886800	-1.890756	1.115664
C	-2.502354	-2.152261	2.717309
H	-1.992242	-2.953102	3.269516
H	-3.112107	-1.590256	3.436842
H	-1.727770	-1.478555	2.320561
C	-4.439227	-3.655009	2.185307
H	-5.074625	-4.094573	1.406411
H	-5.089153	-3.106993	2.878997

H	-3.990636	-4.485114	2.746422
H	1.133411	0.805101	-2.997441
C	2.220524	0.931938	-2.985249
C	2.781076	2.157959	-2.618597
C	3.048589	-0.125782	-3.354747
C	4.164175	2.311908	-2.625699
H	2.136040	2.983740	-2.321713
C	4.440292	0.017784	-3.378276
H	2.606840	-1.083873	-3.630504
C	4.985328	1.250272	-3.005929
H	6.069374	1.375971	-3.010807
H	4.608637	3.262511	-2.331565
C	5.313505	-1.125501	-3.801787
H	5.155017	-1.383320	-4.857304
H	6.377183	-0.893464	-3.675759
H	5.100981	-2.032590	-3.220660

Int-2b-toluene_ortho1.log

SCF (wB97x) = -2687.86852279
H(0 K)= -2686.612420
H(298 K)= -2686.546602
G(298 K)= -2686.715121
Lowest Frequency = 8.1896cm-1

Pd	0.462784	-0.509879	-0.251274
P	2.667568	0.141222	0.142858
C	3.108434	1.682579	-0.809988
H	4.170830	1.924144	-0.630018
C	2.283992	2.907126	-0.407107
H	1.207828	2.672303	-0.510262
H	2.445935	3.152518	0.652585
C	2.646111	4.113408	-1.267882
H	2.054131	4.985449	-0.959061
H	3.702837	4.378065	-1.093509
C	2.442345	3.822195	-2.748871
H	2.710202	4.696688	-3.358496
H	1.371386	3.629293	-2.927149
C	3.246934	2.600688	-3.175553
H	4.323627	2.826216	-3.093928
H	3.061185	2.361667	-4.232611
C	2.916755	1.396106	-2.303182
H	3.516584	0.526193	-2.611318
H	1.861254	1.104748	-2.452798
C	4.084771	-0.997451	-0.335753
H	3.733851	-1.403668	-1.302341
C	5.472750	-0.396682	-0.584410
H	5.866605	0.050315	0.340857
H	5.418008	0.417584	-1.319894
C	6.448263	-1.460058	-1.083901
H	6.102119	-1.827415	-2.065378
H	7.439162	-1.016072	-1.252346
C	6.538518	-2.634822	-0.118791
H	6.951444	-2.283428	0.841520
H	7.237197	-3.393683	-0.496694
C	5.160756	-3.240305	0.115679
H	5.217298	-4.080569	0.821927
H	4.786489	-3.655494	-0.833691
C	4.173025	-2.192868	0.618227

H	3.173545	-2.634919	0.756458
H	4.502640	-1.844620	1.611413
C	2.940863	0.669312	1.911091
H	2.318955	1.579988	1.957769
C	2.309408	-0.304132	2.912013
H	1.281652	-0.527230	2.586731
H	2.850583	-1.263745	2.904516
C	2.324179	0.272757	4.323446
H	1.901456	-0.450141	5.035554
H	1.667138	1.157836	4.353740
C	3.729091	0.685132	4.748981
H	3.715904	1.123369	5.756301
H	4.367176	-0.212059	4.810241
C	4.340546	1.660184	3.749242
H	5.357051	1.943429	4.055637
H	3.747171	2.590612	3.740656
C	4.352387	1.068918	2.341755
H	5.007145	0.182043	2.333580
H	4.789882	1.783661	1.628448
Al	-1.755817	0.142762	0.289646
N	-2.723455	1.858985	0.433276
N	-3.381973	-0.828532	0.831594
C	-3.936101	2.070613	0.958689
C	-4.712284	1.044365	1.515211
H	-5.647500	1.342736	1.979850
C	-4.471653	-0.332432	1.424963
C	-4.524364	3.453040	0.950212
H	-3.944406	4.144417	1.574383
H	-5.555454	3.446913	1.313225
H	-4.509307	3.875128	-0.062130
C	-5.509211	-1.267613	1.973875
H	-6.351127	-0.722786	2.409481
H	-5.075865	-1.916902	2.745701
H	-5.889619	-1.938038	1.192677
C	-2.021725	2.972266	-0.140446
C	-1.381993	3.907518	0.701188
C	-0.717158	4.982846	0.110989
H	-0.213559	5.711709	0.745768
C	-0.670925	5.132053	-1.270812
H	-0.150644	5.980573	-1.713333
C	-1.262358	4.176973	-2.084204
H	-1.199378	4.276966	-3.168525
C	-1.935228	3.077763	-1.542485
C	-1.331053	3.713658	2.204522
H	-2.159034	3.053407	2.496583
C	-0.033354	2.998095	2.586078
H	0.085880	2.055644	2.028564
H	-0.010079	2.771987	3.660521
H	0.837493	3.628893	2.355137
C	-1.484521	5.013233	2.988609
H	-0.624710	5.680247	2.847539
H	-1.553504	4.805749	4.063047
H	-2.383481	5.569754	2.693587
C	-2.518422	2.041877	-2.483843
H	-3.013152	1.269039	-1.876202
C	-1.408086	1.357765	-3.283412
H	-1.817322	0.561795	-3.921422
H	-0.664292	0.906696	-2.606805
H	-0.885790	2.072211	-3.935904

C	-3.584850	2.631065	-3.403973
H	-4.408914	3.079437	-2.834844
H	-4.007487	1.852634	-4.052585
H	-3.168908	3.411207	-4.055237
C	-3.326820	-2.236530	0.557006
C	-3.686016	-2.672012	-0.739371
C	-3.655042	-4.042311	-1.005090
H	-3.931141	-4.401911	-1.994419
C	-3.242087	-4.954762	-0.039907
H	-3.220387	-6.018577	-0.269918
C	-2.817340	-4.499742	1.200134
H	-2.442656	-5.210767	1.937006
C	-2.844014	-3.138982	1.521447
C	-4.026332	-1.670071	-1.828996
H	-3.329004	-0.823385	-1.694076
C	-3.790838	-2.206040	-3.236296
H	-4.530313	-2.968722	-3.514376
H	-2.793269	-2.648415	-3.345423
H	-3.883531	-1.391678	-3.965740
C	-5.445415	-1.111697	-1.716598
H	-6.187673	-1.919326	-1.779318
H	-5.646829	-0.411233	-2.537929
H	-5.611922	-0.569196	-0.779749
C	-2.267731	-2.672930	2.842605
H	-2.504843	-1.605726	2.961503
C	-0.742593	-2.808289	2.810805
H	-0.448858	-3.863647	2.720983
H	-0.293385	-2.412759	3.731737
H	-0.307216	-2.264282	1.956555
C	-2.852500	-3.418655	4.039579
H	-3.947354	-3.349947	4.076563
H	-2.457924	-3.011758	4.978411
H	-2.593598	-4.485107	4.015597
H	-0.509586	-1.629105	-2.511887
C	0.233588	-2.362036	-2.198042
C	1.445928	-2.462503	-2.894687
C	-0.048298	-3.206896	-1.119005
C	2.367100	-3.431690	-2.482439
C	0.883317	-4.164117	-0.721852
H	-0.998635	-3.112302	-0.595808
C	2.089963	-4.277265	-1.410093
H	2.824988	-5.021336	-1.104278
H	3.321688	-3.515271	-3.006601
H	0.665599	-4.816636	0.122948
C	1.729994	-1.571004	-4.066034
H	2.801563	-1.352956	-4.160470
H	1.414527	-2.032956	-5.012274
H	1.199424	-0.614348	-3.980223

Int-2b-toluene_ortho2.log

SCF (wB97x) = -2687.87046957
H(0 K)= -2686.614098
H(298 K)= -2686.548548
G(298 K)= -2686.715959
Lowest Frequency = 11.2965cm-1

Pd	0.462414	-0.238147	0.416958
P	2.750668	0.158874	0.205540

C	3.073565	1.357976	-1.182084
H	4.163404	1.505376	-1.284876
C	2.433832	2.728457	-0.947065
H	1.346701	2.593385	-0.794509
H	2.819189	3.193170	-0.027659
C	2.687482	3.655727	-2.131075
H	2.231986	4.637346	-1.942559
H	3.772255	3.830582	-2.229046
C	2.154124	3.059042	-3.426625
H	2.353366	3.728026	-4.275649
H	1.058367	2.971675	-3.343520
C	2.745786	1.677646	-3.678016
H	3.827211	1.773669	-3.873671
H	2.308473	1.228200	-4.581741
C	2.529546	0.757330	-2.483272
H	2.972891	-0.230707	-2.677063
H	1.449482	0.576209	-2.336656
C	3.874133	-1.272656	-0.256693
H	3.252227	-1.838933	-0.975037
C	5.195476	-0.948207	-0.962733
H	5.840317	-0.345933	-0.304363
H	5.016540	-0.339613	-1.859075
C	5.933315	-2.224091	-1.358577
H	5.319501	-2.776383	-2.089972
H	6.875341	-1.974332	-1.866200
C	6.189840	-3.113541	-0.149420
H	6.853666	-2.584205	0.554797
H	6.720599	-4.028600	-0.445874
C	4.880241	-3.456880	0.548588
H	5.062632	-4.079084	1.435842
H	4.258489	-4.055383	-0.134308
C	4.111973	-2.198801	0.941403
H	3.149115	-2.461367	1.407491
H	4.690639	-1.660036	1.709883
C	3.486374	1.032944	1.679383
H	2.981670	2.013709	1.620108
C	3.027899	0.420925	3.008208
H	1.936433	0.272305	2.978794
H	3.471156	-0.578922	3.137252
C	3.433551	1.305520	4.182391
H	3.120800	0.847988	5.131063
H	2.896615	2.265989	4.109029
C	4.934325	1.575282	4.186185
H	5.208561	2.228749	5.025553
H	5.472116	0.625861	4.344748
C	5.384787	2.186598	2.864476
H	6.469602	2.361078	2.867213
H	4.911536	3.175345	2.743275
C	4.992056	1.302072	1.683777
H	5.537140	0.346773	1.756541
H	5.306864	1.766038	0.736946
Al	-1.865261	0.248434	0.542860
N	-2.766998	1.971428	0.322430
N	-3.594354	-0.661844	0.716787
C	-4.039085	2.261243	0.616776
C	-4.963655	1.286868	1.016798
H	-5.959496	1.638323	1.269849
C	-4.775244	-0.101322	1.001575
C	-4.524071	3.675679	0.490921

H	-3.894497	4.362722	1.069902
H	-5.559137	3.774895	0.828057
H	-4.462733	4.018050	-0.549911
C	-5.964731	-0.972516	1.281951
H	-6.872720	-0.378048	1.413211
H	-5.809390	-1.571428	2.188664
H	-6.126165	-1.690243	0.467952
C	-1.951483	3.005122	-0.255992
C	-1.180325	3.847901	0.566071
C	-0.437260	4.863175	-0.042234
H	0.163885	5.525614	0.581627
C	-0.443198	5.034904	-1.420237
H	0.128703	5.842284	-1.875029
C	-1.159667	4.153082	-2.219907
H	-1.128168	4.262175	-3.304333
C	-1.914007	3.119681	-1.660370
C	-1.091440	3.656320	2.066388
H	-1.816977	2.882073	2.354334
C	0.300749	3.146675	2.446453
H	0.541669	2.217345	1.903686
H	0.360572	2.941034	3.523729
H	1.070826	3.894016	2.203873
C	-1.435249	4.927903	2.839004
H	-0.715371	5.730578	2.632451
H	-1.412056	4.741848	3.919625
H	-2.432441	5.310034	2.585403
C	-2.604390	2.120465	-2.569760
H	-3.273291	1.498924	-1.955358
C	-1.562690	1.190869	-3.199565
H	-2.044460	0.425009	-3.823098
H	-0.961846	0.681210	-2.428554
H	-0.867842	1.754402	-3.839161
C	-3.459928	2.787088	-3.643557
H	-4.213782	3.455344	-3.209529
H	-3.984297	2.032755	-4.243094
H	-2.851095	3.382495	-4.336040
C	-3.519538	-2.089261	0.574137
C	-3.464377	-2.631312	-0.730171
C	-3.387563	-4.020595	-0.858546
H	-3.342793	-4.464704	-1.850422
C	-3.349089	-4.848460	0.257666
H	-3.288658	-5.928105	0.131863
C	-3.352587	-4.293366	1.529158
H	-3.276163	-4.942652	2.401183
C	-3.426044	-2.910165	1.713761
C	-3.472037	-1.734106	-1.957490
H	-2.784817	-0.892984	-1.743621
C	-2.951663	-2.423617	-3.213420
H	-3.651108	-3.190250	-3.572811
H	-1.977543	-2.899768	-3.053264
H	-2.839133	-1.693050	-4.023630
C	-4.850140	-1.132273	-2.241785
H	-5.595391	-1.925048	-2.393547
H	-4.819247	-0.528261	-3.158461
H	-5.204601	-0.481389	-1.435225
C	-3.301117	-2.332034	3.109407
H	-3.612599	-1.278293	3.076576
C	-1.833406	-2.363072	3.544253
H	-1.467990	-3.398261	3.594543

H	-1.707797	-1.912090	4.537027
H	-1.190291	-1.819368	2.836024
C	-4.184448	-3.042413	4.131513
H	-5.236744	-3.070798	3.820225
H	-4.133430	-2.534726	5.102065
H	-3.864340	-4.079228	4.295049
H	-0.386560	-1.861068	-1.479097
C	0.418481	-2.549500	-1.744348
C	1.060675	-2.442885	-2.976171
C	0.794143	-3.531987	-0.817501
C	2.095310	-3.316944	-3.307178
C	1.816477	-4.415469	-1.173831
C	2.466744	-4.309947	-2.402766
H	3.267441	-5.005778	-2.652929
H	2.600275	-3.231679	-4.268466
H	2.114505	-5.193279	-0.468720
H	0.750259	-1.668177	-3.679110
C	0.147799	-3.590741	0.531119
H	0.161766	-4.603756	0.954472
H	0.667163	-2.921235	1.238065
H	-0.891714	-3.242490	0.496478

Int-2b-toluene_para.log

SCF (wB97x) = -2687.86615618
H(0 K)= -2686.610150
H(298 K)= -2686.544112
G(298 K)= -2686.714100
Lowest Frequency = 8.8271cm-1

Pd	0.387922	-0.282166	-0.203697
P	2.405026	0.519436	0.629053
C	2.945929	2.045311	-0.292392
H	3.945484	2.354214	0.062678
C	1.985850	3.220441	-0.088093
H	0.965621	2.901433	-0.371998
H	1.935648	3.507042	0.972712
C	2.406066	4.426351	-0.921445
H	1.706177	5.256762	-0.755503
H	3.393069	4.779671	-0.578212
C	2.484111	4.077765	-2.401761
H	2.795330	4.950785	-2.992407
H	1.475168	3.802532	-2.751152
C	3.427919	2.904472	-2.634822
H	4.458033	3.208935	-2.383872
H	3.440187	2.622473	-3.697825
C	3.032806	1.703806	-1.784425
H	3.726437	0.866805	-1.956127
H	2.039665	1.335513	-2.098479
C	3.879504	-0.616292	0.412036
H	3.676326	-1.067496	-0.577317
C	5.281487	-0.003401	0.343858
H	5.538769	0.466456	1.306055
H	5.323340	0.794177	-0.410185
C	6.316823	-1.074055	0.008807
H	6.093832	-1.476636	-0.994258
H	7.320230	-0.630050	-0.049940
C	6.290460	-2.210627	1.023273
H	6.589257	-1.819062	2.009969

H	7.029674	-2.980566	0.760781
C	4.897476	-2.819840	1.124422
H	4.874237	-3.610741	1.887010
H	4.643030	-3.299810	0.167353
C	3.842794	-1.762301	1.428614
H	2.837731	-2.211830	1.450662
H	4.027139	-1.358153	2.437858
C	2.367854	1.101071	2.399057
H	1.716546	1.989443	2.319639
C	1.618178	0.129499	3.316484
H	0.673049	-0.160109	2.833119
H	2.196703	-0.799207	3.439727
C	1.374582	0.752223	4.686432
H	0.855503	0.039674	5.343099
H	0.699858	1.617222	4.572021
C	2.678719	1.219268	5.324397
H	2.488229	1.687101	6.299943
H	3.318227	0.342852	5.521230
C	3.425604	2.182383	4.408002
H	4.372660	2.498048	4.867091
H	2.823495	3.097386	4.277519
C	3.679709	1.559861	3.036937
H	4.353595	0.695397	3.155300
H	4.203954	2.273237	2.383333
Al	-1.978955	-0.182720	-0.319329
N	-3.111546	1.404151	-0.527192
N	-3.539809	-1.278161	0.151509
C	-4.445003	1.480570	-0.442616
C	-5.250730	0.382459	-0.114992
H	-6.320499	0.564547	-0.070290
C	-4.824231	-0.912420	0.206526
C	-5.128493	2.796201	-0.679025
H	-4.871347	3.517025	0.108176
H	-6.215624	2.682608	-0.697409
H	-4.800791	3.251203	-1.621425
C	-5.865448	-1.900225	0.649844
H	-6.873354	-1.542760	0.420946
H	-5.804819	-2.063335	1.734424
H	-5.718142	-2.881327	0.184741
C	-2.379599	2.615274	-0.779698
C	-2.093212	3.483317	0.294116
C	-1.417207	4.674079	0.021112
H	-1.185116	5.354701	0.840612
C	-1.023868	4.998545	-1.271965
H	-0.512894	5.939567	-1.470559
C	-1.256889	4.101335	-2.304966
H	-0.911967	4.336977	-3.312463
C	-1.920866	2.892056	-2.081101
C	-2.422235	3.116120	1.728736
H	-3.130238	2.273967	1.718673
C	-1.152604	2.634058	2.436595
H	-0.684864	1.794718	1.895477
H	-1.374519	2.306802	3.461296
H	-0.410860	3.444579	2.495500
C	-3.074628	4.257484	2.503924
H	-2.389184	5.104802	2.633172
H	-3.365334	3.924062	3.507400
H	-3.973640	4.637156	2.001811
C	-2.078407	1.916900	-3.230504

H	-2.648650	1.048575	-2.867978
C	-0.704706	1.410734	-3.675428
H	-0.801126	0.636199	-4.448550
H	-0.157582	0.980831	-2.821377
H	-0.094999	2.226404	-4.091227
C	-2.853709	2.518845	-4.399979
H	-3.854136	2.852702	-4.097668
H	-2.973188	1.784683	-5.206135
H	-2.330915	3.386639	-4.823088
C	-3.171019	-2.609970	0.538456
C	-2.838834	-3.541367	-0.468202
C	-2.427387	-4.815299	-0.069502
H	-2.167596	-5.553960	-0.825352
C	-2.332889	-5.153878	1.275313
H	-2.010390	-6.153086	1.563330
C	-2.635296	-4.212608	2.250473
H	-2.534476	-4.477964	3.302236
C	-3.052311	-2.924856	1.906005
C	-2.932986	-3.171433	-1.937751
H	-2.561197	-2.135316	-2.032778
C	-2.058724	-4.032722	-2.840403
H	-2.436708	-5.060943	-2.917983
H	-1.021778	-4.077595	-2.487804
H	-2.046101	-3.618616	-3.855433
C	-4.378125	-3.183816	-2.438366
H	-4.834922	-4.171702	-2.288599
H	-4.416412	-2.958902	-3.511668
H	-5.001085	-2.441984	-1.926311
C	-3.279915	-1.881028	2.984300
H	-3.913173	-1.084104	2.568535
C	-1.949557	-1.232367	3.372519
H	-1.260667	-1.975961	3.796853
H	-2.096953	-0.441062	4.120139
H	-1.444553	-0.790815	2.499201
C	-3.993985	-2.434986	4.213420
H	-4.926086	-2.951087	3.950050
H	-4.240137	-1.625071	4.910365
H	-3.367210	-3.148482	4.763072
H	0.386441	-1.296818	-2.552431
C	1.349757	-1.807214	-2.562512
C	2.379272	-1.381942	-3.398384
C	1.557628	-2.901896	-1.718849
C	3.600464	-2.055422	-3.404474
H	2.232350	-0.515091	-4.043453
C	2.772799	-3.578485	-1.740541
H	0.761424	-3.211290	-1.038431
C	3.812371	-3.171839	-2.590173
H	4.403388	-1.717398	-4.061441
H	2.925378	-4.437765	-1.085407
C	5.107445	-3.926865	-2.638069
H	4.980573	-4.915695	-3.099177
H	5.865838	-3.390707	-3.219890
H	5.521263	-4.101499	-1.635509

Int-2c_cc-PdP2--HPh.log

SCF (wB97x) = -2454.38727320
H(0 K)= -2453.311967
H(298 K)= -2453.263411

G(298 K)= -2453.396863
Lowest Frequency = 10.2098cm-1

Pd	-0.116869	0.309350	-0.467925
P	2.163128	0.428686	-0.059944
P	-2.402690	0.250592	-0.199195
C	-2.723999	0.292792	1.642477
C	-4.110455	0.648328	2.190127
C	-2.198867	-1.010690	2.261824
H	-2.035428	1.091277	1.973218
C	-4.095286	0.694889	3.717198
H	-4.860200	-0.082847	1.853925
H	-4.437104	1.621243	1.800040
C	-2.197146	-0.946194	3.783853
H	-2.833050	-1.854765	1.940478
H	-1.187796	-1.216418	1.871831
C	-3.583380	-0.607552	4.319548
H	-5.099182	0.929602	4.096394
H	-3.441595	1.521249	4.041434
H	-1.837397	-1.895464	4.204488
H	-1.482410	-0.172078	4.109091
H	-3.571569	-0.545723	5.416020
H	-4.279212	-1.423628	4.062809
C	-3.208161	-1.310976	-0.832137
C	-4.600031	-1.732353	-0.349227
C	-3.121027	-1.336816	-2.363652
H	-2.508344	-2.084493	-0.465234
C	-4.983588	-3.093807	-0.926227
H	-5.353992	-0.986331	-0.639046
H	-4.624401	-1.782718	0.746774
C	-3.521631	-2.695994	-2.925826
H	-3.786243	-0.561994	-2.781133
H	-2.101024	-1.065766	-2.680724
C	-4.910179	-3.102109	-2.447688
H	-5.987309	-3.379370	-0.583284
H	-4.291993	-3.856000	-0.529203
H	-3.476375	-2.681528	-4.023073
H	-2.789019	-3.450621	-2.594472
H	-5.180976	-4.090849	-2.841367
H	-5.653665	-2.394026	-2.849818
C	-3.307680	1.694292	-0.960598
C	-4.834100	1.661515	-1.089481
C	-2.823612	2.985389	-0.284927
H	-2.900410	1.707651	-1.987610
C	-5.358103	2.922751	-1.773572
H	-5.303571	1.560566	-0.099923
H	-5.144517	0.780504	-1.666634
C	-3.370139	4.226565	-0.979518
H	-3.149224	2.994377	0.768664
H	-1.722192	2.993844	-0.265875
C	-4.890953	4.186311	-1.063058
H	-6.454639	2.890425	-1.831005
H	-4.994741	2.942220	-2.814218
H	-3.032902	5.131477	-0.456191
H	-2.953070	4.283572	-1.997972
H	-5.275699	5.079601	-1.573145
H	-5.311028	4.206271	-0.043565
C	2.546739	-0.830631	1.261076
C	3.995275	-1.266632	1.499064

C	1.858134	-0.436946	2.572560
H	2.011813	-1.719140	0.879300
C	4.058514	-2.382350	2.540926
H	4.607149	-0.415637	1.837530
H	4.444849	-1.619105	0.558834
C	1.928132	-1.566142	3.592703
H	2.344499	0.459435	2.994049
H	0.813422	-0.152752	2.362997
C	3.372758	-1.984923	3.842420
H	5.103172	-2.669250	2.724768
H	3.556074	-3.273297	2.130735
H	1.443425	-1.265140	4.531925
H	1.358352	-2.429375	3.208407
H	3.418336	-2.809334	4.566988
H	3.917818	-1.141747	4.298889
C	2.598298	2.098628	0.651738
C	3.919668	2.318620	1.395155
C	2.384267	3.183784	-0.410827
H	1.789333	2.226905	1.394404
C	3.962007	3.716076	2.010447
H	4.769578	2.203938	0.707812
H	4.052194	1.563188	2.181146
C	2.448328	4.579129	0.199306
H	3.162760	3.092394	-1.187952
H	1.419144	3.019181	-0.914884
C	3.753550	4.796232	0.955805
H	4.913511	3.868772	2.537933
H	3.168987	3.796178	2.772352
H	2.321334	5.342179	-0.580668
H	1.600922	4.701676	0.893753
H	3.773180	5.793421	1.415893
H	4.593402	4.768426	0.241886
C	3.360661	0.014101	-1.428644
C	2.771155	0.366411	-2.800020
C	4.802486	0.520842	-1.335634
H	3.394976	-1.089136	-1.372774
C	3.621205	-0.209335	-3.927379
H	2.714561	1.463432	-2.901683
H	1.731453	0.009332	-2.859071
C	5.654748	-0.071838	-2.455041
H	4.807463	1.619740	-1.425535
H	5.244005	0.289002	-0.358176
C	5.071703	0.249254	-3.825730
H	3.197654	0.061010	-4.904206
H	3.589219	-1.311437	-3.870672
H	6.689103	0.290750	-2.380049
H	5.699902	-1.166837	-2.324995
H	5.678345	-0.206026	-4.620129
H	5.116985	1.338624	-3.987812
H	-0.560500	-2.264732	-1.454471
C	0.240936	-2.896691	-1.079886
C	0.072694	-3.628642	0.095681
C	1.452512	-2.956024	-1.767389
C	1.115752	-4.414435	0.582877
C	2.496967	-3.738538	-1.278868
C	2.328695	-4.470765	-0.103971
H	1.581548	-2.374659	-2.680258
H	3.445485	-3.775727	-1.815919
H	3.143638	-5.083857	0.278255

H	0.984966	-4.983917	1.502427
H	-0.873441	-3.578115	0.636001

Int-3a_p1-PdAl2.log

SCF (wB97x) = -2842.69876690
H(0 K)= -2841.319241
H(298 K)= -2841.241048
G(298 K)= -2841.429732
Lowest Frequency = 14.6418cm-1

Pd	-0.176262	-0.413391	1.334739
Al	-1.487511	0.246926	-0.447890
N	-2.542600	1.865376	-0.177131
N	-2.334711	-0.128733	-2.156828
C	-3.189094	2.540248	-1.131953
C	-3.367175	2.034311	-2.427805
H	-3.920122	2.663008	-3.119362
C	-3.079140	0.739206	-2.863647
C	-3.822676	3.864200	-0.819353
H	-4.561997	3.765729	-0.014622
H	-4.315768	4.284631	-1.699804
H	-3.076917	4.580611	-0.455024
C	-3.720194	0.303623	-4.151067
H	-3.076396	-0.343611	-4.754179
H	-4.022225	1.169836	-4.746610
H	-4.626056	-0.280613	-3.935597
C	-2.679509	2.363590	1.172177
C	-3.719521	1.839963	1.968369
C	-3.951020	2.429554	3.211887
H	-4.742957	2.038249	3.847381
C	-3.163485	3.477943	3.672490
H	-3.364062	3.924370	4.645229
C	-2.089095	3.914219	2.911577
H	-1.432314	4.694604	3.297406
C	-1.818632	3.360942	1.656182
C	-4.538237	0.645080	1.515259
H	-3.856741	-0.001966	0.935298
C	-5.045913	-0.189973	2.685055
H	-4.241377	-0.430239	3.389571
H	-5.469093	-1.133788	2.320366
H	-5.846632	0.324350	3.233891
C	-5.700303	1.033704	0.599028
H	-6.376978	1.736225	1.105512
H	-6.289492	0.145672	0.330954
H	-5.367925	1.500044	-0.336748
C	-0.572222	3.791778	0.913853
H	-0.545608	3.268286	-0.053362
C	0.657353	3.352756	1.707486
H	1.576143	3.611691	1.168496
H	0.649301	2.266955	1.883713
H	0.690128	3.850277	2.687246
C	-0.518381	5.292151	0.635593
H	-1.369666	5.640538	0.036249
H	0.400425	5.543464	0.090094
H	-0.516094	5.872247	1.568051
C	-2.251311	-1.471456	-2.666848
C	-1.128330	-1.854130	-3.425029
C	-1.115283	-3.138660	-3.976847

H	-0.264066	-3.448871	-4.582570
C	-2.172783	-4.018243	-3.781753
H	-2.144974	-5.011824	-4.225916
C	-3.259733	-3.630373	-3.007848
H	-4.075509	-4.332018	-2.837537
C	-3.320341	-2.359511	-2.431832
C	0.000309	-0.885631	-3.719206
H	-0.047155	-0.082603	-2.968045
C	1.379339	-1.521305	-3.602597
H	1.532625	-2.333828	-4.325587
H	1.552378	-1.931933	-2.599869
H	2.160218	-0.773340	-3.793603
C	-0.165766	-0.241165	-5.095886
H	-0.187054	-1.001935	-5.888156
H	0.674275	0.434408	-5.306612
H	-1.089155	0.345564	-5.172372
C	-4.483879	-1.989898	-1.532303
H	-4.517086	-0.894002	-1.437394
C	-4.257510	-2.561491	-0.130527
H	-4.170902	-3.656314	-0.164901
H	-5.095039	-2.308868	0.532395
H	-3.337376	-2.173481	0.330921
C	-5.832649	-2.441893	-2.085796
H	-5.992177	-2.100079	-3.116440
H	-6.649500	-2.048045	-1.469488
H	-5.930241	-3.534913	-2.083106
Al	1.782706	-0.161696	-0.076443
N	2.818730	-1.827721	-0.161468
N	3.051251	0.827678	-1.150196
C	3.842227	-2.063331	-0.972228
C	4.310026	-1.103686	-1.891277
H	5.088059	-1.433522	-2.574196
C	3.994720	0.254351	-1.924060
C	4.604085	-3.354963	-0.905912
H	4.039707	-4.153500	-0.416859
H	5.534004	-3.211518	-0.340303
H	4.892752	-3.678991	-1.911835
C	4.773058	1.114362	-2.878229
H	4.109466	1.529589	-3.648593
H	5.558203	0.540283	-3.376784
H	5.232329	1.972876	-2.374185
C	2.527247	-2.727137	0.922821
C	3.322575	-2.690252	2.084111
C	2.957377	-3.507086	3.157434
H	3.557374	-3.482886	4.067501
C	1.840407	-4.326102	3.093590
H	1.564491	-4.943709	3.946296
C	1.064179	-4.343142	1.941427
H	0.180018	-4.976083	1.902879
C	1.382105	-3.544505	0.841474
C	4.527283	-1.782778	2.241116
H	4.671253	-1.218839	1.306785
C	4.303186	-0.767251	3.361184
H	4.155365	-1.271310	4.325114
H	3.418110	-0.148107	3.170140
H	5.177717	-0.110982	3.465254
C	5.803164	-2.581104	2.513193
H	6.679768	-1.920818	2.530575
H	5.978106	-3.359867	1.761114

H	5.752408	-3.081416	3.489186
C	0.551247	-3.598112	-0.424025
H	0.531633	-2.580820	-0.849444
C	1.184383	-4.527303	-1.459901
H	2.182270	-4.186909	-1.764695
H	0.560615	-4.577847	-2.362139
H	1.286294	-5.546119	-1.060679
C	-0.899219	-3.975674	-0.172212
H	-1.478174	-3.867943	-1.095992
H	-1.342421	-3.317970	0.587073
H	-1.011191	-5.017103	0.159931
C	3.047258	2.264208	-1.062890
C	2.321080	3.046942	-1.981127
C	2.371577	4.438709	-1.852492
H	1.802382	5.055985	-2.548309
C	3.143800	5.045234	-0.872998
H	3.185453	6.131016	-0.802172
C	3.851381	4.258971	0.028052
H	4.435064	4.740059	0.810416
C	3.798664	2.864275	-0.025995
C	1.501707	2.452718	-3.110189
H	1.695587	1.369208	-3.139410
C	0.005830	2.649065	-2.870903
H	-0.250187	3.713892	-2.765714
H	-0.315655	2.146090	-1.947797
H	-0.588400	2.241620	-3.699591
C	1.887980	3.045995	-4.466180
H	1.346099	2.544854	-5.277976
H	2.962033	2.957930	-4.670375
H	1.634937	4.112357	-4.522066
C	4.524190	2.033004	1.018645
H	3.949062	1.103845	1.157432
C	4.580526	2.726184	2.376478
H	5.239378	3.604182	2.366909
H	4.976130	2.041147	3.133984
H	3.585975	3.049985	2.706477
C	5.930389	1.633467	0.567664
H	6.541067	2.519865	0.346433
H	5.916357	0.999058	-0.326831
H	6.439104	1.068066	1.360143
H	1.799288	0.130331	1.572587
C	-1.296635	-0.821412	3.040777
C	-1.523157	0.197741	3.986460
C	-1.800026	-2.093912	3.370440
C	-2.191892	-0.037466	5.189031
H	-1.177343	1.213815	3.774857
C	-2.462661	-2.343997	4.573786
H	-1.663681	-2.926190	2.673722
C	-2.662783	-1.314364	5.492390
H	-2.350388	0.784352	5.890703
H	-2.831015	-3.347854	4.793469
H	-3.184979	-1.503939	6.430124

Int-3b_p1-PPdAl.log

SCF (wB97x) = -2648.53803854
H(0 K)= -2647.312763
H(298 K)= -2647.249725
G(298 K)= -2647.408844

Lowest Frequency = 20.8571cm-1

Pd	0.863187	0.135809	-1.013540
P	2.151331	-0.005910	0.890386
C	2.084523	1.566136	1.877575
H	2.771624	1.452713	2.734958
C	0.694716	1.893129	2.430431
H	-0.017225	1.963535	1.588611
H	0.328029	1.079321	3.075353
C	0.706084	3.209281	3.203329
H	-0.306804	3.438615	3.565548
H	1.340754	3.101774	4.098814
C	1.228302	4.356033	2.346844
H	1.248065	5.290493	2.924795
H	0.530058	4.519515	1.510119
C	2.605662	4.036909	1.781630
H	3.336532	3.958189	2.604104
H	2.956189	4.851519	1.133433
C	2.577591	2.726982	1.005227
H	3.565070	2.510595	0.573091
H	1.888859	2.820013	0.147897
C	3.975543	-0.208258	0.568833
H	4.111563	0.453622	-0.304102
C	4.982299	0.243380	1.632200
H	4.924745	-0.405280	2.519701
H	4.763178	1.262480	1.979134
C	6.397564	0.194913	1.058952
H	6.465989	0.918110	0.229390
H	7.124037	0.519336	1.816649
C	6.746531	-1.194905	0.539469
H	6.766168	-1.900928	1.386704
H	7.757578	-1.200680	0.110483
C	5.725800	-1.671272	-0.487417
H	5.963281	-2.689683	-0.824603
H	5.767197	-1.027787	-1.380584
C	4.312019	-1.620695	0.079724
H	3.581307	-1.942739	-0.677027
H	4.234087	-2.329989	0.921230
C	1.603568	-1.322243	2.087793
H	0.639237	-0.920964	2.450222
C	1.278915	-2.655194	1.399694
H	0.562509	-2.477878	0.581025
H	2.185372	-3.069261	0.932752
C	0.717243	-3.671551	2.389171
H	0.544767	-4.628681	1.879406
H	-0.273074	-3.329489	2.731095
C	1.626104	-3.857168	3.597799
H	1.185764	-4.573250	4.304907
H	2.584359	-4.294254	3.271234
C	1.898617	-2.524362	4.282992
H	2.558561	-2.657984	5.150913
H	0.951323	-2.113432	4.671128
C	2.511335	-1.529691	3.302404
H	3.484622	-1.920960	2.966412
H	2.718824	-0.571771	3.802224
Al	-1.488859	-0.017501	-0.649002
N	-2.860432	1.360490	-0.701223
N	-2.751536	-1.423733	-1.084766
C	-4.011693	1.249341	-1.376359

C	-4.463303	0.038854	-1.921498
H	-5.400051	0.077146	-2.469041
C	-3.922699	-1.234720	-1.700161
C	-4.896566	2.450974	-1.534273
H	-5.141382	2.890912	-0.559329
H	-5.826560	2.194701	-2.047864
H	-4.390108	3.239566	-2.104769
C	-4.720494	-2.424706	-2.142911
H	-5.611005	-2.124507	-2.700794
H	-5.036920	-3.026223	-1.281261
H	-4.115748	-3.092053	-2.769402
C	-2.582002	2.605193	-0.035856
C	-2.836026	2.696326	1.350466
C	-2.635766	3.929096	1.975864
H	-2.835483	4.028421	3.041032
C	-2.172314	5.031013	1.266414
H	-2.021825	5.982196	1.774837
C	-1.861210	4.901319	-0.079198
H	-1.449184	5.751667	-0.622550
C	-2.050114	3.692061	-0.754366
C	-3.294191	1.487082	2.146502
H	-2.741165	0.615291	1.749688
C	-2.957472	1.590456	3.629456
H	-1.901134	1.836398	3.792836
H	-3.162848	0.639319	4.133637
H	-3.563423	2.354490	4.133650
C	-4.785889	1.196535	1.970881
H	-5.389195	2.059420	2.284634
H	-5.085736	0.340335	2.590557
H	-5.048647	0.957508	0.933815
C	-1.599261	3.567429	-2.195101
H	-1.976249	2.616220	-2.597508
C	-0.071924	3.505849	-2.235889
H	0.295628	3.351363	-3.257957
H	0.304635	2.670497	-1.627214
H	0.367502	4.437223	-1.848463
C	-2.116381	4.695568	-3.084688
H	-3.209553	4.789548	-3.050166
H	-1.827108	4.522190	-4.127746
H	-1.696675	5.666465	-2.791589
C	-2.385775	-2.755629	-0.687547
C	-1.577521	-3.548510	-1.522365
C	-1.229818	-4.828779	-1.081118
H	-0.603442	-5.453451	-1.718376
C	-1.663162	-5.313444	0.145326
H	-1.383253	-6.314791	0.468901
C	-2.442796	-4.507641	0.965854
H	-2.762858	-4.878936	1.940349
C	-2.813429	-3.220299	0.573101
C	-1.057444	-3.057880	-2.856826
H	-1.457455	-2.049584	-3.034277
C	0.465107	-2.944403	-2.827947
H	0.935367	-3.926101	-2.673477
H	0.796429	-2.273242	-2.020421
H	0.841074	-2.530212	-3.771412
C	-1.507332	-3.955233	-4.009047
H	-1.097899	-4.969208	-3.909225
H	-1.158247	-3.554522	-4.968154
H	-2.599663	-4.048643	-4.062050

C	-3.634404	-2.363311	1.517715
H	-3.862817	-1.412484	1.014622
C	-2.835998	-2.032889	2.778116
H	-2.563911	-2.944591	3.327381
H	-3.424540	-1.401571	3.456949
H	-1.903783	-1.499330	2.538063
C	-4.962821	-3.022994	1.883100
H	-5.563058	-3.254434	0.994699
H	-5.559703	-2.365977	2.528186
H	-4.807497	-3.963409	2.427793
H	-0.373456	0.237142	-2.205661
C	2.504956	0.477556	-2.315365
C	3.050121	1.765074	-2.472009
C	3.177880	-0.558641	-2.985099
C	4.197476	2.004435	-3.231572
H	2.576698	2.618720	-1.981139
C	4.323587	-0.332952	-3.752029
H	2.812707	-1.584663	-2.903628
C	4.845828	0.952461	-3.875021
H	4.586395	3.020370	-3.319797
H	4.814854	-1.171072	-4.249838
H	5.742697	1.132614	-4.466741

Int-4a_p1-PdAl2-tra.log

SCF (wB97x) = -2842.69110956
H(0 K)= -2841.314091
H(298 K)= -2841.234819
G(298 K)= -2841.429482
Lowest Frequency = 14.9232cm-1

Pd	-0.060423	-0.063141	-0.034639
Al	2.319434	0.393671	0.373537
N	3.257992	2.011082	-0.200316
N	3.966573	-0.322525	1.172746
C	4.422743	2.469630	0.268006
C	5.257288	1.704979	1.095520
H	6.185724	2.172198	1.410093
C	5.086838	0.359874	1.443002
C	4.897529	3.836336	-0.130406
H	4.957523	3.928319	-1.222198
H	5.878985	4.058383	0.296933
H	4.189129	4.606987	0.199718
C	6.229743	-0.333628	2.124967
H	5.934010	-0.719492	3.108755
H	7.081932	0.338025	2.256920
H	6.556520	-1.205741	1.544500
C	2.586233	2.781594	-1.214519
C	2.771349	2.404109	-2.561355
C	2.189530	3.200788	-3.548558
H	2.312675	2.929303	-4.595013
C	1.410621	4.303713	-3.218529
H	0.960155	4.906605	-4.005799
C	1.167527	4.603194	-1.885420
H	0.510846	5.434850	-1.628665
C	1.745388	3.850091	-0.859148
C	3.533906	1.140621	-2.920836
H	3.253942	0.381445	-2.166182
C	3.134034	0.569090	-4.274645

H	2.047707	0.451833	-4.361955
H	3.595470	-0.416485	-4.415045
H	3.482938	1.202244	-5.102036
C	5.051933	1.317607	-2.855316
H	5.380145	2.104809	-3.548275
H	5.557210	0.387680	-3.149427
H	5.409274	1.578784	-1.852626
C	1.388185	4.143519	0.583278
H	2.088520	3.593686	1.229184
C	-0.021147	3.622719	0.873947
H	-0.294253	3.775655	1.926750
H	-0.111908	2.546515	0.648897
H	-0.764065	4.150512	0.259206
C	1.498677	5.624672	0.936192
H	2.491556	6.033451	0.707196
H	1.308142	5.781021	2.004932
H	0.763244	6.226518	0.386930
C	3.930183	-1.726984	1.478907
C	3.625411	-2.157410	2.785672
C	3.591909	-3.530350	3.038960
H	3.355419	-3.878488	4.044621
C	3.837760	-4.456009	2.032615
H	3.804599	-5.522413	2.250191
C	4.106174	-4.014600	0.744259
H	4.275708	-4.741244	-0.050633
C	4.157672	-2.651627	0.440004
C	3.275105	-1.182583	3.892202
H	3.562240	-0.173364	3.564972
C	1.762537	-1.176373	4.123953
H	1.420496	-2.164704	4.462808
H	1.209665	-0.929649	3.206468
H	1.489900	-0.444526	4.895222
C	4.017030	-1.475093	5.194387
H	3.705564	-2.431799	5.632857
H	3.808028	-0.697422	5.938848
H	5.104328	-1.520749	5.051192
C	4.412847	-2.215897	-0.990585
H	4.599600	-1.131048	-0.989332
C	3.173180	-2.474129	-1.850104
H	2.918659	-3.543807	-1.863130
H	3.334667	-2.154128	-2.888032
H	2.294002	-1.932032	-1.472018
C	5.645610	-2.882894	-1.595466
H	6.544095	-2.708159	-0.990657
H	5.835082	-2.493164	-2.603407
H	5.516059	-3.968848	-1.689275
Al	-2.290216	-0.433306	0.536482
N	-3.270878	-2.065115	0.285893
N	-3.733738	0.476588	1.420431
C	-4.448655	-2.364544	0.839564
C	-5.157893	-1.457417	1.639913
H	-6.093389	-1.815528	2.059133
C	-4.861456	-0.104984	1.854460
C	-5.082209	-3.693019	0.554446
H	-5.482018	-3.713726	-0.468414
H	-5.906920	-3.895683	1.243070
H	-4.351015	-4.507323	0.612041
C	-5.882841	0.719164	2.581486
H	-5.497746	1.060940	3.550208

H	-6.798062	0.148650	2.758530
H	-6.132796	1.625462	2.016256
C	-2.714962	-2.942904	-0.711395
C	-3.238771	-2.900055	-2.019787
C	-2.667953	-3.732249	-2.983685
H	-3.047395	-3.698747	-4.004945
C	-1.609417	-4.576288	-2.671639
H	-1.178610	-5.218384	-3.438390
C	-1.085375	-4.576778	-1.386127
H	-0.238804	-5.220875	-1.147521
C	-1.613384	-3.754470	-0.386108
C	-4.321209	-1.917430	-2.425896
H	-4.777331	-1.499465	-1.516375
C	-3.695220	-0.756664	-3.201974
H	-3.254740	-1.106641	-4.144388
H	-2.878984	-0.278673	-2.641783
H	-4.449471	0.005073	-3.443737
C	-5.440711	-2.566404	-3.235853
H	-6.238491	-1.840242	-3.435704
H	-5.887610	-3.423270	-2.715003
H	-5.081540	-2.925648	-4.208578
C	-1.001048	-3.777896	0.999524
H	-1.423422	-2.943336	1.577301
C	-1.355845	-5.071196	1.733383
H	-2.441285	-5.201571	1.833944
H	-0.922398	-5.079800	2.741355
H	-0.967262	-5.947098	1.196240
C	0.509505	-3.571564	0.951100
H	0.927188	-3.514108	1.964353
H	0.763576	-2.633121	0.433639
H	1.020273	-4.396935	0.436577
C	-3.578775	1.897848	1.587311
C	-3.201860	2.431439	2.834192
C	-3.115871	3.821013	2.956446
H	-2.825491	4.250849	3.915347
C	-3.378963	4.656768	1.880655
H	-3.313298	5.737256	1.998397
C	-3.691956	4.109168	0.642105
H	-3.856243	4.767706	-0.208927
C	-3.782506	2.727404	0.462925
C	-2.822392	1.557484	4.013459
H	-3.117696	0.522959	3.787651
C	-1.303961	1.569818	4.205734
H	-0.953980	2.584140	4.443987
H	-0.777618	1.233280	3.303585
H	-1.014110	0.912219	5.035293
C	-3.518309	1.975314	5.307846
H	-3.290529	1.266346	6.112812
H	-4.609489	2.025188	5.202055
H	-3.180456	2.963881	5.644391
C	-4.097676	2.140474	-0.900750
H	-3.507401	1.211870	-1.001896
C	-3.671623	3.037615	-2.056656
H	-4.321616	3.917813	-2.154760
H	-3.725216	2.483176	-3.001037
H	-2.637919	3.385733	-1.937885
C	-5.573032	1.758618	-1.028126
H	-6.216267	2.637859	-0.884802
H	-5.870522	0.999584	-0.294159

H	-5.782996	1.350479	-2.025483
H	-0.759033	-0.346462	1.579587
C	-0.174042	0.021250	-2.106128
C	-0.427593	1.209642	-2.815937
C	-0.119947	-1.158624	-2.876249
C	-0.622833	1.224753	-4.198502
H	-0.465817	2.157956	-2.272627
C	-0.303152	-1.153952	-4.259852
H	0.046210	-2.118600	-2.378608
C	-0.564189	0.040171	-4.933027
H	-0.817475	2.173456	-4.704638
H	-0.259118	-2.095193	-4.812619
H	-0.717255	0.047731	-6.012173

Int-4b_p1-PPdAl-tra.log

SCF (wB97x) = -2648.53641223
H(0 K)= -2647.311286
H(298 K)= -2647.247885
G(298 K)= -2647.409065
Lowest Frequency = 14.1507cm-1

Pd	0.477276	-0.225546	0.575615
P	2.549192	-0.190497	-0.598614
C	2.591140	-1.506160	-1.910559
H	3.611603	-1.558577	-2.329141
C	1.626332	-1.232711	-3.066517
H	0.608995	-1.102555	-2.655029
H	1.877171	-0.290711	-3.574951
C	1.645471	-2.374041	-4.079988
H	0.942182	-2.163624	-4.898234
H	2.644952	-2.424358	-4.542631
C	1.329250	-3.714133	-3.427596
H	1.369613	-4.523337	-4.169575
H	0.296191	-3.694470	-3.040922
C	2.278014	-3.994111	-2.269077
H	3.303091	-4.118448	-2.656804
H	2.016317	-4.939947	-1.775644
C	2.254406	-2.853945	-1.258918
H	2.934422	-3.063852	-0.419661
H	1.245853	-2.773334	-0.814073
C	4.044962	-0.579913	0.439900
H	3.641151	-1.340277	1.132513
C	5.275589	-1.191141	-0.235914
H	5.725439	-0.468922	-0.936035
H	4.999781	-2.071266	-0.833100
C	6.307515	-1.597576	0.813970
H	5.874821	-2.394096	1.441512
H	7.192441	-2.030210	0.327152
C	6.701424	-0.423578	1.702166
H	7.214305	0.336367	1.088876
H	7.425002	-0.744882	2.463479
C	5.475966	0.203244	2.356659
H	5.764255	1.070239	2.967036
H	5.008565	-0.521320	3.042400
C	4.446412	0.615228	1.311265
H	3.556888	1.043791	1.794726
H	4.875576	1.407073	0.674172
C	2.846745	1.378560	-1.547243

H	2.073988	1.315346	-2.334188
C	2.504116	2.637867	-0.741067
H	1.503194	2.528386	-0.292341
H	3.208594	2.755411	0.096472
C	2.565443	3.876968	-1.628307
H	2.343405	4.775715	-1.036909
H	1.771178	3.807599	-2.391247
C	3.916389	4.007372	-2.322398
H	3.935292	4.895817	-2.968101
H	4.700524	4.160463	-1.562602
C	4.242849	2.755312	-3.127816
H	5.225096	2.848339	-3.610934
H	3.505757	2.644660	-3.940732
C	4.200713	1.509674	-2.247587
H	4.998642	1.577448	-1.490766
H	4.417026	0.609966	-2.843347
Al	-1.831684	0.174038	0.602351
N	-3.373566	-0.866335	0.103349
N	-2.813040	1.833281	0.573721
C	-4.642364	-0.480026	0.282629
C	-4.995860	0.836231	0.613777
H	-6.055144	1.032325	0.749167
C	-4.140251	1.946596	0.672934
C	-5.745045	-1.478914	0.098910
H	-5.661206	-1.983143	-0.871347
H	-6.728312	-1.006865	0.169064
H	-5.684960	-2.268097	0.859361
C	-4.749821	3.305103	0.850580
H	-5.841605	3.257579	0.860914
H	-4.432585	3.991632	0.056622
H	-4.411056	3.756877	1.792644
C	-3.082239	-2.206154	-0.328887
C	-2.753407	-2.416137	-1.687522
C	-2.406755	-3.711717	-2.078562
H	-2.161455	-3.910066	-3.119539
C	-2.347768	-4.754815	-1.160347
H	-2.059796	-5.752041	-1.489243
C	-2.647097	-4.518880	0.172656
H	-2.583606	-5.333464	0.893999
C	-3.026713	-3.248356	0.615870
C	-2.741967	-1.268625	-2.687108
H	-2.175542	-0.445139	-2.216815
C	-2.026371	-1.615974	-3.987389
H	-1.037659	-2.052236	-3.811693
H	-1.890628	-0.714241	-4.596429
H	-2.605281	-2.328730	-4.590397
C	-4.137360	-0.733852	-3.021968
H	-4.789909	-1.540867	-3.382427
H	-4.069743	0.014255	-3.822754
H	-4.628366	-0.248981	-2.171949
C	-3.319846	-3.036912	2.089107
H	-3.753981	-2.034635	2.215783
C	-2.030837	-3.084250	2.909704
H	-2.239512	-2.912982	3.973338
H	-1.306635	-2.328943	2.578892
H	-1.545820	-4.065836	2.819526
C	-4.332430	-4.049927	2.622468
H	-5.257093	-4.069623	2.030926
H	-4.599356	-3.815824	3.659935

H	-3.922480	-5.067911	2.617304
C	-2.008015	3.012112	0.411740
C	-1.526661	3.704789	1.537287
C	-0.722070	4.827307	1.324357
H	-0.336643	5.374666	2.184248
C	-0.406212	5.253647	0.041092
H	0.212652	6.137780	-0.104470
C	-0.870355	4.540637	-1.057538
H	-0.605795	4.868858	-2.063076
C	-1.662399	3.401625	-0.898695
C	-1.805137	3.230694	2.948803
H	-2.619505	2.492567	2.905678
C	-0.571844	2.520065	3.508879
H	0.279476	3.212392	3.569905
H	-0.272829	1.677177	2.869658
H	-0.761725	2.128569	4.516412
C	-2.253172	4.360272	3.872671
H	-1.451508	5.091370	4.037521
H	-2.532293	3.963732	4.856060
H	-3.116116	4.906373	3.470168
C	-2.141354	2.640994	-2.121235
H	-2.562784	1.683680	-1.781070
C	-0.996206	2.312956	-3.076392
H	-0.550493	3.217894	-3.511164
H	-1.356997	1.696291	-3.910610
H	-0.204536	1.754505	-2.559073
C	-3.256189	3.398635	-2.842604
H	-4.130588	3.550275	-2.196996
H	-3.591759	2.850636	-3.732111
H	-2.911982	4.388301	-3.171790
C	1.286440	-1.084710	2.279370
C	1.430371	-2.484776	2.343824
C	1.846911	-0.357068	3.343888
C	2.103122	-3.117645	3.390282
H	0.999696	-3.105777	1.552871
C	2.520093	-0.979620	4.397653
H	1.771569	0.733091	3.352396
C	2.658761	-2.366013	4.424656
H	2.191849	-4.205444	3.398141
H	2.944855	-0.374207	5.200545
H	3.187336	-2.854152	5.242739
H	-0.542022	0.402161	-0.706569

Int-5a_p2-PdAl2.log

SCF (wB97x) = -2842.72037050
H(0 K)= -2841.342235
H(298 K)= -2841.262651
G(298 K)= -2841.459776
Lowest Frequency = 11.1117cm-1

Pd	-0.209762	-0.095799	0.387343
Al	2.410558	0.288088	0.394544
N	3.560921	1.784636	0.033208
N	3.738351	-0.680374	1.405580
C	4.544907	2.127069	0.877076
C	5.035994	1.276977	1.877628
H	5.826839	1.679260	2.503964
C	4.719351	-0.079935	2.071553

C	5.185036	3.477028	0.737318
H	5.562077	3.634027	-0.280708
H	6.011205	3.601709	1.441757
H	4.450348	4.273212	0.914817
C	5.519365	-0.844959	3.083814
H	5.018516	-0.796757	4.060552
H	6.520239	-0.421181	3.206679
H	5.606108	-1.904691	2.823357
C	3.244386	2.670199	-1.051828
C	3.806217	2.407627	-2.320576
C	3.501516	3.283214	-3.364494
H	3.921707	3.104867	-4.352298
C	2.645276	4.363089	-3.173549
H	2.417434	5.029808	-4.003693
C	2.061151	4.570533	-1.932655
H	1.360397	5.394388	-1.794884
C	2.347371	3.734278	-0.848948
C	4.654972	1.169421	-2.555143
H	4.187477	0.359053	-1.972515
C	4.657125	0.718351	-4.011193
H	3.639248	0.618102	-4.407040
H	5.147123	-0.258073	-4.101393
H	5.208444	1.414241	-4.657764
C	6.091884	1.319474	-2.052008
H	6.582921	2.188655	-2.511165
H	6.680410	0.430649	-2.315447
H	6.146313	1.431009	-0.962700
C	1.631045	3.946981	0.469431
H	2.083995	3.286677	1.222797
C	0.162491	3.550431	0.327461
H	-0.363308	3.616789	1.289178
H	0.041818	2.516698	-0.034937
H	-0.349931	4.219856	-0.376992
C	1.740456	5.383900	0.975998
H	2.781891	5.716867	1.075473
H	1.260005	5.479749	1.957396
H	1.235416	6.087028	0.300437
C	3.503713	-2.090109	1.489875
C	2.683073	-2.612726	2.506898
C	2.352488	-3.970466	2.446508
H	1.707882	-4.392105	3.218881
C	2.821737	-4.782099	1.422177
H	2.547611	-5.835665	1.392744
C	3.637875	-4.247093	0.430646
H	3.994968	-4.887134	-0.375017
C	3.982916	-2.895458	0.434584
C	2.160554	-1.765832	3.650489
H	2.531292	-0.739724	3.513969
C	0.635008	-1.699837	3.662165
H	0.199576	-2.693672	3.835013
H	0.232447	-1.311023	2.712656
H	0.286688	-1.039381	4.466618
C	2.674248	-2.288604	4.993505
H	2.263750	-3.284034	5.209625
H	2.369076	-1.624990	5.812106
H	3.767524	-2.378441	5.016062
C	4.856999	-2.303858	-0.653673
H	4.615059	-1.232705	-0.716896
C	4.600217	-2.900506	-2.031856

H	4.898150	-3.955828	-2.090165
H	5.188371	-2.363037	-2.786771
H	3.541439	-2.826216	-2.310176
C	6.335706	-2.408377	-0.276259
H	6.557432	-1.871273	0.654812
H	6.972115	-1.984172	-1.063353
H	6.631897	-3.456702	-0.134431
Al	-2.553074	-0.191838	0.384907
N	-3.901975	-1.535903	-0.124409
N	-3.982475	1.088391	0.846235
C	-5.200541	-1.537711	0.196730
C	-5.825545	-0.456354	0.828289
H	-6.878104	-0.576725	1.066909
C	-5.273055	0.810391	1.062313
C	-6.050079	-2.722717	-0.160289
H	-6.118417	-2.843105	-1.248947
H	-7.062234	-2.623751	0.240903
H	-5.607068	-3.653029	0.216607
C	-6.198670	1.885482	1.556134
H	-5.914864	2.218877	2.562568
H	-7.233048	1.533179	1.589806
H	-6.148152	2.775754	0.917054
C	-3.426996	-2.581317	-0.989580
C	-3.510208	-2.382273	-2.382395
C	-3.060876	-3.399535	-3.226818
H	-3.116417	-3.254737	-4.306543
C	-2.540316	-4.581831	-2.716668
H	-2.199003	-5.366481	-3.390406
C	-2.438613	-4.747455	-1.341780
H	-2.004832	-5.663684	-0.939136
C	-2.866805	-3.755688	-0.455378
C	-4.032341	-1.092523	-2.986502
H	-4.428722	-0.464183	-2.175312
C	-2.892356	-0.320046	-3.650238
H	-2.446022	-0.901905	-4.468601
H	-2.087278	-0.092039	-2.936977
H	-3.259450	0.624581	-4.074696
C	-5.171984	-1.335330	-3.973737
H	-5.565970	-0.383695	-4.352044
H	-6.003858	-1.883185	-3.513617
H	-4.836465	-1.916589	-4.842561
C	-2.680911	-3.964134	1.033108
H	-3.155447	-3.122588	1.558316
C	-3.340309	-5.252556	1.521839
H	-4.409891	-5.288159	1.278155
H	-3.240518	-5.350202	2.609830
H	-2.873509	-6.138316	1.071330
C	-1.193062	-3.949139	1.384807
H	-1.045237	-4.105147	2.461525
H	-0.729638	-2.989294	1.111381
H	-0.652331	-4.746587	0.854455
C	-3.559024	2.453287	0.999213
C	-3.300293	2.980836	2.278247
C	-2.973980	4.335826	2.379938
H	-2.772626	4.760763	3.363300
C	-2.897474	5.144197	1.254012
H	-2.653265	6.200866	1.353916
C	-3.089779	4.589887	-0.005657
H	-2.976894	5.217567	-0.888275

C	-3.395419	3.236339	-0.162784
C	-3.292135	2.110969	3.519446
H	-3.792696	1.162354	3.277950
C	-1.847405	1.782763	3.905741
H	-1.300081	2.701003	4.161884
H	-1.310316	1.291999	3.081071
H	-1.818674	1.119322	4.780188
C	-4.032983	2.741031	4.696442
H	-4.083948	2.039527	5.537786
H	-5.059611	3.030503	4.436792
H	-3.522870	3.641950	5.060863
C	-3.547829	2.628023	-1.545448
H	-3.197576	1.582836	-1.480757
C	-2.674029	3.306430	-2.593986
H	-3.002184	4.332036	-2.808126
H	-2.721294	2.753100	-3.539176
H	-1.622602	3.340195	-2.280935
C	-5.008160	2.585324	-1.995247
H	-5.439074	3.595640	-2.024735
H	-5.626515	1.973857	-1.325839
H	-5.091682	2.157557	-3.003587
H	1.231704	0.710424	1.456587
C	1.672793	-0.690984	-1.168803
C	1.440066	-0.131431	-2.447468
C	1.143834	-1.992601	-0.965946
C	0.813144	-0.840958	-3.468566
H	1.763517	0.891081	-2.656093
C	0.505635	-2.707550	-1.983748
H	1.275865	-2.489237	-0.001840
C	0.353414	-2.139563	-3.243770
H	0.672898	-0.374756	-4.444830
H	0.119958	-3.706909	-1.780979
H	-0.144206	-2.695069	-4.039013

Int-5b_p2-PPdAl.log

SCF (wB97x) = -2648.57641049
H(0 K)= -2647.349855
H(298 K)= -2647.286651
G(298 K)= -2647.446796
Lowest Frequency = 19.9540cm-1

Pd	1.061310	-0.135657	-1.175997
P	3.092702	-0.249294	-0.059217
C	4.439597	-0.763953	-1.234789
H	5.405682	-0.796170	-0.699778
C	4.560984	0.224893	-2.399788
H	3.571322	0.313970	-2.883502
H	4.813660	1.230434	-2.034738
C	5.597602	-0.228554	-3.422489
H	5.648611	0.492060	-4.249792
H	6.594244	-0.228500	-2.951198
C	5.291191	-1.626003	-3.945754
H	6.061951	-1.949383	-4.658129
H	4.341347	-1.602108	-4.503692
C	5.169243	-2.620493	-2.798312
H	6.146707	-2.721206	-2.297870
H	4.912751	-3.619213	-3.176577
C	4.130967	-2.164748	-1.779666

H	4.058313	-2.896220	-0.961770
H	3.131389	-2.134940	-2.251435
C	3.184422	-1.548372	1.274729
H	2.679311	-2.398435	0.782541
C	4.558757	-2.055289	1.725383
H	5.101824	-1.260415	2.256571
H	5.182626	-2.331911	0.863805
C	4.394123	-3.256043	2.655124
H	3.923465	-4.079802	2.092442
H	5.377961	-3.624069	2.977040
C	3.527081	-2.911801	3.860833
H	4.045149	-2.150570	4.467516
H	3.404510	-3.790084	4.509029
C	2.170430	-2.366773	3.430389
H	1.571670	-2.074078	4.303226
H	1.599508	-3.159877	2.919063
C	2.319379	-1.180980	2.484096
H	1.332664	-0.823608	2.154424
H	2.788321	-0.343405	3.028570
C	3.704839	1.374252	0.622758
H	3.962369	1.935192	-0.294524
C	2.577703	2.157156	1.302492
H	1.706997	2.199456	0.632644
H	2.238044	1.615822	2.199161
C	3.024183	3.560222	1.693971
H	2.205138	4.081583	2.210492
H	3.231678	4.141042	0.779365
C	4.277905	3.528756	2.560529
H	4.606342	4.547624	2.806198
H	4.041977	3.039228	3.519799
C	5.398663	2.756767	1.873289
H	6.291287	2.718544	2.512440
H	5.697304	3.288366	0.954856
C	4.950390	1.343296	1.509809
H	4.715638	0.799597	2.438999
H	5.768103	0.790717	1.023406
Al	-1.368766	0.005059	-0.520089
N	-2.739213	-1.220355	-1.119111
N	-2.327831	1.602980	-1.117243
C	-3.360729	-0.952653	-2.277975
C	-3.404892	0.326694	-2.843663
H	-3.923715	0.411068	-3.794936
C	-3.044157	1.547628	-2.233767
C	-4.070821	-2.062425	-2.997687
H	-3.392356	-2.910052	-3.159250
H	-4.456648	-1.728479	-3.964268
H	-4.907984	-2.455241	-2.408672
C	-3.522453	2.801446	-2.907357
H	-2.892298	3.019554	-3.779620
H	-3.481799	3.669056	-2.242035
H	-4.547091	2.681824	-3.274249
C	-3.051198	-2.444005	-0.446228
C	-2.163202	-3.535360	-0.504446
C	-2.496432	-4.701506	0.190753
H	-1.812622	-5.550162	0.155451
C	-3.673937	-4.796898	0.920175
H	-3.918705	-5.716679	1.448795
C	-4.530848	-3.704969	0.982053
H	-5.445645	-3.769785	1.572471

C	-4.236418	-2.513038	0.317334
C	-0.843920	-3.466735	-1.245616
H	-0.803098	-2.519637	-1.803485
C	0.319967	-3.457301	-0.252843
H	0.242534	-2.616227	0.452295
H	1.276231	-3.348117	-0.783684
H	0.351343	-4.389536	0.330959
C	-0.680352	-4.602023	-2.253108
H	-0.642423	-5.581925	-1.758732
H	0.255123	-4.482295	-2.813795
H	-1.504936	-4.630804	-2.976030
C	-5.164739	-1.324972	0.487909
H	-4.738008	-0.472498	-0.059095
C	-5.257404	-0.926168	1.960472
H	-5.903394	-0.046580	2.082375
H	-4.270540	-0.686936	2.377302
H	-5.688611	-1.734229	2.566484
C	-6.559524	-1.594614	-0.074462
H	-6.536108	-1.841414	-1.142826
H	-7.203721	-0.714595	0.048484
H	-7.043305	-2.431959	0.446358
C	-1.989969	2.836519	-0.473868
C	-2.647487	3.150852	0.737162
C	-2.210642	4.263651	1.456552
H	-2.695972	4.516777	2.396848
C	-1.145859	5.038388	1.009043
H	-0.808251	5.892536	1.594144
C	-0.519982	4.722329	-0.187515
H	0.305327	5.339832	-0.544197
C	-0.926620	3.627300	-0.958661
C	-3.797730	2.295672	1.234559
H	-3.540750	1.247117	1.014670
C	-4.020556	2.397736	2.737695
H	-4.371705	3.394906	3.035167
H	-3.102123	2.175141	3.295240
H	-4.787450	1.682783	3.055951
C	-5.087063	2.616575	0.475909
H	-5.379015	3.665222	0.623599
H	-5.913908	1.986440	0.831013
H	-4.982058	2.446868	-0.602728
C	-0.244641	3.391420	-2.295011
H	-0.662122	2.479085	-2.745596
C	1.261498	3.173254	-2.159625
H	1.751817	4.026966	-1.670479
H	1.720084	3.048810	-3.149635
H	1.477152	2.262112	-1.578733
C	-0.505649	4.571471	-3.236233
H	-1.571394	4.812478	-3.323630
H	-0.119053	4.360390	-4.241210
H	0.001086	5.476003	-2.874053
C	-1.172575	-0.002075	1.466577
C	-1.512251	-1.078316	2.310091
C	-0.587150	1.113487	2.098709
C	-1.293642	-1.039839	3.688325
H	-1.959857	-1.982269	1.891442
C	-0.370056	1.168646	3.473992
H	-0.277080	1.971426	1.499960
C	-0.722957	0.085764	4.277849
H	-1.568707	-1.896635	4.303933

H	0.081058	2.059434	3.915314
H	-0.551346	0.116874	5.353502
H	-0.421787	-0.100417	-2.007845

PdbisPCy3.log

SCF (wB97x) = -2222.15623622
H(0 K)= -2221.181895
H(298 K)= -2221.139520
G(298 K)= -2221.260694
Lowest Frequency = 2.4842cm-1

Pd	0.417559	1.170198	1.627974
P	2.665904	1.666765	1.815197
P	-1.822995	0.718995	1.301188
C	-2.490424	1.635913	-0.182684
C	-3.823454	1.220297	-0.814311
C	-2.454439	3.141769	0.112877
H	-1.706879	1.447593	-0.938402
C	-4.134794	2.068491	-2.046069
H	-4.643630	1.317563	-0.088249
H	-3.794046	0.161173	-1.101794
C	-2.780497	3.967549	-1.125723
H	-3.185643	3.379860	0.903574
H	-1.464972	3.408918	0.516278
C	-4.119989	3.557318	-1.724784
H	-5.103102	1.771614	-2.471488
H	-3.379712	1.861719	-2.822311
H	-2.776291	5.037864	-0.878864
H	-1.987111	3.818779	-1.876171
H	-4.340058	4.147751	-2.624359
H	-4.923398	3.780778	-1.003164
C	-2.865778	1.212982	2.769147
C	-4.386161	1.344966	2.628165
C	-2.485367	0.323634	3.961353
H	-2.478180	2.222187	2.996657
C	-5.021623	1.808333	3.937613
H	-4.832287	0.386441	2.325577
H	-4.631665	2.060798	1.832829
C	-3.142149	0.797114	5.252369
H	-2.798662	-0.714847	3.762107
H	-1.388992	0.301126	4.062489
C	-4.654837	0.892562	5.098057
H	-6.112161	1.874084	3.822889
H	-4.672540	2.829542	4.162713
H	-2.876933	0.125192	6.079815
H	-2.741867	1.789038	5.517465
H	-5.118540	1.245436	6.029011
H	-5.064466	-0.113831	4.909587
C	-2.121080	-1.100117	1.000043
C	-3.535268	-1.679994	1.113193
C	-1.455817	-1.493289	-0.326581
H	-1.520096	-1.567569	1.800596
C	-3.530881	-3.189425	0.879412
H	-4.209641	-1.201061	0.388535
H	-3.953485	-1.467672	2.105705
C	-1.476289	-3.001385	-0.544590
H	-1.983437	-1.005151	-1.162993
H	-0.424769	-1.105722	-0.344730

C	-2.894486	-3.551431	-0.456055
H	-4.554963	-3.581965	0.941988
H	-2.963634	-3.675312	1.690306
H	-1.022411	-3.249419	-1.513616
H	-0.851727	-3.485062	0.223908
H	-2.898548	-4.639870	-0.602718
H	-3.500105	-3.126135	-1.273678
C	2.988061	3.505643	1.798235
C	4.342048	4.038597	2.276788
C	2.606554	4.084094	0.430712
H	2.233007	3.873440	2.515826
C	4.360870	5.565968	2.266972
H	5.153390	3.663200	1.635039
H	4.557850	3.673642	3.290979
C	2.642387	5.607611	0.437466
H	3.308090	3.713654	-0.335544
H	1.609938	3.713169	0.143175
C	4.001107	6.123640	0.895055
H	5.344609	5.932931	2.589919
H	3.633794	5.937565	3.007441
H	2.394568	5.997562	-0.559035
H	1.863152	5.983080	1.120406
H	4.013777	7.221724	0.911054
H	4.769302	5.815649	0.166286
C	3.571045	0.961346	0.339981
C	5.006848	1.385360	0.013816
C	3.439354	-0.567467	0.338554
H	2.936018	1.327167	-0.487744
C	5.459148	0.780865	-1.314277
H	5.693029	1.061179	0.808916
H	5.084874	2.479325	-0.036429
C	3.909863	-1.166885	-0.981485
H	4.042881	-0.987887	1.160937
H	2.393798	-0.844618	0.547664
C	5.334808	-0.737582	-1.309779
H	6.491648	1.084731	-1.534461
H	4.835715	1.192142	-2.125356
H	3.832603	-2.262204	-0.949935
H	3.234878	-0.831379	-1.785791
H	5.652157	-1.153457	-2.275547
H	6.020879	-1.153163	-0.553216
C	3.496207	1.079804	3.383432
C	2.781836	-0.158290	3.943111
C	5.009547	0.842228	3.364610
H	3.295951	1.910210	4.087347
C	3.303579	-0.525525	5.327559
H	2.936016	-1.005171	3.253273
H	1.696014	0.018499	3.957954
C	5.527772	0.493344	4.757379
H	5.231923	0.004444	2.683785
H	5.545683	1.712543	2.965829
C	4.814172	-0.727985	5.323598
H	2.794427	-1.425383	5.698519
H	3.050310	0.284467	6.031378
H	6.613473	0.328143	4.726876
H	5.364533	1.354644	5.426494
H	5.178251	-0.953799	6.334969
H	5.059487	-1.605516	4.703109

TS-1a-toluene_meta1.log

SCF (wB97x) = -2881.98773384
H(0 K)= -2880.582500
H(298 K)= -2880.502244
G(298 K)= -2880.695362
Lowest Frequency = -706.9746cm-1

Pd	-0.049122	-0.055924	1.254277
Al	-1.757422	-0.085864	-0.480994
N	-2.937092	1.471351	-0.739079
N	-2.367581	-0.876520	-2.186454
C	-3.567552	1.832506	-1.860421
C	-3.619205	1.013301	-2.997304
H	-4.193776	1.391977	-3.837620
C	-3.162281	-0.304449	-3.102344
C	-4.314622	3.133662	-1.916334
H	-5.034596	3.211048	-1.092070
H	-4.848611	3.244558	-2.863878
H	-3.630280	3.983965	-1.802753
C	-3.677509	-1.108250	-4.264002
H	-2.933297	-1.799522	-4.671244
H	-4.038024	-0.453940	-5.063105
H	-4.525103	-1.727428	-3.937481
C	-3.147794	2.276503	0.437504
C	-4.069009	1.810704	1.399454
C	-4.346808	2.632257	2.492948
H	-5.061795	2.298300	3.242154
C	-3.713223	3.858338	2.654802
H	-3.948824	4.482516	3.515660
C	-2.752269	4.262242	1.739022
H	-2.218460	5.200068	1.892998
C	-2.446914	3.482073	0.620767
C	-4.736883	0.455160	1.259462
H	-3.993260	-0.223782	0.802493
C	-5.114366	-0.161124	2.600864
H	-4.265237	-0.166237	3.295463
H	-5.444135	-1.197669	2.461450
H	-5.946642	0.374339	3.077629
C	-5.943722	0.500211	0.321631
H	-6.703892	1.198453	0.698414
H	-6.412193	-0.490950	0.246247
H	-5.671394	0.814456	-0.693988
C	-1.319772	3.899315	-0.298353
H	-1.381054	3.291830	-1.211311
C	0.017646	3.586072	0.373143
H	0.855883	3.827073	-0.292746
H	0.090945	2.521920	0.649509
H	0.134401	4.175210	1.294446
C	-1.376958	5.366475	-0.715005
H	-2.339388	5.637832	-1.168856
H	-0.584940	5.582757	-1.443879
H	-1.218302	6.037711	0.138947
C	-2.087335	-2.276595	-2.344719
C	-0.872308	-2.679545	-2.935071
C	-0.669392	-4.045215	-3.150758
H	0.249971	-4.377318	-3.631662
C	-1.616213	-4.987358	-2.765982
H	-1.435696	-6.046151	-2.944191

C	-2.780987	-4.574135	-2.132875
H	-3.505336	-5.316836	-1.799086
C	-3.038741	-3.219718	-1.906999
C	0.156540	-1.665411	-3.398779
H	0.030276	-0.761586	-2.780993
C	1.597843	-2.130103	-3.220601
H	1.837323	-3.009610	-3.833174
H	1.824765	-2.380908	-2.176887
H	2.291656	-1.335648	-3.526965
C	-0.083171	-1.271315	-4.856772
H	-0.063587	-2.155206	-5.509196
H	0.700022	-0.584899	-5.204192
H	-1.048753	-0.771514	-4.998895
C	-4.285629	-2.809664	-1.146615
H	-4.431604	-1.726080	-1.273154
C	-4.091697	-3.074542	0.348799
H	-3.908544	-4.141762	0.535301
H	-4.985470	-2.781617	0.915289
H	-3.235201	-2.518340	0.757721
C	-5.546612	-3.504264	-1.654628
H	-5.684706	-3.371065	-2.735059
H	-6.434266	-3.104662	-1.149487
H	-5.524104	-4.583730	-1.457686
Al	1.970616	-0.029904	-0.053899
N	3.214124	-1.547964	0.184120
N	3.167556	0.821454	-1.355714
C	4.257135	-1.850561	-0.587546
C	4.630870	-1.066490	-1.693254
H	5.447003	-1.449147	-2.299592
C	4.174255	0.215805	-2.009329
C	5.129620	-3.026842	-0.258449
H	4.563761	-3.850890	0.187173
H	5.891709	-2.734920	0.476875
H	5.656127	-3.384772	-1.148539
C	4.870352	0.944455	-3.122499
H	4.167873	1.173220	-3.934950
H	5.692280	0.352461	-3.533518
H	5.267167	1.909493	-2.783384
C	3.009767	-2.265179	1.415654
C	3.816670	-1.965749	2.529626
C	3.553163	-2.620753	3.735505
H	4.164050	-2.388348	4.608141
C	2.517171	-3.535407	3.846276
H	2.321400	-4.030167	4.795904
C	1.716013	-3.801375	2.742898
H	0.890817	-4.504884	2.838431
C	1.932852	-3.170371	1.516790
C	4.890140	-0.895144	2.506478
H	5.024216	-0.545881	1.471521
C	4.438501	0.302208	3.343486
H	4.300666	0.012058	4.393315
H	3.479266	0.705086	2.991314
H	5.189274	1.103407	3.316005
C	6.241373	-1.404899	3.004832
H	7.010664	-0.629507	2.898604
H	6.579750	-2.293837	2.458041
H	6.198713	-1.675902	4.067609
C	1.066324	-3.505679	0.320717
H	0.980780	-2.595725	-0.295556

C	1.727526	-4.593386	-0.526376
H	2.695085	-4.267424	-0.930753
H	1.087078	-4.867615	-1.373955
H	1.903467	-5.498138	0.072023
C	-0.360322	-3.889048	0.687688
H	-0.962243	-3.987366	-0.223666
H	-0.824170	-3.115486	1.314467
H	-0.416055	-4.850542	1.216979
C	2.979062	2.232946	-1.567557
C	2.206412	2.718792	-2.639124
C	2.098694	4.102753	-2.809157
H	1.496864	4.489653	-3.631810
C	2.735972	4.987856	-1.952664
H	2.650665	6.061872	-2.109802
C	3.454121	4.494554	-0.870531
H	3.916252	5.192388	-0.175232
C	3.570656	3.121883	-0.641002
C	1.436830	1.813208	-3.579054
H	1.751571	0.774694	-3.393058
C	-0.059809	1.907778	-3.275600
H	-0.423637	2.937248	-3.408663
H	-0.268245	1.617635	-2.234288
H	-0.649145	1.258550	-3.936305
C	1.702497	2.139761	-5.048143
H	1.175233	1.438123	-5.705931
H	2.770463	2.098163	-5.296373
H	1.348217	3.145529	-5.306656
C	4.306390	2.615776	0.586770
H	3.827687	1.669157	0.890941
C	4.186168	3.567280	1.771829
H	4.744885	4.498411	1.612543
H	4.594200	3.102527	2.675713
H	3.139361	3.827120	1.971265
C	5.772230	2.298435	0.289375
H	6.300537	3.191872	-0.070928
H	5.878841	1.513318	-0.469475
H	6.284574	1.950554	1.196665
H	0.688605	0.105383	2.684507
C	-0.952581	-0.199248	3.150907
C	-1.524499	0.921959	3.777754
C	-1.136377	-1.446189	3.770383
C	-2.283065	0.818770	4.948319
H	-1.383695	1.913618	3.337738
C	-1.902284	-1.568072	4.928317
H	-0.668551	-2.335090	3.343948
C	-2.475348	-0.445432	5.518733
H	-2.043329	-2.550245	5.382270
H	-3.061221	-0.542392	6.434139
C	-2.888356	2.035694	5.584892
H	-3.984880	2.043699	5.491478
H	-2.667316	2.084481	6.659385
H	-2.517456	2.956920	5.121365

TS-1a-toluene_meta2.log

SCF (wB97x) = -2881.98713229
H(0 K)= -2880.581939
H(298 K)= -2880.501656
G(298 K)= -2880.695143

Lowest Frequency = -716.8842cm-1

Pd	-0.026070	0.738396	-0.993292
Al	-1.781636	-0.330153	0.310670
N	-2.886070	-1.736556	-0.517496
N	-2.459225	-0.804448	2.105578
C	-3.509221	-2.743986	0.099759
C	-3.620313	-2.823317	1.495643
H	-4.185004	-3.664821	1.886458
C	-3.236971	-1.849193	2.423412
C	-4.182754	-3.815875	-0.706958
H	-4.885978	-3.383025	-1.429323
H	-4.722081	-4.517549	-0.065003
H	-3.449305	-4.379480	-1.297337
C	-3.814702	-1.968343	3.806531
H	-3.118576	-1.656497	4.591155
H	-4.147457	-2.991448	4.004677
H	-4.692860	-1.313287	3.896955
C	-3.037907	-1.622455	-1.946460
C	-3.976489	-0.689160	-2.436479
C	-4.184376	-0.628016	-3.815483
H	-4.901700	0.086039	-4.215307
C	-3.468966	-1.437162	-4.689756
H	-3.645810	-1.371237	-5.762286
C	-2.499266	-2.296698	-4.193154
H	-1.902479	-2.895174	-4.881837
C	-2.259354	-2.402713	-2.820408
C	-4.732739	0.232977	-1.497268
H	-4.037317	0.502124	-0.680658
C	-5.148860	1.541853	-2.156548
H	-4.301596	2.030804	-2.652775
H	-5.549597	2.232566	-1.404886
H	-5.941871	1.390538	-2.901176
C	-5.937374	-0.457476	-0.856525
H	-6.645580	-0.796243	-1.625294
H	-6.474089	0.235827	-0.194204
H	-5.648581	-1.329527	-0.256481
C	-1.122346	-3.271720	-2.327150
H	-1.206708	-3.356872	-1.235164
C	0.210099	-2.587161	-2.633711
H	1.049562	-3.166743	-2.228210
H	0.251603	-1.574019	-2.202980
H	0.355326	-2.491998	-3.719728
C	-1.137896	-4.684260	-2.906290
H	-2.090615	-5.201567	-2.732003
H	-0.337887	-5.286262	-2.455805
H	-0.964281	-4.678573	-3.990271
C	-2.259063	0.198055	3.114908
C	-1.075977	0.186994	3.881602
C	-0.949240	1.129559	4.905424
H	-0.055549	1.121656	5.528369
C	-1.938878	2.075815	5.145006
H	-1.817148	2.800863	5.948024
C	-3.070950	2.106269	4.341659
H	-3.828903	2.871640	4.507605
C	-3.252959	1.176933	3.314532
C	-0.001178	-0.860388	3.659696
H	-0.070604	-1.184983	2.608892
C	1.415946	-0.339594	3.872468

H	1.600939	-0.027965	4.909217
H	1.643400	0.515187	3.223308
H	2.146188	-1.128431	3.646586
C	-0.240663	-2.084525	4.544287
H	-0.274615	-1.799807	5.605006
H	0.571230	-2.813351	4.422065
H	-1.181801	-2.593287	4.303829
C	-4.463913	1.290736	2.408199
H	-4.548719	0.364307	1.819802
C	-4.264480	2.444856	1.422181
H	-4.137437	3.396531	1.956487
H	-5.132844	2.544306	0.757784
H	-3.372782	2.299611	0.794166
C	-5.769152	1.465025	3.180659
H	-5.915038	0.675089	3.928244
H	-6.625809	1.443763	2.496242
H	-5.803977	2.426438	3.708904
Al	1.959000	-0.022622	0.119430
N	3.119979	1.362352	0.923055
N	3.179653	-1.453471	0.663516
C	4.131417	1.154351	1.764993
C	4.530200	-0.134157	2.163848
H	5.312543	-0.182966	2.916031
C	4.140178	-1.351177	1.597982
C	4.940834	2.309248	2.279041
H	5.435294	2.052561	3.220922
H	4.334747	3.209903	2.418807
H	5.726967	2.568231	1.556910
C	4.851822	-2.591177	2.056100
H	4.145966	-3.291033	2.523107
H	5.635892	-2.356964	2.780939
H	5.301494	-3.130185	1.212946
C	2.896537	2.680119	0.387007
C	3.739573	3.163194	-0.631951
C	3.453832	4.409751	-1.195806
H	4.092569	4.787587	-1.994480
C	2.362956	5.156361	-0.777729
H	2.149988	6.121133	-1.235333
C	1.530116	4.655880	0.215028
H	0.662492	5.235136	0.526894
C	1.766367	3.413354	0.806012
C	4.880892	2.351250	-1.212617
H	5.019208	1.443664	-0.605859
C	4.525820	1.907707	-2.632134
H	4.393038	2.777325	-3.288976
H	3.587078	1.338183	-2.656664
H	5.325815	1.287992	-3.058972
C	6.204065	3.115118	-1.216717
H	7.021340	2.471661	-1.566539
H	6.472303	3.491288	-0.221608
H	6.162543	3.981553	-1.889289
C	0.857902	2.908430	1.907432
H	0.808392	1.810105	1.823317
C	1.441052	3.256925	3.277143
H	2.416113	2.779181	3.441407
H	0.769314	2.928897	4.080093
H	1.580567	4.342609	3.374313
C	-0.579118	3.395985	1.783424
H	-1.209211	2.878577	2.517043

H	-0.980800	3.181395	0.783702
H	-0.676307	4.473505	1.977088
C	3.057988	-2.694173	-0.056430
C	2.276765	-3.756460	0.435769
C	2.224726	-4.942961	-0.302814
H	1.616603	-5.769479	0.065642
C	2.926596	-5.086635	-1.490069
H	2.884366	-6.023454	-2.043313
C	3.655830	-4.013620	-1.987254
H	4.169648	-4.115630	-2.940941
C	3.716559	-2.797496	-1.303429
C	1.446563	-3.656609	1.699284
H	1.710130	-2.718668	2.212251
C	-0.039468	-3.589943	1.341463
H	-0.352441	-4.486491	0.786426
H	-0.250938	-2.718027	0.703860
H	-0.668235	-3.517723	2.238464
C	1.709813	-4.818526	2.656764
H	1.136479	-4.698831	3.584133
H	2.770553	-4.904509	2.923815
H	1.407530	-5.777181	2.216724
C	4.460705	-1.620307	-1.908056
H	3.946236	-0.701760	-1.577469
C	4.416171	-1.623680	-3.431970
H	5.010982	-2.440045	-3.861181
H	4.828484	-0.688987	-3.826685
H	3.387897	-1.719486	-3.801572
C	5.901806	-1.528907	-1.405383
H	6.461979	-2.440480	-1.655089
H	5.951704	-1.388265	-0.318502
H	6.420773	-0.679873	-1.870792
H	0.757397	1.472991	-2.201921
C	-0.884521	1.983035	-2.453296
C	-1.454701	1.447170	-3.619589
C	-1.049383	3.358066	-2.227254
C	-2.197884	2.249625	-4.483863
H	-1.333751	0.385649	-3.845380
C	-1.792192	4.177072	-3.082331
H	-0.575810	3.815295	-1.355015
C	-2.371186	3.607424	-4.222050
H	-2.945031	4.232262	-4.908257
H	-2.647928	1.806950	-5.374678
C	-1.967310	5.635240	-2.771673
H	-1.183551	5.995491	-2.092846
H	-1.937621	6.254216	-3.677320
H	-2.931602	5.837201	-2.283553

TS-1a-toluene_ortho1.log

SCF (wB97x) = -2881.99068593
H(0 K)= -2880.584661
H(298 K)= -2880.505015
G(298 K)= -2880.695689
Lowest Frequency = -716.4129cm-1

Pd	-0.024805	-0.472400	1.205716
Al	-1.817324	0.080559	-0.345537
N	-3.038371	1.614733	-0.146837
N	-2.515981	-0.297788	-2.155447

C	-3.764700	2.201570	-1.103515
C	-3.866865	1.682371	-2.401632
H	-4.511775	2.225221	-3.086392
C	-3.379994	0.452248	-2.855667
C	-4.575841	3.426554	-0.790548
H	-5.230975	3.251850	0.072064
H	-5.189708	3.722678	-1.645385
H	-3.932728	4.272366	-0.517292
C	-3.948335	-0.057626	-4.151590
H	-3.213872	-0.590580	-4.763154
H	-4.380080	0.759719	-4.736447
H	-4.753635	-0.776521	-3.944463
C	-3.171895	2.115015	1.197710
C	-3.941971	1.366722	2.112529
C	-4.116619	1.883938	3.398944
H	-4.695274	1.318213	4.126353
C	-3.556304	3.100060	3.768887
H	-3.714047	3.488585	4.773845
C	-2.760036	3.796631	2.869552
H	-2.276249	4.721610	3.182155
C	-2.533754	3.310087	1.580703
C	-4.571985	0.044843	1.713357
H	-3.842131	-0.478541	1.067701
C	-4.833475	-0.877000	2.896483
H	-3.943195	-0.993357	3.525403
H	-5.124836	-1.872979	2.541641
H	-5.655798	-0.508925	3.525288
C	-5.846421	0.243918	0.891343
H	-6.596588	0.804481	1.466069
H	-6.289318	-0.726419	0.627224
H	-5.662449	0.787465	-0.043919
C	-1.515930	3.977401	0.681310
H	-1.714098	3.666599	-0.353773
C	-0.123037	3.462794	1.051879
H	0.640947	3.885852	0.388994
H	-0.064858	2.363806	0.988781
H	0.128104	3.741047	2.085840
C	-1.554366	5.501190	0.715962
H	-2.554519	5.898161	0.496899
H	-0.855612	5.912372	-0.024174
H	-1.250310	5.896373	1.693656
C	-2.225435	-1.604075	-2.680885
C	-1.055307	-1.806211	-3.439714
C	-0.867858	-3.057382	-4.034392
H	0.013883	-3.226918	-4.651281
C	-1.786520	-4.085876	-3.861337
H	-1.621231	-5.050629	-4.338118
C	-2.900660	-3.886149	-3.056303
H	-3.598347	-4.706385	-2.889635
C	-3.139545	-2.651723	-2.447816
C	-0.064085	-0.683265	-3.683050
H	-0.153629	0.024312	-2.842691
C	1.387711	-1.149341	-3.724912
H	1.587087	-1.835708	-4.558630
H	1.685400	-1.658012	-2.799382
H	2.058581	-0.290056	-3.858665
C	-0.402305	0.074133	-4.967818
H	-0.425388	-0.607254	-5.829495
H	0.353973	0.842909	-5.172861

H	-1.376366	0.574565	-4.910298
C	-4.320510	-2.488767	-1.509591
H	-4.500298	-1.413446	-1.358012
C	-3.979460	-3.093266	-0.144278
H	-3.760344	-4.165997	-0.237475
H	-4.819472	-2.979256	0.553703
H	-3.097263	-2.616241	0.307840
C	-5.610009	-3.094060	-2.057500
H	-5.848792	-2.719114	-3.060807
H	-6.453809	-2.855420	-1.398983
H	-5.552880	-4.188229	-2.120384
Al	1.925008	-0.014321	-0.134081
N	3.202233	-1.507322	-0.367594
N	3.048948	1.189623	-1.206915
C	4.210490	-1.564472	-1.235891
C	4.520001	-0.502242	-2.103040
H	5.313913	-0.685151	-2.821545
C	4.035055	0.806423	-2.038369
C	5.115548	-2.761422	-1.271520
H	5.621117	-2.842296	-2.238631
H	4.580103	-3.692237	-1.059865
H	5.893300	-2.665939	-0.501631
C	4.685927	1.824688	-2.930391
H	3.955648	2.262968	-3.623162
H	5.493372	1.378966	-3.517096
H	5.094511	2.661717	-2.350247
C	3.067381	-2.540559	0.625773
C	3.911768	-2.528423	1.751260
C	3.717197	-3.499431	2.736951
H	4.357463	-3.493960	3.619430
C	2.709723	-4.445649	2.625640
H	2.566221	-5.188488	3.408189
C	1.868635	-4.426223	1.520078
H	1.064347	-5.156075	1.448451
C	2.019268	-3.476159	0.508797
C	4.950579	-1.450195	1.989880
H	5.028255	-0.823628	1.088242
C	4.498755	-0.550772	3.141368
H	4.414847	-1.125787	4.072855
H	3.512801	-0.106701	2.946365
H	5.222344	0.257397	3.313793
C	6.337223	-2.021190	2.281294
H	7.077045	-1.215718	2.372261
H	6.679565	-2.706669	1.496015
H	6.348610	-2.580050	3.225916
C	1.107346	-3.489941	-0.700178
H	0.988236	-2.447498	-1.036950
C	1.737446	-4.285971	-1.843179
H	2.683004	-3.841315	-2.180887
H	1.060644	-4.324863	-2.706232
H	1.946725	-5.318310	-1.530091
C	-0.298890	-3.983762	-0.396426
H	-0.933096	-3.864482	-1.281732
H	-0.745103	-3.396843	0.416857
H	-0.325686	-5.046843	-0.119880
C	2.847682	2.604667	-1.027314
C	2.069932	3.358429	-1.926906
C	1.990417	4.742204	-1.742487
H	1.389581	5.335060	-2.432310

C	2.644611	5.369934	-0.692708
H	2.578376	6.449871	-0.571300
C	3.350347	4.604914	0.227057
H	3.823607	5.094099	1.076129
C	3.452212	3.218231	0.092988
C	1.244325	2.725088	-3.027820
H	1.554616	1.674239	-3.131734
C	-0.230450	2.737202	-2.616724
H	-0.586543	3.767653	-2.471588
H	-0.376681	2.203124	-1.664640
H	-0.868626	2.266943	-3.376570
C	1.428852	3.416605	-4.377196
H	0.853708	2.906831	-5.159559
H	2.479847	3.438613	-4.691757
H	1.075639	4.455008	-4.350174
C	4.201925	2.407970	1.134680
H	3.733705	1.409644	1.179434
C	4.086985	3.005764	2.532315
H	4.638705	3.949917	2.625492
H	4.504531	2.318417	3.275804
H	3.040225	3.195072	2.800620
C	5.665311	2.195859	0.746102
H	6.184150	3.158078	0.636233
H	5.764075	1.648560	-0.199984
H	6.191792	1.618054	1.517848
H	0.844990	-0.916912	2.498801
C	-0.802511	-1.213417	3.028260
C	-1.083235	-0.302565	4.072384
C	-1.169593	-2.554794	3.212013
C	-1.768725	-0.737521	5.210059
C	-1.853024	-2.978905	4.351459
H	-0.920744	-3.290292	2.445046
C	-2.163311	-2.066631	5.357019
H	-2.696610	-2.386512	6.251504
H	-1.994783	-0.012874	5.996334
H	-2.142340	-4.025766	4.451921
C	-0.640584	1.121269	3.948273
H	0.447962	1.180721	3.786721
H	-1.099949	1.599932	3.072196
H	-0.896836	1.718310	4.833332

TS-1a-toluene_ortho2.log

SCF (wB97x) = -2881.98938383
H(0 K)= -2880.583581
H(298 K)= -2880.503749
G(298 K)= -2880.695666
Lowest Frequency = -662.9194cm-1

Pd	0.020831	-0.146870	1.257892
Al	1.842228	0.204936	-0.325507
N	3.223879	-1.166963	-0.611635
N	2.433675	1.202431	-1.933171
C	3.983442	-1.342094	-1.697206
C	4.001397	-0.430676	-2.762703
H	4.687828	-0.654688	-3.573973
C	3.369198	0.815404	-2.814658
C	4.914790	-2.517215	-1.783462
H	5.575205	-2.560783	-0.908376

H	5.527969	-2.470744	-2.687410
H	4.358281	-3.463033	-1.790527
C	3.851989	1.768795	-3.873596
H	3.049451	2.374359	-4.305769
H	4.369200	1.232453	-4.674458
H	4.568947	2.476794	-3.434169
C	3.460606	-2.042329	0.510383
C	4.224162	-1.546946	1.586026
C	4.560225	-2.429451	2.614185
H	5.147207	-2.068086	3.456608
C	4.129071	-3.749641	2.599916
H	4.404970	-4.421465	3.411425
C	3.304133	-4.194245	1.575669
H	2.919456	-5.213694	1.596100
C	2.946563	-3.351696	0.520806
C	4.664777	-0.096658	1.636115
H	3.879736	0.497728	1.133395
C	4.754153	0.441176	3.058857
H	3.835823	0.233383	3.622660
H	4.906554	1.527537	3.044800
H	5.599637	0.008726	3.610276
C	5.964761	0.133815	0.866500
H	6.783259	-0.460791	1.295074
H	6.262565	1.190673	0.910670
H	5.869724	-0.139818	-0.192832
C	1.946098	-3.810348	-0.516523
H	2.041000	-3.154677	-1.392724
C	0.533302	-3.627932	0.040596
H	-0.221968	-3.892871	-0.708495
H	0.351641	-2.587334	0.359035
H	0.379846	-4.270312	0.920097
C	2.155373	-5.243814	-0.993334
H	3.175315	-5.416365	-1.362158
H	1.455818	-5.477102	-1.806759
H	1.967414	-5.972162	-0.194085
C	1.970875	2.559987	-2.038974
C	0.755761	2.831661	-2.701143
C	0.373643	4.167592	-2.851138
H	-0.557737	4.402440	-3.364621
C	1.160809	5.203777	-2.361959
H	0.847561	6.237760	-2.499013
C	2.332891	4.914700	-1.675676
H	2.928787	5.727694	-1.261850
C	2.753813	3.595394	-1.490443
C	-0.079793	1.717103	-3.302761
H	0.049087	0.827975	-2.662331
C	-1.570718	2.030679	-3.352949
H	-1.802123	2.843546	-4.053972
H	-1.969230	2.313991	-2.370221
H	-2.134086	1.152379	-3.694918
C	0.408979	1.358715	-4.707183
H	0.399909	2.242439	-5.360212
H	-0.246820	0.603994	-5.160834
H	1.426410	0.951876	-4.704357
C	3.984984	3.308579	-0.652072
H	4.305006	2.273344	-0.844422
C	3.627224	3.408814	0.833361
H	3.282931	4.421856	1.083781
H	4.498387	3.181827	1.462197

H	2.820493	2.712101	1.106850
C	5.163458	4.219903	-0.983402
H	5.405678	4.208829	-2.053655
H	6.057593	3.904514	-0.432262
H	4.965512	5.262072	-0.702170
Al	-1.913118	-0.043178	-0.147935
N	-3.329299	1.312587	-0.004358
N	-2.945375	-1.060239	-1.484737
C	-4.298917	1.536348	-0.894930
C	-4.544046	0.679231	-1.976837
H	-5.336960	0.976073	-2.656951
C	-3.973856	-0.579838	-2.201338
C	-5.185383	2.736809	-0.738288
H	-4.583905	3.653426	-0.681626
H	-5.753532	2.694197	0.198786
H	-5.889723	2.826484	-1.569517
C	-4.576603	-1.418057	-3.292153
H	-3.845308	-1.615177	-4.086762
H	-5.444348	-0.924292	-3.736988
H	-4.885402	-2.400802	-2.914375
C	-3.351394	2.066013	1.224505
C	-4.144681	1.579703	2.284332
C	-4.173647	2.300769	3.479355
H	-4.786371	1.934408	4.303506
C	-3.432513	3.464166	3.637856
H	-3.465461	4.012354	4.577792
C	-2.635280	3.911474	2.594511
H	-2.030231	4.809201	2.724541
C	-2.572302	3.226771	1.377319
C	-4.950266	0.298560	2.185300
H	-4.782073	-0.144420	1.193117
C	-4.480454	-0.713693	3.228395
H	-4.611783	-0.327330	4.247359
H	-3.417028	-0.956554	3.101239
H	-5.061610	-1.642586	3.154779
C	-6.450126	0.557177	2.322051
H	-7.017173	-0.377478	2.223840
H	-6.820053	1.252406	1.557954
H	-6.692176	0.987332	3.302931
C	-1.650666	3.749600	0.297339
H	-1.745726	3.096477	-0.581702
C	-2.018358	5.168458	-0.133245
H	-3.054538	5.240869	-0.489517
H	-1.356430	5.504374	-0.941485
H	-1.908858	5.878401	0.697281
C	-0.196852	3.680446	0.756601
H	0.471626	4.077145	-0.015847
H	0.097856	2.641711	0.968696
H	-0.039005	4.267484	1.672791
C	-2.595692	-2.444519	-1.679954
C	-1.792875	-2.846762	-2.764626
C	-1.587478	-4.214004	-2.971048
H	-0.968593	-4.536502	-3.808506
C	-2.138925	-5.162533	-2.121996
H	-1.977482	-6.223607	-2.304812
C	-2.857884	-4.746511	-1.008773
H	-3.243192	-5.490498	-0.314454
C	-3.083803	-3.391666	-0.753147
C	-1.062018	-1.859159	-3.650947

H	-1.469130	-0.854875	-3.460576
C	0.416658	-1.844525	-3.253476
H	0.868012	-2.836633	-3.399661
H	0.531066	-1.586328	-2.189132
H	0.992940	-1.123693	-3.847982
C	-1.215651	-2.163215	-5.139442
H	-0.717899	-1.397660	-5.747186
H	-2.267743	-2.205127	-5.448347
H	-0.759430	-3.125050	-5.405185
C	-3.842300	-2.968304	0.491036
H	-3.449230	-1.984270	0.799963
C	-3.607897	-3.910181	1.666541
H	-4.070776	-4.893017	1.510185
H	-4.045428	-3.493202	2.579912
H	-2.536526	-4.060152	1.847971
C	-5.335033	-2.793706	0.211512
H	-5.775494	-3.729370	-0.159619
H	-5.525956	-2.010971	-0.534456
H	-5.871252	-2.512965	1.127937
H	-0.806483	-0.630619	2.558315
C	0.798155	-0.496704	3.202543
C	1.312587	-1.752774	3.556952
C	0.908242	0.553696	4.140801
C	1.949989	-1.965179	4.778530
C	1.539115	0.326268	5.367542
C	2.063879	-0.922806	5.694721
H	2.548061	-1.079968	6.657987
H	2.357351	-2.949983	5.011182
H	1.621127	1.151585	6.078700
H	1.230750	-2.583366	2.851863
C	0.392156	1.915939	3.798072
H	-0.585198	1.857883	3.294349
H	0.291268	2.561880	4.680977
H	1.067312	2.422857	3.090298

TS-1a-toluene_para.log

SCF (wB97x) = -2881.98670334
H(0 K)= -2880.581077
H(298 K)= -2880.500957
G(298 K)= -2880.693631
Lowest Frequency = -720.6193cm-1

Pd	-0.071765	0.935464	-0.807527
Al	-1.720034	-0.410863	0.378218
N	-2.843336	-1.693714	-0.607384
N	-2.290076	-1.185554	2.106319
C	-3.405543	-2.808116	-0.130837
C	-3.438434	-3.114016	1.237297
H	-3.960571	-4.025233	1.514335
C	-3.027805	-2.289525	2.290213
C	-4.091219	-3.760701	-1.066552
H	-4.854062	-3.247627	-1.665342
H	-4.563574	-4.582786	-0.522143
H	-3.376359	-4.183998	-1.783394
C	-3.526668	-2.644853	3.663337
H	-2.790687	-2.452587	4.450121
H	-3.837875	-3.692613	3.708023
H	-4.404354	-2.029499	3.907116

C	-3.088379	-1.351676	-1.986246
C	-4.089861	-0.395024	-2.258466
C	-4.390583	-0.120204	-3.593471
H	-5.157912	0.615714	-3.826492
C	-3.704633	-0.740530	-4.630538
H	-3.954397	-0.508905	-5.664913
C	-2.672434	-1.621627	-4.341411
H	-2.099831	-2.070099	-5.153638
C	-2.339539	-1.939481	-3.021767
C	-4.814497	0.327457	-1.136898
H	-4.083073	0.471435	-0.320478
C	-5.292512	1.717949	-1.537207
H	-4.477948	2.314692	-1.965573
H	-5.681682	2.250294	-0.660981
H	-6.110209	1.675706	-2.269448
C	-5.967429	-0.499684	-0.566978
H	-6.711603	-0.718287	-1.345242
H	-6.477990	0.049935	0.235888
H	-5.627912	-1.455003	-0.147439
C	-1.142392	-2.824278	-2.748501
H	-1.144535	-3.080022	-1.680248
C	0.142496	-2.046128	-3.034513
H	1.026237	-2.649319	-2.789131
H	0.189823	-1.113359	-2.449936
H	0.201435	-1.774819	-4.098745
C	-1.156684	-4.129856	-3.540277
H	-2.076852	-4.708378	-3.384714
H	-0.306704	-4.759662	-3.246749
H	-1.065205	-3.948474	-4.619197
C	-2.044727	-0.358764	3.255232
C	-0.818690	-0.471670	3.941696
C	-0.642702	0.290656	5.099471
H	0.286258	0.199483	5.661115
C	-1.627573	1.162682	5.548511
H	-1.467244	1.747261	6.452924
C	-2.805533	1.301575	4.826502
H	-3.561011	2.012437	5.160531
C	-3.036952	0.551553	3.670739
C	0.254021	-1.444435	3.489211
H	0.126708	-1.593732	2.404862
C	1.674042	-0.933636	3.706379
H	1.914286	-0.790922	4.768475
H	1.851991	0.020495	3.194669
H	2.400577	-1.658201	3.314702
C	0.083174	-2.803085	4.170050
H	0.106413	-2.698526	5.263601
H	0.896947	-3.482415	3.884147
H	-0.862849	-3.287297	3.900033
C	-4.299718	0.786758	2.864097
H	-4.416267	-0.040920	2.147857
C	-4.161675	2.079004	2.054575
H	-4.002822	2.939646	2.718886
H	-5.068900	2.269694	1.466596
H	-3.310018	2.040122	1.358818
C	-5.558683	0.825846	3.726581
H	-5.655020	-0.067112	4.357071
H	-6.453309	0.891347	3.095579
H	-5.570707	1.699324	4.390923
Al	1.996411	0.044130	0.036463

N	3.186176	1.291045	1.007032
N	3.261762	-1.435631	0.267480
C	4.243722	0.964678	1.748341
C	4.677977	-0.364321	1.900049
H	5.500908	-0.524801	2.590897
C	4.271158	-1.474079	1.154473
C	5.066216	2.032048	2.409607
H	5.815205	2.419518	1.705756
H	5.607082	1.631239	3.272419
H	4.459301	2.886903	2.723983
C	5.022530	-2.758384	1.355432
H	4.353184	-3.542321	1.734224
H	5.843596	-2.633172	2.066199
H	5.430359	-3.137183	0.410027
C	2.918567	2.675196	0.715679
C	3.701683	3.342335	-0.245399
C	3.371818	4.660392	-0.572086
H	3.963980	5.183023	-1.323675
C	2.293318	5.300151	0.019349
H	2.045861	6.324313	-0.254139
C	1.517799	4.619550	0.949541
H	0.660851	5.118604	1.398342
C	1.802194	3.300975	1.308620
C	4.818787	2.664052	-1.014032
H	4.999909	1.669758	-0.578737
C	4.390220	2.460824	-2.467666
H	4.212170	3.426604	-2.958467
H	3.457281	1.885616	-2.539014
H	5.171430	1.938339	-3.035971
C	6.131586	3.443381	-0.957079
H	6.935372	2.884208	-1.452685
H	6.449370	3.651225	0.072245
H	6.045085	4.409957	-1.469920
C	0.963619	2.596525	2.354552
H	0.930370	1.526173	2.092312
C	1.614159	2.728702	3.731963
H	2.605127	2.256504	3.764578
H	0.993031	2.253997	4.501637
H	1.739404	3.786490	4.002168
C	-0.486703	3.057867	2.386100
H	-1.065519	2.411065	3.056567
H	-0.938273	2.996192	1.386521
H	-0.593623	4.086734	2.757477
C	3.118706	-2.538749	-0.646566
C	2.383065	-3.688599	-0.302555
C	2.307989	-4.733260	-1.229433
H	1.734304	-5.625168	-0.976037
C	2.946052	-4.656902	-2.458153
H	2.887618	-5.487345	-3.159914
C	3.631735	-3.498394	-2.801467
H	4.095235	-3.425223	-3.783191
C	3.710771	-2.414235	-1.924720
C	1.628030	-3.827659	1.003782
H	1.908489	-2.985542	1.655125
C	0.122480	-3.737352	0.748733
H	-0.210999	-4.532031	0.065133
H	-0.139283	-2.773944	0.285560
H	-0.452133	-3.836247	1.678932
C	1.964376	-5.129785	1.730108

H	1.449355	-5.182053	2.697146
H	3.040684	-5.237311	1.913935
H	1.645317	-6.005647	1.151246
C	4.404235	-1.135070	-2.358071
H	3.893395	-0.297170	-1.853629
C	4.282266	-0.884748	-3.856944
H	4.868254	-1.602862	-4.444726
H	4.658511	0.112745	-4.107698
H	3.238466	-0.943069	-4.188822
C	5.867968	-1.093903	-1.917681
H	6.428317	-1.937819	-2.343078
H	5.972611	-1.133783	-0.826416
H	6.348675	-0.167346	-2.260058
H	0.599640	1.920977	-1.900371
C	-1.089599	2.352942	-1.979319
C	-1.691618	1.965762	-3.189229
C	-1.358830	3.653428	-1.525599
C	-2.564810	2.809777	-3.869342
H	-1.496806	0.970195	-3.595021
C	-2.237167	4.495011	-2.207050
H	-0.875249	4.017826	-0.616781
C	-2.864801	4.089641	-3.388149
H	-3.036850	2.460777	-4.791654
H	-2.440634	5.493976	-1.813894
C	-3.846253	4.972985	-4.098734
H	-4.880418	4.617281	-3.973554
H	-3.813271	6.001422	-3.719832
H	-3.659937	5.011924	-5.180037

TS-1a_TS1-PdAl2.log

SCF (wB97x) = -2842.67632580
H(0 K)= -2841.298181
H(298 K)= -2841.219929
G(298 K)= -2841.408364
Lowest Frequency = -728.6078cm-1

Pd	-0.009836	-0.318570	1.292626
Al	-1.817995	0.042909	-0.296089
N	-2.968974	1.631269	-0.135847
N	-2.541101	-0.344283	-2.095407
C	-3.638710	2.247086	-1.114055
C	-3.774874	1.700321	-2.398200
H	-4.378195	2.267306	-3.101242
C	-3.366511	0.428977	-2.815065
C	-4.336825	3.547445	-0.840582
H	-5.016061	3.459029	0.016458
H	-4.907131	3.883814	-1.710609
H	-3.613753	4.329443	-0.576174
C	-3.968736	-0.091025	-4.090913
H	-3.268820	-0.687347	-4.684315
H	-4.355011	0.728406	-4.704058
H	-4.813892	-0.753525	-3.855863
C	-3.103775	2.147100	1.203552
C	-4.007653	1.496338	2.070420
C	-4.203686	2.037312	3.342239
H	-4.894286	1.550470	4.028049
C	-3.508618	3.165058	3.761817
H	-3.676299	3.569355	4.759014

C	-2.569950	3.746041	2.921167
H	-1.986915	4.599543	3.268013
C	-2.343031	3.248565	1.635008
C	-4.739582	0.236926	1.642927
H	-4.044559	-0.339493	1.004634
C	-5.098902	-0.667965	2.814661
H	-4.228087	-0.872492	3.449466
H	-5.485570	-1.626079	2.447147
H	-5.886343	-0.230070	3.442675
C	-5.976411	0.546110	0.798458
H	-6.690514	1.159511	1.365137
H	-6.490640	-0.381463	0.511334
H	-5.728171	1.084505	-0.124774
C	-1.234667	3.842396	0.792674
H	-1.321220	3.434380	-0.223495
C	0.117971	3.401428	1.352705
H	0.939462	3.771099	0.725646
H	0.193382	2.303535	1.407248
H	0.262570	3.795449	2.369311
C	-1.294288	5.364492	0.689860
H	-2.261401	5.724934	0.315203
H	-0.510891	5.728263	0.012022
H	-1.124497	5.841791	1.663869
C	-2.300207	-1.676037	-2.577014
C	-1.128478	-1.945741	-3.313174
C	-0.962645	-3.231625	-3.834365
H	-0.078232	-3.454966	-4.429904
C	-1.902599	-4.230528	-3.609678
H	-1.751504	-5.225282	-4.025606
C	-3.022148	-3.960267	-2.833923
H	-3.739975	-4.754212	-2.628785
C	-3.242252	-2.687322	-2.301843
C	-0.108437	-0.860594	-3.602729
H	-0.188547	-0.110773	-2.799333
C	1.332045	-1.360943	-3.606597
H	1.524300	-2.090254	-4.404873
H	1.608101	-1.831320	-2.654490
H	2.023105	-0.523727	-3.773320
C	-0.416042	-0.159399	-4.926486
H	-0.440690	-0.880680	-5.755001
H	0.357163	0.585238	-5.157020
H	-1.381164	0.360727	-4.906590
C	-4.435012	-2.448530	-1.395828
H	-4.570667	-1.362733	-1.276767
C	-4.155858	-3.032553	-0.008234
H	-3.971600	-4.113959	-0.070904
H	-5.011903	-2.874661	0.660635
H	-3.271549	-2.576110	0.461217
C	-5.735425	-3.012491	-1.962743
H	-5.934167	-2.650248	-2.979295
H	-6.584648	-2.726147	-1.330538
H	-5.720896	-4.109172	-2.002348
Al	1.934680	-0.029316	-0.092566
N	3.156384	-1.574761	-0.265285
N	3.073149	1.076256	-1.242911
C	4.146436	-1.709175	-1.146470
C	4.473405	-0.703670	-2.073682
H	5.244291	-0.950570	-2.798222
C	4.027358	0.620795	-2.072969

C	5.008990	-2.937465	-1.137045
H	4.447203	-3.835594	-0.861654
H	5.810165	-2.827994	-0.393581
H	5.485696	-3.089242	-2.110270
C	4.671314	1.570322	-3.041407
H	3.927614	1.967784	-3.744924
H	5.461221	1.079515	-3.615993
H	5.098776	2.439901	-2.526918
C	3.005786	-2.541633	0.790862
C	3.885634	-2.507426	1.889288
C	3.671036	-3.402827	2.940485
H	4.339140	-3.377143	3.801666
C	2.612497	-4.297981	2.919000
H	2.455759	-4.982092	3.751082
C	1.740810	-4.303168	1.837353
H	0.899859	-4.994325	1.831725
C	1.908248	-3.427209	0.763507
C	4.989338	-1.477434	2.029838
H	5.078197	-0.917586	1.086664
C	4.620406	-0.478549	3.127142
H	4.531795	-0.983621	4.097893
H	3.655603	0.006688	2.926649
H	5.391938	0.296939	3.224943
C	6.349290	-2.106679	2.327336
H	7.134886	-1.340544	2.342240
H	6.630214	-2.864377	1.585229
H	6.356376	-2.597500	3.309043
C	0.965386	-3.481934	-0.420112
H	0.868218	-2.458483	-0.817561
C	1.548712	-4.366340	-1.522489
H	2.499495	-3.971274	-1.904452
H	0.854313	-4.440789	-2.368643
H	1.735917	-5.382003	-1.147016
C	-0.446825	-3.918152	-0.056346
H	-1.105489	-3.797849	-0.924936
H	-0.849228	-3.301164	0.758658
H	-0.497807	-4.974129	0.243951
C	2.900524	2.500653	-1.122885
C	2.063572	3.217122	-1.999182
C	1.963938	4.603184	-1.840708
H	1.312214	5.166520	-2.509104
C	2.674668	5.271259	-0.855261
H	2.594913	6.352782	-0.758001
C	3.462542	4.545984	0.029842
H	3.984953	5.068664	0.828455
C	3.571343	3.156923	-0.064891
C	1.229983	2.552979	-3.076279
H	1.531155	1.496113	-3.141909
C	-0.250339	2.592158	-2.692670
H	-0.599728	3.627623	-2.567030
H	-0.423302	2.069359	-1.739887
H	-0.878425	2.120489	-3.459596
C	1.437976	3.198156	-4.446328
H	0.867372	2.668125	-5.218738
H	2.492759	3.202206	-4.748296
H	1.094568	4.240301	-4.454880
C	4.379871	2.385656	0.963106
H	3.903271	1.397521	1.082464
C	4.362468	3.049192	2.335652

H	4.928435	3.989435	2.347187
H	4.822907	2.392442	3.081385
H	3.338041	3.263287	2.663950
C	5.815181	2.135156	0.499193
H	6.335221	3.083693	0.306890
H	5.854187	1.535592	-0.418617
H	6.381612	1.594146	1.269361
H	0.799189	-0.467729	2.685325
C	-0.831536	-0.822819	3.161767
C	-1.377166	0.176871	3.985920
C	-1.013163	-2.157289	3.563411
C	-2.112267	-0.143023	5.126247
H	-1.243480	1.227467	3.718084
C	-1.749953	-2.479889	4.702011
H	-0.567983	-2.960640	2.972610
C	-2.306799	-1.474665	5.492110
H	-2.541015	0.658640	5.730438
H	-1.884842	-3.526704	4.978397
H	-2.877257	-1.725803	6.385611

TS-1b-toluene_meta1.log

SCF (wB97x) = -2687.83041724
H(0 K)= -2686.579965
H(298 K)= -2686.515437
G(298 K)= -2686.678918
Lowest Frequency = -808.3652cm-1

Pd	0.888853	0.090196	-1.012700
P	2.000603	-0.389572	1.031822
C	2.063591	1.108427	2.133453
H	2.712058	0.887541	3.000215
C	0.689306	1.523287	2.667369
H	0.009865	1.691015	1.811934
H	0.242196	0.712369	3.262931
C	0.780506	2.796072	3.504473
H	-0.220815	3.087070	3.854692
H	1.380997	2.596064	4.407661
C	1.413984	3.936970	2.718129
H	1.484910	4.840894	3.339250
H	0.757021	4.190391	1.870228
C	2.781264	3.537497	2.179772
H	3.476302	3.367064	3.019224
H	3.214118	4.350547	1.580005
C	2.678239	2.270198	1.341862
H	3.661037	1.995892	0.930130
H	2.030455	2.459065	0.467998
C	3.811717	-0.745792	0.748471
H	4.043047	-0.035921	-0.067187
C	4.818600	-0.475751	1.871003
H	4.656367	-1.171990	2.708361
H	4.689775	0.535351	2.281305
C	6.244890	-0.636255	1.349149
H	6.423806	0.124690	0.570681
H	6.968071	-0.437399	2.152161
C	6.468153	-2.021488	0.754343
H	6.367906	-2.775778	1.552860
H	7.492715	-2.114563	0.369421
C	5.454588	-2.314109	-0.345537

H	5.597641	-3.328354	-0.743586
H	5.614409	-1.621051	-1.186414
C	4.025980	-2.144045	0.159006
H	3.305493	-2.323323	-0.654226
H	3.825516	-2.904135	0.933139
C	1.288393	-1.722723	2.117992
H	0.358296	-1.250585	2.486241
C	0.846056	-2.951423	1.310713
H	0.187434	-2.630656	0.486805
H	1.719549	-3.426226	0.838190
C	0.139775	-3.974389	2.195004
H	-0.121067	-4.861857	1.603070
H	-0.816692	-3.549506	2.541018
C	0.980986	-4.359034	3.406198
H	0.441733	-5.078439	4.037489
H	1.896616	-4.871083	3.066391
C	1.373291	-3.126697	4.211609
H	1.985554	-3.406189	5.079949
H	0.463388	-2.649095	4.612337
C	2.120400	-2.125175	3.336881
H	3.060837	-2.587383	2.996503
H	2.406903	-1.237701	3.920885
Al	-1.518742	0.151333	-0.712184
N	-2.815755	1.632844	-0.641574
N	-2.930294	-1.089617	-1.291117
C	-3.964910	1.686800	-1.328515
C	-4.511730	0.576819	-1.986523
H	-5.436346	0.744643	-2.530584
C	-4.079544	-0.752044	-1.882931
C	-4.739901	2.971573	-1.381313
H	-4.966743	3.338470	-0.372403
H	-5.677543	2.847982	-1.929242
H	-4.154903	3.762703	-1.867041
C	-4.978738	-1.824561	-2.423091
H	-5.789624	-1.400916	-3.021471
H	-5.425231	-2.404776	-1.604971
H	-4.419782	-2.544196	-3.032957
C	-2.456599	2.777908	0.151625
C	-2.745612	2.750463	1.534071
C	-2.470972	3.893787	2.288092
H	-2.697368	3.900094	3.352637
C	-1.900588	5.020787	1.708112
H	-1.692741	5.900647	2.315153
C	-1.557583	5.001829	0.364228
H	-1.063252	5.866576	-0.078598
C	-1.818206	3.885986	-0.436131
C	-3.327640	1.511835	2.192138
H	-2.852806	0.638373	1.709083
C	-3.011355	1.426848	3.680886
H	-1.942168	1.568204	3.880312
H	-3.300826	0.446428	4.076079
H	-3.564027	2.178528	4.259404
C	-4.836418	1.385382	1.972082
H	-5.360916	2.266210	2.367201
H	-5.229244	0.503374	2.496233
H	-5.098907	1.280454	0.912998
C	-1.332608	3.875451	-1.870758
H	-1.764388	3.000327	-2.377536
C	0.187422	3.705914	-1.878855

H	0.576220	3.632213	-2.902590
H	0.479524	2.786780	-1.349658
H	0.678686	4.557714	-1.385114
C	-1.740511	5.122343	-2.651917
H	-2.824997	5.291068	-2.635304
H	-1.430503	5.035448	-3.699906
H	-1.264243	6.025442	-2.249119
C	-2.679255	-2.477929	-1.020780
C	-1.855491	-3.231821	-1.877641
C	-1.626524	-4.574425	-1.560686
H	-0.997759	-5.171151	-2.221861
C	-2.180236	-5.156573	-0.429110
H	-1.989350	-6.204258	-0.201864
C	-2.966464	-4.389351	0.421465
H	-3.378513	-4.837410	1.326643
C	-3.227624	-3.045758	0.148531
C	-1.215661	-2.650267	-3.120843
H	-1.448192	-1.576633	-3.160085
C	0.304412	-2.780840	-3.065500
H	0.620517	-3.833379	-3.061923
H	0.706840	-2.293318	-2.163799
H	0.761660	-2.294297	-3.936033
C	-1.766582	-3.300794	-4.389497
H	-1.548322	-4.377072	-4.409576
H	-1.312210	-2.854897	-5.282546
H	-2.854666	-3.185968	-4.475017
C	-4.048601	-2.236808	1.134908
H	-4.214048	-1.234840	0.713144
C	-3.281285	-2.061280	2.445005
H	-3.088693	-3.031908	2.922020
H	-3.854766	-1.450899	3.155285
H	-2.308024	-1.574085	2.283333
C	-5.417144	-2.860443	1.401837
H	-5.993493	-2.999386	0.478984
H	-6.009075	-2.226164	2.073548
H	-5.324184	-3.844357	1.879720
H	0.887270	0.516708	-2.579411
C	2.545413	0.441486	-2.261640
C	3.201668	1.679434	-2.164699
C	3.232512	-0.593648	-2.918105
C	4.509708	1.880742	-2.618980
H	2.696185	2.519278	-1.683829
C	4.530118	-0.406920	-3.391830
H	2.762170	-1.570036	-3.035771
C	5.176601	0.816642	-3.232933
H	5.050245	-1.234549	-3.876417
H	6.197615	0.951628	-3.591876
C	5.168284	3.219482	-2.456341
H	4.908465	3.902519	-3.277262
H	4.856622	3.711714	-1.525801
H	6.261652	3.137174	-2.443279

TS-1b-toluene_meta2.log

SCF (wB97x) = -2687.83109158
H(0 K)= -2686.580527
H(298 K)= -2686.515136
G(298 K)= -2686.681284
Lowest Frequency = -783.9150cm-1

Pd	0.883795	0.215926	-0.976861
P	1.984430	0.148632	1.128264
C	1.768589	1.748301	2.052281
H	2.406297	1.729457	2.954291
C	0.325440	1.994968	2.502460
H	-0.336145	1.959287	1.617915
H	-0.014609	1.192421	3.175258
C	0.183404	3.349036	3.191795
H	-0.864384	3.513727	3.483215
H	0.770136	3.346238	4.125653
C	0.664046	4.483171	2.295535
H	0.567604	5.449408	2.810339
H	0.012011	4.534433	1.408458
C	2.098141	4.249870	1.840074
H	2.774239	4.282502	2.711109
H	2.425153	5.052391	1.164669
C	2.230338	2.899334	1.148691
H	3.261847	2.741951	0.801168
H	1.603745	2.885777	0.239395
C	3.838840	0.060675	0.923612
H	3.985631	0.706302	0.037840
C	4.746074	0.608125	2.030432
H	4.662806	-0.012207	2.936116
H	4.443240	1.622805	2.323147
C	6.199740	0.625992	1.562328
H	6.287755	1.320340	0.709860
H	6.849199	1.021353	2.355552
C	6.663204	-0.758899	1.126798
H	6.652235	-1.433791	1.999095
H	7.703781	-0.724136	0.776748
C	5.751770	-1.321176	0.042797
H	6.072118	-2.331221	-0.249536
H	5.827505	-0.693825	-0.859463
C	4.297028	-1.339030	0.499379
H	3.644913	-1.722704	-0.300709
H	4.195812	-2.033449	1.350776
C	1.453372	-1.155719	2.345230
H	0.447358	-0.803018	2.640377
C	1.242305	-2.520790	1.674959
H	0.572127	-2.403422	0.807529
H	2.197443	-2.896190	1.276465
C	0.674967	-3.542033	2.656145
H	0.581036	-4.519374	2.164181
H	-0.349398	-3.242777	2.932180
C	1.519814	-3.650526	3.919691
H	1.077687	-4.373936	4.618306
H	2.517208	-4.042125	3.659095
C	1.681383	-2.290054	4.586229
H	2.296769	-2.369803	5.492843
H	0.692908	-1.924242	4.911416
C	2.293436	-1.283681	3.617293
H	3.306529	-1.624312	3.349324
H	2.415805	-0.303270	4.101573
Al	-1.512919	-0.082260	-0.730813
N	-3.026874	1.171334	-0.862754
N	-2.692059	-1.591113	-1.182768
C	-4.144614	0.962978	-1.571298
C	-4.488224	-0.288563	-2.100082

H	-5.407608	-0.334154	-2.676034
C	-3.858311	-1.510654	-1.829981
C	-5.106369	2.094288	-1.792234
H	-5.421451	2.537492	-0.839241
H	-5.994328	1.761082	-2.335578
H	-4.633860	2.904500	-2.361706
C	-4.560470	-2.767567	-2.251606
H	-5.408781	-2.550151	-2.905914
H	-4.935230	-3.314264	-1.376348
H	-3.876732	-3.453637	-2.765465
C	-2.876948	2.443267	-0.207708
C	-3.209818	2.532359	1.162241
C	-3.140603	3.784357	1.777542
H	-3.405093	3.879639	2.828944
C	-2.726980	4.911022	1.076605
H	-2.678368	5.877100	1.576788
C	-2.334670	4.789222	-0.248259
H	-1.960905	5.662566	-0.782963
C	-2.392339	3.560799	-0.912827
C	-3.619824	1.303210	1.954213
H	-3.000077	0.464261	1.588384
C	-3.348744	1.442116	3.447845
H	-2.320677	1.767169	3.648420
H	-3.502967	0.482083	3.953522
H	-4.028227	2.162991	3.920892
C	-5.082614	0.918066	1.724841
H	-5.749448	1.746453	2.000846
H	-5.354544	0.053377	2.345600
H	-5.287885	0.649773	0.682084
C	-1.856997	3.459175	-2.326053
H	-2.124004	2.471261	-2.728321
C	-0.330175	3.540949	-2.286906
H	0.102035	3.412122	-3.287479
H	0.086299	2.750866	-1.644467
H	-0.000728	4.513904	-1.892870
C	-2.429347	4.521256	-3.261977
H	-3.526657	4.510552	-3.286884
H	-2.069045	4.363554	-4.285350
H	-2.120336	5.531336	-2.963760
C	-2.238947	-2.882433	-0.745579
C	-1.287583	-3.595309	-1.498994
C	-0.864229	-4.839461	-1.021222
H	-0.134008	-5.404923	-1.600620
C	-1.351939	-5.362956	0.167923
H	-1.008611	-6.333930	0.521553
C	-2.269520	-4.632810	0.913005
H	-2.632742	-5.030330	1.861641
C	-2.726500	-3.387812	0.477930
C	-0.709885	-3.070346	-2.796433
H	-1.107563	-2.060256	-2.968592
C	0.808943	-2.948280	-2.705210
H	1.286461	-3.929743	-2.576376
H	1.098938	-2.306955	-1.858828
H	1.213183	-2.493369	-3.618056
C	-1.110040	-3.945826	-3.983579
H	-0.723412	-4.967625	-3.870286
H	-0.703471	-3.540815	-4.918202
H	-2.199214	-4.018762	-4.096595
C	-3.692995	-2.609284	1.350156

H	-3.999089	-1.702255	0.809162
C	-3.003979	-2.163329	2.639412
H	-2.676619	-3.028988	3.231272
H	-3.687967	-1.574095	3.264587
H	-2.114459	-1.549468	2.432810
C	-4.955622	-3.406360	1.671931
H	-5.473366	-3.741150	0.764722
H	-5.660284	-2.800037	2.254767
H	-4.725872	-4.300798	2.265465
H	0.872908	0.438264	-2.585146
C	2.510201	0.655059	-2.237353
C	2.979230	1.977942	-2.327450
C	3.356645	-0.354117	-2.722562
C	4.256513	2.257376	-2.810310
H	2.351618	2.802037	-1.986398
C	4.636984	-0.091603	-3.222507
H	3.027719	-1.394329	-2.686777
C	5.089329	1.231086	-3.249446
H	6.089379	1.454342	-3.623191
H	4.607487	3.289721	-2.839801
C	5.500911	-1.213218	-3.720793
H	5.393483	-1.357379	-4.805006
H	6.564866	-1.025292	-3.529235
H	5.238173	-2.165504	-3.243293

TS-1b-toluene_ortho1.log

SCF (wB97x) = -2687.83059192
H(0 K)= -2686.579641
H(298 K)= -2686.514302
G(298 K)= -2686.680649
Lowest Frequency = -713.0617cm-1

Pd	0.552289	-0.668344	-0.299544
P	2.441774	0.679826	0.217347
C	2.640835	2.300563	-0.675133
H	3.637748	2.701100	-0.418773
C	1.607641	3.358098	-0.280973
H	0.591745	2.975759	-0.491426
H	1.644639	3.557907	0.799464
C	1.847960	4.655885	-1.046822
H	1.105641	5.409112	-0.750253
H	2.833409	5.062524	-0.763380
C	1.814406	4.430351	-2.552680
H	2.004187	5.369744	-3.090568
H	0.801520	4.102820	-2.838898
C	2.816904	3.360704	-2.966665
H	3.840440	3.724849	-2.776091
H	2.754497	3.167184	-4.046336
C	2.594411	2.068942	-2.188709
H	3.335858	1.314112	-2.490466
H	1.607166	1.644477	-2.441205
C	4.013847	-0.214764	-0.251913
H	3.728336	-0.662190	-1.223264
C	5.304862	0.577734	-0.478112
H	5.646544	1.018332	0.472170
H	5.143127	1.418400	-1.167077
C	6.394308	-0.334489	-1.040929
H	6.079316	-0.681258	-2.038951

H	7.323335	0.233030	-1.190202
C	6.642395	-1.545607	-0.148896
H	7.059792	-1.203659	0.812997
H	7.401118	-2.201122	-0.597602
C	5.351472	-2.312659	0.113195
H	5.532778	-3.157910	0.791262
H	4.973486	-2.746871	-0.826279
C	4.282238	-1.392832	0.689679
H	3.354079	-1.950504	0.876173
H	4.628097	-1.003630	1.662772
C	2.553895	1.188685	2.001664
H	1.743637	1.936940	2.061757
C	2.145708	0.065164	2.959546
H	1.215191	-0.395756	2.596696
H	2.906577	-0.729851	2.957564
C	1.978263	0.596905	4.378552
H	1.714583	-0.221060	5.063284
H	1.131980	1.304051	4.399181
C	3.239586	1.307766	4.858372
H	3.094354	1.714760	5.868268
H	4.058181	0.573470	4.936200
C	3.656887	2.412369	3.893228
H	4.579034	2.899488	4.238594
H	2.878498	3.194719	3.878416
C	3.836068	1.871975	2.476408
H	4.664037	1.144165	2.467729
H	4.128281	2.681788	1.791259
Al	-1.684492	0.049245	0.352889
N	-3.038013	1.465158	0.207242
N	-3.098910	-1.303293	0.637070
C	-4.344709	1.388234	0.495245
C	-4.959808	0.200133	0.898927
H	-6.016319	0.260321	1.142159
C	-4.388028	-1.079747	0.915729
C	-5.206726	2.610125	0.363320
H	-4.864665	3.413621	1.028260
H	-6.250617	2.387464	0.598686
H	-5.155183	3.018167	-0.653726
C	-5.293356	-2.234852	1.235510
H	-6.328566	-1.905877	1.359010
H	-4.977000	-2.737023	2.158909
H	-5.255087	-2.997750	0.448576
C	-2.536438	2.700826	-0.325615
C	-2.182824	3.754059	0.541991
C	-1.697505	4.936182	-0.019435
H	-1.414195	5.760833	0.634523
C	-1.557875	5.075593	-1.395635
H	-1.176462	6.005531	-1.815554
C	-1.881578	4.016604	-2.232088
H	-1.750448	4.120782	-3.309737
C	-2.370642	2.811845	-1.719516
C	-2.260986	3.599937	2.048307
H	-2.940601	2.764921	2.270358
C	-0.886673	3.224146	2.607163
H	-0.496479	2.312971	2.128972
H	-0.934290	3.051703	3.690395
H	-0.159628	4.028630	2.424552
C	-2.809959	4.838672	2.749887
H	-2.125081	5.692056	2.667757

H	-2.949774	4.643062	3.819624
H	-3.776172	5.152295	2.334639
C	-2.682646	1.672416	-2.671219
H	-3.148902	0.858862	-2.094226
C	-1.396639	1.121969	-3.289869
H	-1.616890	0.290043	-3.972601
H	-0.705353	0.748751	-2.516764
H	-0.868957	1.896498	-3.865548
C	-3.678438	2.083770	-3.753638
H	-4.612918	2.466544	-3.325072
H	-3.926565	1.227943	-4.393474
H	-3.267711	2.867591	-4.403279
C	-2.668313	-2.672536	0.526960
C	-2.535891	-3.229782	-0.762697
C	-2.110339	-4.555965	-0.860175
H	-1.982905	-5.006244	-1.842368
C	-1.811431	-5.303350	0.272853
H	-1.471684	-6.332528	0.170392
C	-1.912026	-4.724247	1.529660
H	-1.644893	-5.304133	2.412629
C	-2.334418	-3.400991	1.681937
C	-2.800835	-2.401397	-2.008530
H	-2.425969	-1.383877	-1.797804
C	-2.026546	-2.898388	-3.223441
H	-2.417461	-3.854209	-3.596932
H	-0.961202	-3.033044	-2.993793
H	-2.110796	-2.177082	-4.045936
C	-4.289506	-2.277381	-2.332740
H	-4.742336	-3.265421	-2.493005
H	-4.433670	-1.692783	-3.251506
H	-4.849372	-1.773639	-1.535451
C	-2.334560	-2.753474	3.052252
H	-2.978514	-1.863051	3.011642
C	-0.920496	-2.273090	3.383656
H	-0.232657	-3.124299	3.482811
H	-0.899105	-1.706443	4.324148
H	-0.524845	-1.632079	2.582266
C	-2.876857	-3.659937	4.152585
H	-3.874102	-4.049182	3.910485
H	-2.948547	-3.112835	5.100230
H	-2.222534	-4.522494	4.330599
H	-0.278360	-1.901836	-0.877907
C	1.358415	-2.458503	-1.091090
C	1.837515	-2.556486	-2.414343
C	1.548442	-3.556652	-0.237776
C	2.529820	-3.703719	-2.818643
C	2.243968	-4.693426	-0.646765
H	1.137289	-3.517747	0.774338
C	2.742043	-4.768631	-1.945565
H	3.281366	-5.653243	-2.281895
H	2.907806	-3.760035	-3.841919
H	2.385645	-5.524151	0.045551
C	1.614581	-1.428923	-3.376410
H	1.962451	-1.669961	-4.389220
H	0.549404	-1.159631	-3.432284
H	2.134041	-0.516652	-3.047067

TS-1b-toluene_ortho2.log

SCF (wB97x) = -2687.83377004
H(0 K)= -2686.582361
H(298 K)= -2686.517273
G(298 K)= -2686.681995
Lowest Frequency = -734.6083cm-1

Pd	0.490130	-0.626392	-0.360995
P	2.492460	0.473669	0.297166
C	2.847295	2.029861	-0.653739
H	3.868062	2.361225	-0.392336
C	1.895741	3.184244	-0.333538
H	0.857637	2.877171	-0.558958
H	1.920359	3.427012	0.738682
C	2.263994	4.421020	-1.147426
H	1.584752	5.248404	-0.902130
H	3.275461	4.753094	-0.858531
C	2.240046	4.129822	-2.642349
H	2.521587	5.023318	-3.216974
H	1.207442	3.879251	-2.935815
C	3.151954	2.958742	-2.987158
H	4.200256	3.242556	-2.794220
H	3.089448	2.721540	-4.058138
C	2.808018	1.727828	-2.156587
H	3.481013	0.895432	-2.410019
H	1.791047	1.378981	-2.406203
C	3.981355	-0.569782	-0.131025
H	3.677418	-0.981775	-1.110693
C	5.347035	0.094938	-0.328705
H	5.716607	0.499322	0.626839
H	5.279242	0.946567	-1.019461
C	6.350436	-0.919549	-0.875371
H	6.014166	-1.242322	-1.874819
H	7.331326	-0.444627	-1.015693
C	6.469560	-2.141225	0.028409
H	6.893843	-1.831032	0.998062
H	7.175075	-2.866794	-0.398557
C	5.108492	-2.786641	0.261177
H	5.196839	-3.640854	0.946773
H	4.718717	-3.187476	-0.688259
C	4.110821	-1.772071	0.807000
H	3.127072	-2.239084	0.954687
H	4.450240	-1.426800	1.798938
C	2.627080	1.019172	2.068384
H	1.912632	1.861547	2.094942
C	2.070391	-0.026053	3.040306
H	1.089953	-0.367803	2.675964
H	2.723203	-0.913100	3.056110
C	1.961408	0.545769	4.449642
H	1.591321	-0.220553	5.144734
H	1.210826	1.353791	4.450171
C	3.296541	1.103618	4.931869
H	3.193985	1.539407	5.934947
H	4.019443	0.276679	5.026587
C	3.850207	2.135285	3.955269
H	4.820777	2.515398	4.302230
H	3.170053	3.003633	3.920156
C	3.976287	1.552108	2.550156
H	4.709662	0.729144	2.565236
H	4.371794	2.306881	1.853999

Al	-1.674322	0.154599	0.416755
N	-2.883176	1.690567	0.193578
N	-3.211720	-1.054415	0.668574
C	-4.192117	1.742082	0.471482
C	-4.912824	0.629711	0.919321
H	-5.956881	0.797471	1.166028
C	-4.469310	-0.699116	0.959362
C	-4.942803	3.028648	0.283680
H	-4.551571	3.818779	0.937364
H	-6.007709	2.902001	0.494831
H	-4.828778	3.401755	-0.741536
C	-5.480102	-1.752456	1.312919
H	-6.473579	-1.318190	1.452543
H	-5.195860	-2.271366	2.237616
H	-5.537986	-2.525929	0.537535
C	-2.264951	2.857480	-0.371082
C	-1.854633	3.914692	0.467363
C	-1.269533	5.036170	-0.122293
H	-0.942278	5.861771	0.509487
C	-1.088759	5.114791	-1.498723
H	-0.634168	6.000057	-1.941630
C	-1.463481	4.048801	-2.303815
H	-1.298261	4.101014	-3.380602
C	-2.046512	2.899832	-1.761630
C	-1.975141	3.821861	1.976087
H	-2.727747	3.056503	2.212774
C	-0.649008	3.348493	2.575423
H	-0.333952	2.389660	2.137343
H	-0.730159	3.220664	3.662821
H	0.148723	4.079372	2.379297
C	-2.427139	5.126753	2.624929
H	-1.667092	5.913038	2.534891
H	-2.608174	4.979307	3.696197
H	-3.351690	5.511332	2.175809
C	-2.402103	1.749201	-2.683368
H	-2.872073	0.958631	-2.079307
C	-1.141987	1.158792	-3.317189
H	-1.387866	0.301936	-3.958354
H	-0.441358	0.803998	-2.545139
H	-0.619958	1.902506	-3.936774
C	-3.416041	2.160428	-3.748817
H	-4.344335	2.540348	-3.303865
H	-3.673554	1.305199	-4.386481
H	-3.016152	2.946885	-4.402406
C	-2.937770	-2.460737	0.525792
C	-2.917592	-3.005921	-0.776066
C	-2.701411	-4.379808	-0.906661
H	-2.676122	-4.823848	-1.899817
C	-2.504411	-5.188524	0.206720
H	-2.341642	-6.257600	0.080758
C	-2.478327	-4.624288	1.474552
H	-2.278303	-5.254411	2.340772
C	-2.680256	-3.253899	1.658260
C	-3.126737	-2.130420	-1.998810
H	-2.648787	-1.161529	-1.779769
C	-2.445434	-2.672490	-3.249476
H	-2.923614	-3.590659	-3.615489
H	-1.382956	-2.884551	-3.074292
H	-2.513542	-1.935754	-4.059718

C	-4.606911	-1.858527	-2.271378
H	-5.157409	-2.796409	-2.428060
H	-4.724445	-1.249209	-3.177280
H	-5.089608	-1.315789	-1.449855
C	-2.518415	-2.638926	3.033192
H	-2.992235	-1.646458	3.023284
C	-1.027251	-2.432847	3.311102
H	-0.507883	-3.399503	3.369630
H	-0.866654	-1.901149	4.258561
H	-0.549415	-1.857255	2.504245
C	-3.173590	-3.451503	4.145383
H	-4.233927	-3.649043	3.942153
H	-3.107143	-2.918027	5.101096
H	-2.680609	-4.421647	4.286055
H	-0.362614	-1.679793	-1.227793
C	1.242985	-2.160276	-1.591461
C	1.744552	-1.892314	-2.873773
C	1.456338	-3.443863	-1.041829
C	2.501436	-2.834016	-3.572347
C	2.209219	-4.381759	-1.755222
C	2.742189	-4.084529	-3.008476
H	3.327653	-4.829769	-3.545247
H	2.898082	-2.590323	-4.558397
H	2.379890	-5.366552	-1.314589
H	1.554448	-0.918312	-3.330973
C	0.876906	-3.788765	0.297339
H	0.954464	-4.861082	0.521393
H	1.369795	-3.238387	1.114548
H	-0.183068	-3.500689	0.346691

TS-1b-toluene_para.log

SCF (wB97x) = -2687.82728207
H(0 K)= -2686.575796
H(298 K)= -2686.510489
G(298 K)= -2686.676683
Lowest Frequency = -752.6364cm-1

Pd	0.623739	-0.468669	-0.655038
P	2.096001	0.711748	0.819771
C	2.330238	2.478367	0.289201
H	3.156075	2.906984	0.883926
C	1.100518	3.361708	0.513694
H	0.243444	2.947890	-0.049633
H	0.803724	3.362134	1.572890
C	1.381643	4.791847	0.061809
H	0.497496	5.420452	0.233837
H	2.188345	5.212740	0.685552
C	1.800771	4.842341	-1.401804
H	2.017148	5.875408	-1.708104
H	0.955639	4.500658	-2.021540
C	3.001245	3.940329	-1.661611
H	3.881143	4.338268	-1.128403
H	3.261380	3.941302	-2.729250
C	2.730820	2.516957	-1.189352
H	3.602501	1.873604	-1.380833
H	1.904544	2.079760	-1.774675
C	3.840718	0.045947	0.708632
H	3.906084	-0.230500	-0.359053

C	5.017329	0.983084	0.999018
H	5.014307	1.283090	2.059192
H	4.938505	1.909799	0.414999
C	6.338687	0.291477	0.669435
H	6.362199	0.075290	-0.412413
H	7.181289	0.967450	0.870540
C	6.497509	-1.012130	1.441623
H	6.555893	-0.787266	2.519826
H	7.443944	-1.503534	1.177483
C	5.319679	-1.943783	1.184060
H	5.418546	-2.865745	1.773829
H	5.316534	-2.247069	0.125010
C	3.994607	-1.257959	1.498375
H	3.149929	-1.930973	1.285791
H	3.957687	-1.033821	2.577930
C	1.640738	0.893980	2.613318
H	0.738859	1.527425	2.535847
C	1.169750	-0.411053	3.263305
H	0.456909	-0.921732	2.596490
H	2.019456	-1.098024	3.390930
C	0.547720	-0.131691	4.628341
H	0.229837	-1.070258	5.103295
H	-0.367073	0.469598	4.489831
C	1.510346	0.622992	5.539120
H	1.036074	0.840889	6.505665
H	2.377773	-0.020962	5.758841
C	2.001681	1.907560	4.882441
H	2.714632	2.429063	5.535681
H	1.148489	2.593526	4.741892
C	2.631604	1.623385	3.521813
H	3.527597	0.995973	3.660974
H	2.977494	2.558772	3.057910
Al	-1.772379	-0.289434	-0.304602
N	-3.178465	0.989555	-0.779379
N	-3.103306	-1.742582	-0.180169
C	-4.481238	0.749449	-0.988114
C	-5.058775	-0.513039	-0.825920
H	-6.127364	-0.583642	-1.007105
C	-4.419356	-1.684266	-0.395563
C	-5.384370	1.881186	-1.384392
H	-5.449518	2.628725	-0.583002
H	-6.393906	1.526303	-1.607190
H	-4.993459	2.414145	-2.259662
C	-5.285689	-2.882668	-0.127031
H	-6.224390	-2.822291	-0.685679
H	-5.541575	-2.930441	0.940664
H	-4.782366	-3.823376	-0.369543
C	-2.721595	2.347156	-0.895412
C	-2.817799	3.201715	0.222473
C	-2.381293	4.520877	0.086344
H	-2.442042	5.193724	0.942069
C	-1.862790	4.986431	-1.116303
H	-1.532994	6.020561	-1.207385
C	-1.739770	4.119630	-2.193241
H	-1.307350	4.478693	-3.127877
C	-2.152069	2.787068	-2.104947
C	-3.313289	2.707872	1.569120
H	-3.819777	1.742513	1.419366
C	-2.122451	2.457976	2.498074

H	-1.418172	1.736734	2.056094
H	-2.451635	2.064985	3.469198
H	-1.567018	3.389218	2.680290
C	-4.315778	3.657491	2.219215
H	-3.854720	4.617427	2.484164
H	-4.710518	3.224278	3.146042
H	-5.165251	3.872780	1.559200
C	-1.951126	1.872519	-3.297020
H	-2.356727	0.882140	-3.043723
C	-0.460519	1.696192	-3.587148
H	-0.302336	1.003506	-4.423848
H	0.061350	1.282239	-2.710847
H	0.012338	2.654605	-3.847693
C	-2.694847	2.375283	-4.533126
H	-3.771469	2.482833	-4.350148
H	-2.564806	1.681248	-5.372233
H	-2.317276	3.354268	-4.856491
C	-2.516964	-2.958557	0.309578
C	-1.799096	-3.775863	-0.589269
C	-1.128565	-4.883543	-0.065566
H	-0.559718	-5.526568	-0.734426
C	-1.163189	-5.172930	1.293825
H	-0.625403	-6.037326	1.679771
C	-1.887375	-4.362417	2.157888
H	-1.912590	-4.596737	3.221894
C	-2.580356	-3.244602	1.686213
C	-1.792552	-3.487681	-2.079580
H	-1.751851	-2.393926	-2.200375
C	-0.580531	-4.057906	-2.804443
H	-0.613108	-5.154761	-2.857485
H	0.360895	-3.762482	-2.326785
H	-0.554680	-3.687635	-3.836040
C	-3.080124	-3.973968	-2.748822
H	-3.235168	-5.047505	-2.573550
H	-3.029306	-3.818008	-3.833702
H	-3.965864	-3.441578	-2.384582
C	-3.335095	-2.353871	2.656931
H	-4.025404	-1.719715	2.083484
C	-2.378589	-1.413982	3.387092
H	-1.631731	-1.981665	3.957679
H	-2.918301	-0.763981	4.088953
H	-1.828664	-0.771833	2.683056
C	-4.171984	-3.154122	3.651772
H	-4.835109	-3.867715	3.146738
H	-4.792205	-2.484452	4.259739
H	-3.543267	-3.726458	4.345373
H	0.361217	-1.349850	-1.977000
C	2.084677	-1.366517	-1.863216
C	2.784746	-0.672423	-2.861070
C	2.618923	-2.601608	-1.457257
C	4.002379	-1.140722	-3.356492
H	2.390034	0.272188	-3.240859
C	3.830354	-3.071546	-1.958061
H	2.086760	-3.197645	-0.711563
C	4.559463	-2.339048	-2.901877
H	4.536358	-0.558953	-4.111247
H	4.229248	-4.023116	-1.598332
C	5.898834	-2.812378	-3.381384
H	5.904590	-3.889443	-3.591034

H	6.209583	-2.293283	-4.295529
H	6.682729	-2.635810	-2.629406

TS-1b_TS1-PPdAl.log

SCF (wB97x) = -2648.51943408
H(0 K)= -2647.295750
H(298 K)= -2647.232494
G(298 K)= -2647.393313
Lowest Frequency = -741.9055cm-1

Pd	0.915453	0.200714	-1.097152
P	2.146685	-0.000362	0.923043
C	2.050181	1.561806	1.928306
H	2.747721	1.482118	2.781354
C	0.650535	1.832705	2.488942
H	-0.071187	1.854938	1.652313
H	0.330887	1.012625	3.150612
C	0.600792	3.159760	3.240841
H	-0.418912	3.344197	3.609710
H	1.248738	3.099259	4.131263
C	1.057017	4.316135	2.360487
H	1.026793	5.262014	2.919336
H	0.349127	4.424456	1.522634
C	2.448884	4.059364	1.799283
H	3.182257	4.032616	2.622863
H	2.757023	4.880323	1.137295
C	2.488133	2.737229	1.044535
H	3.487518	2.563474	0.619685
H	1.800473	2.783873	0.181696
C	3.978264	-0.142148	0.587580
H	4.089088	0.537299	-0.277794
C	4.979275	0.323622	1.649640
H	4.935757	-0.333007	2.532263
H	4.735219	1.334229	2.005011
C	6.395972	0.311792	1.079286
H	6.449393	1.039248	0.251917
H	7.114290	0.649626	1.839001
C	6.775408	-1.068026	0.555166
H	6.801075	-1.778749	1.398274
H	7.789508	-1.053673	0.133384
C	5.770710	-1.553933	-0.482788
H	6.030030	-2.564012	-0.829304
H	5.809903	-0.898846	-1.367360
C	4.351038	-1.536416	0.072584
H	3.631126	-1.857876	-0.696157
H	4.279240	-2.264625	0.898263
C	1.648459	-1.340057	2.115850
H	0.677174	-0.967119	2.491595
C	1.346064	-2.666246	1.403356
H	0.619617	-2.489520	0.593084
H	2.257391	-3.049938	0.919809
C	0.817654	-3.716918	2.375386
H	0.663085	-4.667445	1.847500
H	-0.177081	-3.406054	2.734112
C	1.742161	-3.905570	3.571869
H	1.327062	-4.648831	4.266220
H	2.708215	-4.310298	3.226875
C	1.986981	-2.581932	4.285218

H	2.657203	-2.719159	5.144827
H	1.032882	-2.204845	4.690587
C	2.564982	-1.549857	3.322416
H	3.547379	-1.906210	2.973230
H	2.748839	-0.596354	3.839874
Al	-1.468263	-0.016220	-0.696424
N	-2.942435	1.290895	-0.667131
N	-2.727374	-1.461959	-1.128827
C	-4.110768	1.151030	-1.307823
C	-4.530927	-0.062444	-1.869462
H	-5.486038	-0.051178	-2.385877
C	-3.928229	-1.315246	-1.696082
C	-5.047023	2.320022	-1.411019
H	-5.288513	2.722121	-0.419044
H	-5.977165	2.041214	-1.912763
H	-4.584923	3.144738	-1.968031
C	-4.694268	-2.528750	-2.133179
H	-5.589406	-2.254403	-2.697419
H	-5.004011	-3.127263	-1.266743
H	-4.071915	-3.190093	-2.748014
C	-2.706342	2.526151	0.031090
C	-2.940562	2.562800	1.423768
C	-2.790196	3.783930	2.085193
H	-2.977986	3.839678	3.155705
C	-2.391068	4.928848	1.405631
H	-2.278275	5.869804	1.941976
C	-2.094302	4.855335	0.052774
H	-1.730822	5.740754	-0.468878
C	-2.236051	3.659962	-0.657718
C	-3.330412	1.311055	2.190749
H	-2.752717	0.474779	1.755494
C	-2.966604	1.384317	3.669302
H	-1.920430	1.676975	3.819333
H	-3.114624	0.409045	4.146661
H	-3.598091	2.101333	4.209876
C	-4.812681	0.964113	2.037723
H	-5.443194	1.793340	2.386833
H	-5.064383	0.080483	2.640177
H	-5.087633	0.741548	1.000201
C	-1.802007	3.605952	-2.107922
H	-2.134000	2.648486	-2.534786
C	-0.273861	3.630769	-2.170773
H	0.084609	3.532722	-3.203484
H	0.153865	2.797630	-1.593427
H	0.119156	4.572081	-1.758474
C	-2.394275	4.730842	-2.953658
H	-3.490333	4.760707	-2.902694
H	-2.110558	4.608585	-4.005547
H	-2.026519	5.714033	-2.633020
C	-2.296031	-2.784589	-0.770953
C	-1.441282	-3.511370	-1.620506
C	-1.040950	-4.789338	-1.218082
H	-0.384750	-5.365043	-1.871344
C	-1.460053	-5.333672	-0.012186
H	-1.137992	-6.331883	0.280350
C	-2.278564	-4.589460	0.828212
H	-2.585015	-5.004054	1.789589
C	-2.705992	-3.309162	0.472660
C	-0.937596	-2.962620	-2.938742

H	-1.324398	-1.940444	-3.057466
C	0.586776	-2.874083	-2.940458
H	1.047904	-3.868348	-2.858037
H	0.943911	-2.254903	-2.102915
H	0.943561	-2.411775	-3.869332
C	-1.428630	-3.796383	-4.121774
H	-1.050214	-4.825895	-4.065914
H	-1.078910	-3.368050	-5.068854
H	-2.523807	-3.852875	-4.164295
C	-3.561110	-2.518261	1.444373
H	-3.835756	-1.564540	0.970760
C	-2.768019	-2.189519	2.708546
H	-2.459913	-3.104373	3.232809
H	-3.374942	-1.597698	3.406400
H	-1.856293	-1.617890	2.478312
C	-4.854256	-3.247996	1.802602
H	-5.451689	-3.485720	0.913940
H	-5.475376	-2.635782	2.468576
H	-4.650537	-4.193310	2.322375
H	0.804325	0.504562	-2.688155
C	2.460742	0.643624	-2.456122
C	2.966792	1.955412	-2.518104
C	3.231568	-0.362374	-3.064294
C	4.205397	2.235781	-3.093310
H	2.392694	2.775769	-2.085055
C	4.466660	-0.084186	-3.649152
H	2.872907	-1.392058	-3.062118
C	4.968479	1.215698	-3.658857
H	4.575052	3.261876	-3.100619
H	5.045114	-0.895315	-4.093668
H	5.934942	1.432817	-4.111222

TS-1c_TS1-PdP2.log

SCF (wB97x) = -2454.34266441
H(0 K)= -2453.270711
H(298 K)= -2453.223513
G(298 K)= -2453.350130
Lowest Frequency = -485.0241cm-1

Pd	0.050976	1.118739	-0.356669
P	-1.821491	-0.440433	-0.005967
P	2.220693	0.129011	-0.188140
C	2.465242	-0.581933	1.520491
C	3.735558	-1.374513	1.853932
C	2.216476	0.525045	2.556269
H	1.618216	-1.290251	1.604734
C	3.688095	-1.911881	3.282769
H	4.625715	-0.741579	1.735238
H	3.862440	-2.209529	1.153235
C	2.186973	-0.029787	3.975119
H	3.014570	1.282609	2.480905
H	1.276608	1.050883	2.317807
C	3.463837	-0.796215	4.295330
H	4.611946	-2.461993	3.507008
H	2.865574	-2.641748	3.364625
H	2.023462	0.782115	4.696925
H	1.325423	-0.710830	4.075138
H	3.426535	-1.202329	5.314911

H	4.320939	-0.103182	4.268362
C	3.430508	1.546172	-0.363594
C	4.862038	1.434867	0.172470
C	3.405744	2.042790	-1.815850
H	2.928995	2.327252	0.233861
C	5.618548	2.748911	-0.016338
H	5.410149	0.622361	-0.325999
H	4.846627	1.184350	1.241179
C	4.177543	3.346596	-1.976874
H	3.846423	1.281541	-2.480350
H	2.360529	2.171541	-2.134847
C	5.606862	3.208001	-1.468066
H	6.648234	2.643137	0.351395
H	5.145514	3.524576	0.607857
H	4.166965	3.663726	-3.028316
H	3.663310	4.138294	-1.408502
H	6.151447	4.155481	-1.576346
H	6.143267	2.469644	-2.087348
C	2.753935	-1.194359	-1.394688
C	4.246423	-1.442796	-1.642205
C	2.034449	-2.510373	-1.072071
H	2.329276	-0.828445	-2.347528
C	4.449614	-2.486965	-2.738043
H	4.735471	-1.781325	-0.716202
H	4.748879	-0.511752	-1.930605
C	2.244668	-3.546095	-2.170922
H	2.417244	-2.917819	-0.122338
H	0.964474	-2.318058	-0.910610
C	3.728215	-3.789356	-2.418439
H	5.522231	-2.665467	-2.892882
H	4.063129	-2.084466	-3.688715
H	1.740429	-4.486305	-1.910147
H	1.769900	-3.188615	-3.099875
H	3.870897	-4.518418	-3.227065
H	4.174408	-4.237647	-1.515262
C	-2.317333	0.007778	1.733226
C	-3.786136	-0.030999	2.162501
C	-1.402718	-0.684961	2.750233
H	-2.039631	1.073352	1.752660
C	-3.941823	0.559514	3.562647
H	-4.178799	-1.059863	2.151292
H	-4.396540	0.547140	1.454901
C	-1.585423	-0.094246	4.144051
H	-1.615097	-1.766054	2.785997
H	-0.354615	-0.587276	2.424755
C	-3.046561	-0.144253	4.575023
H	-4.993119	0.514258	3.877390
H	-3.674806	1.628839	3.525996
H	-0.949875	-0.621277	4.869301
H	-1.241389	0.954036	4.135429
H	-3.169337	0.299275	5.572092
H	-3.359434	-1.197969	4.664611
C	-1.775241	-2.307455	-0.053899
C	-2.892365	-3.101629	0.635161
C	-1.593484	-2.815373	-1.489933
H	-0.837394	-2.535429	0.489752
C	-2.587256	-4.597814	0.614132
H	-3.848641	-2.919228	0.124365
H	-3.029035	-2.767231	1.670691

C	-1.276362	-4.306334	-1.506306
H	-2.526063	-2.643427	-2.051051
H	-0.815773	-2.237969	-2.013136
C	-2.368089	-5.105797	-0.805561
H	-3.397098	-5.155199	1.104027
H	-1.678286	-4.785344	1.209616
H	-1.134073	-4.657372	-2.537752
H	-0.318219	-4.472117	-0.986199
H	-2.120553	-6.175672	-0.799043
H	-3.307723	-5.008536	-1.373627
C	-3.232203	0.120844	-1.084489
C	-2.760932	0.331446	-2.531326
C	-4.547972	-0.665238	-1.098306
H	-3.438236	1.127700	-0.672919
C	-3.810573	1.085533	-3.339171
H	-2.574514	-0.648447	-3.000310
H	-1.799327	0.863567	-2.537615
C	-5.611629	0.094319	-1.888393
H	-4.380458	-1.642623	-1.580546
H	-4.906927	-0.881212	-0.084865
C	-5.153835	0.364644	-3.316939
H	-3.466607	1.227926	-4.373081
H	-3.926836	2.091996	-2.905757
H	-6.557409	-0.464815	-1.885593
H	-5.813869	1.052144	-1.381218
H	-5.912444	0.943004	-3.861289
H	-5.057676	-0.596578	-3.848685
H	0.545691	2.639093	-0.453702
C	-1.170450	2.814264	-0.323612
C	-1.602575	3.328140	0.913677
C	-1.766323	3.360804	-1.473889
C	-2.648061	4.246428	1.004607
C	-2.806627	4.285492	-1.390654
C	-3.267937	4.721432	-0.149679
H	-1.423960	3.043404	-2.459953
H	-3.259214	4.669126	-2.306069
H	-4.082215	5.441343	-0.083932
H	-2.972752	4.599338	1.984268
H	-1.116806	2.991785	1.833017

TS-2a_TS2-PdAl2.log

SCF (wB97x) = -2842.69025423
H(0 K)= -2841.312092
H(298 K)= -2841.234287
G(298 K)= -2841.422495
Lowest Frequency = -74.3857cm-1

Pd	-0.085038	0.080999	0.337046
Al	-2.460189	-0.448331	0.378464
N	-3.389374	-1.967299	-0.464396
N	-4.176646	0.426358	0.760759
C	-4.687828	-2.270653	-0.372595
C	-5.627247	-1.410173	0.211654
H	-6.654829	-1.760893	0.237066
C	-5.398996	-0.112401	0.685383
C	-5.188767	-3.568233	-0.938476
H	-5.149349	-3.561994	-2.035644
H	-6.222128	-3.760261	-0.636941

H	-4.559359	-4.406879	-0.617714
C	-6.588629	0.694075	1.117245
H	-6.493793	1.007483	2.164758
H	-7.517963	0.129996	1.003096
H	-6.665307	1.620082	0.533692
C	-2.538565	-2.822314	-1.247011
C	-2.557192	-2.692921	-2.652813
C	-1.695595	-3.494842	-3.400083
H	-1.686158	-3.400469	-4.484950
C	-0.828230	-4.391365	-2.784360
H	-0.156027	-5.001327	-3.387012
C	-0.812724	-4.493594	-1.401783
H	-0.125164	-5.188357	-0.916509
C	-1.664310	-3.717132	-0.607542
C	-3.426488	-1.649689	-3.328251
H	-4.326382	-1.495373	-2.714243
C	-2.680860	-0.316417	-3.383243
H	-2.327680	0.001317	-2.390300
H	-3.324504	0.476926	-3.789104
H	-1.785805	-0.394094	-4.013971
C	-3.897532	-2.057603	-4.719832
H	-3.067691	-2.092498	-5.436969
H	-4.619323	-1.327877	-5.105559
H	-4.379520	-3.043712	-4.719913
C	-1.623957	-3.886299	0.896283
H	-2.335330	-3.176001	1.342988
C	-0.238361	-3.566558	1.446303
H	-0.219256	-3.668769	2.539068
H	0.062605	-2.536073	1.193618
H	0.520990	-4.245888	1.039181
C	-2.060477	-5.292815	1.306313
H	-3.068628	-5.532305	0.944645
H	-2.060653	-5.398022	2.398440
H	-1.377905	-6.052041	0.901572
C	-4.057025	1.790042	1.201243
C	-3.944643	2.075634	2.576331
C	-3.832435	3.411750	2.967962
H	-3.743784	3.646085	4.029248
C	-3.820843	4.439373	2.033694
H	-3.736443	5.474996	2.359467
C	-3.890425	4.135966	0.680674
H	-3.845095	4.938809	-0.056221
C	-3.998424	2.815525	0.237229
C	-3.885542	0.986032	3.628754
H	-4.109983	0.026343	3.141973
C	-2.471822	0.892243	4.207912
H	-2.203079	1.822641	4.727532
H	-1.725034	0.721652	3.420574
H	-2.399342	0.070878	4.932948
C	-4.911387	1.193369	4.741041
H	-4.710528	2.111831	5.307775
H	-4.882553	0.360134	5.453741
H	-5.934958	1.267237	4.351907
C	-3.984324	2.531264	-1.252572
H	-4.186832	1.459785	-1.402251
C	-2.593137	2.823129	-1.817852
H	-2.317956	3.875954	-1.659855
H	-2.547061	2.619431	-2.895534
H	-1.826787	2.202921	-1.330282

C	-5.058174	3.309488	-2.008577
H	-6.063041	3.111426	-1.615199
H	-5.052366	3.037148	-3.071463
H	-4.889315	4.392799	-1.950378
Al	2.192418	0.492061	0.703911
N	3.284602	2.023713	0.347302
N	3.634337	-0.596971	1.356705
C	4.467331	2.241973	0.932906
C	5.135469	1.263991	1.679880
H	6.082022	1.560014	2.121734
C	4.787974	-0.089137	1.808738
C	5.143102	3.568069	0.751977
H	4.433119	4.395379	0.866050
H	5.560554	3.658752	-0.259502
H	5.961793	3.693316	1.465546
C	5.784039	-0.993409	2.472577
H	5.400922	-1.362808	3.431544
H	6.727378	-0.473401	2.657081
H	5.980891	-1.881833	1.860714
C	2.786969	2.973242	-0.609531
C	3.345602	2.986474	-1.904953
C	2.781479	3.843419	-2.851057
H	3.182148	3.850606	-3.864785
C	1.708534	4.667534	-2.532118
H	1.280561	5.323572	-3.288528
C	1.186688	4.652129	-1.245743
H	0.351358	5.305307	-0.991339
C	1.708721	3.807746	-0.260951
C	4.493038	2.077433	-2.311096
H	4.937443	1.642741	-1.404640
C	3.995541	0.914677	-3.168791
H	3.528599	1.279512	-4.092351
H	3.236090	0.314601	-2.649537
H	4.829650	0.256903	-3.448511
C	5.599167	2.840189	-3.038861
H	6.459252	2.185103	-3.223499
H	5.951764	3.708546	-2.467192
H	5.260414	3.209712	-4.015073
C	1.126210	3.851748	1.138166
H	1.607567	3.068696	1.740600
C	1.426963	5.195229	1.804204
H	2.504326	5.400149	1.851705
H	1.034635	5.217233	2.828400
H	0.959996	6.022069	1.252127
C	-0.373218	3.570539	1.140676
H	-0.766852	3.555696	2.165499
H	-0.597825	2.590413	0.688599
H	-0.931661	4.337252	0.586037
C	3.410311	-2.011145	1.500234
C	3.005976	-2.536222	2.743439
C	2.872099	-3.922838	2.856220
H	2.560446	-4.347986	3.810665
C	3.113484	-4.760439	1.776944
H	3.009798	-5.838776	1.888281
C	3.453969	-4.218138	0.542590
H	3.606774	-4.879206	-0.308426
C	3.600988	-2.839420	0.372968
C	2.675821	-1.659122	3.937159
H	2.987365	-0.630012	3.708496

C	1.165858	-1.634185	4.183459
H	0.803193	-2.634846	4.458117
H	0.613795	-1.301577	3.295704
H	0.921374	-0.951320	5.007506
C	3.397799	-2.102406	5.209638
H	3.204773	-1.395445	6.025388
H	4.484852	-2.174974	5.078781
H	3.046785	-3.086477	5.546258
C	3.972537	-2.252899	-0.977375
H	3.361996	-1.342952	-1.116774
C	3.654637	-3.177247	-2.147384
H	4.353182	-4.024078	-2.196112
H	3.741444	-2.623506	-3.089818
H	2.635371	-3.579560	-2.093078
C	5.444444	-1.836448	-1.044949
H	6.100397	-2.686987	-0.812420
H	5.686803	-1.020880	-0.354490
H	5.695899	-1.491369	-2.055711
H	0.983334	0.605557	1.910653
C	0.623766	0.002313	-1.644773
C	0.859064	-1.226486	-2.292507
C	0.548931	1.136254	-2.481022
C	0.988736	-1.323738	-3.679160
H	0.912050	-2.140310	-1.695118
C	0.657404	1.046038	-3.868132
H	0.388422	2.121169	-2.038416
C	0.886777	-0.186379	-4.479794
H	1.160926	-2.300869	-4.135588
H	0.579885	1.952555	-4.472593
H	0.989052	-0.259068	-5.562480

TS-2b_TS2-PPdAl.log

SCF (wB97x) = -2648.53480595
H(0 K)= -2647.311421
H(298 K)= -2647.248681
G(298 K)= -2647.407463
Lowest Frequency = -45.0644cm-1

Pd	0.563176	-0.373011	-0.199580
P	2.944433	-0.415156	-0.172449
C	3.824339	-1.020376	-1.700753
H	4.916832	-0.960803	-1.547451
C	3.458964	-0.173750	-2.923436
H	2.358090	-0.149442	-3.014554
H	3.777706	0.869124	-2.783347
C	4.070230	-0.726764	-4.206674
H	3.771649	-0.104704	-5.061722
H	5.169011	-0.659834	-4.145118
C	3.670551	-2.178701	-4.433486
H	4.134162	-2.570129	-5.349160
H	2.580531	-2.231738	-4.588314
C	4.045997	-3.035796	-3.231629
H	5.144021	-3.062106	-3.132912
H	3.724978	-4.075844	-3.381775
C	3.437639	-2.484001	-1.947383
H	3.725651	-3.113402	-1.093276
H	2.337734	-2.539495	-2.008428
C	3.654536	-1.511159	1.164060

H	3.048092	-2.428354	1.040773
C	5.126478	-1.930959	1.084692
H	5.774894	-1.055520	1.238399
H	5.376527	-2.327120	0.090827
C	5.437059	-2.979688	2.151075
H	4.850146	-3.888231	1.936729
H	6.493983	-3.275184	2.095820
C	5.092042	-2.478912	3.549298
H	5.752699	-1.631952	3.798967
H	5.295352	-3.258028	4.296629
C	3.641158	-2.018415	3.629382
H	3.413799	-1.618189	4.626725
H	2.969266	-2.879842	3.484603
C	3.331954	-0.974419	2.562752
H	2.280240	-0.665461	2.620204
H	3.940280	-0.073683	2.754059
C	3.610816	1.311674	0.049580
H	3.475198	1.747766	-0.956521
C	2.731992	2.146761	0.991081
H	1.677272	2.067915	0.678068
H	2.774781	1.730469	2.010314
C	3.180489	3.603843	1.013723
H	2.561307	4.177032	1.717292
H	3.001687	4.046837	0.018672
C	4.658840	3.736482	1.360315
H	4.966562	4.791003	1.347545
H	4.823052	3.378635	2.390150
C	5.520194	2.916483	0.407105
H	6.583027	3.002567	0.671751
H	5.420060	3.322560	-0.613228
C	5.091895	1.451816	0.405523
H	5.266385	1.031133	1.408595
H	5.715159	0.867701	-0.288160
Al	-1.601794	0.184466	-0.777143
N	-3.229676	-0.751659	-1.081425
N	-2.372473	1.925255	-0.915897
C	-4.262419	-0.218590	-1.743925
C	-4.321404	1.140115	-2.088251
H	-5.181330	1.451564	-2.673486
C	-3.480494	2.165516	-1.624452
C	-5.425488	-1.091695	-2.103124
H	-6.004078	-1.351333	-1.206949
H	-5.089440	-2.041809	-2.534978
H	-6.096709	-0.591780	-2.806097
C	-3.871802	3.583090	-1.913525
H	-3.103685	4.080095	-2.519229
H	-3.951902	4.165103	-0.987256
H	-4.824397	3.635196	-2.446509
C	-3.320854	-2.082153	-0.542414
C	-2.641045	-3.136341	-1.180855
C	-2.684012	-4.402412	-0.590320
H	-2.163788	-5.228842	-1.075383
C	-3.367913	-4.621205	0.597798
H	-3.385095	-5.613703	1.044858
C	-4.022493	-3.564934	1.218445
H	-4.540450	-3.731563	2.163273
C	-4.013081	-2.281337	0.669781
C	-1.868106	-2.947328	-2.470593
H	-1.950565	-1.893603	-2.773261

C	-0.384047	-3.247166	-2.268933
H	0.042968	-2.612633	-1.475691
H	0.179211	-3.050506	-3.191185
H	-0.220137	-4.297834	-1.991704
C	-2.447921	-3.798976	-3.599349
H	-2.371534	-4.870000	-3.369678
H	-1.904294	-3.624082	-4.535810
H	-3.507553	-3.577432	-3.778309
C	-4.687247	-1.151598	1.424945
H	-4.647219	-0.242315	0.807019
C	-3.935169	-0.859007	2.723341
H	-4.400896	-0.021016	3.260070
H	-2.880994	-0.613534	2.541710
H	-3.948534	-1.730218	3.391500
C	-6.157222	-1.447471	1.718584
H	-6.727016	-1.682966	0.810663
H	-6.634340	-0.584477	2.199931
H	-6.264889	-2.301009	2.400434
C	-1.649382	3.015467	-0.317183
C	-1.892107	3.308618	1.044418
C	-1.193410	4.374218	1.616139
H	-1.366340	4.627025	2.659842
C	-0.267892	5.109410	0.883486
H	0.265188	5.935666	1.351394
C	-0.002914	4.766141	-0.433176
H	0.754576	5.315164	-0.993593
C	-0.677383	3.712871	-1.058180
C	-2.847770	2.468392	1.876193
H	-2.644564	1.411995	1.622039
C	-2.622952	2.615851	3.376990
H	-2.971854	3.591268	3.742844
H	-1.568513	2.506358	3.650799
H	-3.184259	1.844630	3.917096
C	-4.322277	2.742235	1.566921
H	-4.561464	3.803760	1.720127
H	-4.963640	2.160038	2.242397
H	-4.605083	2.473219	0.543877
C	-0.288619	3.332665	-2.473117
H	-0.950319	2.521888	-2.810784
C	1.142908	2.793997	-2.497153
H	1.859916	3.569386	-2.188978
H	1.418660	2.463037	-3.507143
H	1.253500	1.933960	-1.820945
C	-0.426356	4.501230	-3.448558
H	-1.437695	4.926845	-3.455480
H	-0.190738	4.180812	-4.470566
H	0.265300	5.315185	-3.194813
C	-0.200107	-0.773494	1.700296
C	-0.522243	-2.105093	2.036372
C	-0.213584	0.155632	2.758874
C	-0.774343	-2.495583	3.351710
H	-0.567039	-2.862860	1.248921
C	-0.470822	-0.226101	4.077412
H	0.013021	1.205252	2.549428
C	-0.745455	-1.558014	4.383960
H	-1.008581	-3.539501	3.568904
H	-0.457590	0.523300	4.871294
H	-0.946358	-1.859117	5.411480
H	-0.282303	0.003921	-1.909422

MesAlH2.log

SCF (wB97x) = -1006.60804023
H(0 K)= -1006.126309
H(298 K)= -1006.097358
G(298 K)= -1006.185227
Lowest Frequency = 15.8994cm-1

N	1.421984	0.497184	0.163916
C	1.257695	1.611641	0.878165
Al	-0.000029	-0.564356	-0.629308
H	0.000030	-2.015214	-0.024845
H	-0.000088	-0.410290	-2.194890
N	-1.421996	0.497164	0.163998
C	0.000012	2.150549	1.188362
H	0.000025	3.068983	1.767416
C	-1.257682	1.611627	0.878244
C	2.472539	2.326290	1.396465
H	3.154054	2.593830	0.579506
H	3.049839	1.680732	2.070467
H	2.201006	3.236264	1.937303
C	-2.472500	2.326287	1.396593
H	-3.153871	2.594136	0.579614
H	-2.200916	3.236097	1.937684
H	-3.049977	1.680641	2.070352
C	2.742344	-0.016103	-0.048368
C	3.453778	0.354208	-1.200041
C	4.716364	-0.204353	-1.407043
H	5.276397	0.082376	-2.298984
C	5.277713	-1.115727	-0.512380
C	4.542886	-1.461794	0.623581
H	4.964911	-2.170871	1.338249
C	3.277790	-0.930981	0.873992
C	-2.742357	-0.016111	-0.048310
C	-3.453798	0.354289	-1.199947
C	-4.716376	-0.204277	-1.406994
H	-5.276413	0.082506	-2.298915
C	-5.277714	-1.115732	-0.512410
C	-4.542857	-1.461927	0.623495
H	-4.964844	-2.171102	1.338088
C	-3.277767	-0.931115	0.873947
C	6.620909	-1.727909	-0.773891
H	7.198685	-1.852062	0.149629
H	6.526410	-2.725655	-1.223695
H	7.216346	-1.120169	-1.464282
C	-6.620961	-1.727808	-0.773914
H	-6.526568	-2.725753	-1.223292
H	-7.198938	-1.851458	0.149551
H	-7.216176	-1.120219	-1.464633
C	-2.500592	-1.314160	2.096273
H	-2.287830	-0.448030	2.738756
H	-3.044999	-2.048966	2.698554
H	-1.525507	-1.744416	1.830450
C	-2.870181	1.334471	-2.171225
H	-2.684221	2.312923	-1.706456
H	-1.900995	0.991033	-2.556087
H	-3.539241	1.493421	-3.023372
C	2.870157	1.334331	-2.171375
H	1.901024	0.990818	-2.556301

H	2.684091	2.312772	-1.706627
H	3.539262	1.493317	-3.023480
C	2.500657	-1.313871	2.096393
H	1.525556	-1.744144	1.830664
H	3.045081	-2.048608	2.698743
H	2.287940	-0.447656	2.738778

MesAlHPh.log

SCF (wB97x) = -1237.63036884
H(0 K)= -1237.065596
H(298 K)= -1237.031345
G(298 K)= -1237.132960
Lowest Frequency = 15.5419cm-1

N	1.523546	-1.199235	0.197296
C	1.417944	-2.432764	-0.316650
Al	0.030581	-0.332290	1.084876
H	-0.072141	-0.778812	2.588262
N	-1.317498	-1.306325	0.063799
C	0.194168	-3.076587	-0.539975
H	0.248494	-4.084955	-0.939268
C	-1.087458	-2.500484	-0.477537
C	2.668083	-3.162436	-0.718593
H	3.415768	-3.135996	0.083428
H	3.142073	-2.694234	-1.590387
H	2.453111	-4.204346	-0.969527
C	-2.223567	-3.254328	-1.105618
H	-3.080402	-3.334155	-0.426300
H	-1.916620	-4.258446	-1.409163
H	-2.590347	-2.725178	-1.994815
C	2.786347	-0.532090	0.099971
C	3.620302	-0.429263	1.221649
C	4.822449	0.271995	1.093031
H	5.476257	0.352943	1.963172
C	5.209920	0.865404	-0.106889
C	4.352414	0.749950	-1.205002
H	4.626601	1.221913	-2.150455
C	3.142642	0.063709	-1.124423
C	-2.621418	-0.722906	-0.044706
C	-3.556480	-0.923026	0.981610
C	-4.788904	-0.271419	0.892393
H	-5.519911	-0.422055	1.688796
C	-5.107115	0.565378	-0.176767
C	-4.153689	0.739228	-1.182670
H	-4.378307	1.396032	-2.025151
C	-2.909243	0.113184	-1.136730
C	6.509939	1.602351	-0.224449
H	7.219550	1.069865	-0.871731
H	6.373503	2.598693	-0.662894
H	6.991009	1.731272	0.751044
C	-6.430462	1.266561	-0.245870
H	-6.311283	2.357604	-0.220795
H	-6.966674	1.032325	-1.174389
H	-7.079803	0.987658	0.591033
C	-1.880999	0.368621	-2.194895
H	-1.398456	-0.553103	-2.546776
H	-2.319564	0.876679	-3.060293
H	-1.078064	1.010990	-1.802797

C	-3.232964	-1.807346	2.147645
H	-4.062346	-1.838725	2.861901
H	-3.021801	-2.840151	1.836875
H	-2.335382	-1.466842	2.680973
C	3.233926	-1.061214	2.524235
H	2.377587	-0.555908	2.990859
H	2.927811	-2.107715	2.397231
H	4.063238	-1.031725	3.238762
C	2.199899	0.016071	-2.288355
H	2.610801	0.546125	-3.153960
H	1.960929	-1.008773	-2.604684
H	1.239273	0.484085	-2.028101
C	-0.152027	1.603044	0.691288
C	0.807317	2.427992	0.075611
C	-1.385064	2.213478	0.997417
H	1.794139	2.027763	-0.165718
H	-2.171542	1.632903	1.488812
C	0.548370	3.761010	-0.241340
C	-1.662984	3.540427	0.676081
H	1.318562	4.366428	-0.719396
H	-2.637245	3.966821	0.915852
C	-0.693946	4.319149	0.048699
H	-0.903119	5.357581	-0.206425
